# Diastereoselective [3 + 2] Cycloaddition between Tertiary Amine *N*-Oxides and Substituted Alkenes to Access 7-Azanorbornanes

**DOI:** 10.1021/acs.orglett.4c02013

**Published:** 2024-07-22

**Authors:** Alexander H. Cocolas, Aiden M. Lane, Benjamin S. Musiak, Eric J. Chartier, Derek A. Bedillion, Sarah L. Hejnosz, Jeffrey J. Rohde, Paul A. Lummis, Jeffrey D. Evanseck, Thomas D. Montgomery

**Affiliations:** † Department of Chemistry and Biochemistry, Duquesne University, 600 Forbes Avenue, Pittsburgh, Pennsylvania 15282, United States; ‡ Department of Mathematics and Physical Sciences, Franciscan University of Steubenville, 1235 University Boulevard, Steubenville, Ohio 43952, United States

## Abstract

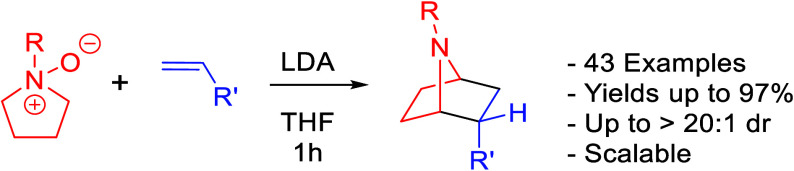

We have developed a diastereoselective synthesis of 43 novel 7-azanorbornanes using tertiary amine *N*-oxides and substituted alkenes. Our method uses an efficient [3 + 2] cycloaddition, starting from either commercially available or easily accessible precursors to generate yields up to 97% and diastereomeric ratios up to >20:1. Density functional theory (DFT) calculations were performed, suggesting that the observed diastereoselectivity is likely due to steric considerations.

Small lipophilic molecules with bridged stereocenters have gained popularity as potential drug targets.^1,2^ Moreover, motifs that exhibit spherical or highly rigid characteristics have demonstrated a high propensity to be more selective than primarily planar scafolds.^3−5^ 7-Azanorbornanes (7-azabicyclo[2.2.1]heptanes) generally score high in both of these metrics due to their biological and pharmacological relevance.^6−13^ Naturally occurring epibatidine (**1**), and its synthetic derivatives (**2** and **3**) have demonstrated biological relevance by binding to the α_4_β_2_ subunit of the nicotinic acetylcholine receptor (nAChR) at nanomolar concentrations (Scheme 1A).^14,15^ Recently, the Fujii group expanded the utility of 7-azanorbornanes, demonstrating agonism at the growth hormone secretagogue receptor (GHSR)^16^ along with the *κ-* and δ-opioid receptors.^9,10^

**Scheme 1:**
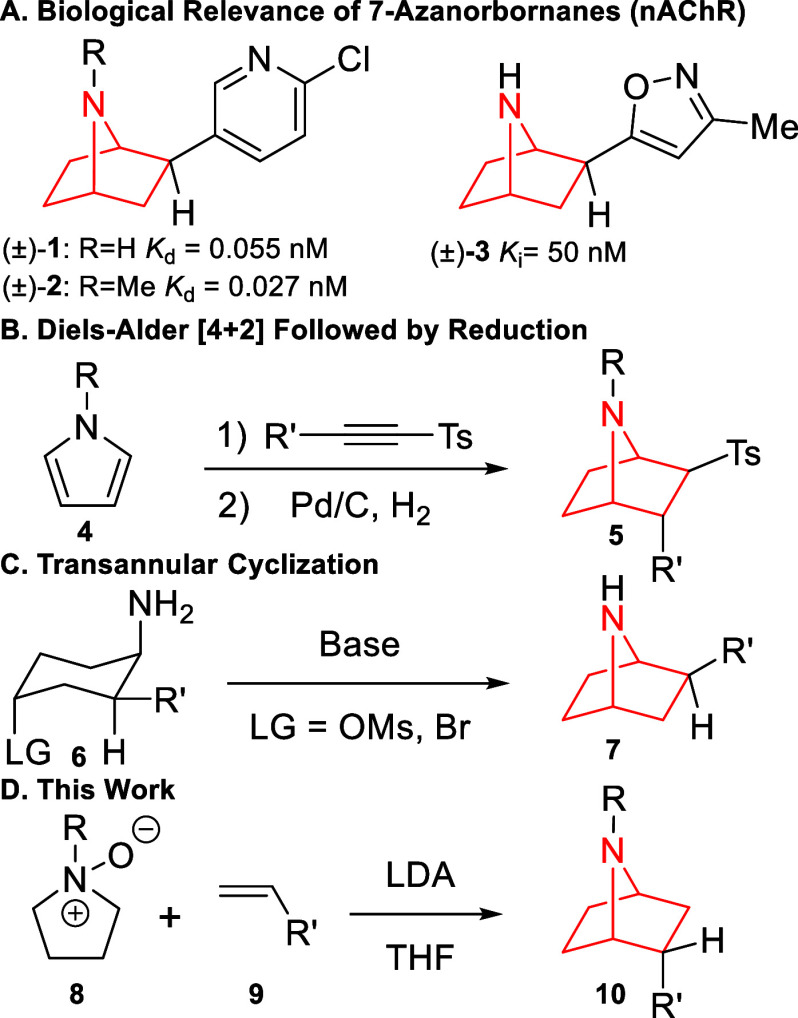
7-Azanorbornane Relevance and Syntheses

Given the importance of these scaffolds, several approaches have been employed to generate the 7-azanorbornane core. The most developed method utilizes a Diels–Alder [4 + 2] cycloaddition between *N*-protected pyrroles and alkynes, followed by hydrogenation (Scheme 1B).^17−21^ This method installs multiple substituents onto the 7-azanorbornane ring but requires 3 to 6 steps and frequently suffers from poor diastereoselectivity. Another effective tactic uses a leaving group-assisted intramolecular cyclization approach (Scheme 1C);^22−30^ this transannular route is more stereoselective but requires 6 to 11 steps to set the desired stereocenters and isolate the product. Armstrong (3-steps 36% overall yield)^31,32^ and Pandey (5-steps 40–46% overall yield)^33−35^ demonstrated alternative methods to generate *exo*-7-azanorbornanes with a limited substrate scope.

While these approaches are undoubtably effective at generating the bicyclic core, a more concise method that selectively generates *endo*-7-azanorbornanes remains absent in the literature. This work aims to fill this gap as *endo*-7-azanorbornanes can be isolated in an efficient and diastereoselective manner by coupling tertiary amine *N*-oxides with substituted alkenes in three total steps and up to 88% overall yield (Scheme 1D).

The use of tertiary amine *N*-oxides as precursors for [3 + 2] cycloadditions was originally reported by Roussi where they demonstrated the formation of various pyrrolidines.^36−41^ Later work by Davoren expanded this area through use of various stilbenes.^42^ Previously, we probed this chemistry computationally^43^ and established a synthetic method producing 1,2-diamines and imidazolidines by coupling tertiary amine *N*-oxides with silyl imines.^44^ We present here our most recent studies into the reaction of pyrrolidine *N*-oxides with substituted alkenes to give *endo*-7-azanorbornanes in good yields and high diastereoselectivities.

From the optimization of reaction conditions (Supporting Information (SI), Table S1), we observed an excellent level of conversion of **9a** to 7-azanorbornane **10a**. The major diastereomer (19:1 dr) could be either the *endo***10ea** or the *exo***11ea** (Scheme 2). Density functional theory (DFT)^45,46^ calculations produced the predicted distance from the benzylic proton to the nearest CH_3_ from the *tert*-butyl group (Figure 1A). A minimized ground state structure of **10aa** showed a minimum distance of 2.07 Å and an average distance of 3.04 Å, whereas for **11aa** these distances were 4.34 and 4.78 Å, respectively. A NOESY experiment showed a strong interaction between the well resolved *tert*-butyl and benzylic peaks, giving strong evidence for the assignment of **10aa** as the major stereoisomer. We performed similar analyses on compounds **10ab**, **10ea**, **10 lb**, and **10mb**, observing the same strong correlation in each. A crystal structure of **10mb** was obtained following the addition of tetrafluoroboric acid to generate **[10mb•H]BF**_**4**_ which confirmed the assigned diastereoselectivity (Figure 1B).

**Figure 1:**
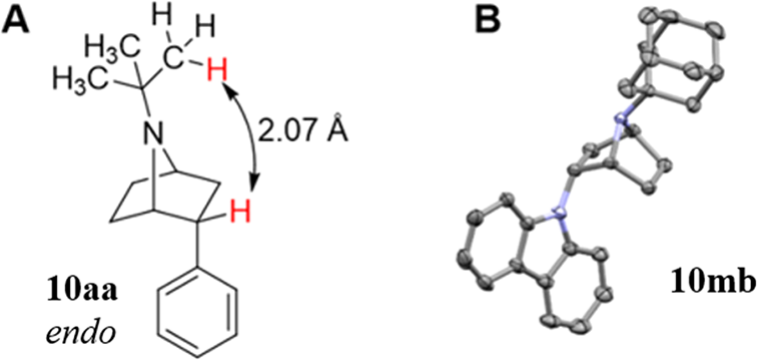
(A) Minimized ground state for **10aa** using M062x/jul-cc-pvTz. (B) X-ray structure of **[10mb•H]BF**_**4**_ (CD CCDC 2352512). Hydrogen atoms and BF_4_^–^ anion omitted for clarity.

With optimized conditions in hand, we examined the substrate scope (Scheme 2, SI, Scheme S1). To selectively generate 7-azanorboranes, pyrrolidine *N*-oxides containing *tert*-butyl, adamantyl, and *tert*-octyl were used. Our baseline reaction using styrene afforded good yields with an average isolated yield of 91% (**10a**); **10aa** was run on a gram scale, demonstrating scalability. *Para*-substituted alkyl groups (**10b** and **10c**) demonstrated an average of 87% isolated yield. Biphenyl **10d**, carbazole **10m**, and naphthyl **10n**, were well tolerated with modest to excellent yields. Electron-rich *para*-4-OMe **10e** afforded an average yield of 95%, whereas *meta*-4-OMe **10p** gave an average yield of 83%, presumably due to lower electronic contribution of the *meta* position. Phenol **10f** gave only modest yields, although this could be improved by using a silyl protecting group (**10g** and **10l**). Polysubstituted compounds **10k** and **10o** worked well, with the 2,4,6-trimethyl substrate **10o** having comparable isolated yields as the 4-Me (**10d**). Across these substrates we noticed a strong correlation between *ortho* substitution and improved diastereotopic ratios (**10c** and **10k** compared to **10l** and **10o**). As we observed in our prior work, electron-deficient substrates were tolerated but gave lower isolated yields (**10h** and **10i**). Reactions starting with 4-fluorostyrene showed significant degradation under the reaction conditions, partially explaining the poor yields. From the reaction mixture, along with expected **10h** we observed **10f** by ^1^H NMR and HRMS, showing a halogen to hydroxyl conversion. Examination of nonfluoro halogens showed similar issues and gave no meaningful amounts of halogenated products. Excitingly, boronic acid (**10j**) and alkyl (**10q**) containing substrates were also tolerated.

**Scheme 2:**
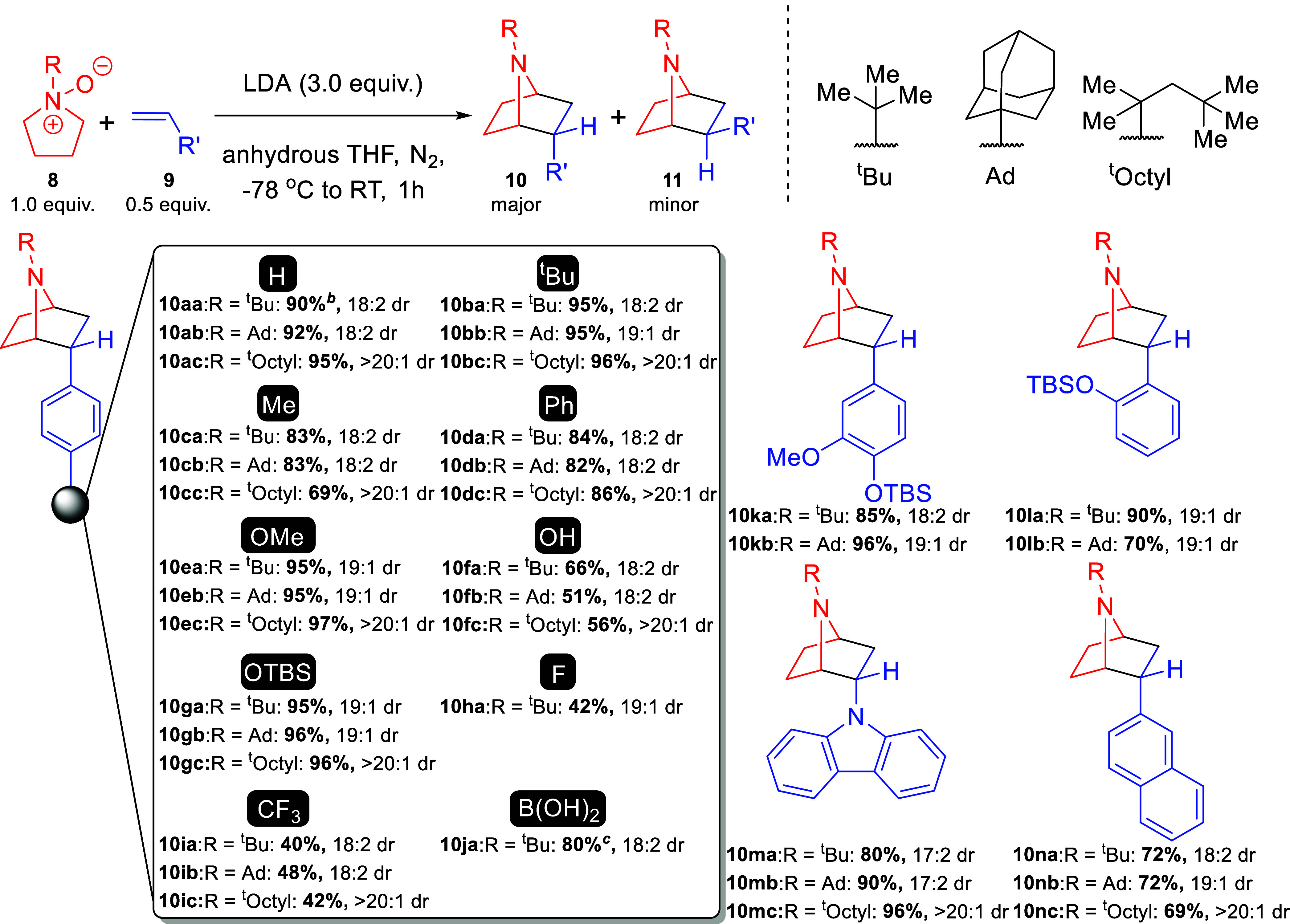
Substrate Scope Expanded substrate scope found in SI, Scheme S1. (a) Reactions carried on a 0.4 mmol scale. Conditions: *N*-oxide (1.0 equiv) alkene (0.5 equiv), LDA (3.0 equiv), dry THF (0.1M), −78 °C to RT, N_2_. Isolated yields were reported. (b) Carried out on a 7.0 mmol scale. (c) LDA (4.5 equiv).

To overcome the limitations of our substrate scope and demonstrate the versatility of these products, we subjected 7-azanorbornanes **10aa**, **10bc** and **10gb** to additional transformations (SI, Scheme S2). While strong electron withdrawing groups were not generally compatible with this chemistry, **10aa** was successfully nitrated (**S1**). **10gb** was subjected to TBAF deprotection followed by exposure to Tf_2_O installing the pseudohalogen^47^ triflate group (**S2**) in excellent yield over two steps. The *tert*-octyl *N*-protecting group was cleaved in the presence of BCl_3_, affording free amine (**S3**).

The mechanism of this reaction is expected to be analogous to our previous studies with generation of an azomethine ylide.^43,44^ Of greater interest to this work was an explanation for the diastereotopic selectivity for the *endo* product. We postulate that this is due to steric considerations since we saw a universal increase in d.r. going from ^*t*^Butyl to the more hindered ^*t*^Octyl functional group (Scheme 2). Additionally, *ortho*-substituted products (**10l** and **10o**) yielded some of the highest diastereomeric ratios. These experimental observations agreed with computational calculations performed using M06-2X^45^ with Dunning’s correlation consistent complete basis sets.^46,48^ Analysis of transition structures for both the *endo* and *exo* pathways predicted an 18:2 diastereomeric ratio for **10aa**, giving good agreement with experimental results (SI, Table S3).

In summary we developed a diastereoselective [3 + 2] cycloaddition between pyrrolidine *N*-oxides and substituted alkenes, generating 43 novel 7-azanorbornanes. This work makes use of easily accessible or commercially available starting materials. Finally, using both experimental observations and computational calculations we postulate a reasonable mechanism for the high diastereoselectivity.

## Data Availability

The data underlying this study are available in the published article and its online Supporting Information.

## Supporting Information Available

Detailed experimental procedures, compound characterization data, copies of NMR spectra for new compounds, and computational data (PDF)
FAIR Data, including the primary NMR FID files, for compound(s) **8a**–**8c**, **10aa**–**10qa**, ^t^Bu pyrrolidine, and ^t^octyl pyrrolidine (ZIP)
