# Supplementary figures and images for: Diastereoselective [3 + 2] Cycloaddition between Tertiary Amine N-Oxides and Substituted Alkenes to Access 7-Azanorbornanes
Source: Org Lett. 2024 Jul 22;26(31):6546–50. doi: 10.1021/acs.orglett.4c02013 (PMC11320637; doi:10.1021/acs.orglett.4c02013)

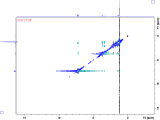

Supplement: Supplementary file 1 — ol4c02013_si_001.zip [file ol4c02013_si_001.zip › 10aa/10aa_NOSEY/pdata/1/thumb.png]

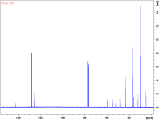

Supplement: Supplementary file 1 — ol4c02013_si_001.zip [file ol4c02013_si_001.zip › 10ab/10ab_13C/pdata/1/thumb.png]

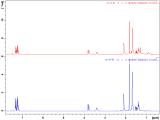

Supplement: Supplementary file 1 — ol4c02013_si_001.zip [file ol4c02013_si_001.zip › 10ab/10ab_1H/pdata/1/thumb.png]

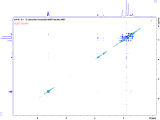

Supplement: Supplementary file 1 — ol4c02013_si_001.zip [file ol4c02013_si_001.zip › 10ab/10ab_NOESY/pdata/1/thumb.png]

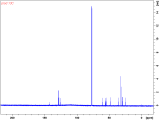

Supplement: Supplementary file 1 — ol4c02013_si_001.zip [file ol4c02013_si_001.zip › 10ac/10ac_13C/pdata/1/thumb.png]

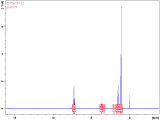

Supplement: Supplementary file 1 — ol4c02013_si_001.zip [file ol4c02013_si_001.zip › 10ac/10ac_1H/pdata/1/thumb.png]

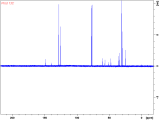

Supplement: Supplementary file 1 — ol4c02013_si_001.zip [file ol4c02013_si_001.zip › 10ba/10ba_13C/pdata/1/thumb.png]
