# Supplementary material for: Diastereoselective [3 + 2] Cycloaddition between Tertiary Amine N-Oxides and Substituted Alkenes to Access 7-Azanorbornanes
Source: Org Lett. 2024 Jul 22;26(31):6546–50. doi: 10.1021/acs.orglett.4c02013 (PMC11320637; doi:10.1021/acs.orglett.4c02013)
Supplement: Supplementary file 2 — ol4c02013_si_002.pdf [file ol4c02013_si_002.pdf]

Diastereoselective [3+2] cycloaddition between tertiary amine *N*-oxides and substituted alkenes to access 7-azanorbornanes

Alexander H. Cocolas, Aiden Lane, Benjamin S. Musiak, Eric J. Chartier, Derek A. Bedillion, Sarah L. Hejnosz, Paul A. Lummis, Jeffrey D. Evanseck, Thomas D. Montgomery*

* Corresponding author email: Montgomeryt1@duq.edu

Department of Chemistry and Biochemistry, Duquesne University, 600 Forbes Avenue, Pittsburgh, PA 15282

Supplementary Information

Contents

1.1 Scheme S1: Substrate Scope with Expanded Labels 8

1.2 Table S1: Optimization of Reaction Conditions[a] 9

1.3 Scheme S2: Additional Transformations for 7-Azanorbornanes 10

1.4 Experimental Section General Information 10

1.4.1 Materials 11

1.5 General synthetic procedures 11

1.5.1 General procedure for the preparation of *N*-substituted pyrrolidines.[3] 11

1.5.2 General procedure for the preparation of pyrrolidine *N*-oxides using *m*CPBA 11

1.5.3 General procedure for the preparation of pyrrolidine *N*-oxides using 30% H_2O_2 11

1-(*tert*-butyl)pyrrolidine 12

1-(*tert*-butyl)pyrrolidine 1-oxide (8a). 12

1-(adamantan-1-yl)pyrrolidine 12

1-(adamantan-1-yl)pyrrolidine 1-oxide (8b) 12

1-(2,4,4-trimethylpentan-2-yl)pyrrolidine 13

1-(2,4,4-trimethylpentan-2-yl)pyrrolidine 1-oxide (8c) 13

1.5.4 General procedure for -OTBS protected styrenes[5] 14

Tert-butyldimethyl(4-vinylphenoxy)silane (4-OTBS styrene) 14

Tert-butyldimethyl(2-vinylphenoxy)silane (2-OTBS styrene) 14

1.5.5 Procedure for OTBDPS protected alkene[7] 15

1.6 General procedure for the preparation of 7-azanorbornanes (10a-p) 15

7-(tert-butyl)-2-phenyl-7-azabicyclo[2.2.1]heptane (10aa) 16

Gram-scale synthesis of 7-(tert-butyl)-2-phenyl-7-azabicyclo[2.2.1]heptane (10aa) 16

7-(adamantan-1-yl)-2-phenyl-7-azabicyclo[2.2.1]heptane (10ab) 16

2-phenyl-7-(2,4,4-trimethylpentan-2-yl)-7-azabicyclo[2.2.1]heptane (10ac) 17

7-(*tert*-butyl)-2-(4-(*tert*-butyl)phenyl)-7-azabicyclo[2.2.1]heptane (10ba) 17

7-(adamantan-1-yl)-2-(4-(*tert*-butyl)phenyl)-7-azabicyclo[2.2.1]heptane (10bb) 18

2-phenyl-7-(2,4,4-trimethylpentan-2-yl)-7-azabicyclo[2.2.1]heptane (10bc) 18

7-(tert-butyl)-2-(*p*-tolyl)-7-azabicyclo[2.2.1]heptane (10ca) 19

7-(adamantan-1-yl)-2-(*p*-tolyl)-7-azabicyclo[2.2.1]heptane (10cb) 19

2-(*p*-tolyl)-7-(2,4,4-trimethylpentan-2-yl)-7-azabicyclo[2.2.1]heptane (10cc) 20

2-([1,1'-biphenyl]-4-yl)-7-(tert-butyl)-7-azabicyclo[2.2.1]heptane (10da) 20

2-([1,1'-biphenyl]-4-yl)-7-(adamantan-1-yl)-7-azabicyclo[2.2.1]heptane (10db) 21

2-([1,1'-biphenyl]-4-yl)-7-(2,4,4-trimethylpentan-2-yl)-7-azabicyclo[2.2.1]heptane (10dc) 21

7-(tert-butyl)-2-(4-methoxyphenyl)-7-azabicyclo[2.2.1]heptane (10ea) 22

7-(adamantan-1-yl)-2-(4-methoxyphenyl)-7-azabicyclo[2.2.1]heptane (10eb) 22
2-(4-methoxyphenyl)-7-(2,4,4-trimethylpentan-2-yl)-7-azabicyclo[2.2.1]heptane (10ec) 23
4-(-7-(*tert*-butyl)-7-azabicyclo[2.2.1]heptan-2-yl)phenol (10fa) 23
4-(-7-adamantan-1-yl)-7-azabicyclo[2.2.1]heptan-2-yl)phenol (10fb) 24
4-(-7-(2,4,4-trimethylpentan-2-yl)-7-azabicyclo[2.2.1]heptan-2-yl)phenol (10fc) 24
7-(*tert*-butyl)-2-(4-((*tert*-butyldimethylsilyl)oxy)phenyl)-7-azabicyclo[2.2.1]heptane (10ga) 25
7-(adamantan-1-yl)-2-(4-((*tert*-butyldimethylsilyl)oxy)phenyl)-7-azabicyclo[2.2.1]heptane (10gb) 25
2-(4-((*tert*-butyldimethylsilyl)oxy)phenyl)-7-(2,4,4-trimethylpentan-2-yl)-7-azabicyclo[2.2.1]heptane (10gc) 26
7-(*tert*-butyl)-2-(4-fluorophenyl)-7-azabicyclo[2.2.1]heptane (10ha) 26
7-(*tert*-butyl)-2-(4-(trifluoromethyl)phenyl)-7-azabicyclo[2.2.1]heptane (10ia) 27
7-(adamantan-1-yl)-2-(4-(trifluoromethyl)phenyl)-7-azabicyclo[2.2.1]heptane (10ib) 27
2-(4-(trifluoromethyl)phenyl)-7-(2,4,4-trimethylpentan-2-yl)-7-azabicyclo[2.2.1]heptane (10ic) 28
4-(-7-*tert*-butyl)-7-azabicyclo[2.2.1]heptan-2-yl)phenyl)boronic acid (10ja) 28
7-(*tert*-butyl)-2-(4-((*tert*-butyldimethylsilyl)oxy)-3-methoxyphenyl)-7-azabicyclo[2.2.1]heptane (10ka) 29
7-(adamantan-1-yl)-2-(4-((tert-butyldimethylsilyl)oxy)-3-methoxyphenyl)-7-azabicyclo[2.2.1]heptane (10kb) 29
7-(*tert*-butyl)-2-(2-((*tert*-butyldimethylsilyl)oxy)phenyl)-7-azabicyclo[2.2.1]heptane (10la) 30
7-(adamantan-1-yl)-2-(2-((*tert*-butyldimethylsilyl)oxy)phenyl)-7-azabicyclo[2.2.1]heptane (10lb) 30
9-(4-(-7-(tert-butyl)-7-azabicyclo[2.2.1]heptan-2-yl)phenyl)-9H-carbazole (10ma) 31
9-(4-(-7-(adamantan-1-yl)-7-azabicyclo[2.2.1]heptan-2-yl)phenyl)-9H-carbazole (10mb) 31
9-(4-(-7-(2,4,4-trimethylpentan-2-yl)-7-azabicyclo[2.2.1]heptan-2-yl)phenyl)-9H-carbazole (10mc) 32
7-(*tert*-butyl)-2-(naphthalen-2-yl)-7-azabicyclo[2.2.1]heptane (10na) 32
7-(adamantan-1-yl)-2-(naphthalen-2-yl)-7-azabicyclo[2.2.1]heptane (10nb) 33
2-(naphthalen-2-yl)-7-(2,4,4-trimethylpentan-2-yl)-7-azabicyclo[2.2.1]heptane (10nc) 33
7-(*tert*-butyl)-2-mesityl-7-azabicyclo[2.2.1]heptane (10oa) 34
7-(adamantan-1-yl)-2-mesityl-7-azabicyclo[2.2.1]heptane (10ob) 34
2-mesityl-7-(2,4,4-trimethylpentan-2-yl)-7-azabicyclo[2.2.1]heptane (10oc) 35
7-(*tert*-butyl)-2-(3-methoxyphenyl)-7-azabicyclo[2.2.1]heptane (10pa) 35
7-(adamantan-1-yl)-2-(3-methoxyphenyl)-7-azabicyclo[2.2.1]heptane (10pb) 36
2-(3-methoxyphenyl)-7-(2,4,4-trimethylpentan-2-yl)-7-azabicyclo[2.2.1]heptane (10pc) 36
7-(tert-butyl)-2-(((tert-butyldiphenylsilyl)oxy)methyl)-7-azabicyclo[2.2.1]heptane (10qa) 37
7-(*tert*-butyl)-2-(4-nitrophenyl)-7-azabicyclo[2.2.1]heptane (S1) 37
4-7-(adamantan-1-yl)-7-azabicyclo[2.2.1]heptan-2-yl)phenyl trifluoromethanesulfonate (S2) 38

2-(4-(tert-butyl)phenyl)-7-azabicyclo[2.2.1]heptane (S3)[8] 39
2.1 Experimental data 40
Figure S1: 8a ^{1}H NMR, 400 MHz, $CDCl_3$ 40
Figure S2: 8a ^{13}C NMR, 126 MHz, $CDCl_3$ 40
Figure S3: 8b ^{1}H NMR, 400 MHz, $CDCl_3$ 41
Figure S4: 8b ^{13}C NMR, 126 MHz, $CDCl_3$ 41
Figure S5: 1-(2,4,4-trimethylpentan-2-yl)pyrrolidine ^{1}H NMR, 500 MHz, $CDCl_3$ 42
Figure S6: 1-(2,4,4-trimethylpentan-2-yl)pyrrolidine ^{13}C NMR, 126 MHz, $CDCl_3$ 42
Figure S7: 8c ^{1}H NMR, 500 MHz, $CDCl_3$ 43
Figure S8: 8c ^{13}C NMR, 126 MHz, $CDCl_3$ 43
Figure S9: 10aa ^{1}H NMR, 400 MHz, $CDCl_3$ 44
Figure S10: 10aa ^{13}C NMR, 126 MHz, $CDCl_3$ 44
Figure S11: 10aa NOESY, 400 MHz, $CDCl_3$ 45
Figure S12: 10ab ^{1}H NMR, 400 MHz, $CDCl_3$ 46
Figure S13: 10ab ^{13}C NMR, 126 MHz, $CDCl_3$ 46
Figure S14: 10ab NOESY, 400 MHz, $CDCl_3$ 47
Figure S15: 10ac ^{1}H NMR, 400 MHz, $CDCl_3$ 48
Figure S16: 10ac ^{13}C NMR, 126 MHz, $CDCl_3$ 48
Figure S17: 10ba ^{1}H NMR, 400 MHz, $CDCl_3$ 49
Figure S18: 10ba ^{13}C NMR, 126 MHz, $CDCl_3$ 49
Figure S19: 10bb ^{1}H NMR, 500 MHz, $CDCl_3$ 50
Figure S20: 10bb ^{13}C NMR, 126 MHz, $CDCl_3$ 50
Figure S21: 10bc ^{1}H NMR, 500 MHz, $CDCl_3$ 51
Figure S22: 10bc ^{13}C NMR, 126 MHz, $CDCl_3$ 51
Figure S23: 10ca ^{1}H NMR, 400 MHz, $CDCl_3$ 52
Figure S24: 10ca ^{13}C NMR, 126 MHz, $CDCl_3$ 52
Figure S25: 10cb ^{1}H NMR, 400 MHz, $CDCl_3$ 53
Figure S26: 10cb ^{13}C NMR, 126 MHz, $CDCl_3$ 53
Figure S27: 10cc ^{1}H NMR, 400 MHz, $CDCl_3$ 54
Figure S28: 10cc ^{13}C NMR, 126 MHz, $CDCl_3$ 54
Figure S29: 10da ^{1}H NMR, 400 MHz, $CDCl_3$ 55
Figure S30: 10da ^{13}C NMR, 126 MHz, $CDCl_3$ 55
Figure S31: 10db ^{1}H NMR, 400 MHz, $CDCl_3$ 56
Figure S32: 10db ^{13}C NMR, 125 MHz, $CDCl_3$ 56
Figure S33: 10dc ^{1}H NMR, 400 MHz, $CDCl_3$ 57

Figure S34: 10dc ^{13}C NMR, 126 MHz, $CDCl_3$ 57
Figure S35: 10ea ^{1}H NMR, 400 MHz, $CDCl_3$ 58
Figure S36: 10ea ^{13}C NMR, 126 MHz, $CDCl_3$ 58
Figure S37: 10ea NOESY, 400 MHz, $CDCl_3$.......... 59
Figure S38: 10eb ^{1}H NMR, 400 MHz, $CDCl_3$ 60
Figure S39: 10eb ^{13}C NMR, 125 MHz, $CDCl_3$ 60
Figure S40: 10ec ^{1}H NMR, 400 MHz, $CDCl_3$ 61
Figure S41: 10ec ^{13}C NMR, 126 MHz, $CDCl_3$ 61
Figure S42: 10fa ^{1}H NMR, 400 MHz, $CDCl_3$.......... 62
Figure S43: 10fa ^{13}C NMR, 126 MHz, $CDCl_3$.......... 62
Figure S44: 10fb ^{1}H NMR, 400 MHz, $CDCl_3$ 63
Figure S45: 10fb ^{13}C NMR, 126 MHz, $CDCl_3$ 63
Figure S46: 10fc ^{1}H NMR, 400 MHz, $CDCl_3$.......... 64
Figure S47: 10fc ^{13}C NMR, 126 MHz, $CDCl_3$.......... 64
Figure S48: 10ga ^{1}H NMR, 400 MHz, $CDCl_3$ 65
Figure S49: 10ga ^{13}C NMR, 126 MHz, $CDCl_3$ 65
Figure S50: 10gb ^{1}H NMR, 400 MHz, $CDCl_3$.......... 66
Figure S51: 10gb ^{13}C NMR, 101 MHz, $CDCl_3$.......... 66
Figure S52: 10gc ^{1}H NMR, 400 MHz, $CDCl_3$ 67
Figure S53: 10gc ^{13}C NMR, 126 MHz, $CDCl_3$ 67
Figure S54: 10ha ^{1}H NMR, 400 MHz, $CDCl_3$ 68
Figure S55: 10ha ^{13}C NMR, 126 MHz, $CDCl_3$ 68
Figure S56: 10ha ^{19}F NMR, 376 MHz, $CDCl_3$ 69
Figure S57: 10ia ^{1}H NMR, 400 MHz, $CDCl_3$.......... 70
Figure S58: 10ia ^{13}C NMR, 101 MHz, $CDCl_3$.......... 70
Figure S59: 10ia ^{19}F NMR, 376 MHz, $CDCl_3$ 71
Figure S60: 10ib ^{1}H NMR, 400 MHz, $CDCl_3$.......... 72
Figure S61: 10ib ^{13}C NMR, 101 MHz, $CDCl_3$.......... 72
Figure S62: 10ib ^{19}F NMR, 376 MHz, $CDCl_3$ 73
Figure S63: 10ic ^{1}H NMR, 400 MHz, $CDCl_3$.......... 74
Figure S64: 10ic ^{13}C NMR, 126 MHz, $CDCl_3$.......... 74
Figure S65: 10ic ^{19}F NMR, 376 MHz, $CDCl_3$ 75
Figure S66: 10ja ^{1}H NMR, 400 MHz, $CDCl_3$.......... 76
Figure S67: 10ja ^{13}C NMR, 126 MHz, $CDCl_3$.......... 76
Figure S68: 10ka ^{1}H NMR, 400 MHz, $CDCl_3$.......... 77

Figure S69: 10ka ^{13}C NMR, 126 MHz, $CDCl_3$ 77
Figure S70: 10kb ^{1}H NMR, 400 MHz, $CDCl_3$ 78
Figure S71: 10kb ^{13}C NMR, 126 MHz, $CDCl_3$ 78
Figure S72: 10la ^{1}H NMR, 400 MHz, $CDCl_3$ 79
Figure S73: 10la ^{13}C NMR, 126 MHz, $CDCl_3$ 79
Figure S74: 10lb ^{1}H NMR, 400 MHz, $CDCl_3$ 80
Figure S75: 10lb ^{13}C NMR, 126 MHz, $CDCl_3$ 80
Figure S76: 10lb NOESY, 400 MHz, $CDCl_3$ 81
Figure S77: 10ma ^{1}H NMR, 400 MHz, $CDCl_3$ 82
Figure S78: 10ma ^{13}C NMR, 126 MHz, $CDCl_3$ 82
Figure S79: 10mb ^{1}H NMR, 400 MHz, $CDCl_3$ 83
Figure S80: 10mb ^{13}C NMR, 101 MHz, $CDCl_3$ 83
Figure S81: 10mb NOESY, 500 MHz, $CDCl_3$ 84
Figure S82: 10mc ^{1}H NMR, 400 MHz, $CDCl_3$ 85
Figure S83: 10mc ^{13}C NMR, 126 MHz, $CDCl_3$ 85
Figure S84: 10na ^{1}H NMR, 400 MHz, $CDCl_3$ 86
Figure S85: 10na ^{13}C NMR, 126 MHz, $CDCl_3$ 86
Figure S86: 10nb ^{1}H NMR, 400 MHz, $CDCl_3$ 87
Figure S87: 10nb ^{13}C NMR, 126 MHz, $CDCl_3$ 87
Figure S88: 10nc ^{1}H NMR, 400 MHz, $CDCl_3$ 88
Figure S89: 10nc ^{13}C NMR, 126 MHz, $CDCl_3$ 88
Figure S90: 10oa ^{1}H NMR, 400 MHz, $CDCl_3$ 89
Figure S91: 10oa ^{13}C NMR, 126 MHz, $CDCl_3$ 89
Figure S92: 10ob ^{1}H NMR, 400 MHz, $CDCl_3$ 90
Figure S93: 10ob ^{13}C NMR, 126 MHz, $CDCl_3$ 90
Figure S94: 10oc ^{1}H NMR, 400 MHz, $CDCl_3$ 91
Figure S95: 10oc ^{13}C NMR, 126 MHz, $CDCl_3$ 91
Figure S96: 10pa ^{1}H NMR, 400 MHz, $CDCl_3$ 92
Figure S97: 10pa ^{13}C NMR, 126 MHz, $CDCl_3$ 92
Figure S98: 10pb ^{1}H NMR, 400 MHz, $CDCl_3$ 93
Figure S99: 10pb ^{13}C NMR, 101 MHz, $CDCl_3$ 93
Figure S100: 10pc ^{1}H NMR, 400 MHz, $CDCl_3$ 94
Figure S101: 10pc ^{13}C NMR, 126 MHz, $CDCl_3$ 94
Figure S102: 10qa ^{1}H NMR, 400 MHz, $CDCl_3$ 95
Figure S103: 10qa ^{13}C NMR, 125 MHz, $CDCl_3$ 95

Figure S104: S1 ^{1}H NMR, 400 MHz, $CDCl_3$ 96
Figure S105: S1 ^{13}C NMR, 126 MHz, $CDCl_3$ 96
Figure S106: S2 ^{1}H NMR, 400 MHz, $CDCl_3$ 97
Figure S107: S2 ^{13}C NMR, 126 MHz, $CDCl_3$ 97
Figure S108: S2 ^{19}F NMR, 376 MHz, $CDCl_3$ 98
Figure S109: S3 ^{1}H NMR, 400 MHz, $CDCl_3$ 99
Figure S110: S3 ^{13}C NMR, 125 MHz, $CDCl_3$ 99
2.2 Single Crystal X-Ray crystallography 100
Figure S111. Molecular structure of (r)-[10mb•H]BF_4 with thermal ellipsoids shown at the 30% probability level. All carbon-bound hydrogen atoms are omitted for clarity. 101
Figure S112. Molecular structure of (s)-[10mb•H]BF4 with thermal ellipsoids shown at the 30% probability level 102
Figure S113. Packing diagram of (rac)-[10mb•H]BF4 with thermal ellipsoids shown at the 30% probability level. 103
Table S2. Bond lengths for (rac)-[10mb•H]BF_4 104
3.1 Computational Calculations 105
Table S3: Calculated energies for the [3+2] transition state between styrene and tert-butyl pyrrolidine *N*-oxide 105
3.1.1 Computational Outputs 106
Figure S114: Ground state of 10aa M062x-cc-pvTz (pcm = THF) 114
Figure S115: Ground state of 11aa M062x-cc-pvTz (pcm = THF) 116
Figure S115: (A) Endo (10aa) transition structure. (B) Exo (11aa) transition structure. 118
Figure S116. Shared steric interactions for both endo and exo shown in red. 118
Figure S117. (a) Three t-butyl pyrrolidine ylide categories for steric analysis: 119
Table S4. Endo transition structure unshared steric interactions (kcal/mol) 119
Table S5. Exo transition structure unshared steric interactions (kcal/mol) 119
Table S6. Difference in steric interactions (kcal/mol) [exo-endo] 119
Figure S118. Major steric effects unique to exo transition structure shown in red. 120

1.1 Scheme S1: Substrate Scope with Expanded Labels

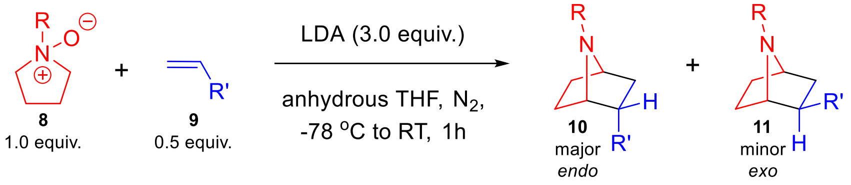

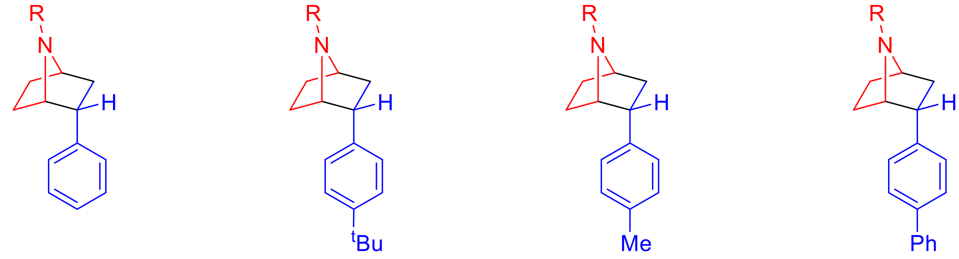

10aa:R = ^{t}Bu: **90%[b]**, 18:2 dr
10ab:R = Ad: **92%,** 18:2 dr
10ac:R = ^{t}Octyl: **95%,** > 20:1 dr

10ba:R = ^{t}Bu: **95%,** 18:2 dr
10bb:R = Ad: **95%,** 19:1 dr
10bc:R = ^{t}Octyl: **96%,** > 20:1 dr

10ca:R = ^{t}Bu: **83%,** 18:2 dr
10cb:R = Ad: **83%,** 18:2 dr
10cc:R = ^{t}Octyl: **69%,** > 20:1 dr

10da:R = ^{t}Bu: **84%,** 18:2 dr
10db:R = Ad: **82%,** 18:2 dr
10dc:R = ^{t}Octyl: **86%,** > 20:1 dr

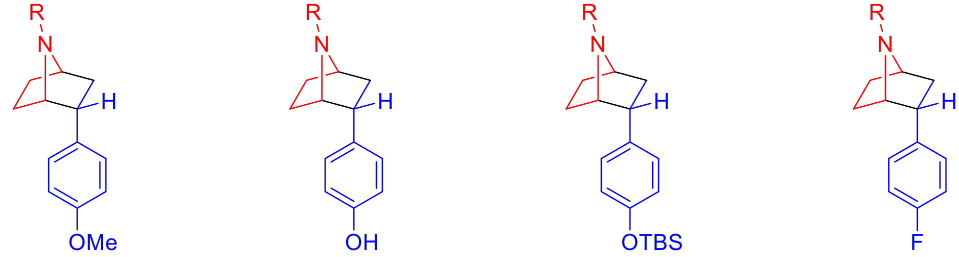

10ea:R = ^{t}Bu: **95%,** 19:1 dr
10eb:R = Ad: **95%,** 19:1 dr
10ec:R = ^{t}Octyl: **97%,** > 20:1 dr

10fa:R = ^{t}Bu: **66%,** 18:2 dr
10fb:R = Ad: **51%,** 18:2 dr
10fc:R = ^{t}Octyl: **56%,** > 20:1 dr

10ga:R = ^{t}Bu: **95%,** 19:1 dr
10gb:R = Ad: **96%,** 19:1 dr
10gc:R = ^{t}Octyl: **96%,** > 20:1 dr

10ha:R = ^{t}Bu: **42%,** 19:1 dr

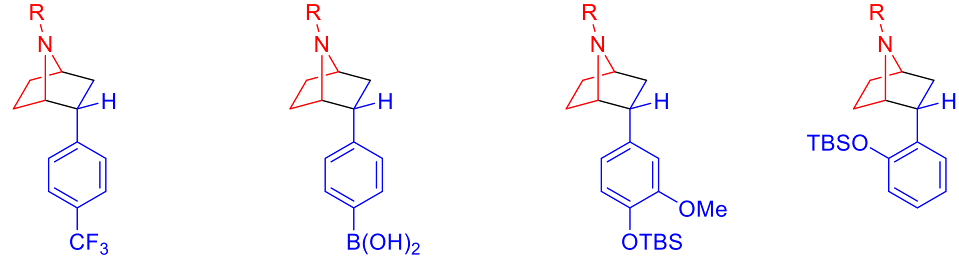

10ia:R = ^{t}Bu: **40%,** 18:2 dr
10ib:R = Ad: **48%,** 18:2 dr
10ic:R = ^{t}Octyl: **42%,** > 20:1 dr

10ja:R = ^{t}Bu: **80%[c]**, 18:2 dr

10ka:R = ^{t}Bu: **85%,** 18:2 dr
10kb:R = Ad: **96%,** 19:1 dr

10la:R = ^{t}Bu: **90%,** 19:1 dr
10lb:R = Ad: **70%,** 19:1 dr

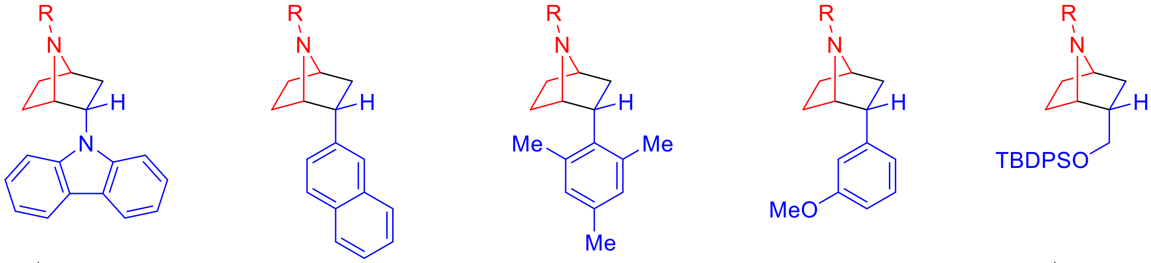

10ma:R = ^{t}Bu: **80%,** 17:2 dr
10mb:R = Ad: **90%,** 17:2 dr
10mc:R = ^{t}Octyl: **96%,** > 20:1 dr

10na:R = ^{t}Bu: **72%,** 18:2 dr
10nb:R = Ad: **72%,** 19:1 dr
10nc:R = ^{t}Octyl: **69%,** > 20:1 dr

10oa:R = ^{t}Bu: **80%,** 20:1 dr
10ob:R = Ad: **82%,** 20:1 dr
10oc:R = ^{t}Octyl: **94%,** > 20:1 dr

10pa:R = ^{t}Bu: **82%,** 18:2 dr
10pb:R = Ad: **86%,** 19:1 dr
10pc:R = ^{t}Octyl: **80%,** > 20:1 dr

10qa:R = ^{t}Bu: **75%,**19:1 dr

1.2 Table S1: Optimization of Reaction Conditions[a]

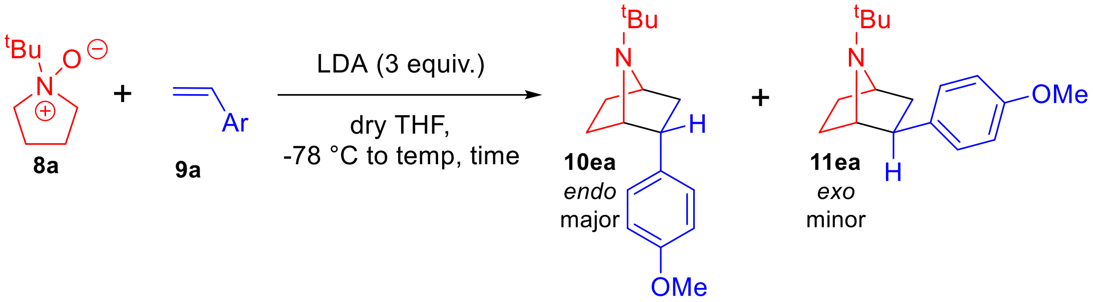

entry	equiv. of **8a**	time	end temp (°C)	conversion (%)[b]	**10ca** yield (%)[c]	d.r.[d]
1	1.0	16 h	0	33	32	ND
2	1.0	16 h	RT	58	57	ND
3	1.5	16 h	RT	67	65	18:2
4	1.5	0.5 h	RT	66	64	18:2
5	1.5	0.5 h	40	70	68	18:2
6	2.0	0.5 h	RT	97	(93)[e]	19:1
7	2.0	1 h	RT	100	(95)[e]	19:1
8*	2.0	1 h	RT	100	(95)[e]	19:1

[a]Reaction conditions: 0.2 mmol 1.0 equiv. (**9a**), 0.2-0.4 mmol, (**8a**), 3.0 equiv. LDA (in respect to **8a**), dry THF (4 mL, 0.1M), N_2, time, temp.
[b]Conversion: determined by crude ^{1}H NMR.
[c]NMR yield: determined by ^{1}H NMR with 1,3,5-trimethoxybenzene as NMR standard.
[d]d.r. determined by crude ^{1}H NMR.
[e]Isolated yield.
*4.0 equivalents of LDA (in respect to **8a**)
ND = not determined.

For our initial investigation we combined **8a** with **9a** in the presence of 3 equivalents of LDA (Table 1). Warming the reaction from -78 °C to 0 °C gave the desired product **10ea** in 32% NMR yield.[1] Warming to 23 °C (RT) saw an improvement to 57% yield (entry 2). When a super-stoichiometric amount of **8a** was used at RT, the yield slightly increased (entry 3) and we were able to shorten the reaction time (entry 4); warming past RT led to a minor improvement in yield (entry 5). When the equivalence of **8a** was further increased the reaction afforded an isolated yield of 93% for **10ea** in 30 minutes at room temperature (entry 6). The reaction was then run for 1-hour giving **10ea** in 95% isolated yield. (entry 7). Along with providing an excellent level of conversion of **9a** to 7-azanorbornane, we were very pleased to observe a high level of diastereoselectivity. Adding a 4th equivalent of LDA did not produce an appreciable difference (entry 8).

1.3 Scheme S2: Additional Transformations for 7-Azanorbornanes

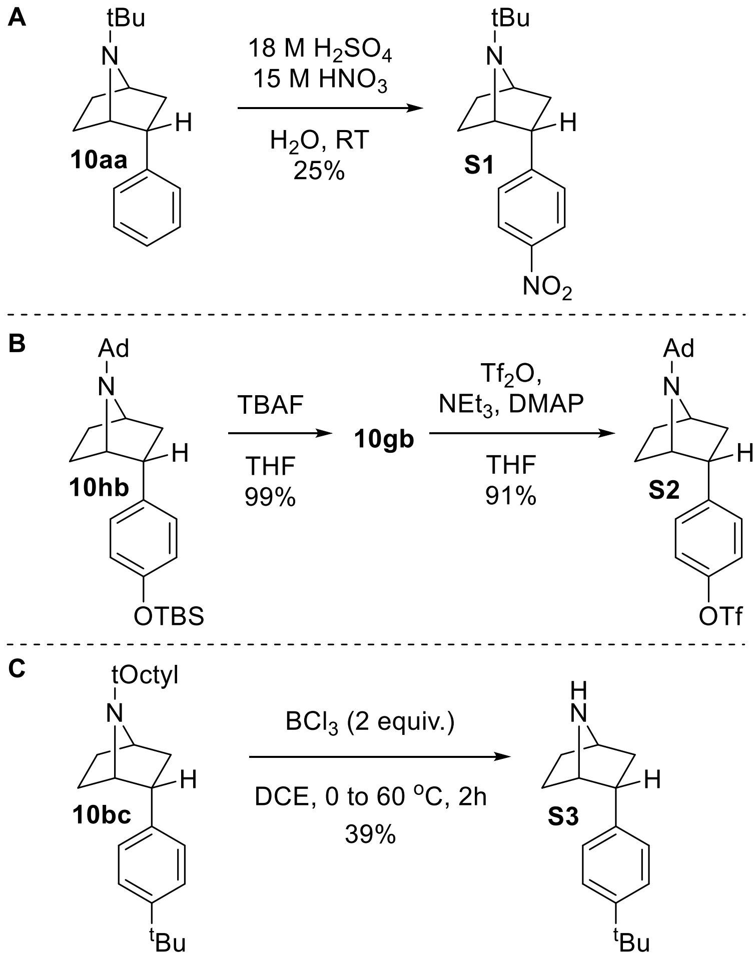

1.4 Experimental Section General Information

Nuclear magnetic resonance (NMR) spectra were measured on a Bruker AVANCE™ 400 MHz or AVANCE™ II 500 MHz spectrometer. ^{1}H NMR spectra were calibrated from standard TMS (δ 0.00) or solvent resonance ($CDCl_3$: δ 7.27, MeOD: δ 3.31). ^{13}C[^{1}H] NMR spectra were calibrated from solvent resonance ($CDCl_3$: δ 77.16, MeOD: δ 49.00). Structural assignments were made with additional information from gCOSY, gHSQC, and gNOESY experiments. High-resolution mass spectrometric analysis (HRMS) was measured on an Agilent Technologies 6530 Accurate-Mass QTOF LC/MS equipped with the Agilent Technologies 1200 series LC system. Infrared (IR) spectral analysis was performed on a Thermo Scientific Everest ATR. Reactions monitored by thin layer chromatography (TLC) used TLC silica gel 60 F_{254} and visualized under a 4-Watt 254/365 nm UV lamp. Flash column chromatography (FCC) (EtOAc/Hex or DCM/MeOH) was performed using a Biotage Isolera One Flash Chromatography instrument with a 10 g or 25 g Biotage® Sfär Silica D- Duo 60 μm column. An IKA heating mantel was used as the heat source for transformations that required heating.

Single crystal X-ray diffraction analysis was performed on a Bruker APEX II diffractometer based on a platform goniometer and equipped with an APEX II CCD Detector and Mo Kα radiation. Samples were mounted onto MiteGen MicroMounts using Paratone-N oil, and data was collected at 173 K unless otherwise noted.

1.4.1 Materials

All materials were used as purchased from MilliporeSigma, Thermo Fisher Scientific, TCI, Ambeed, or Oakwood Chemical, unless otherwise noted. Tetrahydrofuran (THF) was dried by a column of activated alumina and 4 Å molecular sieves via Inert PurSolv Solvent System. Pyrrolidine N-oxides were stored under rigorous anhydrous conditions in a desiccator with Drierite and phosphorus pentoxide. -78 ºC cooling baths were achieved using dry ice in acetone. Solutions of lithium diisopropyl amine (LDA) were titrated using salicylaldehyde phenylhydrazone before use.[2]

1.5 General synthetic procedures

1.5.1 General procedure for the preparation of *N*-substituted pyrrolidines.[3]

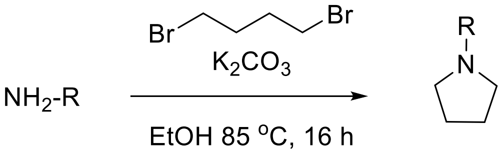

Amine (10 mmol, 1.0 equiv.) and K_2CO_3 (11 mmol, 1.1 equiv.) were dissolved in EtOH (10 mL, 1M). The solution was equipped with a stir bar and stirred at room temperature under a positive pressure of nitrogen. 1,4-dibromobutane (11 mmol, 1.1 equiv.) was added *via* syringe and the solution was heated to reflux for 16h. After incubation, the reaction was cooled to room temperature and the excess K_2CO_3 was filtered off. Given the volatility of free base pyrrolidines, the solution was acidified with 1M HCl, generating the hydrochloride ammonium salt, and then the ethanol was removed *in vacuo*. The aqueous solution was washed with ethyl ether (x2). The aqueous layer was then basified with 15% NaOH and extracted with ethyl ether (x2). This organic layer was dried over $MgSO_4$ and the ether was gently removed under reduced pressure. Unless otherwise noted, the pyrrolidines were taken onto the next step without further purification.

1.5.2 General procedure for the preparation of pyrrolidine *N*-oxides using *m*CPBA

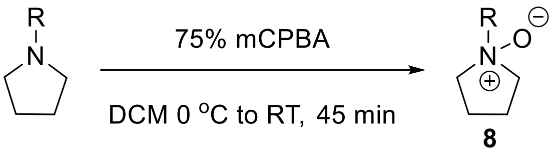

8

N-pyrrolidine (1.0 equiv.) was dissolved in DCM (1M) and was cooled to 0 ºC. 75% *m*CPBA (1.05 equiv.) was added portion-wise over the course of 10 minutes. The solution was warmed to RT and stirred for 45 minutes. The reaction was quenched by adding K_2CO_3 (6.0 equiv.) to the solution and stirred for 30 minutes. The solution was filtered and dried with $MgSO_4$. The solution was then concentrated *in vacuo*. Unless otherwise noted, no further purification was necessary.

1.5.3 General procedure for the preparation of pyrrolidine *N*-oxides using 30% H_2O_2

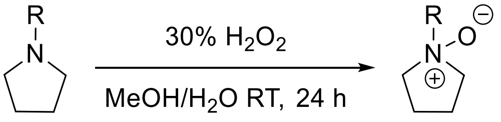

8

N-pyrrolidine (1.0 equiv.) was dissolved in MeOH (2M) and DI H_2O (4M). 30% H_2O_2 (4.0 equiv.) was added and the reaction stirred at room temperature for 24 hours. After this all solvents were removed *in*

vacuo with MgO being added into the solvent trap. The *N*-oxide was further dried by stirring $MgSO_4$ in DCM for 10 minutes. After 10 minutes, the solution was filtered and concentrated *in vacuo*. The *N*-oxides did not require further purification.

1-(*tert*-butyl)pyrrolidine

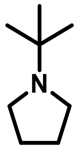

Pyrrolidine was prepared according to the general procedure using *tert*-butyl amine (3.58 mL, 30.0 mmol, 1.0 equiv.), 1,4-dibromobutane (3.47 mL, 33 mmol, 1.1 equiv.) K_2CO_3 (4.56 g, 33.0 mmol, 1.1 equiv.), and EtOH (30 mL, 1M). Aqueous workup was performed. Product was then purified by short path vacuum distillation at 35 °C to afford a colorless oil. (96% yield, 3.69 g, 29.0 mmol). Compound matched literature spectra.[3]

^{1}H NMR (400 MHz, $CDCl_3$) δ 2.68 – 2.59 (m, 4H), 1.81 – 1.71 (m, 4H), 1.09 (s, 9H).

^{13}C NMR (126 MHz, $CDCl_3$) δ 52.3, 46.0, 26.1, 24.0

1-(*tert*-butyl)pyrrolidine 1-oxide (8a).

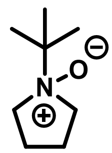

N-oxide was prepared according to the general procedure using 1-(*tert*-butyl)pyrrolidine (3.69 g, 29.0 mmol, 1.0 equiv.) 75% mCPBA (7.01 g, 30.45 mmol, 1.05 equiv.), and DCM (29 mL, 1M). The reaction was quenched using K_2CO_3 (24.05 g, 174 mmol, 6.0 equiv.). Following filtration of K_2CO_3 the solution was dried using $MgSO_4$. The solution was filtered and the solvent was removed *in vacuo.* No additional purification was necessary. Product was isolated as a white amorphous solid (97% yield, 4.03 g, 28.1 mmol). Product was kept dry and stored in a desiccator.

^{1}H NMR (400 MHz, $CDCl_3$) δ 3.46 – 3.37 (m, 2H), 3.27 (t, J = 9.2 Hz, 2H), 2.57 – 2.44 (m, 2H), 1.90 (m, J = 7.5 Hz, 2H), 1.48 (s, 9H).

^{13}C NMR (126 MHz, $CDCl_3$) δ 69.60, 60.86, 24.60, 21.43.

IR: 3020, 2969, 1228, 1217, 1068, 1047, 971 cm-1

HRMS (ESI) m/z: $[M+H]^+$ Calc'd for $C_8H_{18}NO$ 144.1383; Found 144.1377

1-(adamantan-1-yl)pyrrolidine

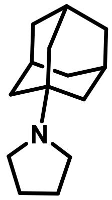

Pyrrolidine was prepared according to the general procedure using 1-adamantylamine (1.51 g, 10.0 mmol, 1.0 equiv.), 1,4-dibromobutane (1.31 mL, 11 mmol, 1.1 equiv.) K_2CO_3 (1.52 g, 11.0 mmol, 1.1 equiv.), and EtOH (10 mL, 1M). Aqueous workup was performed and no additional purification was necessary to afford a light yellow oil (95% yield, 1.95 g, 9.5 mmol). Compound matched literature spectra.[4]

1-(adamantan-1-yl)pyrrolidine 1-oxide (8b)

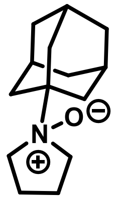

N-oxide was prepared according to the general procedure using 1-(adamantan-1-yl)pyrrolidine (1.95 g, 9.5 mmol, 1.0 equiv.) 75% mCPBA (2.30 g, 9.98 mmol, 1.05 equiv.), and DCM (9.5 mL, 1M). The reaction was quenched using K_2CO_3 (7.88 g, 57 mmol, 6.0 equiv.). Following filtration of K_2CO_3 the solution was dried using $MgSO_4$. The solution was filtered and the solvent was removed *in vacuo.* No additional purification was necessary. Product was isolated as a light yellow flakey solid (80% yield, 1.68 g, 7.6 mmol). Product was kept dry and stored in a desiccator.

^{1}H NMR (400 MHz, $CDCl_3$) δ 3.36 (dd, J = 16.2, 7.2 Hz, 2H), 3.19 (t, J = 8.8 Hz, 2H), 2.50 (dd, J = 12.3, 5.9 Hz, 2H), 2.23 (s, 3H), 2.15 (s, 6H), 1.87 (p, J = 6.6 Hz, 2H), 1.75 – 1.61 (m, 6H).

^{13}C NMR (126 MHz, $CDCl_3$) δ 70.4, 59.8, 36.7, 36.1, 29.9, 21.4.

IR: 2906, 2849, 1250, 1109, 1072, 1053, 989 cm-1

HRMS (ESI) m/z: $[M+H_3O]^+$ Calc'd for $C_{14}H_{26}NO_2$ 240.1958; Found 240.1972

1-(2,4,4-trimethylpentan-2-yl)pyrrolidine

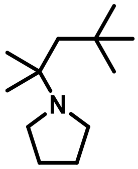

Pyrrolidine was prepared according to the general procedure using 2,4,4-trimethylpentan-2-amine (3.20 mL, 20.0 mmol, 1.0 equiv.), 1,4-dibromobutane (2.62 mL, 22 mmol, 1.1 equiv.) K_2CO_3 (3.04 g, 22.0 mmol, 1.1 equiv.), and EtOH (20 mL, 1M). Aqueous workup was followed by short path vacuum distillation at (40 °C internal vapor thermometer, 135 °C on IKA hotplate) to yield a colorless oil (80% yield, 2.93 g, 16.0 mmol).

^{1}H NMR (500 MHz, $CDCl_3$) δ 2.63 (t, J = 5.8 Hz, 4H), 1.71 (dd, J = 6.4, 3.0 Hz, 4H), 1.46 (s, 2H), 1.14 (s, 6H), 1.02 (s, 9H).

^{13}C NMR (126 MHz, $CDCl_3$) δ 56.0, 50.8, 45.3, 32.0, 31.8, 31.6, 25.4, 24.0.

IR: 2969, 1228, 1216 cm-1

HRMS (ESI) m/z: [M+Na]+ Calc'd for $C_{12}H_{25}NNa$ 206.1879; Found 206.1874

1-(2,4,4-trimethylpentan-2-yl)pyrrolidine 1-oxide (8c)

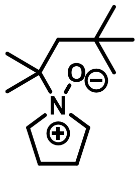

N-oxide was prepared according to the general procedure using 1-(2,4,4-trimethylpentan-2-yl)pyrrolidine (2.93 g, 16.0 mmol, 1.0 equiv.), 30% H_2O_2 (4.96 mL, 64.0 mmol, 4.0 equiv.), MeOH(8.0 mL, 2M), and DI H_2O (4.0 mL, 4M). After incubation, all solvents were removed *in vacuo* with MgO being added into the solvent trap. The *N*-oxide was further dried by stirring $MgSO_4$ in DCM for 10 minutes. After 10 minutes, the solution was filtered and concentrated *in vacuo*. No further purification was necessary. Product was isolated as a white amorphous solid (95% yield, 3.03 g, 15.2 mmol). Product was kept dry and stored in a desiccator.

^{1}H NMR (500 MHz, $CDCl_3$) δ 3.42 (m, 4H), 2.51 (m, 2H), 1.93 – 1.91 (m, 4H), 1.54 (s, 6H), 1.07 (s, 9H).

^{13}C NMR (126 MHz, $CDCl_3$) δ 76.3, 60.6, 47.6, 31.8, 31.6, 23.1, 21.9.

IR: 2955, 2870, 1242, 1126, 981, 922 cm-1

HRMS (ESI) m/z: [M+Na]+ Calc'd for $C_{12}H_{25}NNa$ 218.2115; Found 218.2111

1.5.4 General procedure for -OTBS protected styrenes[5]

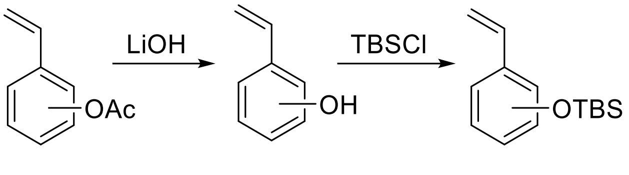

OAc substituted styrene (1.0 equiv.) was dissolved in 2:2:1 THF/MeOH/H_2O (1.85 M). LiOH (2.0 equiv.) was added and the reaction stirred for 2h at room temperature. The solution was acidified to pH 7 using 1M HCl and extracted with EtOAC (x2). The solution was dried with $MgSO_4$ and concentrated *in vacuo*. The OH-styrene was taken onto the next step without further purification.

The OH-styrene (1.0 equiv.) was dissolved in DCM (0.3M) along with NEt_3 (2.0 equiv.) and DMAP (0.1 equiv.). The solution was cooled to 0 °C and TBSCl (1.3 equiv.) was added. The reaction was warmed to RT and was monitored by TLC. After 4h, the reaction was quenched with DI H_2O and extracted with DCM (x2). The organic layer was dried with $MgSO_4$ and concentrated *in vacuo*. Products were purified by silica plug or column chromatography (100% hexanes, 100-200 mL).

***Tert*-butyldimethyl(4-vinylphenoxy)silane (4-OTBS styrene)**

4-OTBS styrene was prepared according to the general procedure for -OTBS protected styrenes. 4-vinylphenyl acetate (0.85 mL, 5.56 mmol, 1.0 equiv.) was dissolved in 2:2:1 THF/MeOH/H_2O (3 mL, 1.85 M). LiOH (0.266 g, 11.12 mmol, 2.0 equiv.) was added and the reaction stirred for 2h at room temperature. 4-vinylphenol was isolated as a white crystalline solid following the general procedure and was taken onto next step without further purification (99%, 5.53 mmol, 0.665 g). ^1H matched literature spectra.[5]

4-vinylphenol (0.665g, 5.53 mmol, 1.0 equiv.) was dissolved in DCM (18.4 mL, 0.3M) along with NEt_3 (1.47 mL, 11.06 mmol, 2.0 equiv.) and DMAP (64 mg, 0.53 mmol, 0.1 equiv.). The solution was cooled to 0 °C and TBSCl (1.09 g, 7.20 mmol, 1.3 equiv.) was added all at once. The reaction was warmed to RT and was monitored by TLC. After 4h, the reaction was quenched with DI H_2O and extracted with DCM (x2). The organic layer was dried with $MgSO_4$ and concentrated *in vacuo*. Products were purified by silica plug or column chromatography (100% hexanes, 100-200 mL) to afford *tert*-butyldimethyl(4-vinylphenoxy)silane as a colorless oil (99%, 5.47 mmol, 1.28 g). ^1H matched literature spectra.[5]

***Tert*-butyldimethyl(2-vinylphenoxy)silane (2-OTBS styrene)**

2-OTBS styrene was prepared according to the general procedure for -OTBS protected styrenes. 2-vinylphenyl acetate (0.497 g, 3.06 mmol, 1.0 equiv.) was dissolved in 2:2:1 THF/MeOH/H_2O (1.65 mL, 1.85 M). LiOH (0.1.47 g, 6.12 mmol, 2.0 equiv.) was added and the reaction stirred for 2h at room temperature. 2-vinylphenol was isolated as a white crystalline solid following the general procedure and was taken onto next step without further purification (100%, 3.06 mmol, 0.367 g).

2-vinylphenol (0.367g, 3.06 mmol, 1.0 equiv.) was dissolved in DCM (10.2 mL, 0.3M) along with NEt_3 (0.82 mL, 6.12 mmol, 2.0 equiv.) and DMAP (37 mg, 0.31 mmol, 0.1 equiv.). The solution was cooled to 0 °C and TBSCl (0.692 g, 4.59 mmol, 1.5 equiv.) was added all at once. The reaction was warmed to RT and was monitored by TLC. After 4h, the reaction was quenched with DI H_2O and extracted with DCM (x2). The organic layer was dried with $MgSO_4$ and concentrated *in vacuo*. Products were purified by silica plug or column chromatography (100% hexanes, 100-200 mL) to afford *tert*-butyldimethyl(2-vinylphenoxy)silane as a colorless oil (89%, 2.73 mmol, 0.640 g). ^1H matched literature spectra.[6]

1.5.5 Procedure for OTBDPS protected alkene[7]

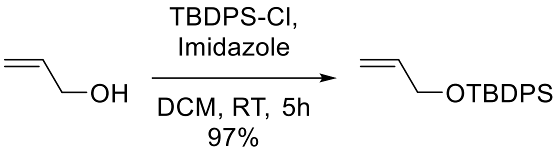

To a 50 mL RBF, prop-2-en-1-ol (0.10 mL, 1.47 mmol, 1.0 equiv.) and imidazole (0.108 g, 1.59 mmol, 1.08 equiv.) was dissolved in DCM (3.68 mL, 0.4M). Tert-butyldimethylsilyl chloride (0.382 mL, 1.47 mmol, 1.0 equiv.) was added dropwise and the reaction stirred at room temperature for 5 hours. After 5 hours, the reaction was quenched with DI water (5 mL), and the DCM layer was separated. The aqueous layer was washed 2 more times with DCM (5 mL) and the organic layers were combined, dried with $MgSO_4$ and concentrated *in vacuo*. No further purification was necessary as the (allyloxy)(tert-butyl)diphenylsilane was isolated as a clear oil (97%, 1.43 mmol, 0.423 g). ^{1}H NMR matched literature spectra.[7]

1.6 General procedure for the preparation of 7-azanorbornanes (10a-p)

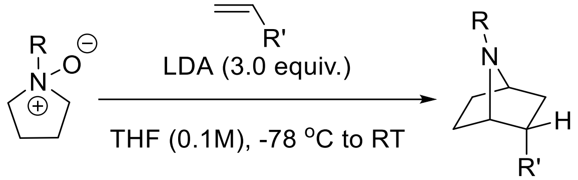

To an oven-dried borosilicate test tube, pyrrolidine *N*-oxide was added and charged with N_2. Dry THF (0.1M) was added and the solution was purged with N_2 and cooled to -78 °C in a dry ice/acetone bath. A solution of 1.8M LDA in THF (3.0 equiv.) was added dropwise *via* syringe and stirred for 5 minutes. Alkene (0.25-0.5 equiv.) was added *via* syringe dropwise at -78 °C. The solution was allowed to warm to room temperature and was monitored by TLC. After 1h, the reaction was quenched with DI H_2O and extracted with diethyl ether (x2). Unless otherwise noted, the ether layer was acidified using 1M HCl and was washed with ethyl ether (x2). The aqueous layer was basified with 15% NaOH, and was extracted with diethyl ether (x2). The organic layer was dried with Na_2SO_4 and concentrated *in vacuo*. The formation of the 7-azanorbornane was confirmed by ^{1}H NMR and was purified by column chromatography (Hexanes/EtOAc or EtOAc/MeOH).

7-(tert-butyl)-2-phenyl-7-azabicyclo[2.2.1]heptane (10aa)

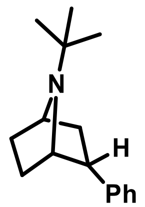

Azanorbornane was prepared according to the general procedure using 1-(*tert*-butyl)pyrrolidine 1-oxide **8a** (57 mg, 0.4 mmol, 1.0 equiv.), styrene (0.023 mL, 0.2 mmol, 0.5 equiv.), 1.8M LDA (0.67 mL, 1.2 mmol, 3.0 equiv.), dry THF (4.0 mL, 0.1M). Aqueous workup was performed followed by purification by FCC on silica gel (20% EtOAc in hexanes) to isolate product as a light-yellow oil (87% yield, 50 mg, 0.174 mmol) (Rf = 0.35 1:1 Hex/EtOAc).

^{1}H NMR (400 MHz, $CDCl_3$) δ 7.30 (t, *J* = 7.4 Hz, 2H), 7.19 (dd, *J* = 15.9, 7.6 Hz, 3H), 3.72 (t, *J* = 4.0 Hz, 1H), 3.68 (t, *J* = 4.6 Hz, 1H), 3.40 (q, *J* = 3.7 Hz, 1H), 2.13 – 2.02 (m, 1H), 1.73 – 1.60 (m, 1H), 1.46 (dd, *J* = 12.0, 5.7 Hz, 1H), 1.41 – 1.27 (m, 3H), 1.18 (s, 9H).

^{13}C NMR (101 MHz, $CDCl_3$) δ 142.5, 128.5, 128.2, 125.8, 61.0, 56.8, 51.8, 48.1, 36.2, 31.8, 30.3, 24.5.

IR: 3025, 2969, 2871, 1738, 1601, 1227, 1217, 1205, 1178, 1158, 1103, 1083, 1058, 1027, 985 cm-1

HRMS (ESI) m/z: [M+H3O]$^+$ Calc'd for $C_{13}H_{23}NNa$ 248.2009; Found 248.1996

Gram-scale synthesis of 7-(tert-butyl)-2-phenyl-7-azabicyclo[2.2.1]heptane (10aa)

To an oven-dried RBF, 1-(*tert*-butyl)pyrrolidine 1-oxide **8a** (1.003 g, 7.0 mmol, 1.0 equiv.) was added and charged with N_2. To the flask, dry THF (70.0 mL, 0.1M) was added, purged with N_2, and cooled to -78 °C in a dry ice/acetone bath. 1.8M LDA in THF (11.67 mL, 21 mmol, 3.0 equiv.) was added dropwise *via* syringe and stirred for 5 minutes. Styrene (0.40 mL, 3.5 mmol, 0.5 equiv.) was added *via* syringe dropwise at -78 °C. The solution was allowed to warm to room temperature and was monitored by TLC. After 1h, the reaction was quenched with DI H_2O and extracted with diethyl ether (x2). The ether layer was acidified using 1M HCl and the aqueous layer was washed with ethyl ether (x2). The aqueous layer was basified with 15% NaOH, and was extracted with diethyl ether (x2). The organic layer was dried with $MgSO_4$ and concentrated *in vacuo*. The formation of the azanorbornane was confirmed by ^{1}H NMR and was purified by flash column chromatography (20% EtOAc in hexanes) to isolate product as a light-yellow oil (90%, 1.45g, 6.3 mmol). (Rf = 0.35 1:1 Hex/EtOAc).

7-(adamantan-1-yl)-2-phenyl-7-azabicyclo[2.2.1]heptane (10ab)

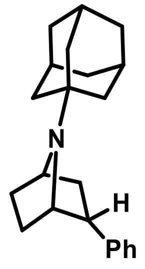

Azanorbornane was prepared according to the general procedure using 1-(adamant-1-yl)pyrrolidine 1-oxide (88 mg, 0.4 mmol, 1.0 equiv.), styrene (0.023 mL, 0.2 mmol, 0.5 equiv.), 1.8M LDA (0.67 mL, 1.2 mmol, 3.0 equiv.), dry THF (4.0 mL, 0.1M). Aqueous work up was performed followed by purification by FCC on silica gel (40 % EtOAc in Hexanes) to isolate product as a white amorphous solid (92% yield, 56 mg, 0.184 mmol) (Rf = 0.28 1:1 Hex/EtOAc).

^{1}H NMR (400 MHz, $CDCl_3$) δ 7.33 (dd, *J* = 10.1, 4.9 Hz, 2H), 7.27 – 7.17 (m, 3H), 3.82 (t, *J* = 4.0 Hz, 1H), 3.78 (t, *J* = 4.3 Hz, 1H), 3.45 – 3.36 (m, 1H), 2.09 (s, 4H), 1.82 (s, 6H), 1.67 (d, *J* = 11.1 Hz, 7H), 1.49 (dd, *J* = 12.0, 5.6 Hz, 1H), 1.44 – 1.31 (m, *J* = 7.4 Hz, 3H).

^{13}C NMR (126 MHz, $CDCl_3$) δ 142.7, 128.5, 128.2, 125.7, 59.6, 55.5, 52.1, 48.4, 43.3, 36.9, 36.3, 32.2, 30.0, 25.0.

IR: 3024, 2900, 2848, 1937, 1600, 1266, 1186, 1168, 1135, 1099, 1075, 1056, 1032, 1012, 976 cm-1

HRMS (ESI) m/z: [M+Na]$^+$ Calc'd for $C_{22}H_{29}NNa$ 330.2192; Found 330.2191

2-phenyl-7-(2,4,4-trimethylpentan-2-yl)-7-azabicyclo[2.2.1]heptane (10ac)

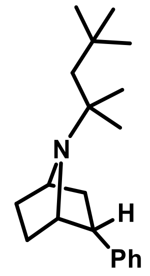

Azanorbornane was prepared according to the general procedure using 1-(2,4,4-trimethylpentan-2-yl)pyrrolidine 1-oxide (94 mg, 0.47 mmol, 1.0 equiv.), styrene (0.014 mL, 0.12 mmol, 0.25 equiv.), 1.6M LDA (0.84 mL, 1.4 mmol, 3.0 equiv.), dry THF (4.7 mL, 0.1M). Aqueous work up was performed followed by purification by FCC on silica gel (10 % EtOAc in Hexanes) to isolate product as a yellow oil (95% yield, 32 mg, 0.112 mmol). (Rf = 0.67 1:1 Hex/EtOAc).

^{1}H NMR (400 MHz, $CDCl_3$) δ 7.29 (t, *J* = 7.6 Hz, 2H), 7.18 (dd, *J* = 17.6, 7.5 Hz, 3H), 3.70 (t, *J* = 4.1 Hz, 1H), 3.65 (t, *J* = 4.7 Hz, 1H), 3.43 – 3.32 (m, 1H), 2.06 (t, *J* = 11.6 Hz, 1H), 1.70 – 1.60 (m, 1H), 1.47 – 1.41 (m, 3H), 1.38 – 1.25 (m, 3H), 1.17 (s, 6H), 1.04 (s, 9H).

^{13}C NMR (126 MHz, $CDCl_3$) δ 142.8, 128.5, 128.2, 125.7, 60.7, 56.4, 55.6, 55.1, 48.1, 36.2, 32.1, 32.0, 31.9, 29.6, 29.3, 24.9.

IR: 2951, 2901, 2873, 1737, 1708, 1228, 1180, 1162, 1142, 1109, 1090, 1072, 1048, 1031, 940 cm-1

HRMS (ESI) m/z: $[M+Na]^+$ Calc'd for $C_{20}H_{31}NNa$ 308.2349; Found 308.2343

7-(*tert*-butyl)-2-(4-(*tert*-butyl)phenyl)-7-azabicyclo[2.2.1]heptane (10ba)

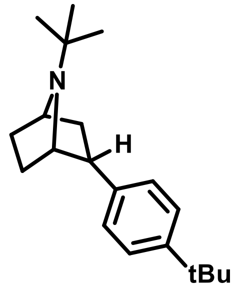

Azanorbornane was prepared according to the general procedure using 1-(*tert*-butyl)pyrrolidine 1-oxide (57 mg, 0.4 mmol, 1.0 equiv.), 4-*tert*-butylstyrene (0.037 mL, 0.2 mmol, 0.5 equiv.), 1.8M LDA (0.67 mL, 1.2 mmol, 3.0 equiv.), dry THF (4.0 mL, 0.1M). Aqueous work up was performed followed by purification by FCC on silica gel (15 % EtOAc in Hexanes) to isolate product as an off-white solid (95% yield, 54 mg, 0.190 mmol). (Rf = 0.54 1:1 Hex/EtOAc)

^{1}H NMR (500 MHz, $CDCl_3$) δ 7.35 (d, *J* = 8.2 Hz, 2H), 7.17 (d, *J* = 8.1 Hz, 2H), 3.73 (t, *J* = 3.8 Hz, 1H), 3.70 (t, *J* = 4.4 Hz, 1H), 3.43 – 3.35 (m, 1H), 2.10 (t, *J* = 11.5 Hz, 1H), 1.71 (d, *J* = 3.9 Hz, 1H), 1.44 (m, 4H), 1.35 (s, 9H), 1.21 (s, 9H).

^{13}C NMR (125 MHz, $CDCl_3$) δ 148.4, 139.4, 128.0, 125.0, 60.9, 56.7, 51.7, 47.6, 36.3, 34.3, 31.7, 31.4, 30.2, 24.5.

IR: 2961, 2902, 2868, 1897, 1601, 1267, 1228, 1179, 1202, 1179, 1123, 1111, 1085, 985 cm-1

HRMS (ESI) m/z: $[M+H_3O]^+$ Calc'd for $C_{20}H_{34}NO$ 304.2635; Found 304.2634

7-(adamantan-1-yl)-2-(4-(*tert*-butyl)phenyl)-7-azabicyclo[2.2.1]heptane (10bb)

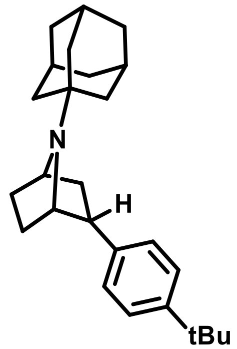

Azanorbornane was prepared according to the general procedure using 1-(adamant-1-yl)pyrrolidine 1-oxide (88 mg, 0.4 mmol, 1.0 equiv.), 4-*tert*-butylstyrene (0.037 mL, 0.2 mmol, 0.5 equiv.), 1.8M LDA (0.67 mL, 1.2 mmol, 3.0 equiv.), dry THF (4.0 mL, 0.1M). Aqueous work up was performed followed by purification by FCC on silica gel (15 % EtOAc in Hexanes) to isolate product as a white amorphous solid (95% yield, 64 mg, 0.190 mmol). (Rf = 0.55 1:1 Hex/EtOAc).

^{1}H NMR (499 MHz, $CDCl_3$) δ 7.31 (d, J = 8.0 Hz, 2H), 7.13 (d, J = 8.1 Hz, 2H), 3.76 (t, J = 4.0 Hz, 1H), 3.73 (t, J = 4.3 Hz, 1H), 3.37 – 3.27 (m, 1H), 2.05 (s, 4H), 1.78 (s, 6H), 1.64 (s, 8H), 1.46 – 1.39 (m, 2H), 1.33 (m, 1H), 1.31 (s, 9H).

^{13}C NMR (126 MHz, $CDCl_3$) δ 148.5, 139.6, 128.2, 125.1, 59.7, 55.5, 52.1, 48.0, 43.3, 36.9, 36.6, 34.5, 32.3, 31.6, 30.0, 25.1.

IR: 2956, 2903, 2846, 1268, 1186, 1171, 1137, 1107, 1097, 1073, 1008, 986, 968 cm-1

HRMS (ESI) m/z: $[M+Na]^+$ Calc'd for $C_{26}H_{37}NNa$ 386.2818; Found 386.2805

2-phenyl-7-(2,4,4-trimethylpentan-2-yl)-7-azabicyclo[2.2.1]heptane (10bc)

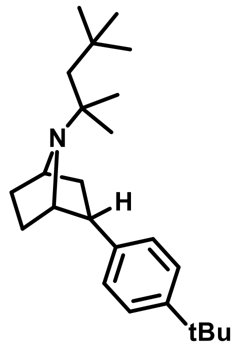

Azanorbornane was prepared according to the general procedure using 1-(2,4,4-trimethylpentan-2-yl)pyrrolidine 1-oxide (94 mg, 0.47 mmol, 1.0 equiv.), 4-*tert*-butylstyrene (0.022 mL, 0.113 mmol, 0.25 equiv.), 1.6M LDA (0.88 mL, 1.41 mmol, 3.0 equiv.), dry THF (4.7 mL, 0.1M). Aqueous work up was performed followed by purification by FCC on silica gel (10 % EtOAc in Hexanes) to isolate product as a off-white amorphous solid (96% yield, 39 mg, 0.113 mmol). (Rf = 0.73 1:1 Hex/EtOAc).

^{1}H NMR (400 MHz, $CDCl_3$) δ 7.31 (d, J = 8.2 Hz, 2H), 7.13 (d, J = 8.1 Hz, 2H), 3.67 (t, J = 4.2 Hz, 1H), 3.63 (t, J = 4.4 Hz, 1H), 3.38 – 3.29 (m, 1H), 2.10 – 1.98 (m, 1H), 1.71 – 1.56 (m, 2H), 1.47 – 1.38 (m, 4H), 1.31 (s, 10H), 1.17 (s, 6H), 1.04 (s, 9H).

^{13}C NMR (126 MHz, $CDCl_3$) δ 148.5, 139.7, 128.2, 125.1, 60.6, 56.4, 55.6, 55.1, 47.7, 36.4, 34.5, 32.1, 32.0, 31.9, 31.6, 29.6, 29.3, 25.0.

IR: 2952, 2901, 2870, 1266, 1228, 1204, 1182, 1143, 1111, 1068, 1050, 1017, 985 cm-1

HRMS (ESI) m/z: $[M+H_3O]^+$ Calc'd for $C_{24}H_{42}NO$ 360.3261; Found 360.3244

7-(tert-butyl)-2-(*p*-tolyl)-7-azabicyclo[2.2.1]heptane (10ca)

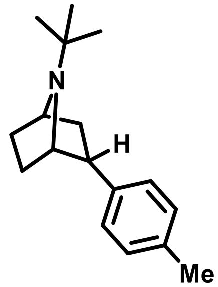

Azanorbornane was prepared according to the general procedure using 1-(*tert*-butyl)pyrrolidine 1-oxide (57 mg, 0.4 mmol, 1.0 equiv.), 1-methyl-4-vinylbenzene (0.026 mL, 0.2 mmol, 0.5 equiv.), 1.8M LDA (0.67 mL, 1.2 mmol, 3.0 equiv.), dry THF (4.0 mL, 0.1M). Aqueous work up was performed followed by purification by FCC on silica gel (40% MeOH in DCM) to isolate product as a yellow oil (83% yield, 40 mg, 0.166 mmol). (Rf = 0.25 9:1 DCM/MeOH).

^{1}H NMR (400 MHz, $CDCl_3$) δ 7.10 (s, 4H), 3.67 (dd, *J* = 10.0, 4.5 Hz, 2H), 3.41 – 3.31 (m, 1H), 2.32 (s, 3H), 2.07 (t, *J* = 11.5 Hz, 1H), 1.72 – 1.64 (m, *J* = 12.6, 8.1, 4.3 Hz, 1H), 1.46 – 1.29 (m, 4H), 1.18 (s, 9H).

^{13}C NMR (125 MHz, $CDCl_3$) δ 139.4, 135.2, 128.9, 128.4, 61.0, 56.9, 47.7, 36.3, 31.8, 30.2, 24.5, 21.0.

IR: 2966, 2870, 1606, 1298, 1267, 1227, 1267, 1179, 1120, 1101, 1084, 1055, 1028, 1010, 986, 943 cm-1

HRMS (ESI) m/z: $[M+Na]^+$ Calc'd for $C_{17}H_{25}NNa$ 266.1879; Found 266.1893

7-(adamantan-1-yl)-2-(*p*-tolyl)-7-azabicyclo[2.2.1]heptane (10cb)

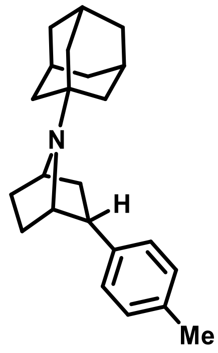

Azanorbornane was prepared according to the general procedure using 1-(adamant-1-yl)pyrrolidine 1-oxide (88 mg, 0.4 mmol, 1.0 equiv.), 1-methyl-4-vinylbenzene (0.026 mL, 0.2 mmol, 0.5 equiv.), 1.8M LDA (0.67 mL, 1.2 mmol, 3.0 equiv.), dry THF (4.0 mL, 0.1M). Aqueous work up was performed followed by purification by FCC on silica gel (30% MeOH in DCM) to isolate product as a light yellow amorphous solid (83% yield, 53 mg, 0.166 mmol). (Rf = 0.57 9:1 DCM/MeOH).

^{1}H NMR (400 MHz, $CDCl_3$) δ 7.10 (s, 4H), 3.82 – 3.68 (m, 2H), 3.40 – 3.28 (m, 1H), 2.32 (s, 3H), 2.05 (s, 4H), 1.78 (s, 6H), 1.64 (s, 7H), 1.47 – 1.28 (m, 4H).

^{13}C NMR (126 MHz, $CDCl_3$) δ 135.2, 128.9, 128.4, 114.0, 59.7, 55.6, 52.2, 48.0, 43.2, 36.9, 36.4, 32.3, 30.0, 25.0, 21.1.

IR: 2912, 2854, 1712, 1204, 1062, 975, 942 cm-1

HRMS (ESI) m/z: $[M+H_3O]^+$ Calc'd for $C_{23}H_{34}NO$ 340.2640; Found 340.2665

2-(*p*-tolyl)-7-(2,4,4-trimethylpentan-2-yl)-7-azabicyclo[2.2.1]heptane (10cc)

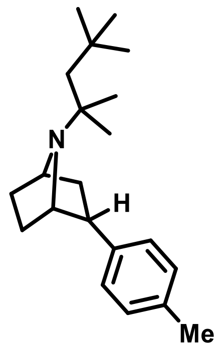

Azanorbornane was prepared according to the general procedure using 1-(2,4,4-trimethylpentan-2-yl)pyrrolidine 1-oxide (87 mg, 0.473 mmol, 1.0 equiv.), 1-methyl-4-vinylbenzene (0.015 mL, 0.118 mmol, 0.25 equiv.), 1.69M LDA (0.85 mL, 1.42 mmol, 3.0 equiv.), dry THF (4.7 mL, 0.1M). Aqueous work up was performed followed by purification by FCC on silica gel (10% EtOAc in hexanes) to isolate product as a white amorphous solid (69% yield, 24 mg, 0.081 mmol). (Rf = 0.69 1:1 Hex/EtOAc).

^{1}H NMR (400 MHz, $CDCl_3$) δ 7.13 (s, 4H), 3.69 (t, *J* = 4.5 Hz, 1H), 3.67 (t, *J* = 4.7 Hz, 1H), 3.41 – 3.31 (m, 1H), 2.35 (s, 3H), 2.13 – 2.02 (m, 1H), 1.72 – 1.63 (m, 1H), 1.48 (s, 2H), 1.46 – 1.31 (m, 4H), 1.20 (d, *J* = 1.7 Hz, 6H), 1.07 (s, 9H).

^{13}C NMR (126 MHz, $CDCl_3$) δ 139.7, 135.1, 128.9, 128.4, 60.6, 56.4, 55.6, 55.0, 47.7, 36.3, 32.2, 31.9, 29.6, 29.3, 24.9, 21.1.

IR: 2950, 2900, 2871, 1266, 1228, 1181, 1164, 1142, 1100, 1067, 1050, 1020, 983 cm-1

HRMS (ESI) m/z: $[MH+CH_3CN]^+$ Calc'd for $C_{23}H_{37}N_2$ 341.2951; Found 341.2978

2-([1,1'-biphenyl]-4-yl)-7-(tert-butyl)-7-azabicyclo[2.2.1]heptane (10da)

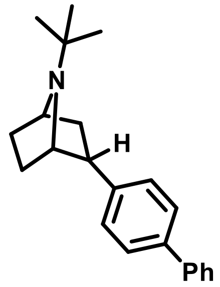

Azanorbornane was prepared according to the general procedure using 1-(*tert*-butyl)pyrrolidine 1-oxide (57 mg, 0.4 mmol, 1.0 equiv.), 4-vinyl-1,1'-biphenyl (36 mg, 0.2 mmol, 0.5 equiv.), 1.8M LDA (0.67 mL, 1.2 mmol, 3.0 equiv.), dry THF (4.0 mL, 0.1M). Aqueous work up was performed followed by purification by FCC on silica gel (20 % EtOAc in Hexanes) to isolate product as an amorphous white solid (84% yield, 57 mg, 0.188 mmol). (Rf = 0.48 1:1 Hex/EtOAc).

^{1}H NMR (400 MHz, $CDCl_3$) δ 7.60 (dd, *J* = 8.2, 1.1 Hz, 2H), 7.54 (d, *J* = 8.3 Hz, 2H), 7.44 (t, *J* = 7.6 Hz, 2H), 7.37 – 7.27 (m, 3H), 3.76 (t, *J* = 4.0 Hz, 1H), 3.70 (t, *J* = 4.7 Hz, 1H), 3.47 – 3.39 (m, 1H), 2.16 – 2.06 (m, 1H), 1.76 – 1.65 (m, 1H), 1.50 (dd, *J* = 12.0, 5.8 Hz, 1H), 1.46 – 1.31 (m, 3H), 1.20 (s, 9H).

^{13}C NMR (126 MHz, $CDCl_3$) δ 141.8, 141.2, 138.7, 128.9, 128.9, 127.2, 127.1, 127.0, 61.0, 56.8, 51.8, 47.9, 36.4, 31.9, 30.3, 24.6.

IR: 2976, 2954, 2847, 1909, 1596, 1260, 1242, 1187, 1135, 1099, 1039, 1005, 987, 937 cm-1

HRMS (ESI) m/z: $[M+H_3O]^+$ Calc'd for $C_{22}H_{30}NO$ 324.2327 Found 324.2340

2-([1,1'-biphenyl]-4-yl)-7-(adamantan-1-yl)-7-azabicyclo[2.2.1]heptane (10db)

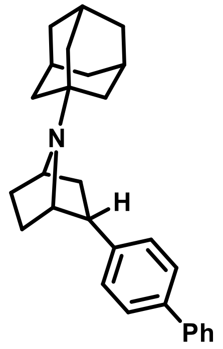

Azanorbornane was prepared according to the general procedure using 1-(adamant-1-yl)pyrrolidine 1-oxide (88 mg, 0.4 mmol, 1.0 equiv.), 4-vinyl-1,1'-biphenyl (36 mg, 0.2 mmol, 0.5 equiv.), 1.8M LDA (0.67 mL, 1.2 mmol, 3.0 equiv.), dry THF (4.0 mL, 0.1M). Aqueous work up was performed followed by purification by FCC on silica gel (15 % EtOAc in Hexanes) to isolate product as a white amorphous solid (82% yield, 63 mg, 0.164 mmol). (Rf = 0.63 1:1 Hex/EtOAc).

^{1}H NMR (400 MHz, $CDCl_3$) δ 7.59 (d, *J* = 7.6 Hz, 2H), 7.53 (d, *J* = 8.2 Hz, 2H), 7.43 (t, *J* = 7.6 Hz, 2H), 7.32 (t, *J* = 7.4 Hz, 1H), 7.28 (d, *J* = 8.0 Hz, 2H), 3.82 (t, *J* = 3.9 Hz, 1H), 3.76 (t, *J* = 4.6 Hz, 1H), 3.44 – 3.36 (m, 1H), 2.06 (s, 4H), 1.79 (d, *J* = 2.4 Hz, 6H), 1.63 (m, 7H), 1.53 – 1.30 (m, 4H).

^{13}C NMR (125 MHz, $CDCl_3$) δ 141.9, 141.2, 138.6, 128.9, 128.8, 127.1, 127.1, 126.9, 59.7, 55.5, 52.1, 48.2, 43.3, 36.9, 36.5, 32.3, 30.0, 25.1.

IR: 2966, 2867, 1891, 1799, 1637, 1255, 1178, 1160, 1097, 1085, 1039, 1005, 984, 943 cm-1

HRMS (ESI) m/z: $[M+H_3O]^+$ Calc'd for $C_{28}H_{36}NO$ 402.2791; Found 402.2773

2-([1,1'-biphenyl]-4-yl)-7-(2,4,4-trimethylpentan-2-yl)-7-azabicyclo[2.2.1]heptane (10dc)

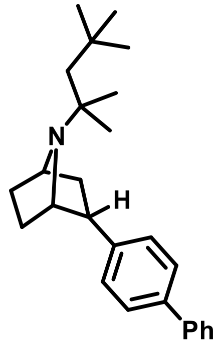

Azanorbornane was prepared according to the general procedure using 1-(2,4,4-trimethylpentan-2-yl)pyrrolidine 1-oxide (145 mg, 0.725 mmol, 1.0 equiv.), 4-vinyl-1,1'-biphenyl (32 mg, 0.181 mmol, 0.25 equiv.), 1.63M LDA (1.33 mL, 2.18 mmol, 3.0 equiv.), dry THF (6 mL, 0.14M). Aqueous work up was performed followed by purification by FCC on silica gel (5 % EtOAc in Hexanes) to isolate product as an off white powder (86% yield, 56 mg, 0.156 mmol). (Rf = 0.83 1:1 Hex/EtOAc).

^{1}H NMR (400 MHz, $CDCl_3$) δ 7.63 – 7.57 (m, 2H), 7.53 (d, *J* = 8.2 Hz, 2H), 7.43 (t, *J* = 7.6 Hz, 2H), 7.33 (d, *J* = 7.2 Hz, 1H), 7.30 – 7.25 (m, 2H), 3.73 (t, *J* = 4.1 Hz, 1H), 3.67 (t, *J* = 4.7 Hz, 1H), 3.46 – 3.37 (m, 1H), 2.15 – 2.04 (m, 1H), 1.74 – 1.64 (m, 1H), 1.50 – 1.45 (m, 3H), 1.44 – 1.27 (m, 3H), 1.19 (s, 6H), 1.05 (s, 9H).

^{13}C NMR (126 MHz, $CDCl_3$) δ 142.0, 141.2, 138.6, 128.9, 128.8, 127.1, 126.9, 60.7, 56.4, 55.7, 55.0, 47.9, 36.4, 32.1, 32.0, 31.9, 29.6, 29.3, 25.0.

IR: 2949, 2902, 1597, 1268, 1229, 1180, 1142, 1099, 1066, 1054, 1005, 939 cm-1

HRMS (ESI) m/z: $[M+Na]^+$ Calc'd for $C_{26}H_{35}NNa$ 384.2662; Found 384.2646

7-(tert-butyl)-2-(4-methoxyphenyl)-7-azabicyclo[2.2.1]heptane (10ea)

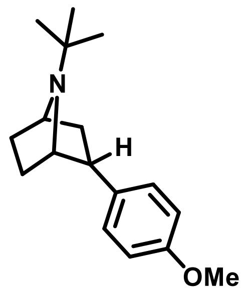

Azanorbornane was prepared according to the general procedure using 1-(*tert*-butyl)pyrrolidine 1-oxide (57 mg, 0.4 mmol, 1.0 equiv.), 4-methoxystyrene (0.027 mL, 0.2 mmol, 0.5 equiv.), 1.8M LDA (0.67 mL, 1.2 mmol, 3.0 equiv.), dry THF (4.0 mL, 0.1M). Aqueous work up was performed followed by purification by FCC on silica gel (40 % EtOAc in Hexanes) to isolate product as a light yellow oil (95% yield, 49 mg, 0.190 mmol). (Rf = 0.36 1:1 Hex/EtOAc)

^{1}H NMR (400 MHz, $CDCl_3$) δ 7.12 (d, *J* = 8.3 Hz, 2H), 6.84 (d, *J* = 8.3 Hz, 2H), 3.79 (s, 3H), 3.66 (t, *J* = 3.8 Hz, 2H), 3.38 – 3.28 (m, 1H), 2.06 (t, *J* = 11.9 Hz, 1H), 1.72 – 1.62 (m, *J* = 9.8, 5.6 Hz, 1H), 1.42 – 1.25 (m, 4H), 1.17 (s, 9H).

^{13}C NMR (125 MHz, $CDCl_3$) δ 157.7, 134.6, 129.3, 113.6, 61.06, 56.82, 55.4, 51.80, 47.30, 36.5, 31.8, 30.3, 24.4.

IR: 2965, 2907, 2872, 2833, 2073, 1877, 1611, 1268, 1249, 1178, 1113, 1085, 1040, 986 cm-1

HRMS (ESI) m/z: $[M+H_3O]^+$ Calc'd for $C_{17}H_{28}NO_2$ 278.2115; Found 278.2102

7-(adamantan-1-yl)-2-(4-methoxyphenyl)-7-azabicyclo[2.2.1]heptane (10eb)

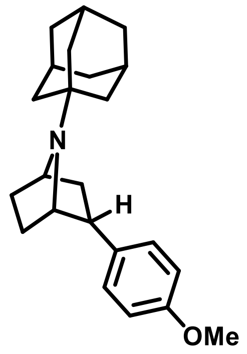

Azanorbornane was prepared according to the general procedure using 1-(adamant-1-yl)pyrrolidine 1-oxide (88 mg, 0.4 mmol, 1.0 equiv.), 4-methoxystyrene (0.027 mL, 0.2 mmol, 0.5 equiv.), 1.8M LDA (0.67 mL, 1.2 mmol, 3.0 equiv.), dry THF (4.0 mL, 0.1M). Aqueous work up was performed followed by purification by FCC on silica gel (20 % EtOAc in Hexanes) to isolate product as a off-white flakey solid (95% yield, 69 mg, 0.190 mmol). (Rf = 0.68 1:1 Hex/EtOAc).

^{1}H NMR (400 MHz, $CDCl_3$) δ 7.12 (d, *J* = 8.6 Hz, 2H), 6.84 (d, *J* = 8.5 Hz, 2H), 3.79 (s, 3H), 3.73 (s, 2H), 3.34 – 3.25 (m, *J* = 6.4 Hz, 1H), 2.05 (s, 3H), 1.77 (s, 6H), 1.63 (t, *J* = 1.6 Hz, 8H), 1.42 – 1.26 (m, 4H).

^{13}C NMR (126 MHz, $CDCl_3$) δ 157.7, 134.7, 129.3, 113.6, 59.7, 55.5, 55.4, 52.1, 47.5, 43.3, 36.9, 36.6, 32.3, 30.0, 24.9.

IR: 2987, 2957, 2898, 2847, 1882, 1611, 1252, 1243, 1210, 1178, 1101, 1071, 1032, 1012, 987 cm-1

HRMS (ESI) m/z: $[M+Na]^+$ Calc'd for $C_{23}H_{31}NONa$ 360.2298; Found 360.2276

2-(4-methoxyphenyl)-7-(2,4,4-trimethylpentan-2-yl)-7-azabicyclo[2.2.1]heptane (10ec)

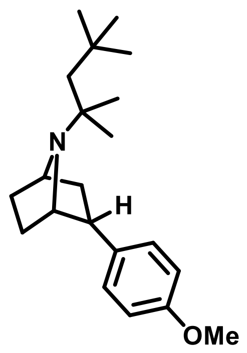

Azanorbornane was prepared according to the general procedure using 1-(2,4,4-trimethylpentan-2-yl)pyrrolidine 1-oxide (119 mg, 0.597 mmol, 1.0 equiv.), 4-methoxystyrene (0.020 mL, 0.149 mmol, 0.25 equiv.), 1.6M LDA (1.12 mL, 1.79 mmol, 3.0 equiv.), dry THF (6.0 mL, 0.1M). Aqueous work up was performed followed by purification by FCC on silica gel (20 % EtOAc in Hexanes) to isolate product as a yellow oil (97% yield, 45 mg, 0.144 mmol). (Rf = 0.55 1:1 Hex/EtOAc).

^{1}H NMR (400 MHz, $CDCl_3$) δ 7.11 (d, *J* = 8.4 Hz, 2H), 6.84 (d, *J* = 8.3 Hz, 2H), 3.79 (s, 3H), 3.67 – 3.59 (m, 2H), 3.35 – 3.25 (m, 1H), 2.04 (tt, *J* = 11.8, 4.0 Hz, 1H), 1.64 (m, 1H), 1.44 (s, 2H), 1.36 (dd, *J* = 12.5, 5.2 Hz, 2H), 1.33 – 1.23 (m, 2H), 1.17 (s, 3H), 1.16 (s, 3H), 1.04 (s, 9H).

^{13}C NMR (126 MHz, $CDCl_3$) δ 157.7, 134.9, 129.4, 113.6, 60.7, 56.4, 55.6, 55.4, 55.0, 47.3, 36.5, 32.1, 32.0, 31.9, 29.6, 29.3, 24.8.

IR: 2954, 2904, 2889, 1611, 1269, 1251, 1227, 1176, 1142, 1110, 1102, 1061, 1033, 1015, 986, 976, 942 cm-1

HRMS (ESI) m/z: $[M+H_3O]^+$ Calc'd for $C_{21}H_{36}NO_2$ 334.2741; Found 334.2739

4-(-7-(*tert*-butyl)-7-azabicyclo[2.2.1]heptan-2-yl)phenol (10fa)

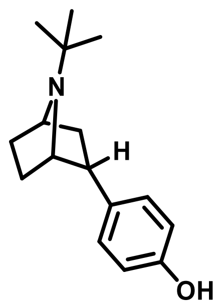

Azanorbornane was prepared according to the general procedure using 1-(*tert*-butyl)pyrrolidine 1-oxide (57 mg, 0.4 mmol, 1.0 equiv.), 4-vinylphenyl acetate (0.031 mL, 0.2 mmol, 0.5 equiv.), 1.8M LDA (0.67 mL, 1.2 mmol, 3.0 equiv.), dry THF (4.0 mL, 0.1M). Aqueous work up was performed followed by purification by FCC on silica gel (30% MeOH in DCM) to isolate product as an off-white amorphous solid (66% yield, 32 mg, 0.132 mmol). The minor diastereomer could not be completely separated but was < 5% (Major diastereomer Rf = 0.11 9:1 DCM/MeOH)

^{1}H NMR (400 MHz, $CDCl_3$) δ 7.13 minor (d, *J* = 9.0 Hz,), 7.06 (d, *J* = 8.3 Hz, 2H), 6.81 minor (d, *J* = 7.7 Hz), 6.77 (d, *J* = 8.2 Hz, 2H), 3.68 (t, *J* = 4.2 Hz, 2H), 3.34 (s, 1H), 2.08 (t, *J* = 12.6 Hz, 1H), 1.75 – 1.64 (m, 2H), 1.38 (dd, *J* = 11.1, 4.8 Hz, 3H), 1.35 – 1.24 (m, 2H), 1.18 (s, 9H).

^{13}C NMR (126 MHz, $CDCl_3$) δ 154.3, 133.6, 129.4, 127.9 minor, 115.4, 114.0 minor, 61.5, 57.3, 52.6, 46.8, 36.2, 31.7, 30.1, 24.3.

IR: 3054, 2975, 1881, 1610, 1276, 1222, 1190, 1171, 1163, 1110, 1069, 1036, 1011, 993, 984, 949,cm-1

HRMS (ESI) m/z (z=2): $[M+2H]^{+2}$ Calc'd for $C_{16}H_{25}NO$ 123.5963; Found 123.5973

4-(-7-adamantan-1-yl)-7-azabicyclo[2.2.1]heptan-2-yl)phenol (10fb)

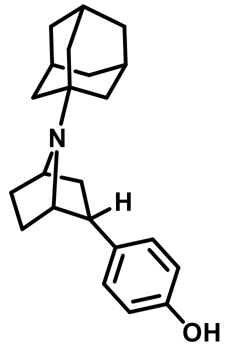

Azanorbornane was prepared according to the general procedure using 1-(adamant-1-yl)pyrrolidine 1-oxide (88 mg, 0.4 mmol, 1.0 equiv.), 4-vinylphenyl acetate (0.031 mL, 0.2 mmol, 0.5 equiv.), 1.8M LDA (0.67 mL, 1.2 mmol, 3.0 equiv.), dry THF (4.0 mL, 0.1M). Aqueous work up was performed followed by purification by FCC on silica gel (5% MeOH in EtOAc) to isolate product as a white amorphous solid (51% yield, 33 mg, 0.102 mmol). (Rf = 0.31 9:1 DCM/MeOH).

^{1}H NMR (400 MHz, $CDCl_3$) δ 6.96 (d, J = 8.4 Hz, 2H), 6.71 (d, J = 8.5 Hz, 2H), 5.12 (br s, 1H), 3.70 (s, 2H), 3.28 (s, 1H), 1.99 (s, 4H), 1.74 (s, 6H), 1.56 (s, 7H), 1.39 – 1.16 (m, 4H).

^{13}C NMR (101 MHz, $CDCl_3$) δ 154.1, 129.4, 127.9, 115.3, 60.3, 56.2, 53.1, 46.9, 42.9, 36.7, 36.3, 32.0, 30.0, 24.7.

IR: 2976, 2887, 2851, 1864, 1616, 1283, 1208, 1188, 1125, 1096, 1000, 967, 924 cm-1

HRMS (ESI) m/z: $[M+H]^+$ Calc'd for $C_{22}H_{30}NO$ 324.2322; Found 324.2292

4-(-7-(2,4,4-trimethylpentan-2-yl)-7-azabicyclo[2.2.1]heptan-2-yl)phenol (10fc)

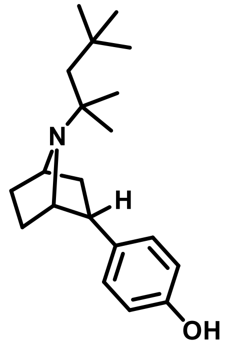

Azanorbornane was prepared according to the general procedure using 1-(2,4,4-trimethylpentan-2-yl)pyrrolidine 1-oxide (119 mg, 0.598 mmol, 1.0 equiv.), 4-vinylphenyl acetate (23 mg, 0.149 mmol, 0.25 equiv.), 1.43M LDA (1.30 mL, 1.79 mmol, 3.0 equiv.), dry THF (3.0 mL, 0.1M). Aqueous work up was performed followed by purification by FCC on silica gel (20% EtOAc in Hexanes) to isolate product as a light yellow amorphous solid (56% yield, 25 mg, 0.083 mmol). (Rf = 0.56 1:1 Hex/EtOAc).

^{1}H NMR (400 MHz, $CDCl_3$) δ 7.06 (d, J = 8.4 Hz, 2H), 6.77 (d, J = 8.5 Hz, 2H), 3.64 (t, J = 4.2 Hz, 2H), 3.33 – 3.25 (m, 1H), 2.04 (t, J = 11.9 Hz, 1H), 1.63 (ddd, J = 12.9, 9.7, 5.3 Hz, 1H), 1.44 (s, 2H), 1.39 – 1.23 (m, 4H), 1.16 (s, 6H), 1.03 (s, 9H).

^{13}C NMR (126 MHz, $CDCl_3$) δ 153.5, 135.0, 129.6, 115.0, 60.7, 56.4, 55.6, 55.0, 47.2, 36.5, 32.1, 32.0, 31.9, 29.6, 29.3, 24.8.

IR: 3306, 2951, 2900, 2872, 1702, 1612 1227, 1173, 1141, 1108, 1065, 1050, 1014, 985 cm-1

HRMS (ESI) m/z: $[M+H_3O]^+$ Calc'd for $C_{20}H_{34}NO_2$ 320.2584; Found 320.2594

7-(*tert*-butyl)-2-(4-((*tert*-butyldimethylsilyl)oxy)phenyl)-7-azabicyclo[2.2.1]heptane (10ga)

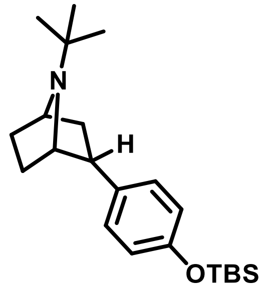

Azanorbornane was prepared according to the general procedure using 1-(*tert*-butyl)pyrrolidine 1-oxide (57 mg, 0.4 mmol, 1.0 equiv.), *tert*-butyldimethyl(4-vinylphenoxy)silane (47 mg, 0.2 mmol, 0.5 equiv.), 1.8M LDA (0.67 mL, 1.2 mmol, 3.0 equiv.), dry THF (4.0 mL, 0.1M). Quenched with DI H_2O and extracted with diethyl ether (x2) followed by purification by FCC on silica gel (10% EtOAc in Hexanes) to isolate product as a light yellow oil (95% yield, 68 mg, 0.190 mmol). (Rf = 0.41 1:1 Hexanes/EtOAc)

^{1}H NMR (400 MHz, $CDCl_3$) δ 7.07 (d, *J* = 8.0 Hz, 2H), 6.79 (d, *J* = 8.3 Hz, 2H), 3.68 (t, *J* = 3.3 Hz, 2H), 3.43 – 3.27 (m, 1H), 2.07 (t, *J* = 10.4 Hz, 1H), 1.77 – 1.63 (m, *J* = 12.2, 6.6 Hz, 1H), 1.48 – 1.27 (m, 4H), 1.19 (s, 9H), 1.00 (s, 9H), 0.21 (s, 6H).

^{13}C NMR (126 MHz, $CDCl_3$) δ 153.6, 135.1, 129.2, 119.7, 61.1, 56.89, 51.8, 47.4, 36.4, 31.8, 30.3, 25.8, 24.4, 18.4, -4.3.

IR: 2956, 2929, 2858, 1607, 1255, 1318, 1255, 1229, 1171, 1108, 1056, 1013 cm-1

HRMS (ESI) m/z: $[M+H]^+$ Calc'd for $C_{22}H_{38}NOSi$ 360.2717; Found 360.2688

7-(adamantan-1-yl)-2-(4-((*tert*-butyldimethylsilyl)oxy)phenyl)-7-azabicyclo[2.2.1]heptane (10gb)

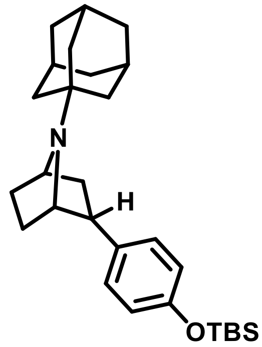

Azanorbornane was prepared according to the general procedure using 1-(adamant-1-yl)pyrrolidine 1-oxide (88 mg, 0.4 mmol, 1.0 equiv.), *tert*-butyldimethyl(4-vinylphenoxy)silane (47 mg, 0.2 mmol, 0.5 equiv.), 1.8M LDA (0.67 mL, 1.2 mmol, 3.0 equiv.), dry THF (4.0 mL, 0.1M). Quenched with DI H_2O and extracted with diethyl ether (x2) followed by purification by FCC on silica gel (10% EtOAc in Hexanes) to isolate product as a white amorphous solid (96% yield, 84 mg, 0.192 mmol). (Rf = 0.59 3:1 Hexanes/EtOAc).

^{1}H NMR (400 MHz, $CDCl_3$) δ 7.04 (d, *J* = 8.5 Hz, 2H), 6.76 (d, *J* = 8.5 Hz, 2H), 3.72 (t, *J* = 4.3 Hz, 2H), 3.33 – 3.24 (m, 1H), 2.05 (s, 3H), 2.02 – 1.93 (m, 1H), 1.77 (d, *J* = 2.3 Hz, 6H), 1.63 (m, 7H), 1.44 – 1.24 (m, 4H), 0.98 (s, 9H), 0.19 (s, 6H).

^{13}C NMR (101 MHz, $CDCl_3$) δ 153.6, 135.2, 129.2, 119.7, 59.8, 55.5, 52.1, 47.6, 43.3, 36.9, 36.6, 32.2, 30.0, 25.8, 24.9, 18.3, -4.3.

IR: 2962, 2900, 2849, 1607 1245, 1188, 1167, 1136, 1097, 1074, 1011, 983 cm-1

HRMS (ESI) m/z: $[M+Na]^+$ Calc'd for $C_{28}H_{43}NOSiNa$ 460.3006; Found 460.3049

2-(4-((*tert*-butyldimethylsilyl)oxy)phenyl)-7-(2,4,4-trimethylpentan-2-yl)-7-azabicyclo[2.2.1]heptane (10gc)

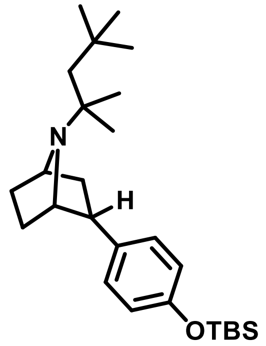

Azanorbornane was prepared according to the general procedure using 1-(2,4,4-trimethylpentan-2-yl)pyrrolidine 1-oxide (116 mg, 0.581 mmol, 1.0 equiv.), *tert*-butyldimethyl(4-vinylphenoxy)silane (34 mg, 0.145 mmol, 0.25 equiv.), 1.82M LDA (0.96 mL, 1.74 mmol, 3.0 equiv.), dry THF (5.8 mL, 0.1M). Quenched with DI H_2O and extracted with diethyl ether (x2) followed by purification by FCC on silica gel (5-10% EtOAc in Hexanes) to isolate product as a light yellow oil (96% yield, 58 mg, 0.139 mmol). (Rf = 0.86 3:1 Hex/EtOAc).

^{1}H NMR (400 MHz, $CDCl_3$) δ 7.03 (d, *J* = 8.6 Hz, 2H), 6.74 (d, *J* = 8.5 Hz, 2H), 3.60 (t, *J* = 8.0 Hz, 2H), 3.33 – 3.19 (m, 1H), 2.01 (dd, *J* = 12.7, 9.4 Hz, 1H), 1.61 (m, 1H), 1.43 (s, 2H), 1.38 – 1.22 (m, 4H), 1.14 (s, 6H), 1.02 (s, 9H), 0.96 (s, 9H), 0.17 (s, 6H).

^{13}C NMR (101 MHz, $CDCl_3$) δ 153.5, 135.4, 129.3, 119.7, 60.8, 56.4, 55.6, 55.0, 47.3, 36.4, 32.1, 32.0, 31.9, 29.6, 29.3, 25.9, 24.8, 18.4, -4.3.

IR: 2952, 2899, 1738, 1608, 1227, 1172, 1141, 1106, 1065, 1050, 1014, 985 cm-1

HRMS (ESI) m/z: $[M+H]^+$ Calc'd for $C_{26}H_{45}NOSi$ 416.3343; Found 416.3350

7-(*tert*-butyl)-2-(4-fluorophenyl)-7-azabicyclo[2.2.1]heptane (10ha)

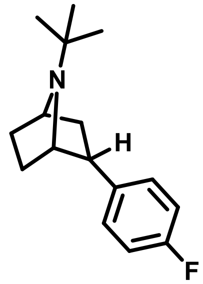

Azanorbornane was prepared according to the general procedure using 1-(*tert*-butyl)pyrrolidine 1-oxide (72 mg, 0.5 mmol, 1.0 equiv.), 1-fluoro-4-vinylbenzene (0.030 mL, 0.25 mmol, 0.5 equiv.), 1.82M LDA (0.82 mL, 1.5 mmol, 3.0 equiv.), dry THF (4.5 mL, 0.1M). Aqueous work up was performed followed by purification by FCC on silica gel (35% EtOAc in hexanes) to isolate product as a yellow oil (42% yield, 26 mg, 0.105 mmol). The minor diastereomer could not be completely separated but was less than 5% (Major diastereomer Rf = 0.28 1:1 Hexanes/EtOAc).

^{1}H NMR (400 MHz, $CDCl_3$) δ 7.32 minor (t, *J* = 7.5 Hz), 7.23 minor (d, *J* = 7.8 Hz), 7.17 (dd, *J* = 8.4, 5.6 Hz, 2H), 7.00 (t, *J* = 8.7 Hz, 2H), 3.70 (d, *J* = 3.2 Hz, 2H), 3.42 – 3.32 (m, 1H), 2.11 (t, *J* = 9.8 Hz, 1H), 1.77 – 1.62 (m, 1H), 1.38 (ddd, *J* = 16.0, 12.4, 5.4 Hz, 4H), 1.20 (s, 9H).

^{13}C NMR (126 MHz, $CDCl_3$) δ 161.1 (d, *J* = 244.4 Hz), 138.2 (d, *J* = 3.8 Hz) 129.7 (d, *J* = 7.6 Hz), 128.3 (d, *J* = 30.2 Hz) minor, 114.9 (d, *J* = 21.4 Hz), 60.9, 56.8, 51.8, 47.8 minor, 47.4, 36.6, 31.8, 30.3, 24.4, 21.5 minor.

^{19}F NMR (376 MHz, $CDCl_3$) δ -117.98.

IR: 2969, 2871, 1603, 1359, 1268, 1223, 1182, 1160, 1105, 1056, 1028, 1013, 948 cm-1

HRMS (ESI) m/z: $[M+Na]^+$ Calc'd for $C_{16}H_{22}FNNa$ 270.1628; Found 270.1631

7-(*tert*-butyl)-2-(4-(trifluoromethyl)phenyl)-7-azabicyclo[2.2.1]heptane (10ia)

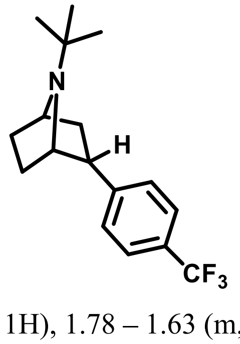

Azanorbornane was prepared according to the general procedure using 1-(*tert*-butyl)pyrrolidine 1-oxide (57 mg, 0.4 mmol, 1.0 equiv.), 1-(trifluoromethyl)-4-vinylbenzene (0.029 mL, 0.2 mmol, 0.5 equiv.), 1.8M LDA (0.67 mL, 1.2 mmol, 3.0 equiv.), dry THF (4.0 mL, 0.1M). Aqueous work up was performed followed by purification by FCC on silica gel (35% EtOAc in hexanes) to isolate product as a yellow oil (40% yield, 24 mg, 0.080 mmol). (Rf = 0.30 1:1 Hexanes/EtOAc).

^{1}H NMR (400 MHz, $CDCl_3$) δ 7.57 (d, *J* = 8.1 Hz, 2H), 7.33 (d, *J* = 8.0 Hz, 2H), 3.78 (t, *J* = 4.3 Hz, 1H), 3.73 (t, *J* = 4.4 Hz, 1H), 3.50 – 3.40 (m, 1H), 2.11 (t, *J* = 11.8 Hz, 1H), 1.78 – 1.63 (m, 1H), 1.46 (ddd, *J* = 17.8, 13.0, 8.0 Hz, 2H), 1.36 – 1.28 (m, 2H), 1.21 (s, 9H).

^{13}C NMR (101 MHz, $CDCl_3$) δ 146.9, 128.7, 128.0 (q, *J* = 31.5 Hz), 124.9 (q, *J* = 4.0 Hz), 124.5 (q, *J* = 272.2 Hz), 60.8, 56.8, 51.9, 48.2, 36.3, 31.8, 30.2, 24.6.

^{19}F NMR (376 MHz, $CDCl_3$) δ -62.25.

IR: 2966, 2951, 2870, 1616, 1298, 1270, 1257, 1197, 1179, 1155, 1111, 1068, 1025, 1015, 983, 953, 945 cm-1

HRMS (ESI) m/z: $[M+Na]^+$ Calc'd for $C_{17}H_{22}F_3NNa$ 320.1597; Found 320.1612

7-(adamantan-1-yl)-2-(4-(trifluoromethyl)phenyl)-7-azabicyclo[2.2.1]heptane (10ib)

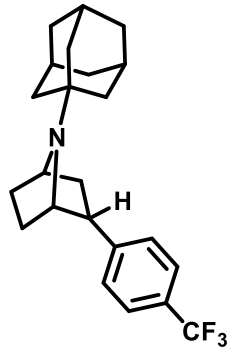

Azanorbornane was prepared according to the general procedure using 1-(adamant-1-yl)pyrrolidine 1-oxide (88 mg, 0.4 mmol, 1.0 equiv.), 1-(trifluoromethyl)-4-vinylbenzene (0.029 mL, 0.2 mmol, 0.5 equiv.), 1.8M LDA (0.67 mL, 1.2 mmol, 3.0 equiv.), dry THF (4.0 mL, 0.1M). Aqueous work up was performed followed by purification by FCC on silica gel (10% MeOH in DCM) to isolate product as a yellow oil (48% yield, 36 mg, 0.096 mmol). (Rf = 0.49 1:3 Hexanes/EtOAc).

^{1}H NMR (400 MHz, $CDCl_3$) δ 7.56 (d, *J* = 8.1 Hz, 2H), 7.33 (d, *J* = 8.1 Hz, 2H), 3.85 (t, *J* = 4.0 Hz, 1H), 3.79 (t, *J* = 4.8 Hz, 1H), 3.44 – 3.37 (m, 1H), 2.08 (s, 3H), 1.79 (s, 6H), 1.73 – 1.59 (m, 8H), 1.48 (dd, *J* = 12.0, 5.5 Hz, 1H), 1.38 – 1.26 (m, 3H).

^{13}C NMR (101 MHz, $CDCl_3$) δ 147.0, 128.7, 128.0 (q, *J* = 32.3 Hz) 125.1 (q, *J* = 4.0 Hz), 124.52 (q, *J* = 273.7 Hz), 59.5, 55.5, 52.1, 48.4, 43.3, 36.9, 36.4, 32.2, 30.0, 25.1.

^{19}F NMR (376 MHz, $CDCl_3$) δ -62.23.

IR: 2902, 2849, 1738, 1617, 1268, 1236, 1186, 1161, 1122, 1067, 988, 938 cm-1

HRMS (ESI) m/z: $[M+H_3O]^+$ Calc'd for $C_{23}H_{31}F_3NO$ 394.2352; Found 394.2376

2-(4-(trifluoromethyl)phenyl)-7-(2,4,4-trimethylpentan-2-yl)-7-azabicyclo[2.2.1]heptane (10ic)

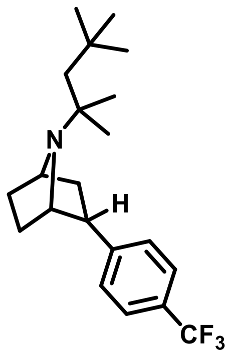

Azanorbornane was prepared according to the general procedure using 1-(2,4,4-trimethylpentan-2-yl)pyrrolidine 1-oxide (126 mg, 0.632 mmol, 1.0 equiv.), 1-(trifluoromethyl)-4-vinylbenzene (0.023 mL, 0.158 mmol, 0.25 equiv.), 1.82M LDA (1.05 mL, 1.90 mmol, 3.0 equiv.), dry THF (6.3 mL, 0.1M). Aqueous work up was performed followed by purification by FCC on silica gel (10% EtOAc in hexanes) to isolate product as a yellow oil (42% yield, 24 mg, 0.067 mmol). (Rf = 0.86 1:1 Hex/EtOAc).

^{1}H NMR (400 MHz, $CDCl_3$) δ 7.53 (d, *J* = 8.2 Hz, 2H), 7.31 (d, *J* = 8.0 Hz, 2H), 3.73 (t, *J* = 4.2 Hz, 1H), 3.68 (t, *J* = 4.7 Hz, 1H), 3.44 – 3.36 (m, 1H), 2.15 – 2.04 (m, 1H), 1.74 – 1.62 (m, 1H), 1.44 (q, *J* = 5.5 Hz, 3H), 1.40 – 1.33 (m, 1H), 1.29 (t, *J* = 8.3 Hz, 2H), 1.18 (s, 6H), 1.04 (s, 9H).

^{13}C NMR (126 MHz, $CDCl_3$) δ 147.2, 128.8, 128.1 (q, *J* = 31.5 Hz), 125.1 (q, *J* = 3.8 Hz), 124.5 (q, *J* = 272.2 Hz), 60.5, 56.4, 55.7, 55.0, 48.2, 36.3, 32.1, 31.9, 29.6, 29.3, 25.0.

^{19}F NMR (376 MHz, $CDCl_3$) δ -62.22.

IR: 2953, 2901, 1737, 1617, 1542, 1214, 1268, 1229, 1164, 1127, 1069, 1017, 986 cm-1

HRMS (ESI) m/z: $[M+H]^+$ Calc'd for $C_{21}H_{31}F_3N$ 354.2403; Found 354.2371

4-(-7-*tert*-butyl)-7-azabicyclo[2.2.1]heptan-2-yl)phenyl)boronic acid (10ja)

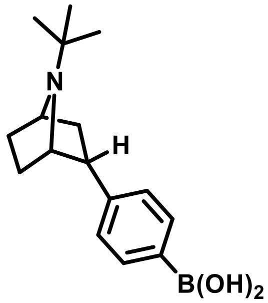

Azanorbornane was prepared according to the general procedure using 1-(*tert*-butyl)pyrrolidine 1-oxide (57 mg, 0.4 mmol, 1.0 equiv.), (4-vinylphenyl)boronic acid (30 mg, 0.2 mmol, 0.5 equiv.), 1.8M LDA (1.0 mL, 1.8 mmol, 4.5 equiv.), dry THF (4.0 mL, 0.1M). Aqueous work up was performed followed by purification by FCC on silica gel (25% EtOAc in hexanes) to isolate product as a yellow oil (80% yield, 44 mg, 0.160 mmol). (Rf = 0.61 1:3 Hex/EtOAc).

^{1}H NMR (400 MHz, $CDCl_3$) δ 7.29 (d, *J* = 7.5 Hz, 1H), 7.21 (d, *J* = 7.4 Hz, 2H) 7.19 minor (d, *J* = 7.3 Hz), 3.70 (d, *J* = 8.1, 2H), 3.43 – 3.36 (m, 1H), 2.08 (t, *J* = 12.1 Hz, 1H), 1.66 (d, *J* = 14.8, Hz, 1H), 1.46 (dd, *J* = 11.7, 5.6 Hz, 1H), 1.41 – 1.28 (m, 3H), 1.18 (s, 9H).

^{13}C NMR (101 MHz, $CDCl_3$) δ 142.6, 128.5, 128.3, 125.8, 61.0, 56.8, 51.8, 48.2, 36.2, 31.8, 30.3, 24.6.

IR: 3268, 2968, 2872, 1672, 1603, 1510, 1456, 1406, 1378, 1360, 1298, 1267, 1228, 1200, 1180, 1124, 1084, 1056, 1032, 863, 829, 799, 768, 748, 720, 698, 671, 610, 552, 445, 417, 407 cm-1

HRMS (ESI) m/z: $[M+Na]^+$ Calc'd for $C_{16}H_{24}BNO_2Na$ 296.1793; Found 296.1777

7-(*tert*-butyl)-2-(4-((*tert*-butyldimethylsilyl)oxy)-3-methoxyphenyl)-7-azabicyclo[2.2.1]heptane (10ka)

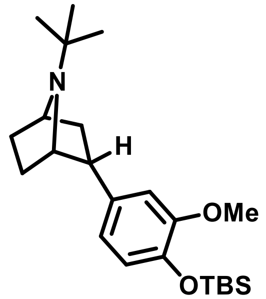

Azanorbornane was prepared according to the general procedure using 1-(*tert*-butyl)pyrrolidine 1-oxide (57 mg, 0.4 mmol, 1.0 equiv.), *tert*-butyl(2-methoxy-4-vinylphenoxy)dimethylsilane (53 mg, 0.2 mmol, 0.5 equiv.), 1.8M LDA (0.67 mL, 1.2 mmol, 3.0 equiv.), dry THF (4.0 mL, 0.1M). Quenched with DI H_2O and extracted with diethyl ether (x2) followed by purification by FCC on silica gel (15% EtOAc in hexanes) to isolate product as a light yellow oil (85% yield, 66 mg, 0.170 mmol). (Rf = 0.51 1:1 Hexanes/EtOAc).

^{1}H NMR (400 MHz, $CDCl_3$) δ 6.77 (d, J = 8.0 Hz, 1H), 6.67 (d, J = 5.0 Hz, 1H), 6.65 (s, 1H), 3.80 (s, 3H), 3.65 (t, J = 4.2 Hz, 2H), 3.36 – 3.26 (m, 1H), 2.03 (tt, J = 11.1, 3.7 Hz, 1H), 1.72 – 1.59 (m, 1H), 1.43 – 1.26 (m, 4H), 1.17 (s, 9H), 0.99 (s, 9H), 0.15 (s, 6H).

^{13}C NMR (126 MHz, $CDCl_3$) δ 150.6, 142.9, 136.0, 120.5, 120.2, 112.6, 61.2, 56.8, 55.6, 51.8, 47.7, 36.3, 31.7, 30.3, 25.9, 24.4, 18.5, 18.5, 18.5, -4.6, -4.6.

IR: 2956, 2929, 2857, 1605, 1256, 1232, 1181, 1156, 1128, 1041 cm-1

HRMS (ESI) m/z: $[M+Na]^+$ Calc'd for $C_{23}H_{39}NO_2SiNa$ 412.2642; Found 412.2680

7-(adamantan-1-yl)-2-(4-((tert-butyldimethylsilyl)oxy)-3-methoxyphenyl)-7-azabicyclo[2.2.1]heptane (10kb)

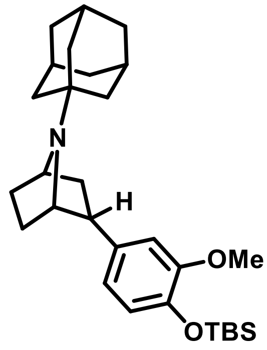

Azanorbornane was prepared according to the general procedure using 1-(adamant-1-yl)pyrrolidine 1-oxide (88 mg, 0.4 mmol, 1.0 equiv.), *tert*-butyl(2-methoxy-4-vinylphenoxy)dimethylsilane (53 mg, 0.2 mmol, 0.5 equiv.), 1.8M LDA (0.67 mL, 1.2 mmol, 3.0 equiv.), dry THF (4.0 mL, 0.1M). Quenched with DI H_2O and extracted with diethyl ether (x2) followed by purification by FCC on silica gel (10% EtOAc in hexanes) to isolate product as an off-white amorphous solid (96% yield, 90 mg, 0.192 mmol). (Rf = 0.49 1:1 Hexanes/EtOAc).

^{1}H NMR (400 MHz, $CDCl_3$) δ 6.77 (d, J = 8.0 Hz, 1H), 6.68 (d, J = 1.7, 1H), 6.65 (d, J = 8.1 Hz, 1H), 3.80 (s, 3H), 3.72 (t, J = 4.3 Hz, 2H), 3.33 – 3.25 (m, 1H), 2.09 – 1.96 (m, 4H), 1.78 (s, 6H), 1.64 (s, 7H), 1.44 – 1.35 (m, 2H), 1.30 (dd, J = 13.9, 6.3 Hz, 2H), 0.99 (s, 9H), 0.15 (s, 6H).

^{13}C NMR (126 MHz, $CDCl_3$) δ 150.6, 143.0, 136.1, 120.5, 120.2, 112.8, 59.8, 55.7, 55.5, 52.1, 48.0, 43.3, 36.9, 36.5, 32.2, 30.0, 25.9, 25.0, 18.6, -4.5, -4.5.

IR: 2970, 2900, 2849, 1739, 1606, 1280, 1263, 1247, 1228, 1185, 1155, 1139, 1124, 1097, 1076, 1034, 1011 cm-1

HRMS (ESI) m/z: $[M+H3O]^+$ Calc'd for $C_{29}H_{48}NO_3Si$ 486.3398; Found 486.3376

7-(*tert*-butyl)-2-(2-((*tert*-butyldimethylsilyl)oxy)phenyl)-7-azabicyclo[2.2.1]heptane (10la)

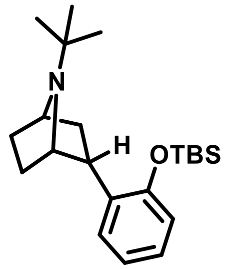

Azanorbornane was prepared according to the general procedure using 1-(*tert*-butyl)pyrrolidine 1-oxide (57 mg, 0.4 mmol, 1.0 equiv.), *tert*-butyldimethyl(2-vinylphenoxy)silane (47 mg, 0.2 mmol, 0.5 equiv.), 1.8M LDA (0.67 mL, 1.2 mmol, 3.0 equiv.), dry THF (4.0 mL, 0.1M). Quenched with DI H_2O and extracted with diethyl ether (x2) followed by purification by FCC on silica gel (10% EtOAc in Hexanes) to isolate product as a yellow oil (90% yield, 45 mg, 0.190 mmol). (Rf = 0.40 1:1 Hexanes/EtOAc).

^{1}H NMR (400 MHz, $CDCl_3$) δ 7.21 (dd, *J* = 7.7, 1.5 Hz, 1H), 7.08 (td, *J* = 7.7, 1.7 Hz, 1H), 6.91 (td, *J* = 7.5, 1.1 Hz, 1H), 6.80 (dd, *J* = 8.0, 1.2 Hz, 1H), 3.80 (t, *J* = 4.3 Hz, 1H), 3.68 (t, *J* = 4.4 Hz, 1H), 3.63 – 3.55 (m, 1H), 1.93 (tdd, *J* = 11.7, 4.5, 3.5 Hz, 1H), 1.73 – 1.62 (m, 1H), 1.46 (dd, *J* = 12.0, 5.6 Hz, 1H), 1.42 – 1.22 (m, 3H), 1.17 (s, 9H), 1.05 (s, 9H), 0.30 (s, 3H), 0.24 (s, 3H).

^{13}C NMR (126 MHz, $CDCl_3$) δ 154.7, 132.4, 128.2, 126.6, 120.7, 118.2, 59.4, 56.7, 51.8, 42.5, 35.7, 31.6, 30.4, 26.2, 26.1, 24.8, 18.5, -3.5, -4.0.

IR: 2957, 2929, 2858, 1251, 1178, 1156, 1116, 1098, 1065, 1007, 985 cm-1

HRMS (ESI) m/z: $[M+H_3O]^+$ Calc'd for $C_{22}H_{40}NO_2Si$ 378.2837; Found 378.2837

7-(adamantan-1-yl)-2-(2-((*tert*-butyldimethylsilyl)oxy)phenyl)-7-azabicyclo[2.2.1]heptane (10lb)

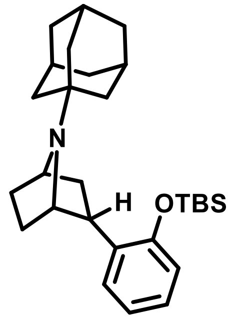

Azanorbornane was prepared according to the general procedure using 1-(adamant-1-yl)pyrrolidine 1-oxide (88 mg, 0.4 mmol, 1.0 equiv.), *tert*-butyldimethyl(2-vinylphenoxy)silane (47 mg, 0.2 mmol, 0.5 equiv.), 1.8M LDA (0.67 mL, 1.2 mmol, 3.0 equiv.), dry THF (4.0 mL, 0.1M). Quenched with DI H_2O and extracted with diethyl ether (x2) followed by purification by FCC on silica gel (15% EtOAc in Hexanes) to isolate product as a white amorphous solid (70% yield, 123 mg, 0.28 mmol). (Rf = 0.58 3:1 Hexanes/EtOAc).

^{1}H NMR (400 MHz, $CDCl_3$) δ 7.21 (dd, *J* = 7.7, 1.6 Hz, 1H), 7.06 (td, *J* = 7.7, 1.7 Hz, 1H), 6.90 (td, *J* = 7.5, 1.2 Hz, 1H), 6.79 (dd, *J* = 8.0, 1.2 Hz, 1H), 3.84 (t, *J* = 4.0 Hz, 1H), 3.74 (t, *J* = 4.2 Hz, 1H), 3.63 – 3.53 (m, 1H), 2.04 (s, 3H), 1.94 – 1.84 (m, 1H), 1.77 (d, *J* = 3.0 Hz, 6H), 1.63 (s, 6H), 1.47 (dd, *J* = 12.0, 5.5 Hz, 1H), 1.41 – 1.33 (m, 1H), 1.32 – 1.19 (m, 3H), 1.05 (s, 9H), 0.30 (s, 3H), 0.24 (s, 3H).

^{13}C NMR (126 MHz, $CDCl_3$) δ 154.6, 132.5, 128.0, 126.5, 120.5, 118.0, 57.9, 55.3, 52.0, 43.2, 42.4, 36.8, 35.6, 32.0, 29.9, 26.1, 25.2, 18.4, -3.6, -4.0.

IR: 2902, 2849, 1738, 1251, 1217, 1186, 1138, 1096, 1047, 1010, 932 cm-1

HRMS (ESI) m/z: $[M+Na]^+$ Calc'd for $C_{28}H_{43}NOSiNa$ 460.3006; Found 460.2975

9-(4-(-7-(tert-butyl)-7-azabicyclo[2.2.1]heptan-2-yl)phenyl)-9H-carbazole (10ma)

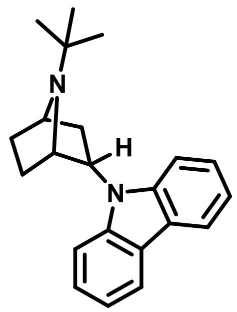

Azanorbornane was prepared according to the general procedure using 1-(*tert*-butyl)pyrrolidine 1-oxide (57 mg, 0.4 mmol, 1.0 equiv.), 9-vinyl-9H-carbazole (38 mg, 0.2 mmol, 0.5 equiv.), 1.8M LDA (0.67 mL, 1.2 mmol, 3.0 equiv.), dry THF (4.0 mL, 0.1M). Aqueous work up was performed followed by purification by FCC on silica gel (5% MeOH in DCM) to isolate product as an off-white amorphous solid (80% yield, 51 mg, 0.160 mmol). (Rf = 0.61 1:3 Hex/EtOAc).

^{1}H NMR (400 MHz, $CDCl_3$) δ 8.09 (d, *J* = 7.7 Hz, 2H), 7.56 (d, *J* = 8.4 Hz, 2H), 7.42 (t, *J* = 7.7 Hz, 2H), 7.22 (t, *J* = 7.4 Hz, 2H), 4.96 – 4.86 (m, 1H), 4.10 (t, *J* = 4.4 Hz, 1H), 3.89 (t, *J* = 3.9 Hz, 1H), 2.52 (dd, *J* = 12.7, 5.3 Hz, 1H), 2.26 (dt, *J* = 12.3 Hz 1H), 1.86 (dd, *J* = 13.2, 5.8 Hz, 2H), 1.79 – 1.69 (m, 1H), 1.66 – 1.56 (m, 1H), 1.22 (s, 9H).

^{13}C NMR (125 MHz, $CDCl_3$) δ 141.8, 125.7, 123.6, 120.2, 119.0, 110.6, 60.0, 59.9, 57.3, 51.8, 32.2, 31.7, 30.2, 25.2.

IR: 3055, 2975, 2920, 2896, 2865, 1886, 1666, 1625, 1278, 1224, 1186, 1150, 1123, 1085, 1066, 1029, 1003, 992, 976, 962, 938 cm-1

HRMS (ESI) m/z: $[M+H]^+$ Calc'd for $C_{22}H_{27}N_2$ 319.2169; Found 319.2159

9-(4-(-7-(adamantan-1-yl)-7-azabicyclo[2.2.1]heptan-2-yl)phenyl)-9H-carbazole (10mb)

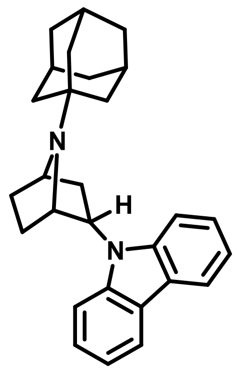

Azanorbornane was prepared according to the general procedure using 1-(adamant-1-yl)pyrrolidine 1-oxide (88 mg, 0.4 mmol, 1.0 equiv.), 9-vinyl-9H-carbazole (38 mg, 0.2 mmol, 0.5 equiv.), 1.8M LDA (0.67 mL, 1.2 mmol, 3.0 equiv.), dry THF (4.0 mL, 0.1M). Aqueous work up was performed followed by purification by FCC on silica gel (5 % EtOAc in Hexanes) to isolate product as a white amorphous solid (90% yield, 71 mg, 0.180 mmol). (Rf = 0.73 3:1 Hex/EtOAc).

Crystals of suitable quality for single crystal X-ray diffraction analysis of the tetrafluoroborate salt, [**10mb•H]BF$_4$** were prepared as follows. Compound **10mb** (50 mg, 0.12 mmol) was dissolved in 4 mL of DCM, and 50 μL (0.40 mmol) of 48% w/w tetrafluoroboric acid was added. After stirring for 30 minutes, 0.50 g $MgSO_4$ was added to the solution, and the mixture was filtered using a 0.20 μm PTFE syringe filter. The solution was layered with 5 mL of Et_2O and stored at -35 °C for 5 days, after which crystals of suitable quality for analysis had grown.

^{1}H NMR (400 MHz, $CDCl_3$) δ 8.09 (d, *J* = 7.7 Hz, 2H), 7.55 (d, *J* = 8.3 Hz, 2H), 7.42 (t, *J* = 7.7 Hz, 2H), 7.21 (t, *J* = 7.4 Hz, 2H), 4.96 – 4.83 (m, 1H), 4.17 (t, *J* = 4.0 Hz, 1H), 3.95 (t, *J* = 3.8 Hz, 1H), 2.51 (dd, *J* = 12.7, 5.2 Hz, 1H), 2.21 (t, *J* = 11.2 Hz, 1H), 2.09 (s, 3H), 1.91 – 1.71 (m, 9H), 1.71 – 1.62 (m, 6H), 1.53 (t, *J* = 11.8 Hz, 1H).

^{13}C NMR (101 MHz, $CDCl_3$) δ 141.7, 125.6, 123.6, 120.1, 118.9, 110.7, 60.1, 58.6, 56.0, 52.1, 43.3, 36.8, 32.2, 32.2, 29.9, 25.7.

IR: 2899, 2847, 1625, 1269, 1239, 1221, 1179, 1156, 1122, 1099, 1030, 1004, 976 cm-1

HRMS (ESI) m/z: $[M+Na]^+$ Calc'd for $C_{28}H_{32}N_2Na$ 419.2457; Found 419.2455

9-(4-(-7-(2,4,4-trimethylpentan-2-yl)-7-azabicyclo[2.2.1]heptan-2-yl)phenyl)-9H-carbazole (10mc)

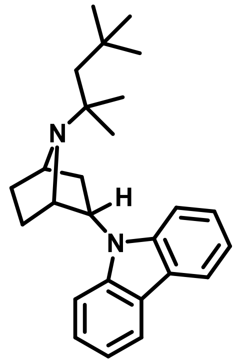

Azanorbornane was prepared according to the general procedure using 1-(2,4,4-trimethylpentan-2-yl)pyrrolidine 1-oxide (84 mg, 0.459 mmol, 1.0 equiv.), 9-vinyl-9H-carbazole (22 mg, 0.115 mmol, 0.25 equiv.), 1.69M LDA (0.82 mL, 1.38 mmol, 3.0 equiv.), dry THF (4.5 mL, 0.1M). Quenched with DI H_2O and extracted with diethyl ether (x2) followed by purification by FCC on silica gel (5 % EtOAc in Hexanes) to isolate product as a colorless oil (96% yield, 41 mg, 0.110 mmol). (Rf = 0.82 3:1 Hex/EtOAc).

Alternative purification: Crude mixture was dissolved in hexanes. 1M HCl was added, and the solids were filtered off. The solids were taken into DCM and basified used 15% NaOH. The aqueous layer was extracted with DCM (x2) and dried over $MgSO_4$ to afford a colorless oil.

^{1}H NMR (400 MHz, $CDCl_3$) δ 8.13 (d, J = 7.8 Hz, 2H), 7.56 (d, J = 8.4 Hz, 2H), 7.47 (t, J = 7.1 Hz, 2H), 7.26 (t, J = 2H), 4.98 – 4.88 (m, 1H), 4.15 – 4.07 (m, 1H), 3.89 (t, J = 4.1 Hz, 1H), 2.53 (dd, J = 12.7, 5.3 Hz, 1H), 2.30 – 2.19 (m, 1H), 1.87 (dd, J = 8.2, 6.0 Hz, 2H), 1.74 (ddd, J = 14.2, 8.6, 5.6 Hz, 1H), 1.63 – 1.50 (m, 3H), 1.23 (d, J = 5.6 Hz, 6H), 1.11 (s, 9H).

^{13}C NMR (101 MHz, $CDCl_3$) δ 141.8, 125.7, 123.6, 120.2, 119.0, 110.7, 59.8, 59.7, 57.0, 55.8, 54.8, 32.2, 32.0, 31.9, 30.0, 29.1, 25.6.

IR: 2944, 2892, 1921, 1883, 1761, 1270, 1184, 1171, 1123, 1060, 1028, 1016, 1004, 963, 924 cm-1

HRMS (ESI) m/z: $[M+H]^+$ Calc'd for $C_{26}H_{35}N_2$ 375.2794; Found 375.2800

7-(*tert*-butyl)-2-(naphthalen-2-yl)-7-azabicyclo[2.2.1]heptane (10na)

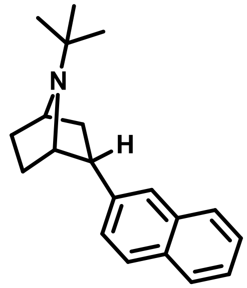

Azanorbornane was prepared according to the general procedure using 1-(*tert*-butyl)pyrrolidine 1-oxide (57 mg, 0.4 mmol, 1.0 equiv.), 2-vinylnaphthalene (31 mg, 0.2 mmol, 0.5 equiv.), 1.8M LDA (0.67 mL, 1.2 mmol, 3.0 equiv.), dry THF (4.0 mL, 0.1M). Aqueous work up was performed followed by purification by FCC on silica gel (20 % EtOAc in Hexanes) to isolate product as an off-white powder (72% yield, 40 mg, 0.144 mmol). (Rf = 0.25 1:1 Hex/EtOAc).

^{1}H NMR (400 MHz, $CDCl_3$) δ 7.83 – 7.74 (m, 3H), 7.64 (s, 1H), 7.49 – 7.39 (m, 2H), 7.35 (d, J = 8.5 Hz, 1H), 3.84 (t, J = 3.7 Hz, 1H), 3.73 (t, J = 4.6 Hz, 1H), 3.59 – 3.51 (m, 1H), 2.22 – 2.09 (m, 1H), 1.77 – 1.57 (m, 3H), 1.38 (t, J = 8.8 Hz, 2H), 1.21 (s, 9H).

^{13}C NMR (126 MHz, $CDCl_3$) δ 140.1, 133.6, 132.0, 127.7, 127.6, 127.6, 127.6, 126.1, 126.0, 125.3, 61.0, 56.9, 51.9, 48.2, 36.0, 31.8, 30.2, 24.7.

IR: 2965, 1269, 1229, 1184, 1160, 1070, 1054, 1026, 946 cm-1

HRMS (ESI) m/z: $[M+H]^+$ Calc'd for $C_{20}H_{26}N$ 280.2060; Found 280.2033

7-(adamantan-1-yl)-2-(naphthalen-2-yl)-7-azabicyclo[2.2.1]heptane (10nb)

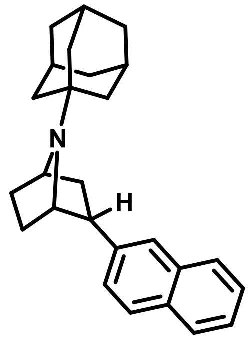

Azanorbornane was prepared according to the general procedure using 1-(adamant-1-yl)pyrrolidine 1-oxide (88 mg, 0.4 mmol, 1.0 equiv.), 2-vinylnaphthalene (31 mg, 0.2 mmol, 0.5 equiv.), 1.8M LDA (0.67 mL, 1.2 mmol, 3.0 equiv.), dry THF (4.0 mL, 0.1M). Aqueous work up was performed followed by purification by FCC on silica gel (20 % EtOAc in Hexanes) to isolate product as a white amorphous solid (72% yield, 51 mg, 0.144 mmol). (Rf = 0.30 1:1 Hex/EtOAc).

^{1}H NMR (400 MHz, $CDCl_3$) δ 7.83 – 7.73 (m, J = 13.0, 7.8 Hz, 3H), 7.63 (s, 1H), 7.48 – 7.38 (m, 2H), 7.35 (d, J = 8.5 Hz, 1H), 3.96 – 3.86 (m, J = 7.3, 3.9 Hz, 1H), 3.79 (s, 1H), 3.59 – 3.46 (m, 1H), 2.07 (s, 3H), 1.81 (s, 6H), 1.70 – 1.57 (m, 9H), 1.42 – 1.29 (m, 3H).

^{13}C NMR (125 MHz, $CDCl_3$) δ 133.6, 132.1, 127.7, 127.6, 126.2, 126.0, 125.3, 59.7, 55.6, 52.2, 48.6, 43.3, 36.9, 36.2, 32.3, 30.0, 25.2.

IR: 2911, 2852, 1706, 1189, 1110, 1062, 1047, 1017, 976, 937 cm-1

HRMS (ESI) m/z: $[MH+CH_3CN]^+$ Calc'd for $C_{28}H_{35}N_2$ 399.2795; Found 399.2769

2-(naphthalen-2-yl)-7-(2,4,4-trimethylpentan-2-yl)-7-azabicyclo[2.2.1]heptane (10nc)

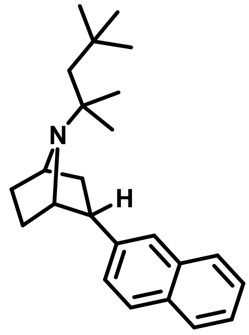

Azanorbornane was prepared according to the general procedure using 1-(2,4,4-trimethylpentan-2-yl)pyrrolidine 1-oxide (117 mg, 0.589 mmol, 1.0 equiv.), 2-vinylnaphthalene (23 mg, 0.147 mmol, 0.25 equiv.), 1.69M LDA (1.05 mL, 1.77 mmol, 3.0 equiv.), dry THF (5 mL, 0.12M). Aqueous work up was performed followed by purification by FCC on silica gel (5 % EtOAc in Hexanes) to isolate product as a white amorphous solid (69% yield, 34 mg, 0.101 mmol). (Rf = 0.80 3:1 Hex/EtOAc).

^{1}H NMR (400 MHz, $CDCl_3$) δ 7.83 – 7.74 (m, 3H), 7.64 (s, 1H), 7.50 – 7.39 (m, 2H), 7.35 (d, J = 8.5 Hz, 1H), 3.82 (t, J = 4.0 Hz, 1H), 3.70 (t, J = 4.5 Hz, 1H), 3.60 – 3.48 (m, 1H), 2.14 (ddd, J = 16.0, 9.9, 4.0 Hz, 1H), 1.71 – 1.54 (m, 3H), 1.48 (s, 2H), 1.35 (s, 2H), 1.20 (s, 6H), 1.06 (s, 9H).

^{13}C NMR (101 MHz, $CDCl_3$) δ 140.5, 133.6, 132.0, 127.8, 127.7, 127.6, 127.6, 126.1, 126.0, 125.2, 60.7, 56.5, 55.7, 55.0, 48.3, 36.1, 32.2, 32.0, 32.0, 29.6, 29.3, 25.1.

IR: 2948, 2893, 2867, 1631, 1266, 1257, 1229, 1200, 1183, 1142, 1097, 1069, 1053, 1022, 964, 950 cm-1

HRMS (ESI) m/z: $[M+H]^+$ Calc'd for $C_{24}H_{34}N$ 336.2686; Found 336.2655

7-(*tert*-butyl)-2-mesityl-7-azabicyclo[2.2.1]heptane (10oa)

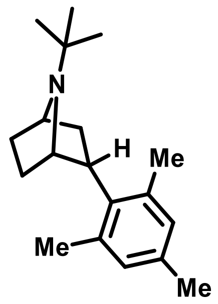

Azanorbornane was prepared according to the general procedure using 1-(*tert*-butyl)pyrrolidine 1-oxide (57 mg, 0.4 mmol, 1.0 equiv.), 1,3,5-trimethyl-2-vinylbenzene (0.032 mL, 0.2 mmol, 0.5 equiv.), 1.8M LDA (0.67 mL, 1.2 mmol, 3.0 equiv.), dry THF (4.0 mL, 0.1M). Aqueous work up was performed followed by purification by FCC on silica gel (20% EtOAc in hexanes) to isolate product as a white amorphous solid (80% yield, 43 mg, 0.160 mmol). (Rf = 0.40 1:1 Hex/EtOAc).

^{1}H NMR (400 MHz, $CDCl_3$) δ 6.85 (s, 2H), 3.82 – 3.71 (m, 2H), 3.62 – 3.49 (m, 1H), 2.44 (s, 6H), 2.26 (s, 3H), 2.12 (dd, *J* = 12.3, 6.5 Hz, 1H), 1.91 (tdd, *J* = 12.2, 4.7, 3.0 Hz, 1H), 1.72 (ddd, *J* = 12.0, 10.2, 4.5 Hz, 1H), 1.64 – 1.56 (m, 1H), 1.48 (dd, *J* = 9.4, 5.2 Hz, 2H), 1.22 (s, 9H).

^{13}C NMR (126 MHz, $CDCl_3$) δ 137.9, 134.9, 134.7, 130.9, 60.6, 57.4, 51.7, 47.0, 32.8, 32.3, 30.4, 25.7, 23.0, 20.6.

IR: 2964, 2870, 1736, 1612, 1293, 1266, 1227, 1203, 1175, 1163, 1107, 1056, 1032, 985, 939 cm-1

HRMS (ESI) m/z: $[M+H_3O]^+$ Calc'd for $C_{19}H_{32}NO$ 290.2478; Found 290.2494

7-(adamantan-1-yl)-2-mesityl-7-azabicyclo[2.2.1]heptane (10ob)

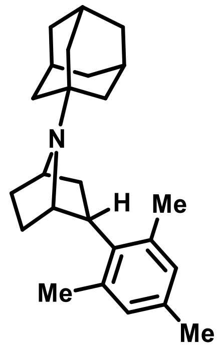

Azanorbornane was prepared according to the general procedure using 1-(adamant-1-yl)pyrrolidine 1-oxide (88 mg, 0.4 mmol, 1.0 equiv.), 1,3,5-trimethyl-2-vinylbenzene (0.032 mL, 0.2 mmol, 0.5 equiv.), 1.8M LDA (0.67 mL, 1.2 mmol, 3.0 equiv.), dry THF (4.0 mL, 0.1M). Aqueous work up was performed followed by purification by FCC on silica gel (20 % EtOAc in hexanes) to isolate product as an off-white amorphous solid (82% yield, 57 mg, 0.164 mmol). (Rf = 0.43 1:1 Hex/EtOAc).

^{1}H NMR (400 MHz, $CDCl_3$) δ 6.82 (s, 2H), 3.80 (dt, *J* = 7.9, 4.0 Hz, 2H), 3.54 – 3.43 (m, 1H), 2.41 (s, 6H), 2.23 (s, 3H), 2.06 (s, 4H), 1.79 (s, 7H), 1.64 (m, 8H), 1.51 – 1.36 (m, 2H).

^{13}C NMR (126 MHz, $CDCl_3$) δ 137.9, 134.9, 134.8, 130.9, 59.3, 56.1, 52.1, 47.3, 43.4, 36.9, 33.0, 32.7, 30.0, 26.0, 23.0, 20.6.

IR: 2974, 2913, 2898, 2847, 1724, 1699, 1609, 1269, 1231, 1203, 1195, 1178, 1107, 1100, 1080, 1016, 989, 966 cm-1

HRMS (ESI) m/z: $[M+Na]^+$ Calc'd for $C_{25}H_{35}NNa$ 372.2662; Found 372.2676

2-mesityl-7-(2,4,4-trimethylpentan-2-yl)-7-azabicyclo[2.2.1]heptane (10oc)

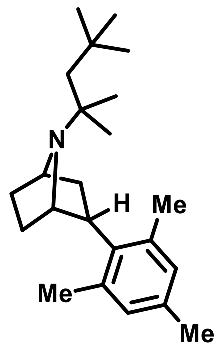

Azanorbornane was prepared according to the general procedure using 1-(2,4,4-trimethylpentan-2-yl)pyrrolidine 1-oxide (146 mg, 0.732 mmol, 1.0 equiv.), 1,3,5-trimethyl-2-vinylbenzene (0.030 mL, 0.183 mmol, 0.25 equiv.), 1.43M LDA (1.55 mL, 2.20 mmol, 3.0 equiv.), dry THF (5.0 mL, 0.15M). Aqueous work up was performed followed by purification by FCC on silica gel (10% EtOAc in hexanes) to isolate product as a colorless oil (94% yield, 56 mg, 0.172 mmol). (Rf = 0.70 1:1 Hex/EtOAc).

^{1}H NMR (400 MHz, $CDCl_3$) δ 6.83 (s, 2H), 3.76 – 3.68 (m, 2H), 3.57 – 3.48 (m, 1H), 2.42 (s, 6H), 2.24 (s, 3H), 2.11 (dd, *J* = 12.2, 6.4 Hz, 1H), 1.91 – 1.81 (m, 1H), 1.69 (dq, *J* = 11.4, 6.2 Hz, 1H), 1.61 – 1.51 (m, 2H), 1.46 (s, 2H), 1.44 (d, *J* = 8.5 Hz, 1H), 1.22 (s, 3H), 1.20 (s, 3H), 1.05 (s, 9H).

^{13}C NMR (126 MHz, $CDCl_3$) δ 137.9, 134.8, 134.8, 130.9, 60.3, 57.0, 55.6, 55.0, 46.9, 32.6, 32.3, 32.2, 31.9, 30.1, 28.9, 26.0, 23.1, 20.6.

IR: 2952, 2873, 2729, 1738, 1708, 1612, 1292, 1266, 1228, 1203, 1180, 1140, 1107, 1051, 1029, 986 cm-1

HRMS (ESI) m/z: $[M+Na]^+$ Calc'd for $C_{23}H_{37}NNa$ 350.2818; Found 350.2784

7-(*tert*-butyl)-2-(3-methoxyphenyl)-7-azabicyclo[2.2.1]heptane (10pa)

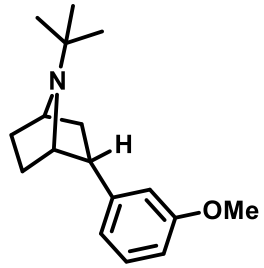

Azanorbornane was prepared according to the general procedure using 1-(*tert*-butyl)pyrrolidine 1-oxide (57 mg, 0.4 mmol, 1.0 equiv.), 3-methoxystyrene (0.028 mL, 0.2 mmol, 0.5 equiv.), 1.8M LDA (0.67 mL, 1.2 mmol, 3.0 equiv.), dry THF (4.0 mL, 0.1M). Aqueous work up was performed followed by purification by FCC on silica gel (15 % EtOAc in Hexanes) to isolate product as a light yellow oil (82% yield, 43 mg, 0.164 mmol). (Rf = 0.53 1:1 Hex/EtOAc).

^{1}H NMR (400 MHz, $CDCl_3$) δ 7.22 (t, *J* = 7.9 Hz, 1H), 6.81 (d, *J* = 7.7 Hz, 1H), 6.76 (s, 1H), 6.73 (d, *J* = 8.1 Hz, 1H), 3.81 (s, 3H), 3.71 (t, *J* = 2.1 Hz, 1H), 3.67 (t, *J* = 4.6 Hz, 1H), 3.42 – 3.31 (m, 1H), 2.13 – 2.00 (m, 1H), 1.67 (m, 1H), 1.49 – 1.25 (m, 4H), 1.18 (s, 9H).

^{13}C NMR (126 MHz, $CDCl_3$) δ 159.6, 144.4, 129.1, 120.9, 114.7, 110.6, 61.0, 56.8, 55.3, 51.8, 48.2, 36.1, 31.8, 30.3, 24.7

IR: 2967, 2871, 2834, 1686, 1601, 1253, 1227, 1202, 1159, 1050 cm-1

HRMS (ESI) m/z: $[M+H]^+$ Calc'd for $C_{17}H_{26}NO$ 260.2009; Found 260.1989

7-(adamantan-1-yl)-2-(3-methoxyphenyl)-7-azabicyclo[2.2.1]heptane (10pb)

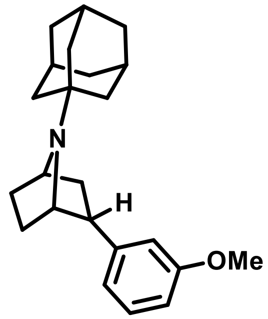

Azanorbornane was prepared according to the general procedure using 1-(adamant-1-yl)pyrrolidine 1-oxide (88 mg, 0.4 mmol, 1.0 equiv.), 3-methoxystyrene (0.028 mL, 0.2 mmol, 0.5 equiv.), 1.8M LDA (0.67 mL, 1.2 mmol, 3.0 equiv.), dry THF (4.0 mL, 0.1M). Aqueous work up was performed followed by purification by FCC on silica gel (10 % EtOAc in Hexanes) to isolate product as a light yellow amorphous solid (86% yield, 58 mg, 0.172 mmol). (Rf = 0.50 1:1 Hex/EtOAc).

^{1}H NMR (400 MHz, $CDCl_3$) δ 7.21 (t, *J* = 7.9 Hz, 1H), 6.81 (d, *J* = 7.7 Hz, 1H), 6.76 (s, 1H), 6.73 (d, *J* = 8.2 Hz, 1H), 3.81 (s, 3H), 3.78 (t, *J* = 4.2 Hz, 1H), 3.74 (t, *J* = 4.6 Hz, 1H), 3.37 – 3.30 (m, 1H), 2.09 – 2.00 (m, 4H), 1.78 (s, 6H), 1.64 (s, 7H), 1.44 (dd, *J* = 12.0, 5.6 Hz, 1H), 1.39 – 1.27 (m, 3H).

^{13}C NMR (101 MHz, $CDCl_3$) δ 159.59, 144.50, 129.12, 120.92, 114.64, 110.59, 59.66, 55.51, 55.30, 52.12, 48.42, 43.27, 36.88, 36.28, 32.24, 29.97, 25.13.

IR: 2978, 2965, 2934, 2898, 2849, 1598, 1251, 1193, 1157, 1130, 1112, 1099, 1062, 1017, 992 cm-1

HRMS (ESI) m/z: $[M+H]^+$ Calc'd for $C_{23}H_{32}NO$ 338.2478; Found 388.2453

2-(3-methoxyphenyl)-7-(2,4,4-trimethylpentan-2-yl)-7-azabicyclo[2.2.1]heptane (10pc)

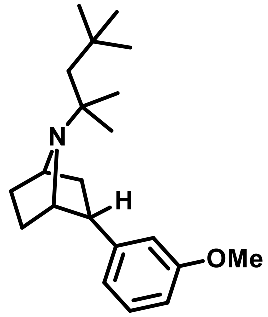

Azanorbornane was prepared according to the general procedure using 1-(2,4,4-trimethylpentan-2-yl)pyrrolidine 1-oxide (166 mg, 0.834 mmol, 1.0 equiv.), 3-methoxystyrene (0.029 mL, 0.208 mmol, 0.25 equiv.), 1.43M LDA (1.76 mL, 2.50 mmol, 3.0 equiv.), dry THF (6 mL, 0.14M). Aqueous work up was performed followed by purification by FCC on silica gel (10 % EtOAc in Hexanes) to isolate product as a colorless oil (80% yield, 53 mg, 0.166 mmol). (Rf = 0.78 1:1 Hex/EtOAc).

^{1}H NMR (400 MHz, $CDCl_3$) δ 7.25 (t, *J* = 7.7 Hz 1H), 6.84 (d, *J* = 7.6 Hz, 1H), 6.80 (s, 1H), 6.76 (dd, *J* = 8.1, 2.3 Hz, 1H), 3.84 (s, 3H), 3.73 (t, *J* = 4.0 Hz, 1H), 3.67 (t, *J* = 4.6 Hz, 1H), 3.38 (dt, *J* = 9.8, 4.7 Hz, 1H), 2.14 – 2.03 (m, 1H), 1.77 – 1.62 (m, 1H), 1.48 (s, 2H), 1.47 – 2.40 (m, 2H), 1.36 – 1.29 (m, 2H) 1.21 (s, 6H), 1.08 (s, 9H).

^{13}C NMR (126 MHz, $CDCl_3$) δ 159.6, 144.6, 129.1, 121.0, 114.7, 110.5, 60.6, 56.4, 55.6, 55.3, 55.0, 48.1, 36.1, 32.1, 32.0, 31.9, 29.6, 29.3, 25.0.

IR: 2952, 2902, 2833, 1600, 1268, 1253, 1229, 1185, 1050, 987 cm-1

HRMS (ESI) m/z: $[M+H]^+$ Calc'd for $C_{21}H_{34}NO$ 316.2635; Found 316.2608

7-(tert-butyl)-2-(((tert-butyldiphenylsilyl)oxy)methyl)-7-azabicyclo[2.2.1]heptane (10qa)

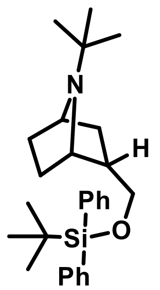

Azanorbornane was prepared according to the general procedure using 1-(*tert*-butyl)pyrrolidine 1-oxide (0.258 mg, 1.80 mmol, 1.0 equiv.), (allyloxy)(tert-butyl)diphenylsilane (0.267 mg, 0.9 mmol, 0.5 equiv.), 1.66M LDA (3.26 mL, 5.40 mmol, 3.0 equiv.), dry THF (8.45 mL, 0.2M). The reaction stirred at RT for 16h until it was quenched with DI water (5 mL). The aqueous layer was extracted 2x with diethyl ether, and the organic layers were combined, dried with Na_2SO_4, and concentrated *in vacuo*. The crude reaction mixture was purified by FCC on silica gel (15% EtOAc in hexanes) to isolate product as a yellow oil (75% yield, 285 mg, 0.414 mmol). (Rf = 0.21 1:1 Hex/EtOAc).

1H NMR (400 MHz, CDCl3) δ 7.68 – 7.65 (m, 4H), 7.43 – 7.34 (m, 6H), 3.69 (dd, *J* = 10.4, 6.4 Hz, 1H), 3.65 (t, *J* = 4.4 Hz, 1H), 3.52 – 3.44 (m, 2H), 2.29 (m, 1H), 1.75 – 1.66 (m, 1H), 1.64 – 1.54 (m, 3H), 1.53 – 1.42 (m, 1H), 1.13 (s, 9H), 1.04 (s, 9H), 0.53 (dd, *J* = 11.6, 5.3 Hz, 1H).

13C NMR (126 MHz, CDCl3) δ 135.7, 135.6, 134.3, 134.2, 129.7, 129.6, 127.7, 127.6, 66.0, 57.8, 56.0, 51.6, 44.3, 35.4, 31.4, 30.3, 27.0, 24.1, 19.4.

IR: 2958, 2858, 1427, 1227, 1111, 1059, 1008, 822, 738, 701, 609, 505, 407 cm-1

HRMS (ESI) m/z: $[M+Na]^+$ Calc'd for $C_{27}H_{39}NOSiNa$ 444.2693; Found 444.2685

7-(*tert*-butyl)-2-(4-nitrophenyl)-7-azabicyclo[2.2.1]heptane (S1)

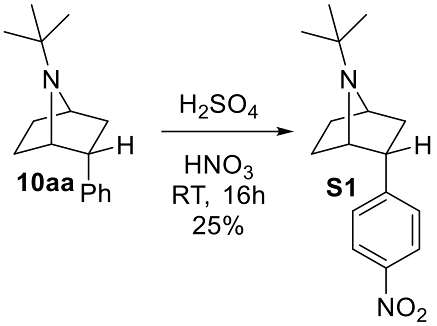

To a RBF, **10aa** was added (423 mg, 1.82 mmol, 1.0 equiv.) followed by 14M HNO_3 (1.3 mL, 18.2 mmol, 10 equiv.). 18M H_2SO_4 (0.1 mL, 1.82 mmol, 1.0 equiv.) was added dropwise and the solution stirred at RT for 16h. After incubation, was basified to pH 7 with excess $NaHCO_3$ and was extracted with ethyl ether (x2). The organic layer was dried with Na_2SO_4 and concentrated *in vacuo*. The crude mixture was purified by silica gel FCC (in 50% EtOAc in hexanes) to elute product as a yellow oil (25% yield, 126 mg, 0.459 mmol). The minor product could not be completely separated but was less than 5% (Major product Rf = 0.40 1:1 Hexanes/EtOAc).

^{1}H NMR (400 MHz, $CDCl_3$) δ 8.15 (d, *J* = 8.9 Hz, 2H), 7.35 (d, *J* = 8.9 Hz, 2H), 3.78 (t, *J* = 4.4 Hz, 1H), 3.72 (t, *J* = 4.8 Hz, 1H), 3.50 – 3.42 (m, 1H), 2.19 – 2.09 (m, 1H), 1.74 – 1.66 (m, 1H), 1.48 (dd, *J* = 12.0, 5.4 Hz, 1H), 1.40 (ddd, *J* = 7.8, 6.4, 3.1 Hz, 1H), 1.35 – 1.20 (m, 2H), 1.18 (s, 9H).

^{13}C NMR (126 MHz, $CDCl_3$) δ 150.9, 146.2 minor, 134.8, 129.2, 123.5, 60.8, 56.8, 51.9, 48.5, 36.4, 31.7, 30.2, 24.8.

IR: 2963, 2874, 1293, 1271, 1254, 1231, 1175, 1157, 1110, 1068, 1057, 1012, 988, 942 cm-1

HRMS (ESI) m/z: $[M+Na]^+$ Calc'd for $C_{16}H_{22}N_2O_2Na$ 297.1574; Found 297.1581

4-7-(adamantan-1-yl)-7-azabicyclo[2.2.1]heptan-2-yl)phenyl trifluoromethanesulfonate (S2)

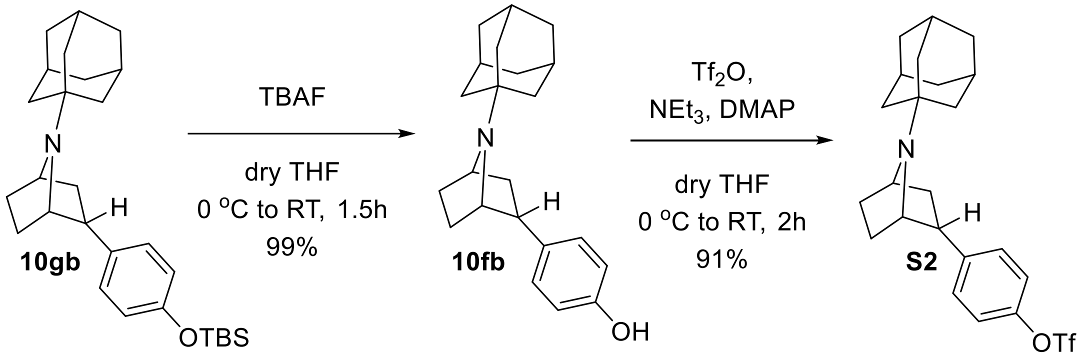

To a dry RBF, **10gb** (65 mg, 0.148 mmol, 1.0 equiv.) was dissolved in dry THF (1.5 mL, 0.1M). The solution was purged with N_2 and cooled to 0 °C. Then 1M TBAF in THF (0.45 mL, 0.445 mmol, 3.0 equiv.) was added and the reaction stirred at RT. After 1.5h, the reaction was quenched with pH 7.4 buffer and extracted with EtOAc (x2). The organic layers were dried with $MgSO_4$ and concentrated *in vacuo*. The crude mixture was purified by FCC on silica (5% MeOH in EtOAc) to afford **10fb** as a white amorphous solid (99% yield, 36 mg, 0.147 mmol)

To a dry test tube, **10fb** (36 mg, 0.147 mmol, 1.0 equiv.) was added along with DMAP (1.8 mg, 0.0147 mmol, 0.1 equiv.), NEt_3 (0.041 mL, 0.309 mmol, 2.1 equiv.) and dry THF (0.5 mL, 0.3M). The solution was purged with N_2 and cooled to 0 °C. Then trifluoromethanesulfonic anhydride [Tf_2O] (49.3 µL, 0.293 mmol, 2.0 equiv.) was added and the reaction was slowly warmed to RT. After 2h the reaction was quenched with DI H_2O and extracted with ethyl ether (x2). The organic layers were dried with $MgSO_4$ and concentrated *in vacuo*. The crude mixture was purified by silica gel FCC (in 50% EtOAc in hexanes) to isolate **S2** as a white amorphous solid (91% yield, 61 mg, 0.134 mmol). (Rf = 0.30 1:3 Hexanes/EtOAc).

^1H NMR (400 MHz, $CDCl_3$) δ 7.26 (d, *J* = 5.0 Hz, 2H), 7.18 (d, *J* = 8.4 Hz, 2H), 3.78 (m, 2H), 3.35 (m, 1H), 2.06 (s, 3H), 1.76 (s, 5H), 1.64 (s, 6H), 1.48 – 1.35 (m, 2H), 1.29 (m, 4H).

^{13}C NMR (126 MHz, $CDCl_3$) δ 147.6, 143.5, 130.1, 122.92 (q, *J* = 321.3 Hz), 120.9, 59.4, 55.4, 52.1, 47.9, 43.3, 36.8, 36.7, 32.2, 29.9, 25.0.

^{19}F NMR (376 MHz, $CDCl_3$) δ -72.85.

IR: 2902, 2849, 1500, 1450, 1422, 1356, 1309, 1267, 1249, 1208, 1137, 1100, 1015, 976, 888, 834, 816, 778, 755, 726, 639, 608, 571, 522, 410 cm-1

HRMS (ESI) m/z: $[M+H]^+$ Calc'd for $C_{23}H_{29}F_3NO_3S$ 456.1815; Found 456.1801

2-(4-(tert-butyl)phenyl)-7-azabicyclo[2.2.1]heptane (S3)[8]

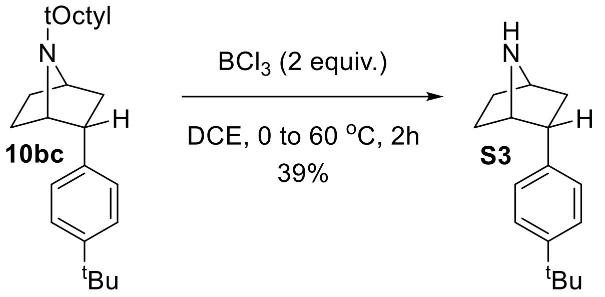

A three neck RBF was sealed with two septa and an air condenser connected to the nitrogen. This setup was then purged with N_2 gas for three cycles of 5 mins under vacuum. DCE (1 mL, 1M) (stored under nitrogen) was used to dissolve **10bc** (29 mg, 0.10 mmol, 1.0 equiv.). Then this solution was added to the RBF using air-free transfer. The solution was then allowed to stir and cool to 0 °C. Then 1M BCl_3 in hexanes (0.2 mL, 0.2 mmol, 2.0 equiv.) was added to the stirring cold solution using a needle and syringe (added dropwise over a period of 5 mins). The ice bath was then removed, and the solution was heated in air until RT. Solution was then gently heated over a period of 1 hour to reach 60 °C. Solution then remained at 60 °C and allowed to stir for 1 hour. Solution was then allowed to cool to RT and then their afterward back to 0 °C. When the solution was at 0 °C, DI water (10 mL) was added dropwise to quench the BCl_3. Solution was then removed from N_2 atmosphere and 5 mL of DCM was added to form a distinct separation of layers. The extraction was done with 3 x 5 mL DCM. The DCM layers were then concentrated *in vacuo*. The crude reaction mixture was purified by FCC (1:1 Hex:EtOAc to 9:1 EtOAc:MeOH). The product eluted as an off white amorphous solid (9 mg, 0.039 mmol, 39% yield).

1H NMR (400 MHz, CDCl3) δ 7.33 (d, *J* = 8.2 Hz, 2H), 7.15 (d, *J* = 8.1 Hz, 2H), 3.73 (dd, *J* = 8.6, 4.3 Hz, 2H), 3.35 – 3.28 (m, 1H), 2.08 – 2.00 (m, 1H), 1.80 (s, 1H), 1.66 – 1.59 (m, 1H), 1.56 (dd, *J* = 12.4, 5.8 Hz, 1H), 1.53 – 1.47 (m, 1H), 1.47 – 1.39 (m, 1H), 1.36 (dd, *J* = 11.7, 2.7 Hz, 1H), 1.32 (s, 9H).

^{13}C NMR (126 MHz, $CDCl_3$) δ 148.8, 138.4, 127.8, 125.1, 61.6, 57.7, 47.7, 35.1, 34.4, 31.4, 31.2, 24.3.

IR: 3219, 2957, 2867, 1650, 1513, 1361, 1268, 1054, 824, 548, 408 cm-1

HRMS (ESI) m/z: $[M+H_3O]^+$ Calc'd for $C_{16}H_{26}NO$ 248.2009; Found 248.2016

2.1 Experimental data

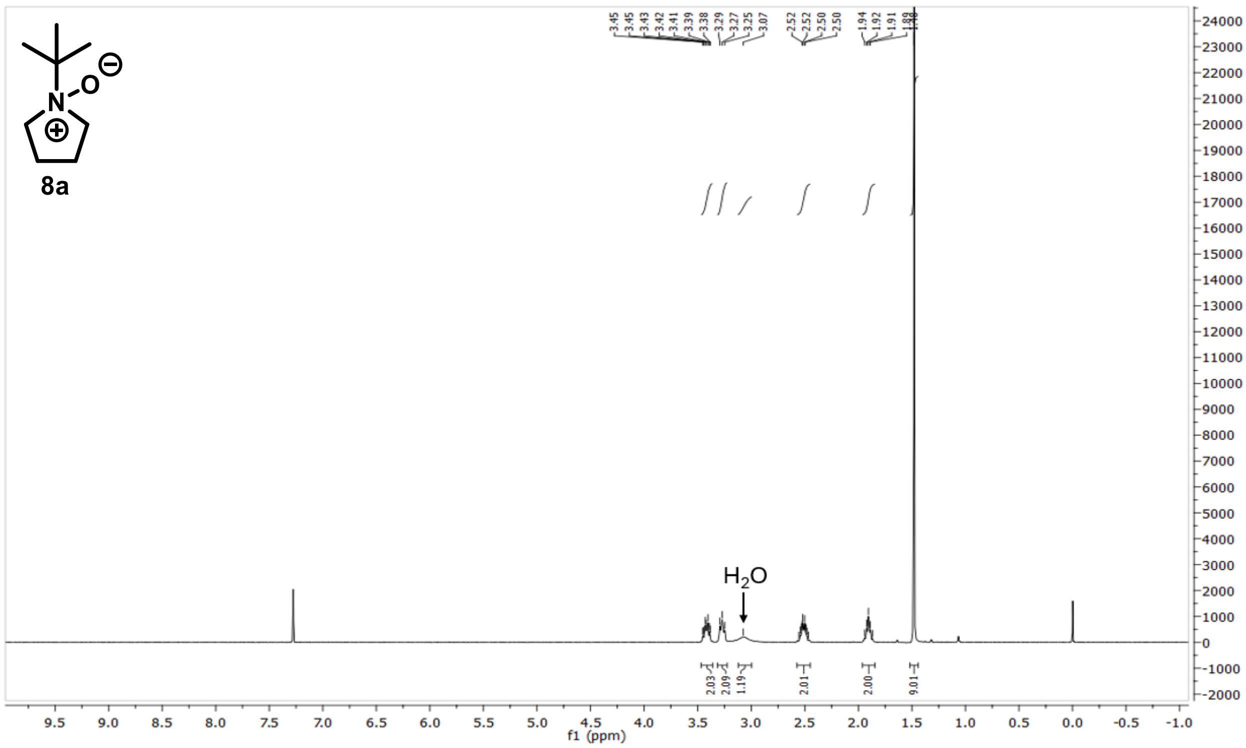

Figure S1: 8a ^{1}H NMR, 400 MHz, $CDCl_3$

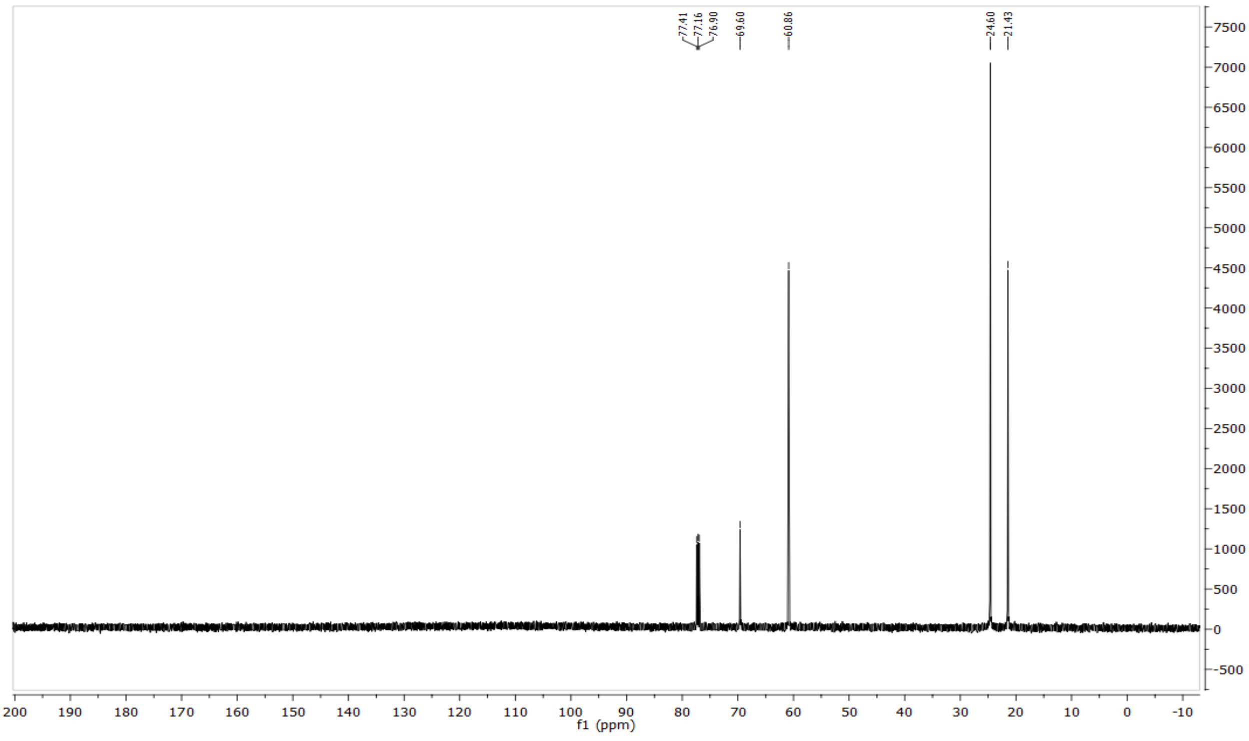

Figure S2: 8a ^{13}C NMR, 126 MHz, $CDCl_3$

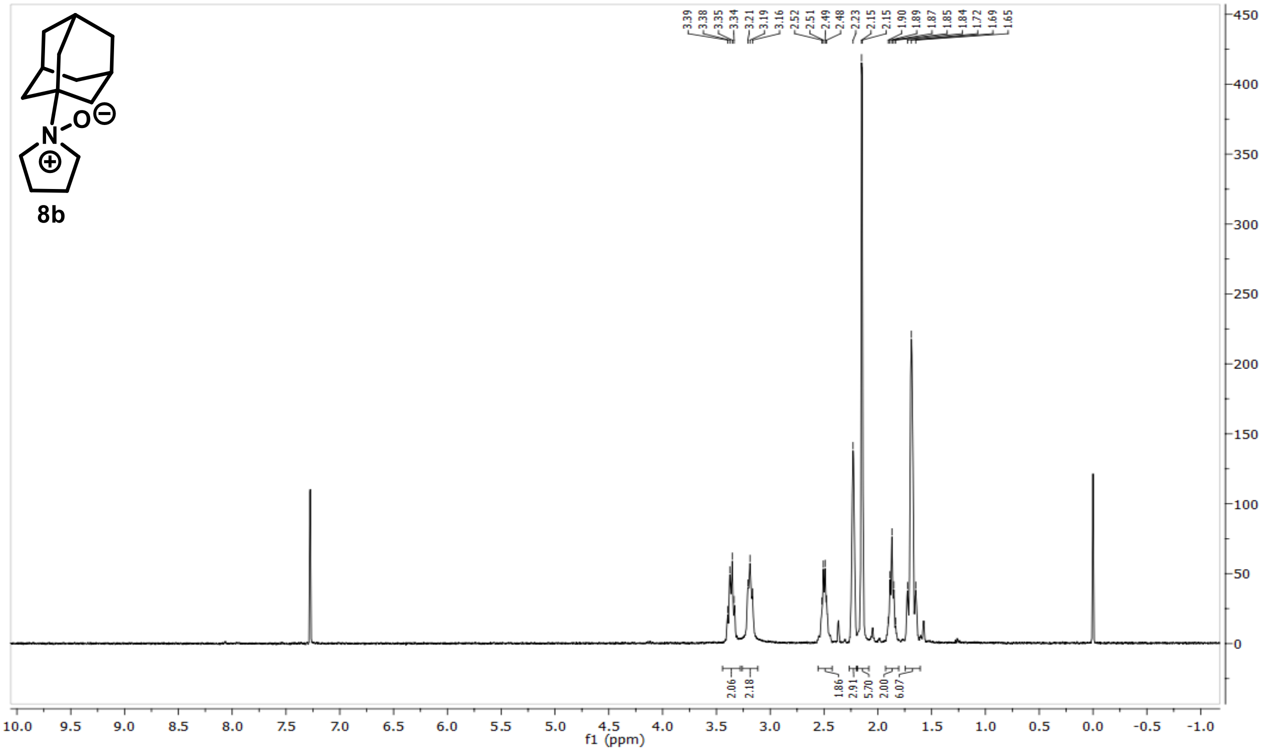

Figure S3: 8b ¹H NMR, 400 MHz, $CDCl_3$

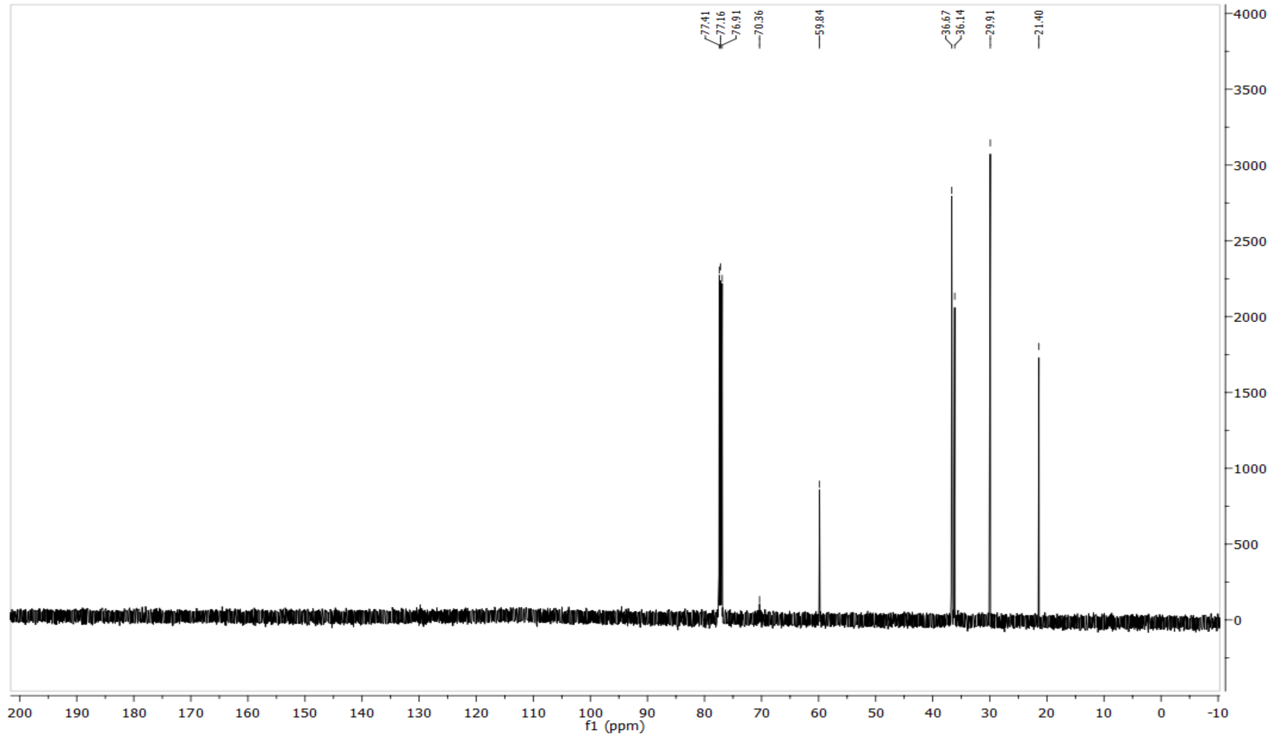

Figure S4: 8b ¹³C NMR, 126 MHz, $CDCl_3$

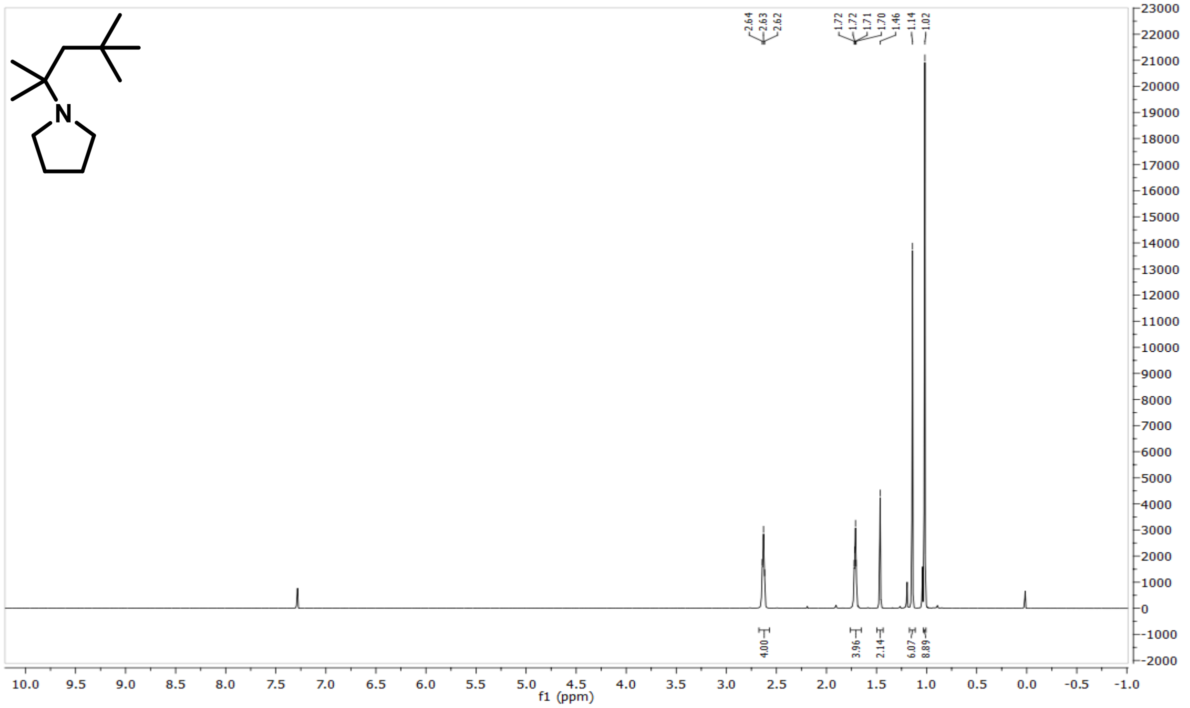

Figure S5: 1-(2,4,4-trimethylpentan-2-yl)pyrrolidine ^{1}H NMR, 500 MHz, $CDCl_3$

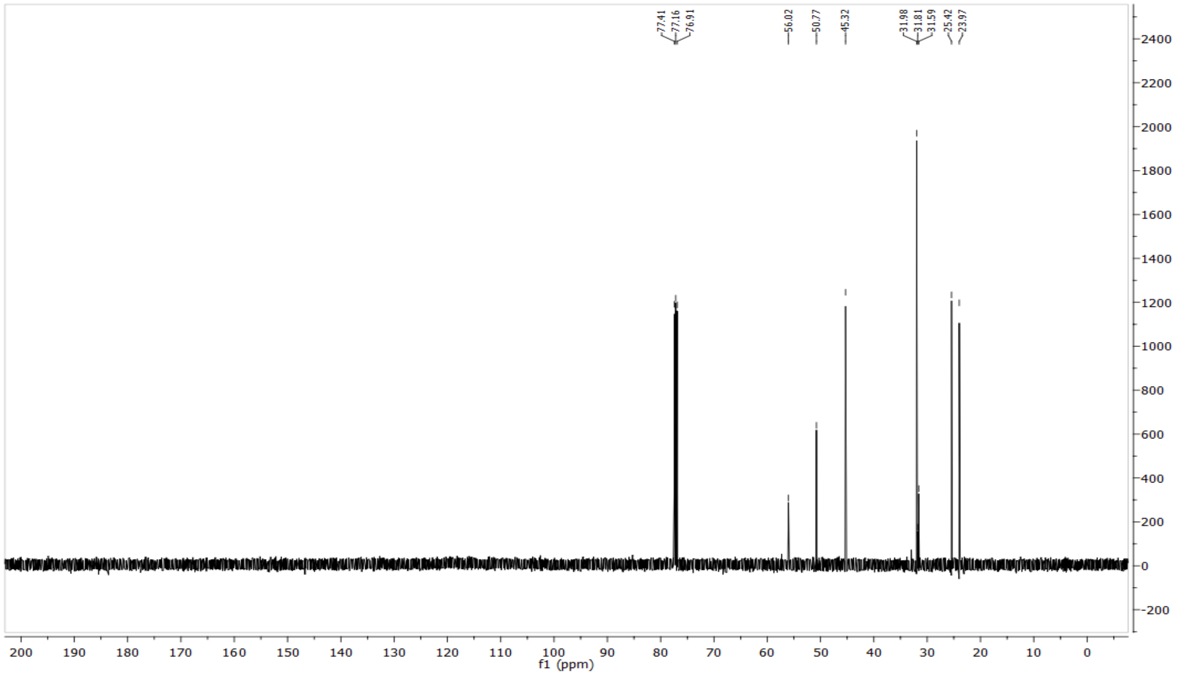

Figure S6: 1-(2,4,4-trimethylpentan-2-yl)pyrrolidine ^{13}C NMR, 126 MHz, $CDCl_3$

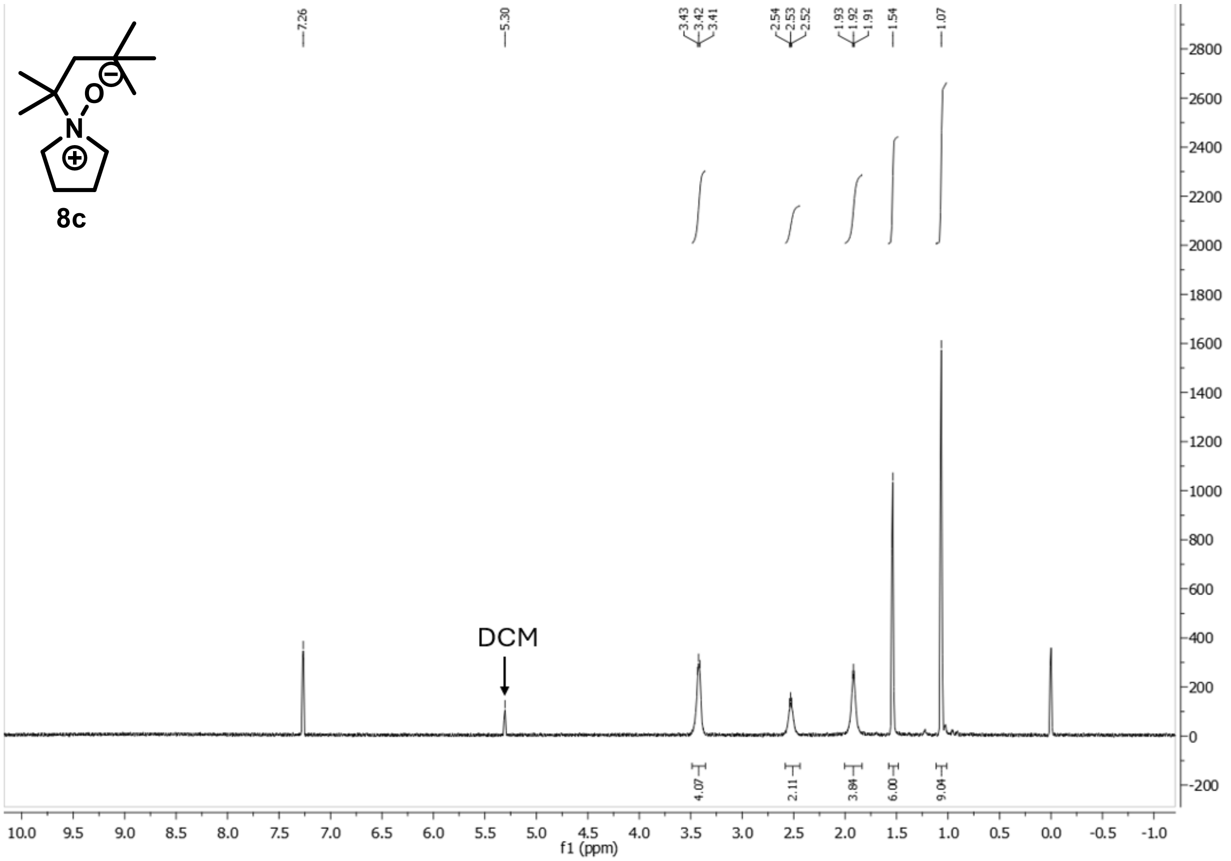

Figure S7: 8c ^{1}H NMR, 500 MHz, $CDCl_3$

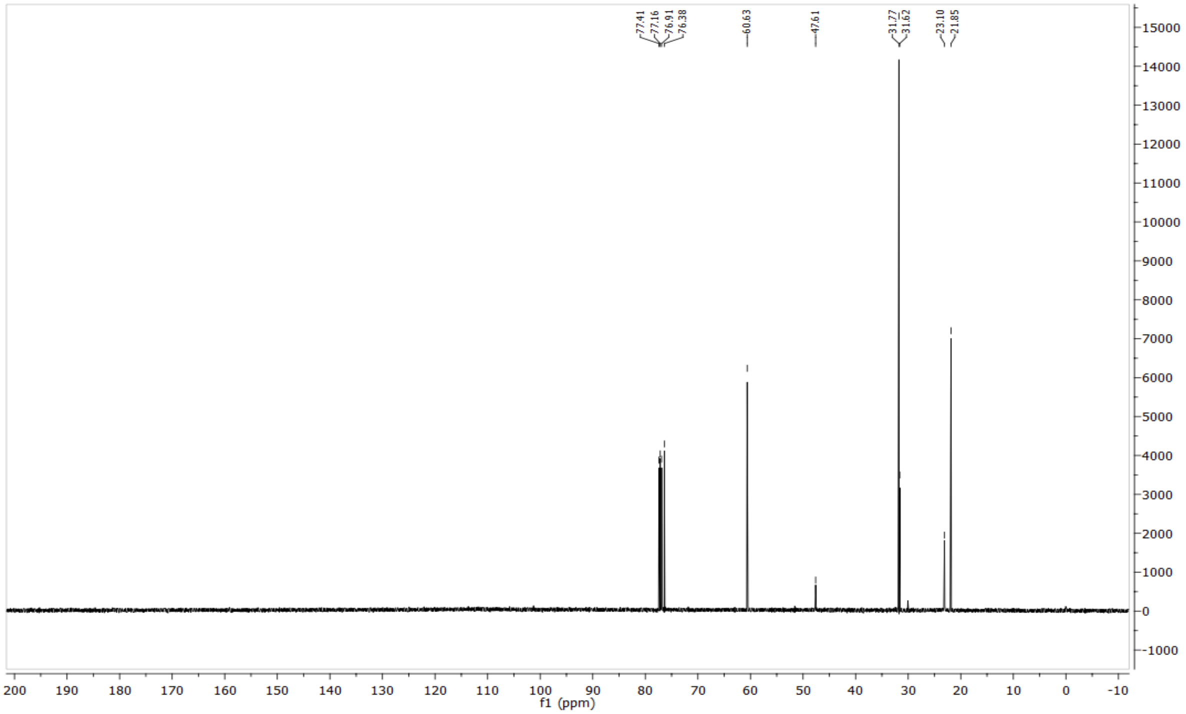

Figure S8: 8c ^{13}C NMR, 126 MHz, $CDCl_3$

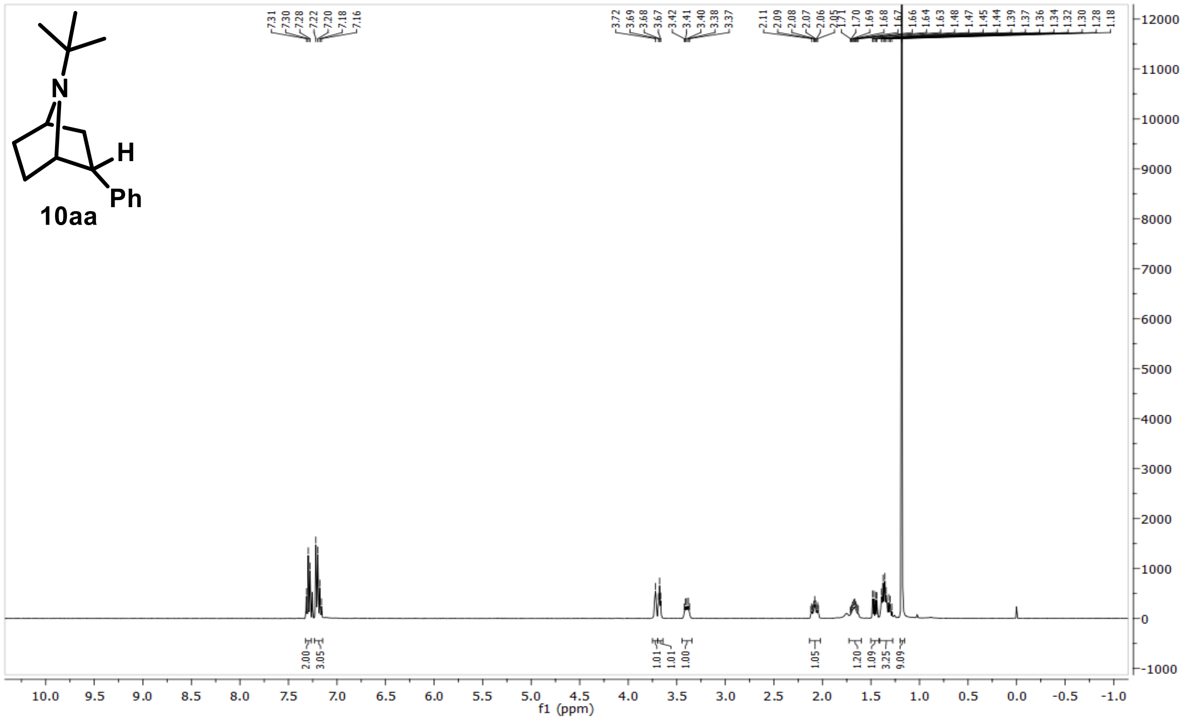

Figure S9: 10aa ^{1}H NMR, 400 MHz, $CDCl_3$

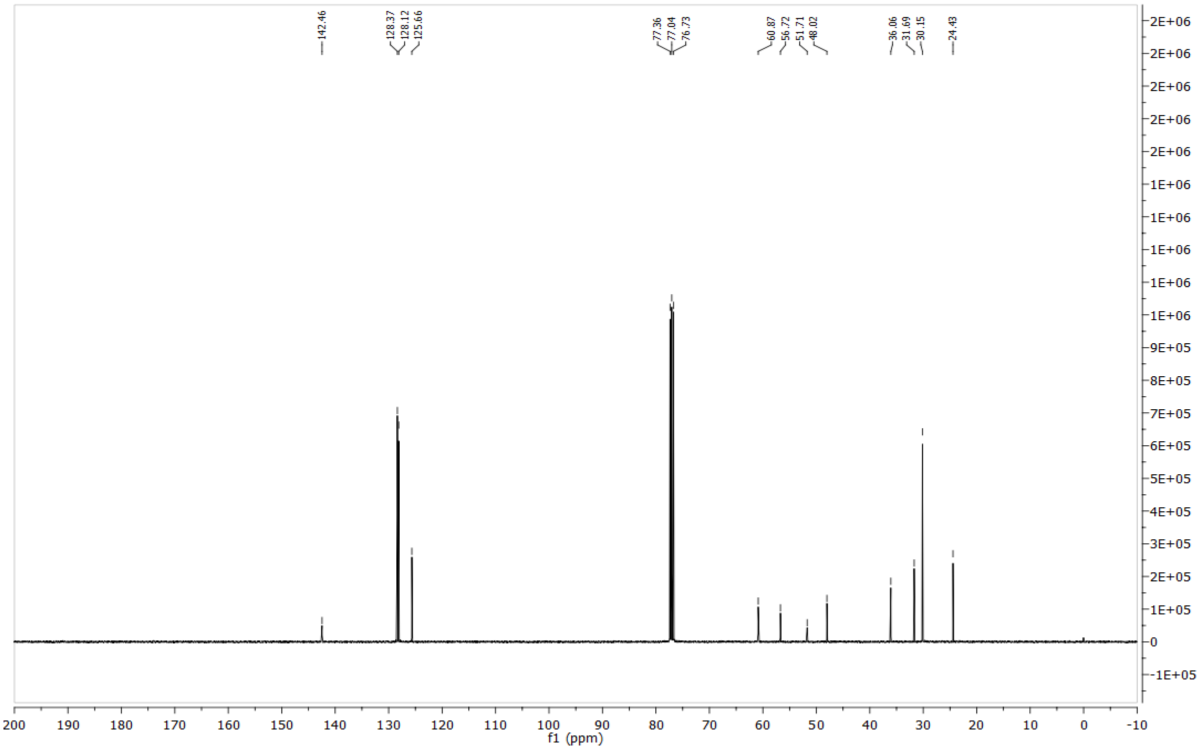

Figure S10: 10aa ^{13}C NMR, 126 MHz, $CDCl_3$

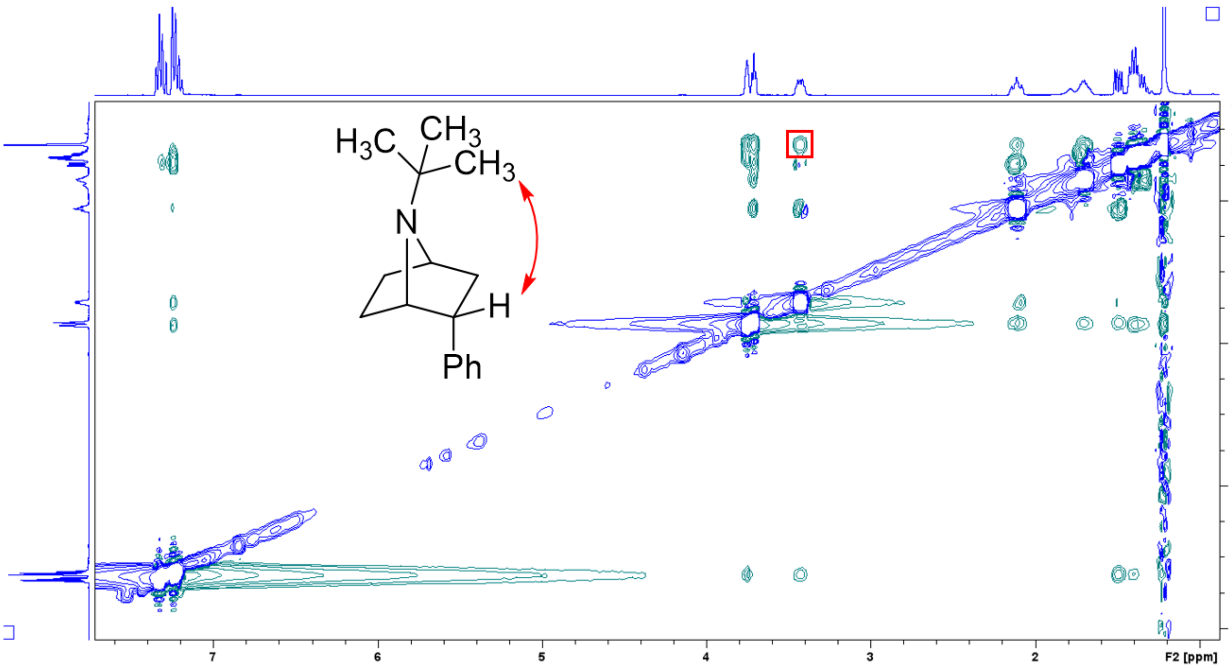

Figure S11: 10aa NOESY, 400 MHz, CDCl$_3$

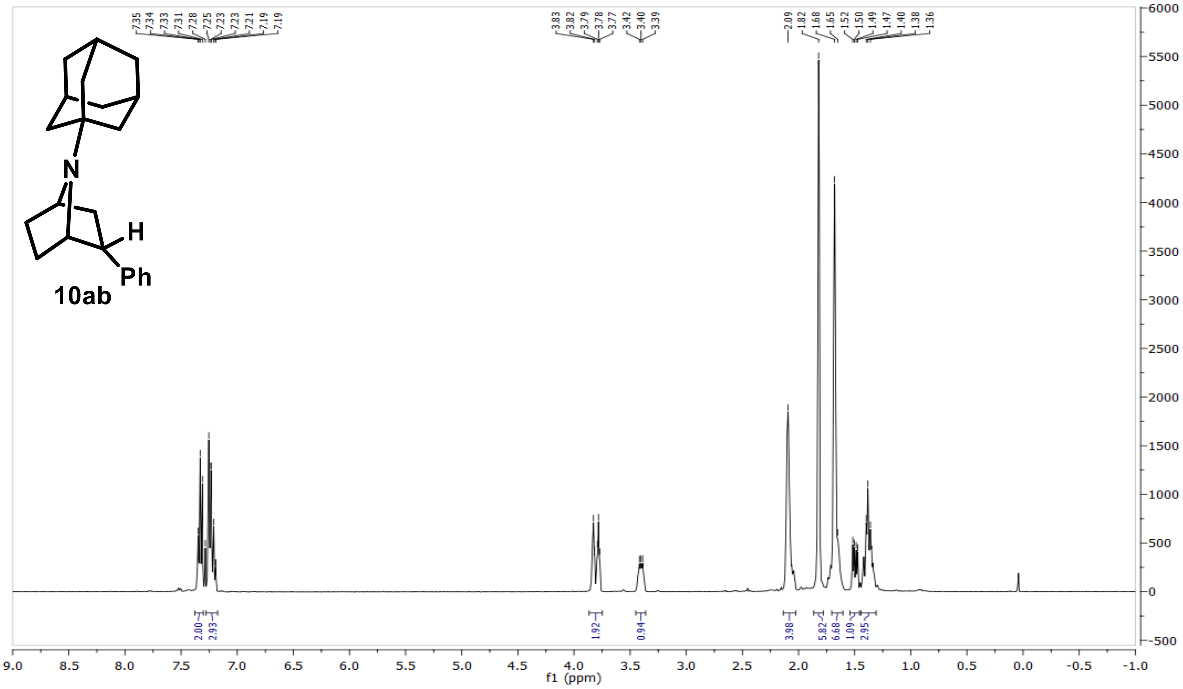

Figure S12: 10ab ^{1}H NMR, 400 MHz, $CDCl_3$

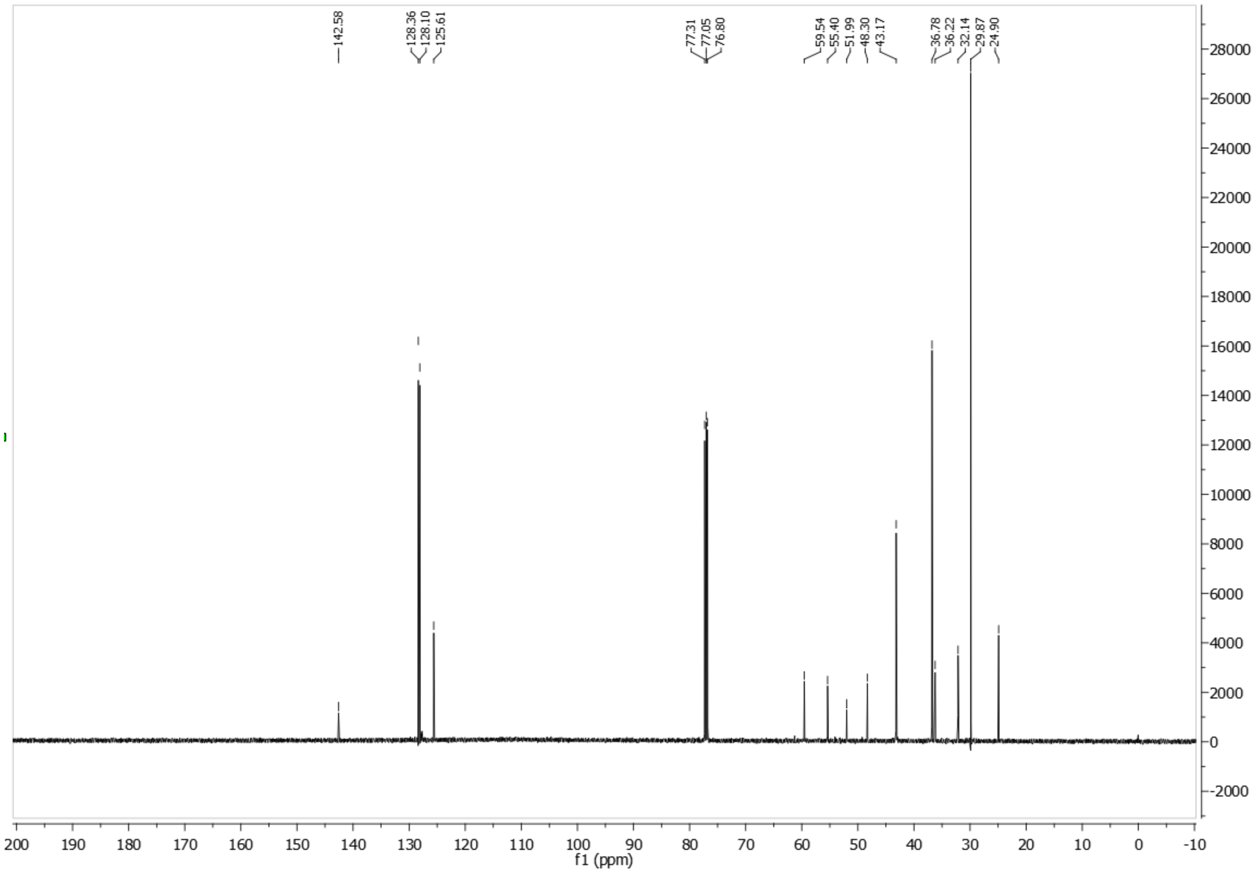

Figure S13: 10ab ^{13}C NMR, 126 MHz, $CDCl_3$

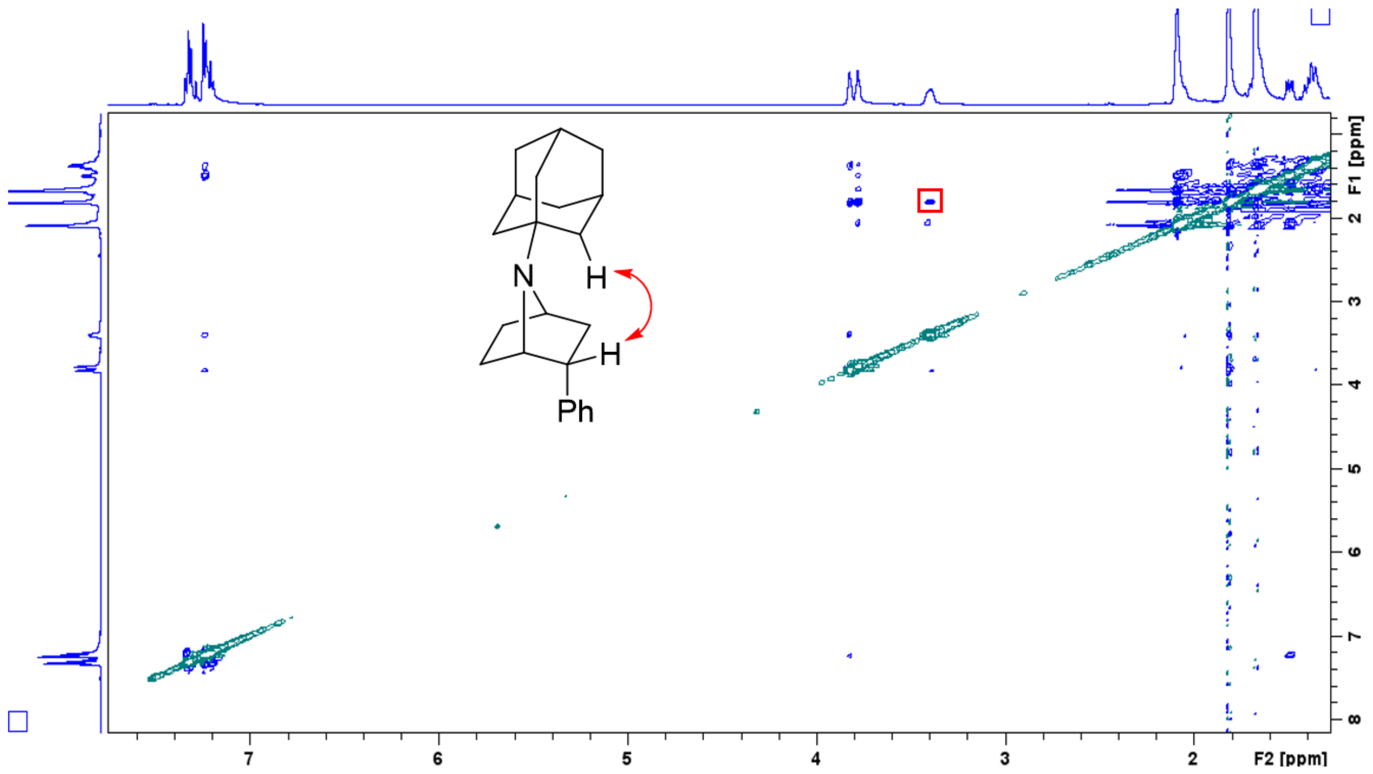

Figure S14: 10ab NOESY, 400 MHz, $CDCl_3$

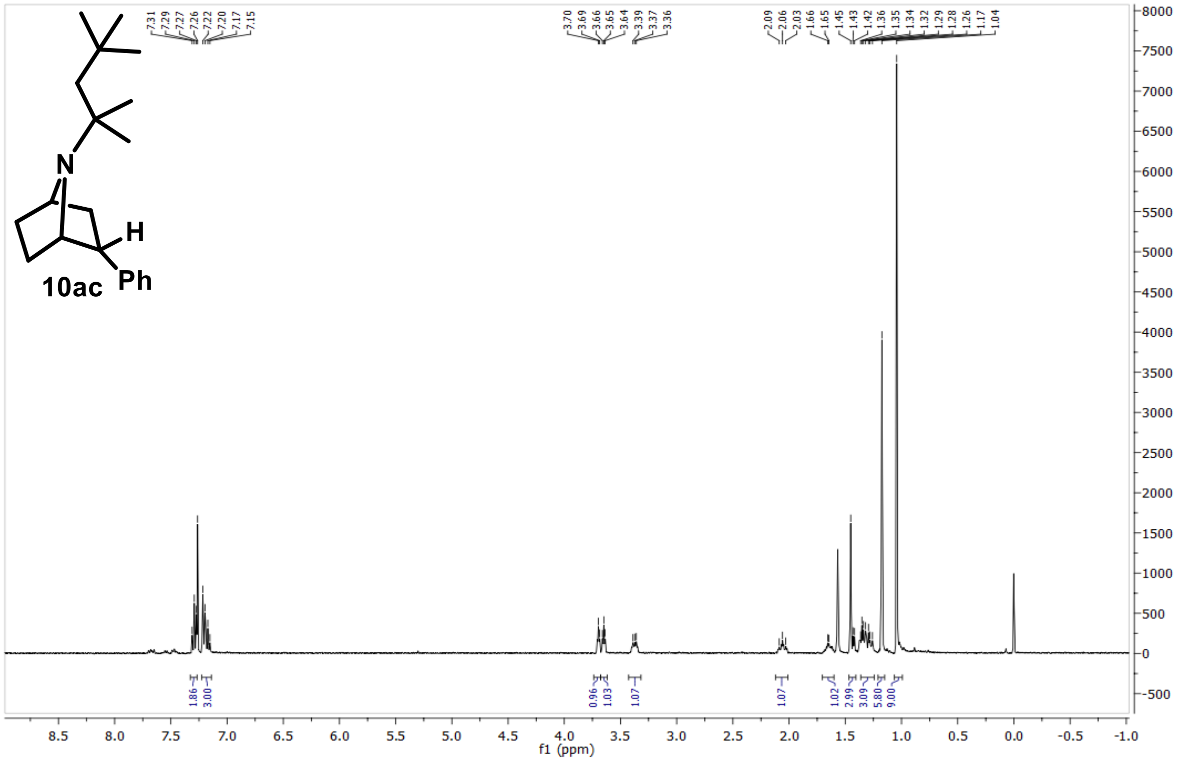

Figure S15: 10ac ^{1}H NMR, 400 MHz, $CDCl_3$

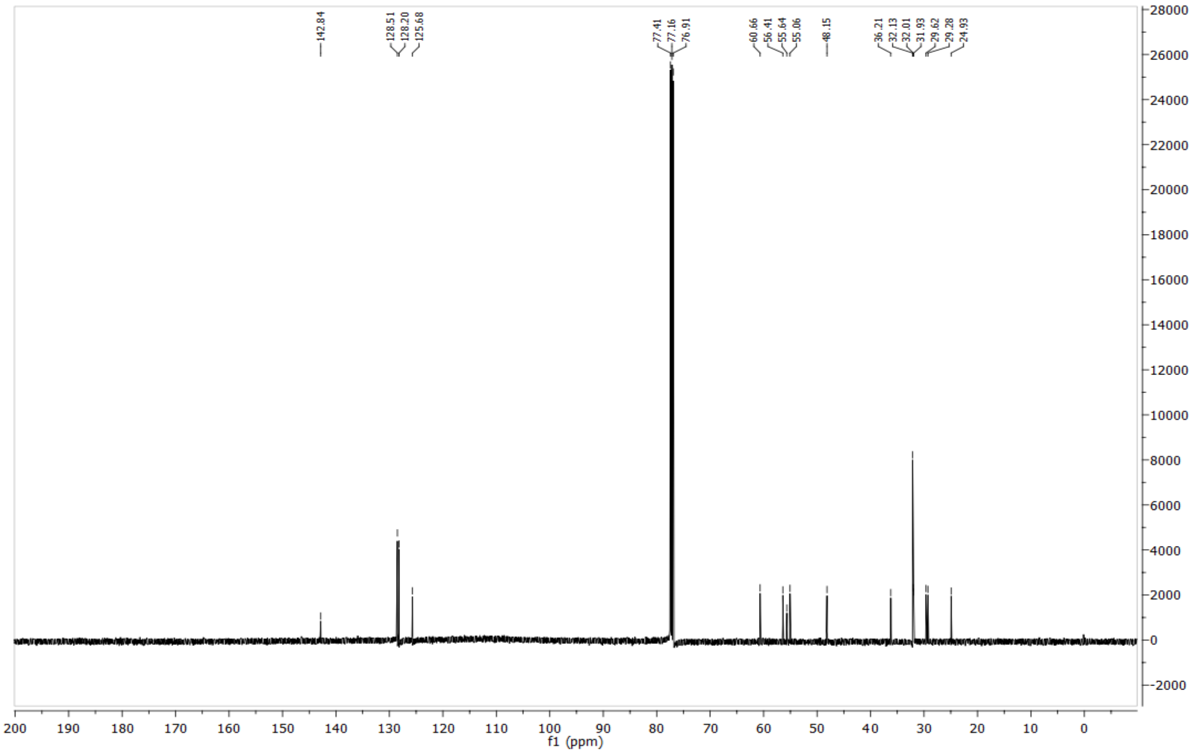

Figure S16: 10ac ^{13}C NMR, 126 MHz, $CDCl_3$

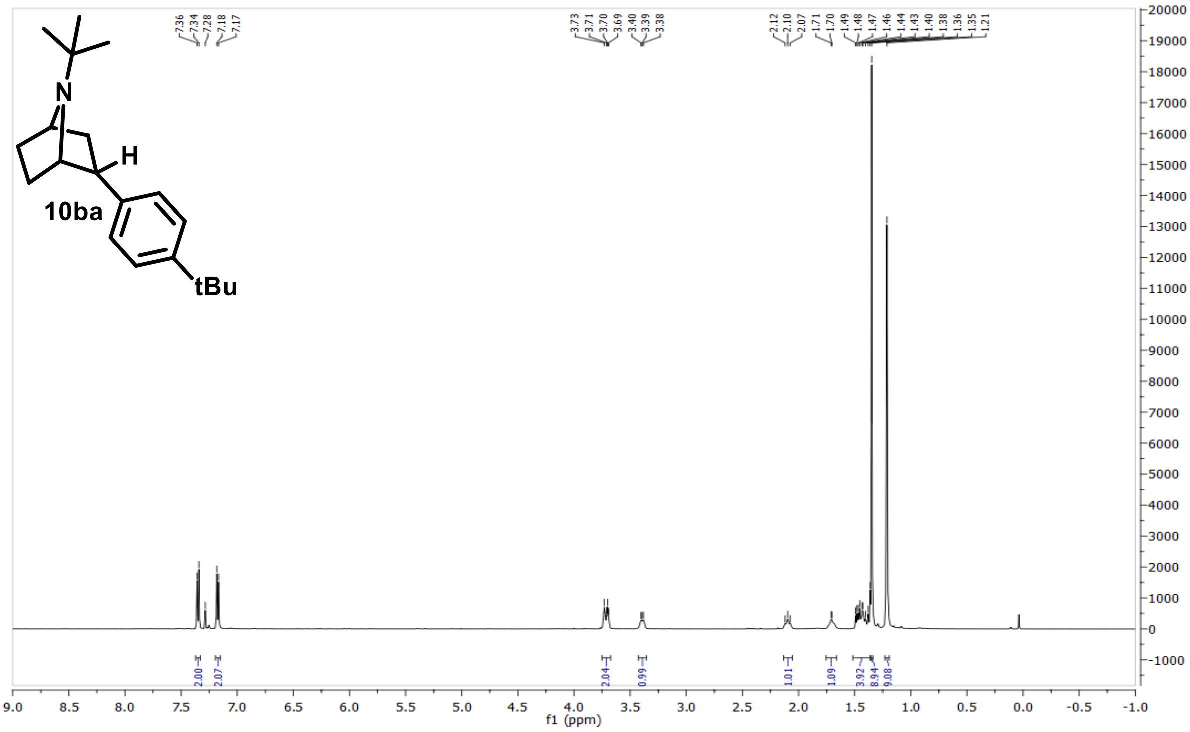

Figure S17: 10ba ^{1}H NMR, 400 MHz, $CDCl_3$

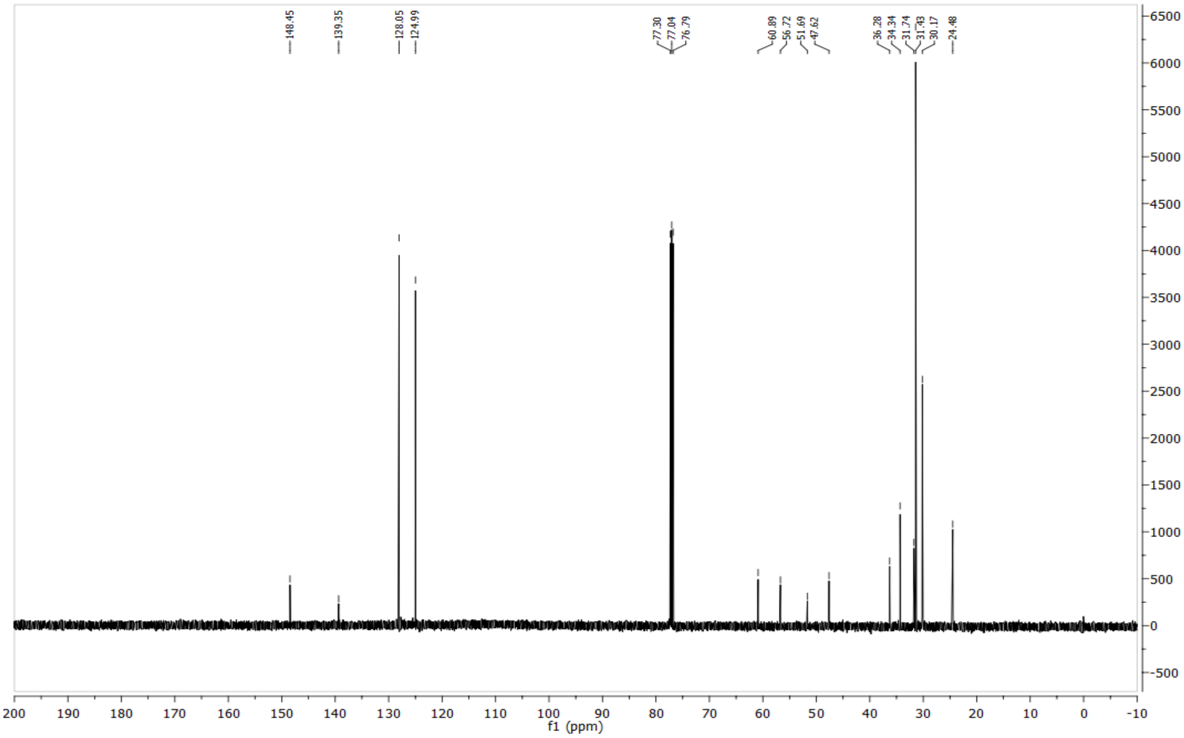

Figure S18: 10ba ^{13}C NMR, 126 MHz, $CDCl_3$

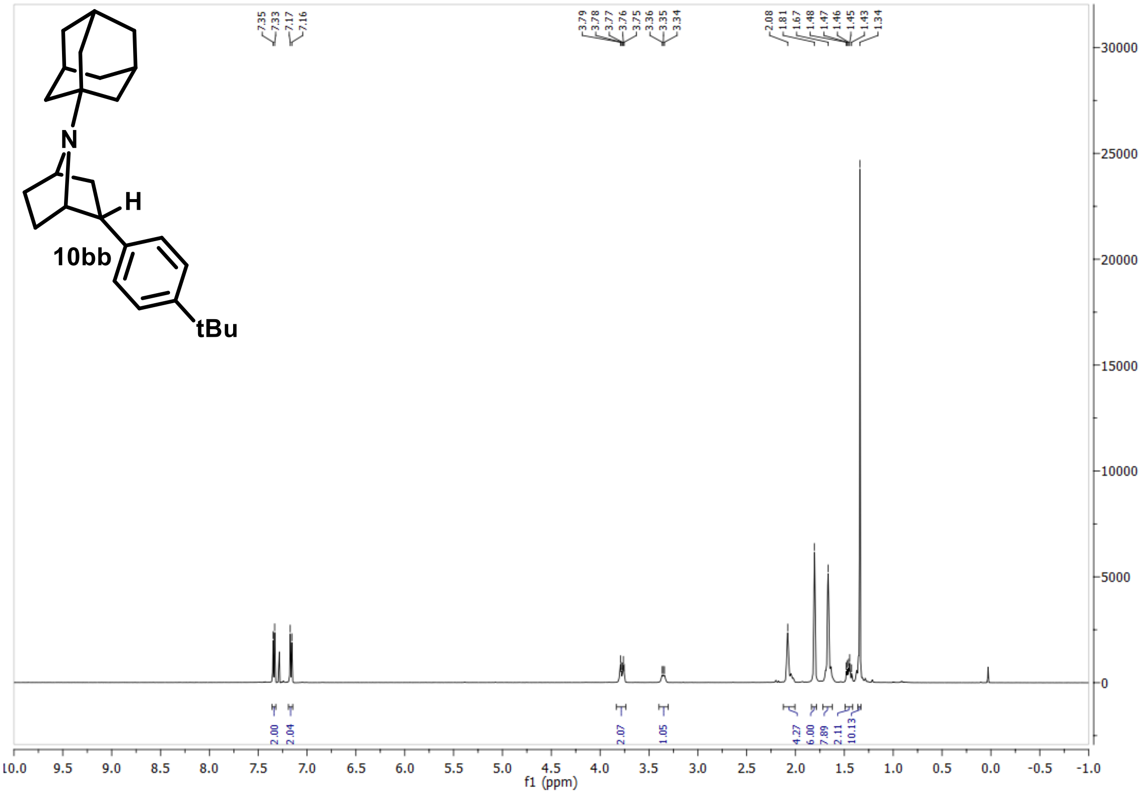

Figure S19: 10bb ^{1}H NMR, 500 MHz, $CDCl_3$

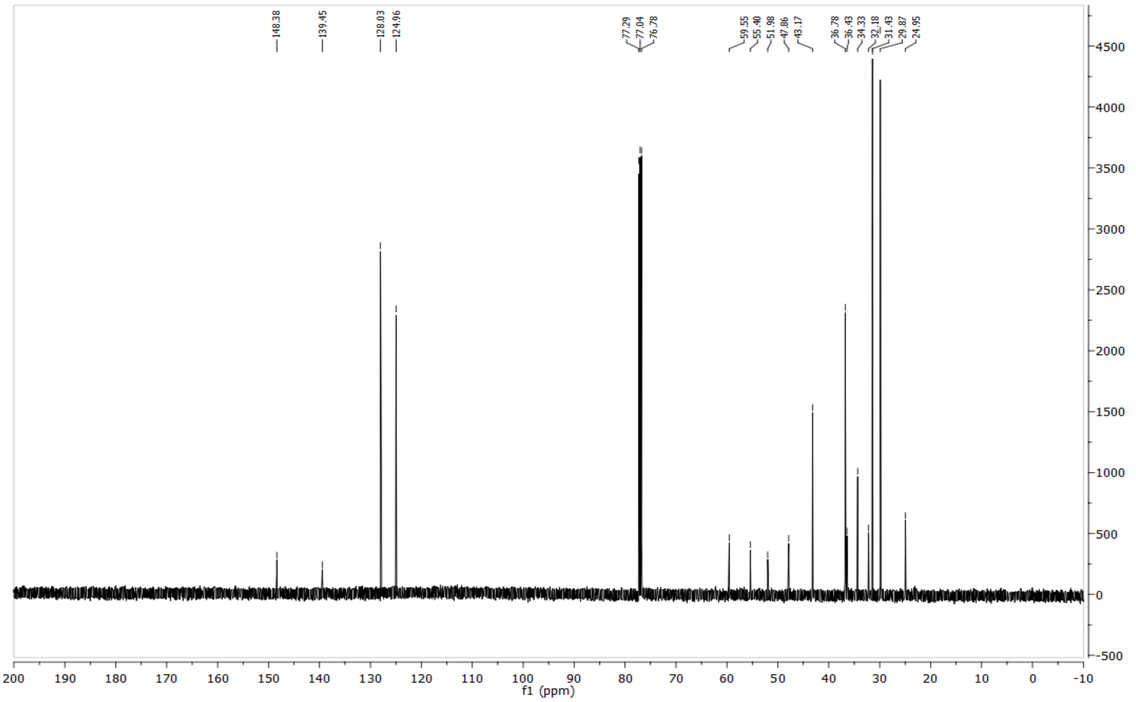

Figure S20: 10bb ^{13}C NMR, 126 MHz, $CDCl_3$

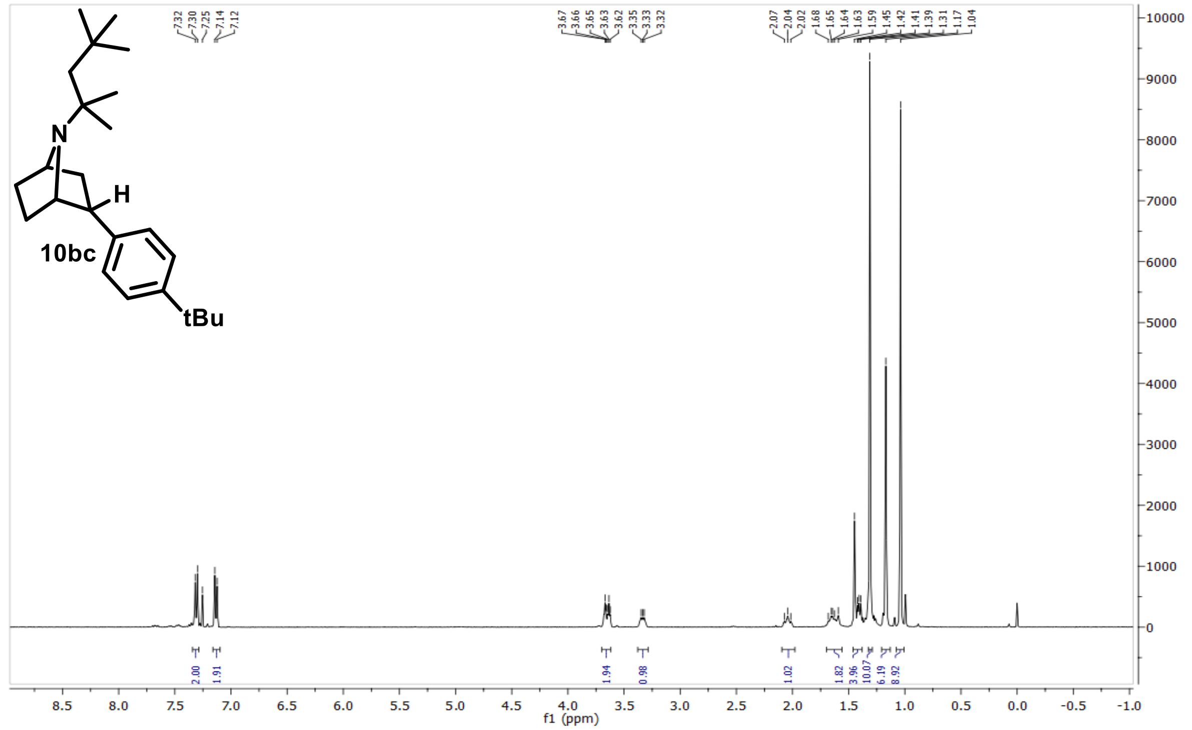

Figure S21: 10bc ^{1}H NMR, 500 MHz, $CDCl_3$

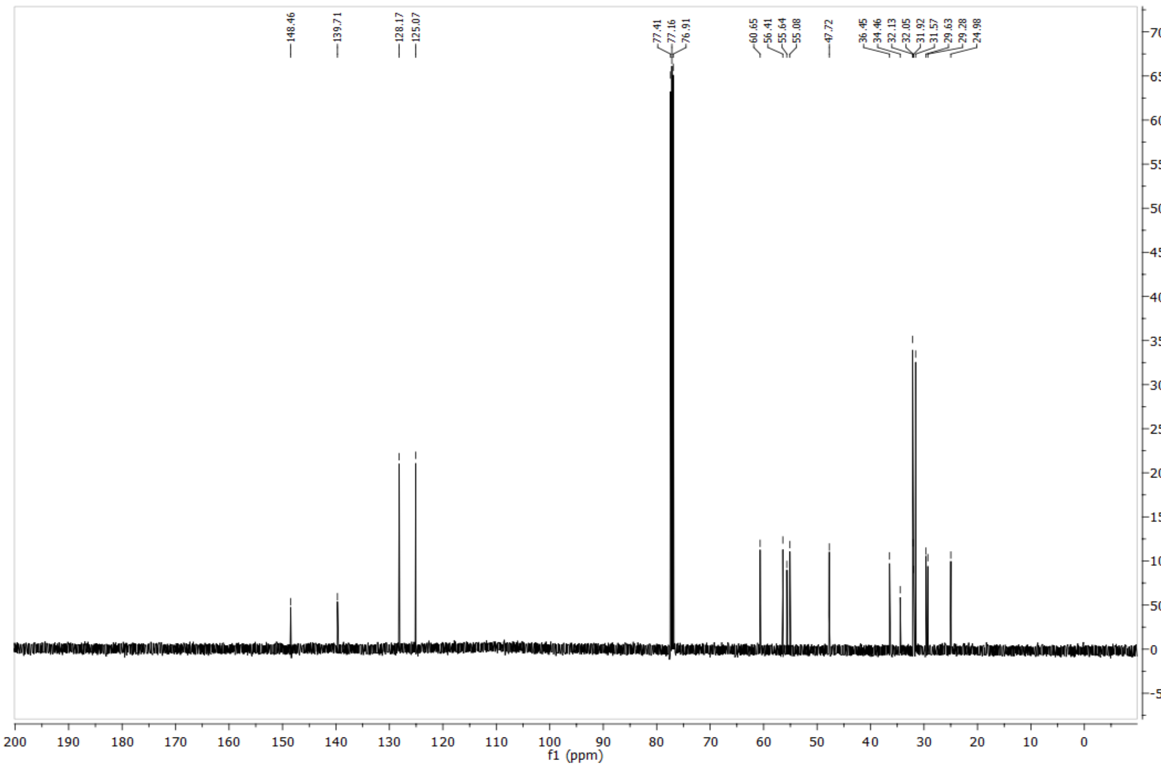

Figure S22: 10bc ^{13}C NMR, 126 MHz, $CDCl_3$

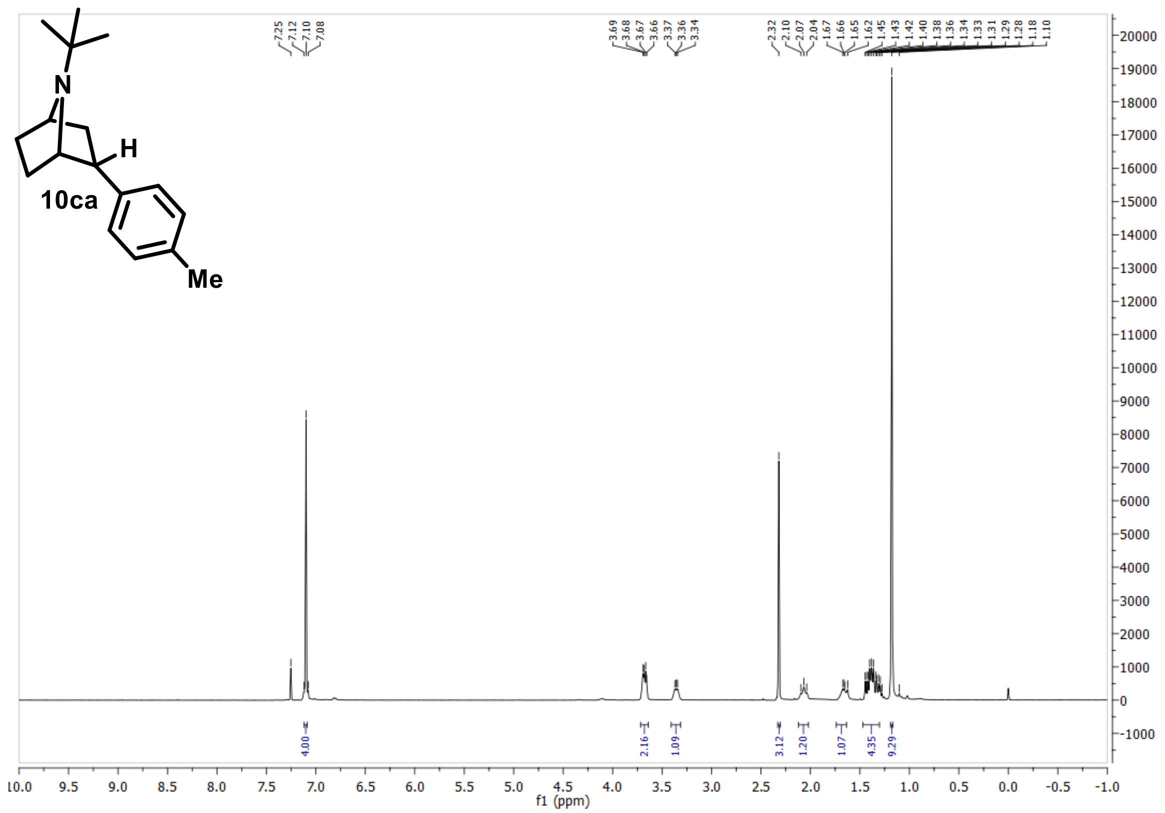

Figure S23: 10ca ^{1}H NMR, 400 MHz, $CDCl_3$

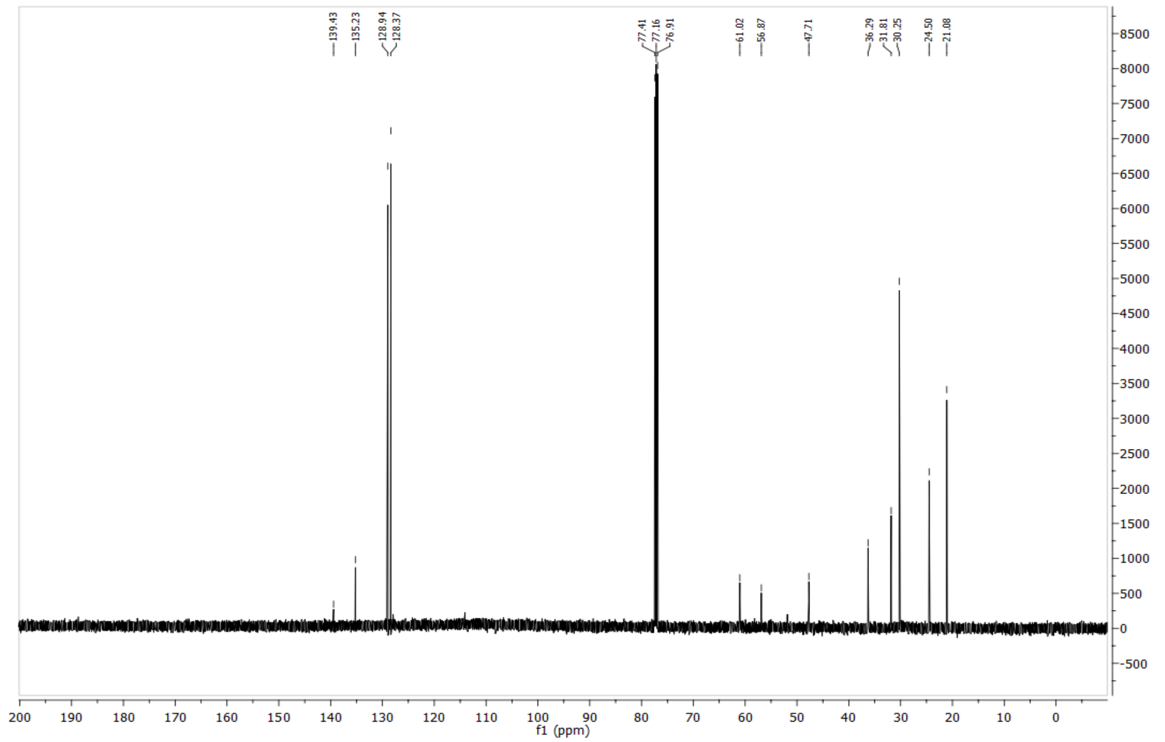

Figure S24: 10ca ^{13}C NMR, 126 MHz, $CDCl_3$

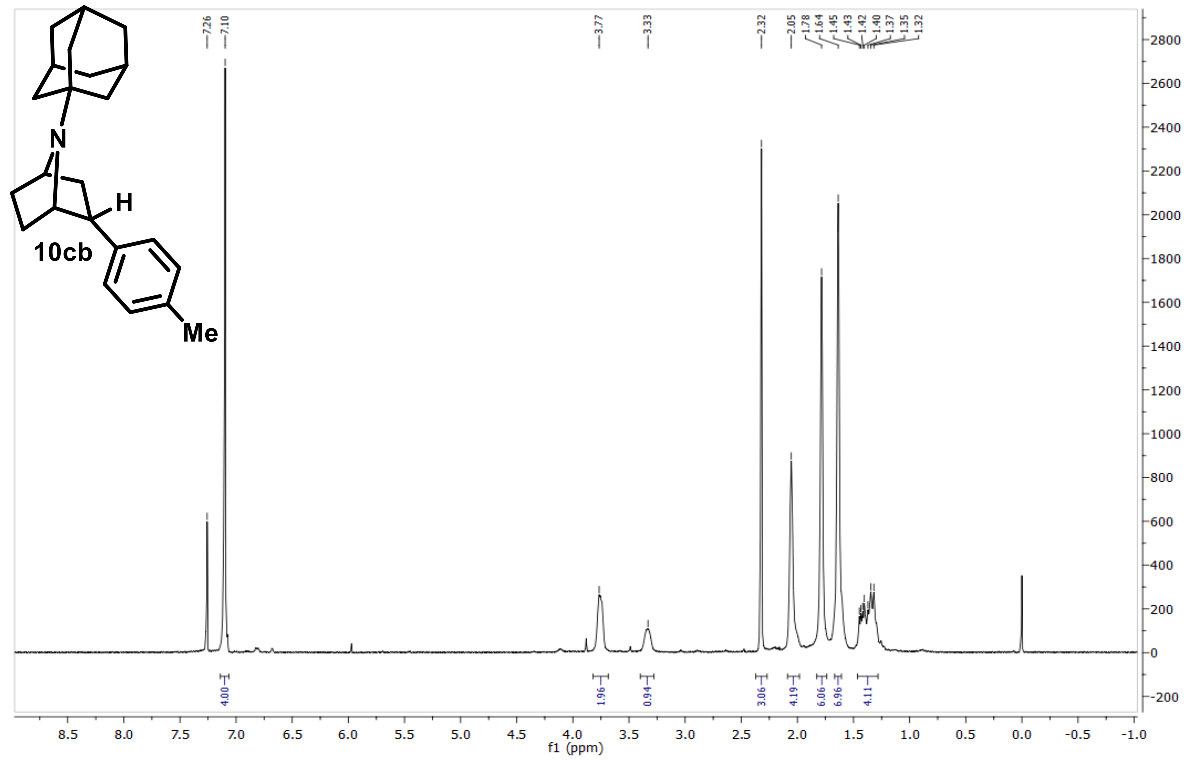

Figure S25: 10cb ^{1}H NMR, 400 MHz, $CDCl_3$

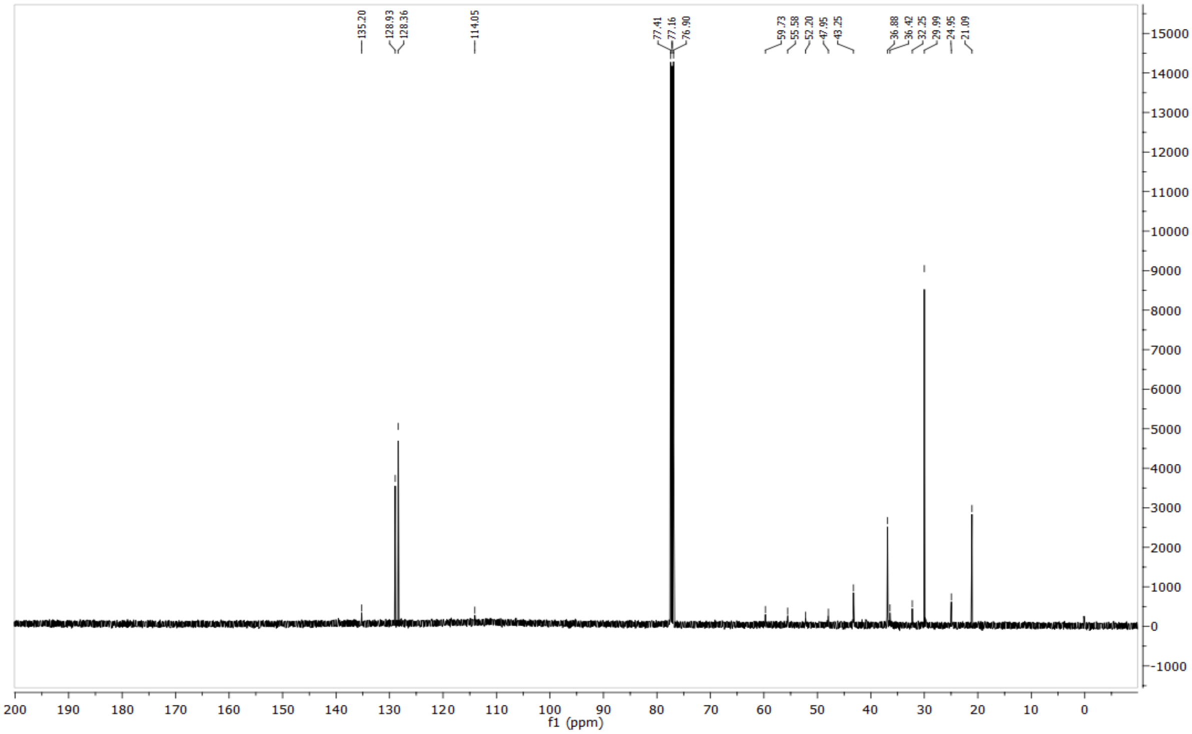

Figure S26: 10cb ^{13}C NMR, 126 MHz, $CDCl_3$

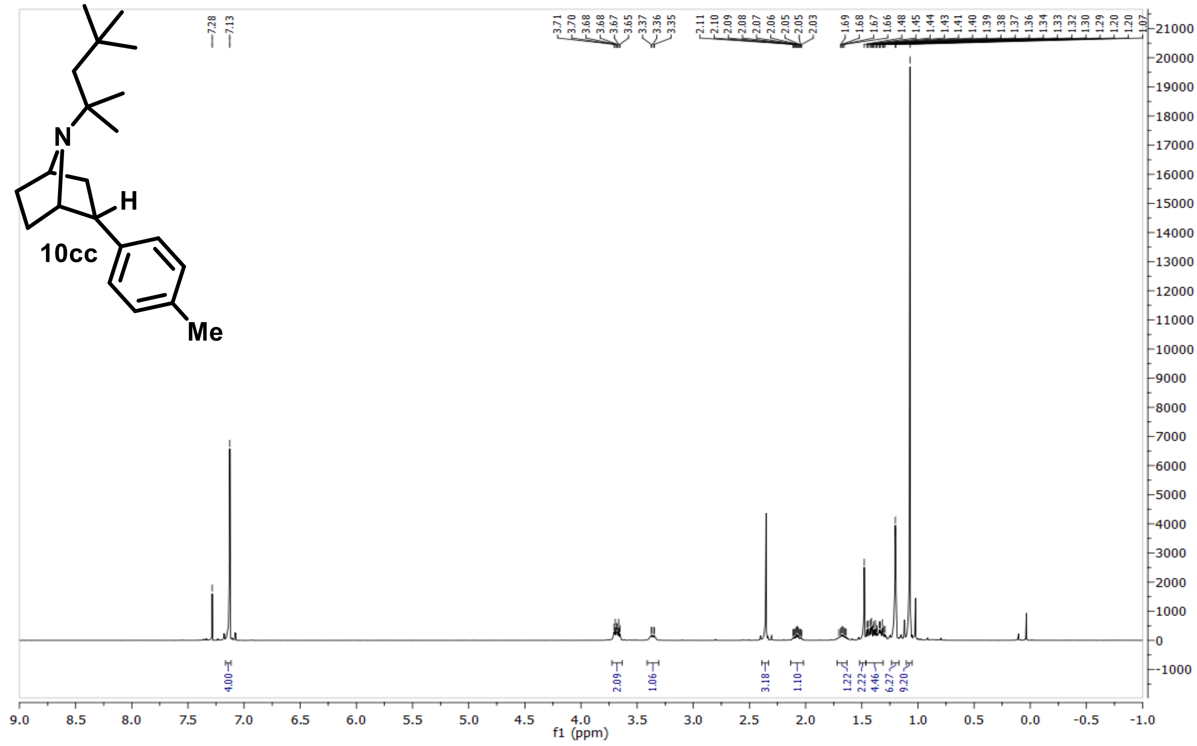

Figure S27: 10cc ^{1}H NMR, 400 MHz, $CDCl_3$

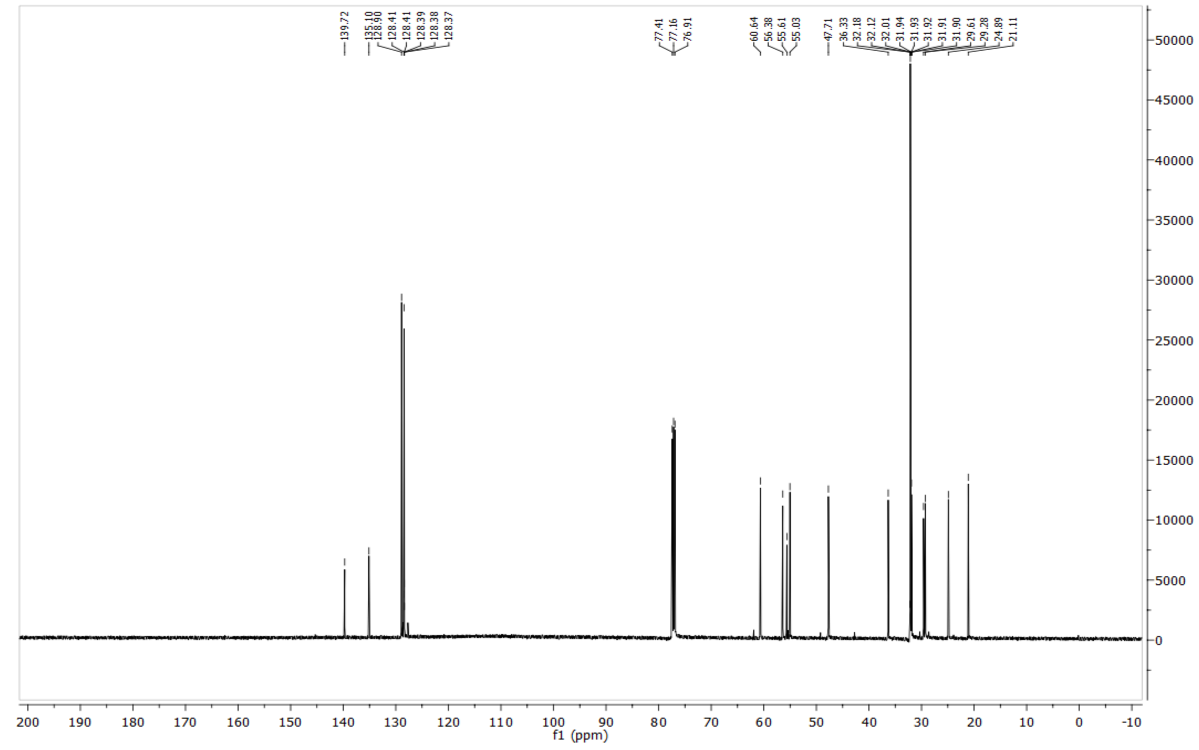

Figure S28: 10cc ^{13}C NMR, 126 MHz, $CDCl_3$

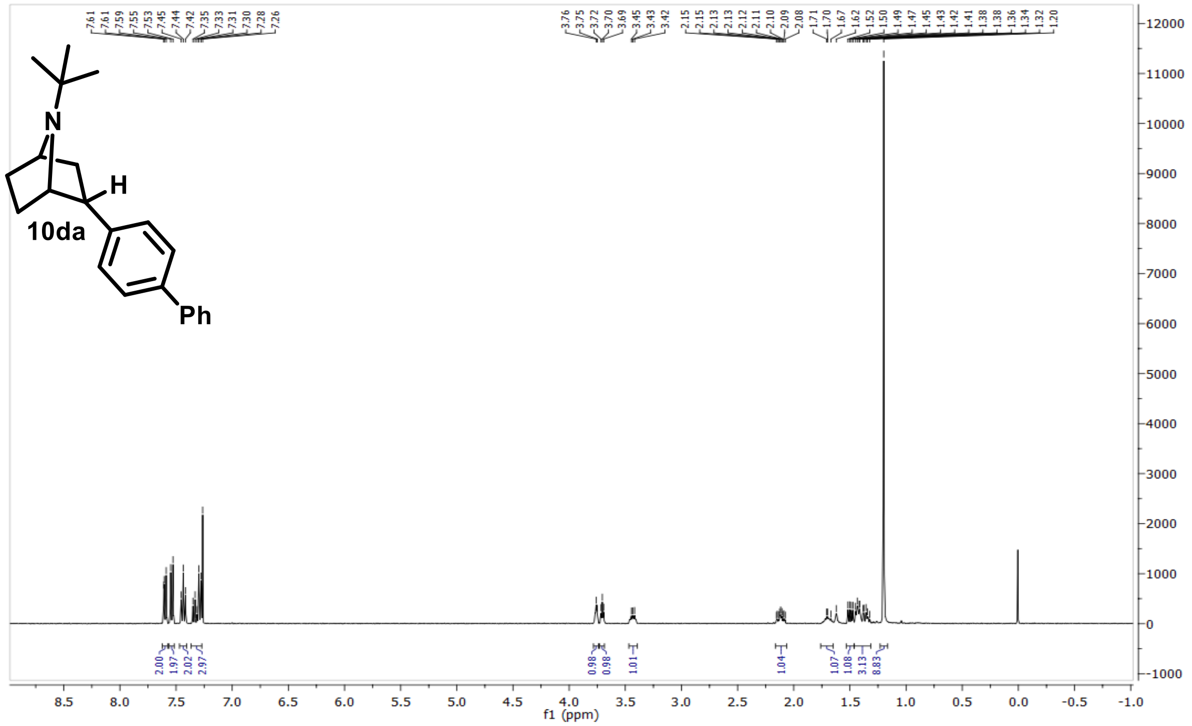

Figure S29: 10da ^{1}H NMR, 400 MHz, $CDCl_3$

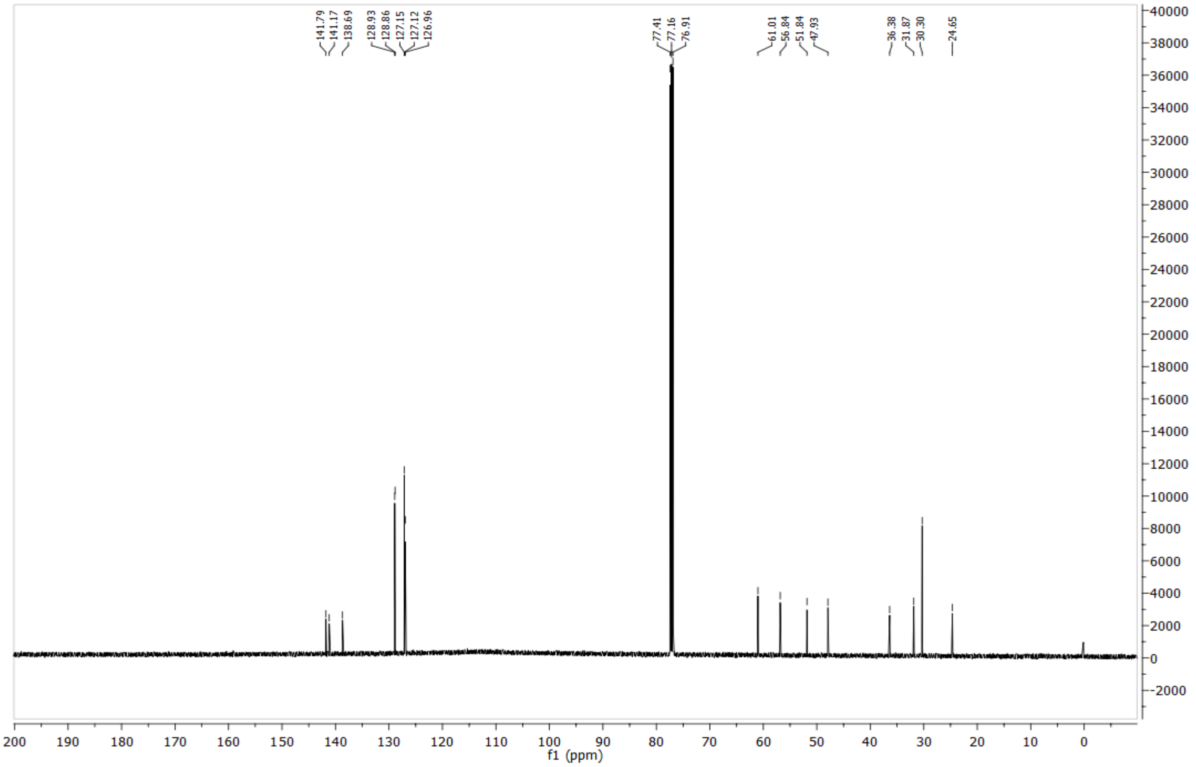

Figure S30: 10da ^{13}C NMR, 126 MHz, $CDCl_3$

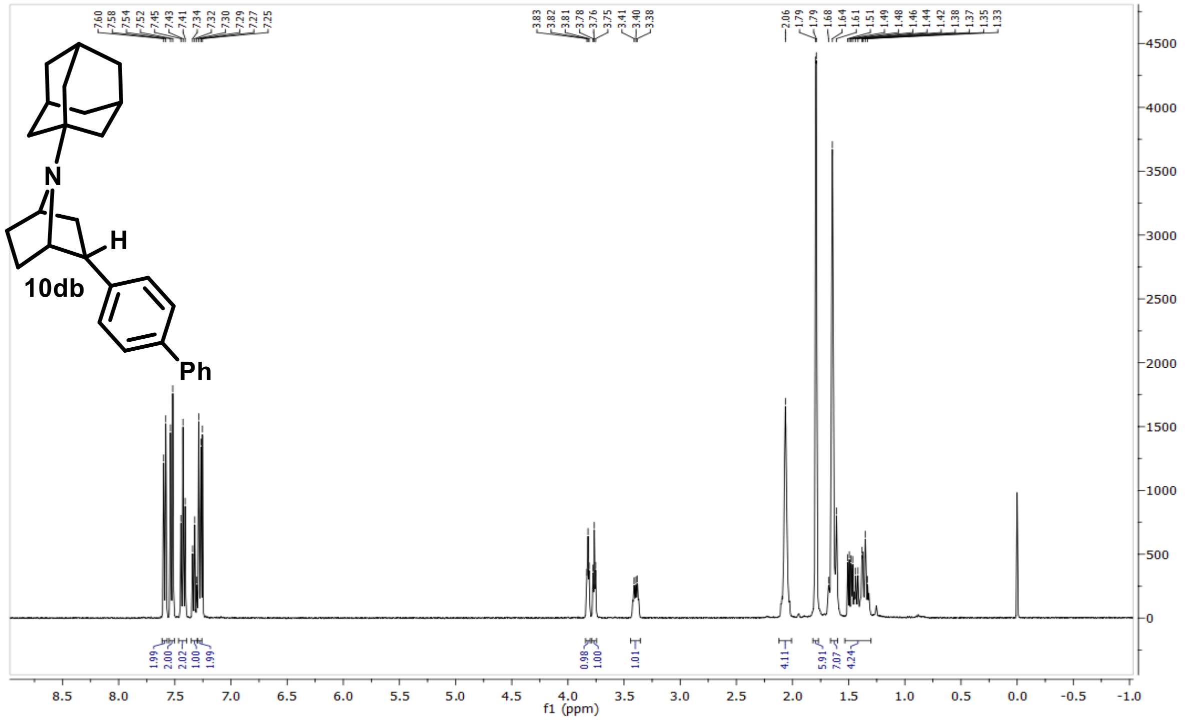

Figure S31: 10db ^{1}H NMR, 400 MHz, $CDCl_3$

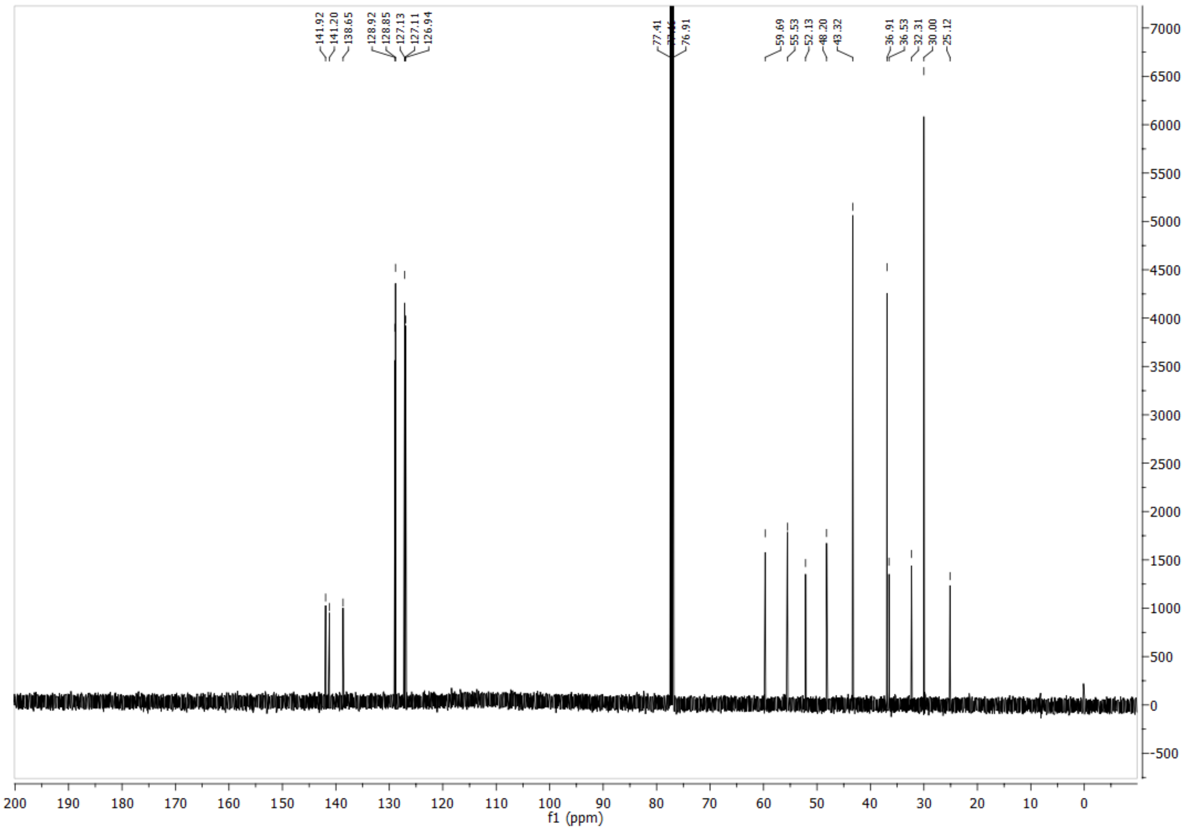

Figure S32: 10db ^{13}C NMR, 125 MHz, $CDCl_3$

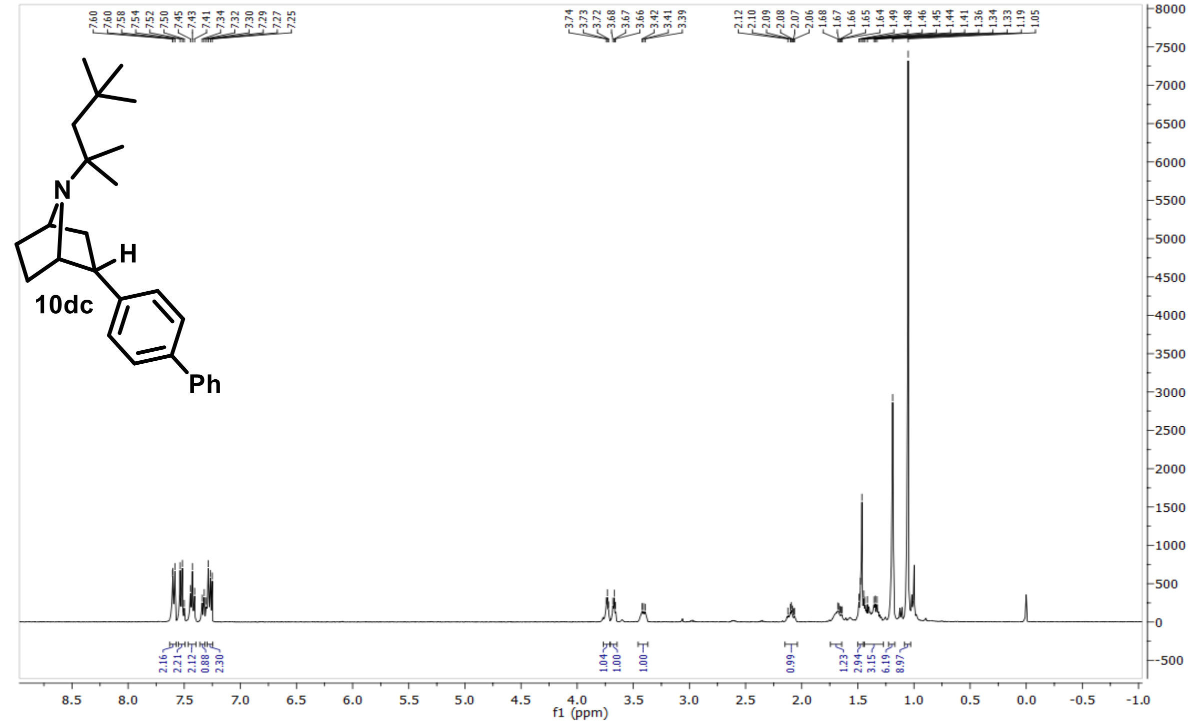

Figure S33: 10dc ^{1}H NMR, 400 MHz, $CDCl_3$

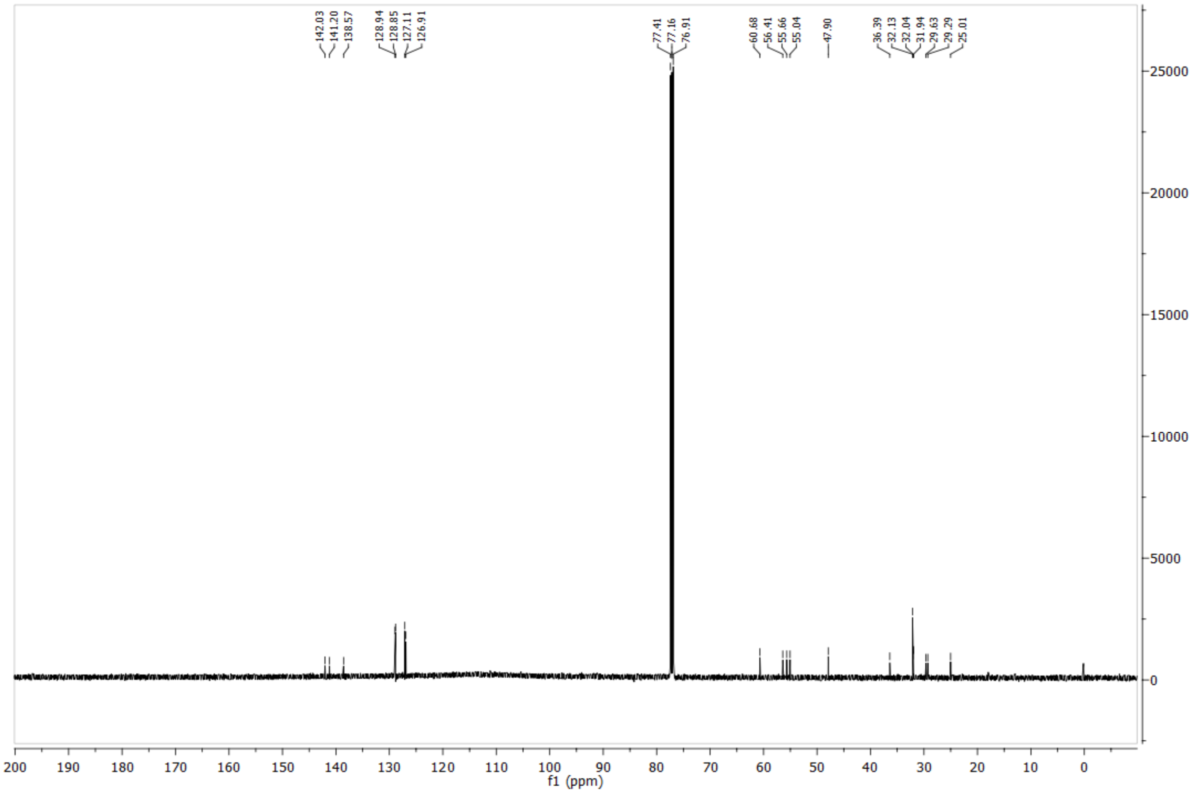

Figure S34: 10dc ^{13}C NMR, 126 MHz, $CDCl_3$

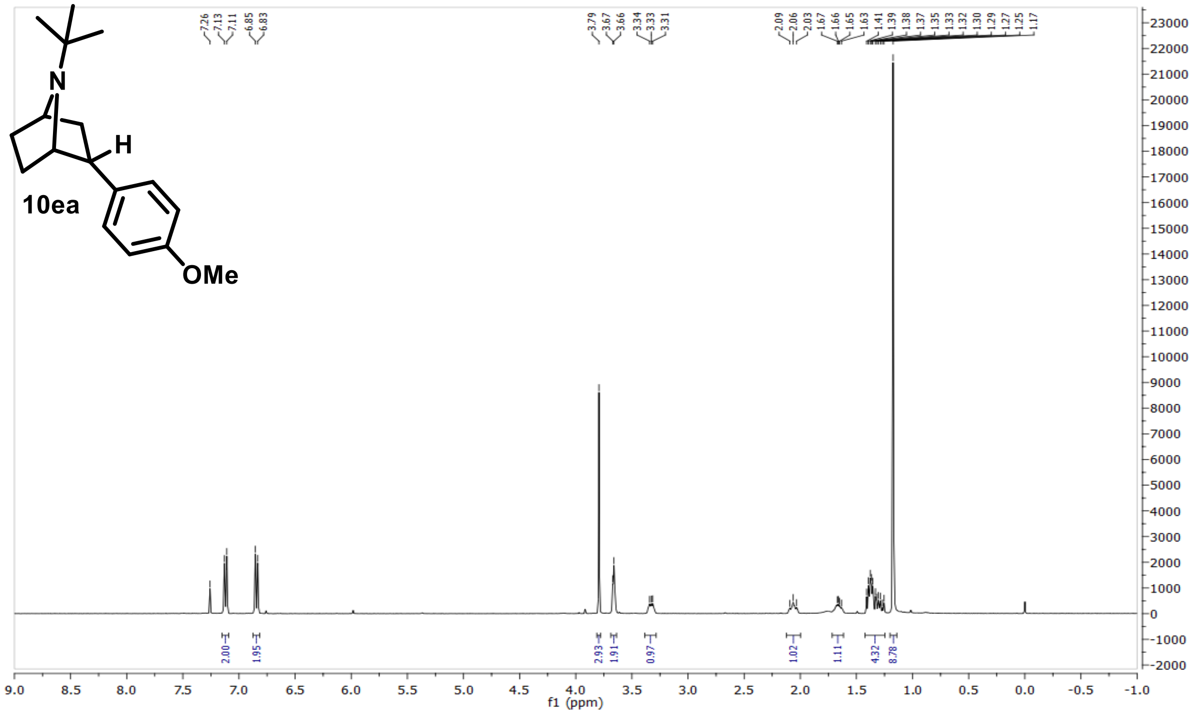

Figure S35: 10ea ^{1}H NMR, 400 MHz, $CDCl_3$

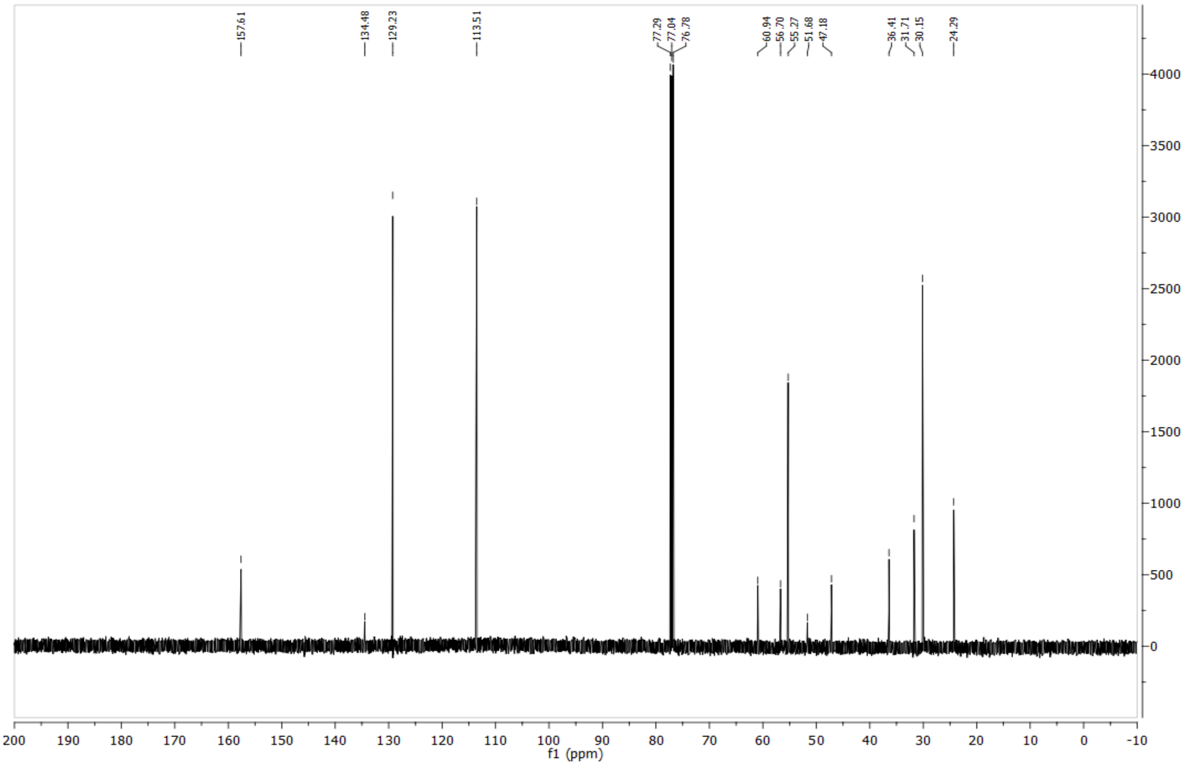

Figure S36: 10ea ^{13}C NMR, 126 MHz, $CDCl_3$

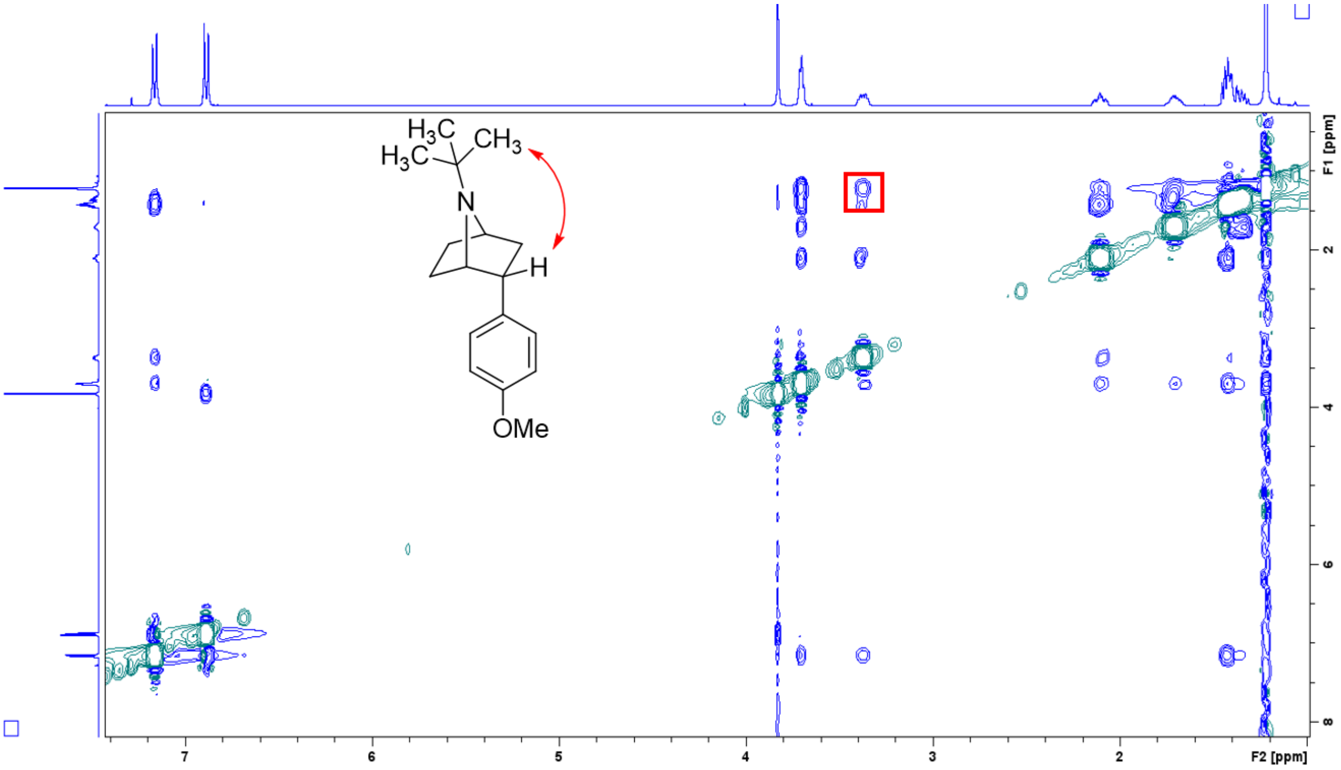

Figure S37: 10ea NOESY, 400 MHz, $CDCl_3$

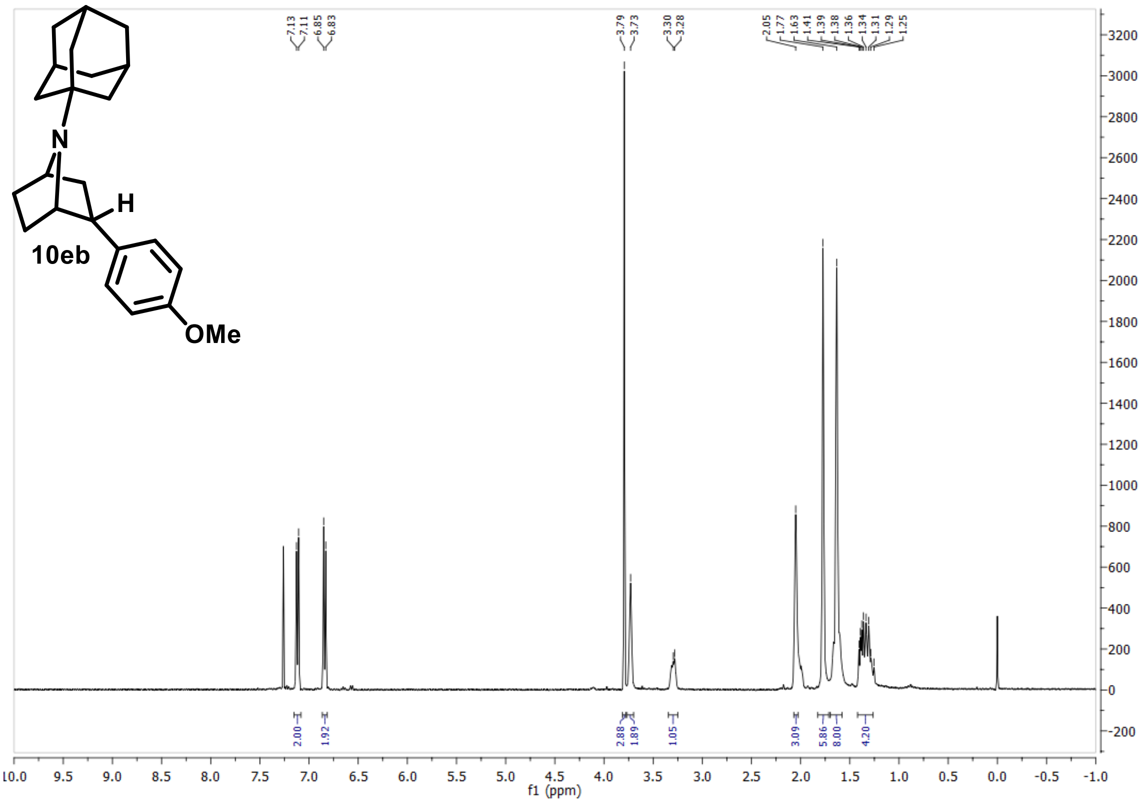

Figure S38: 10eb ^{1}H NMR, 400 MHz, CDCl$_3$

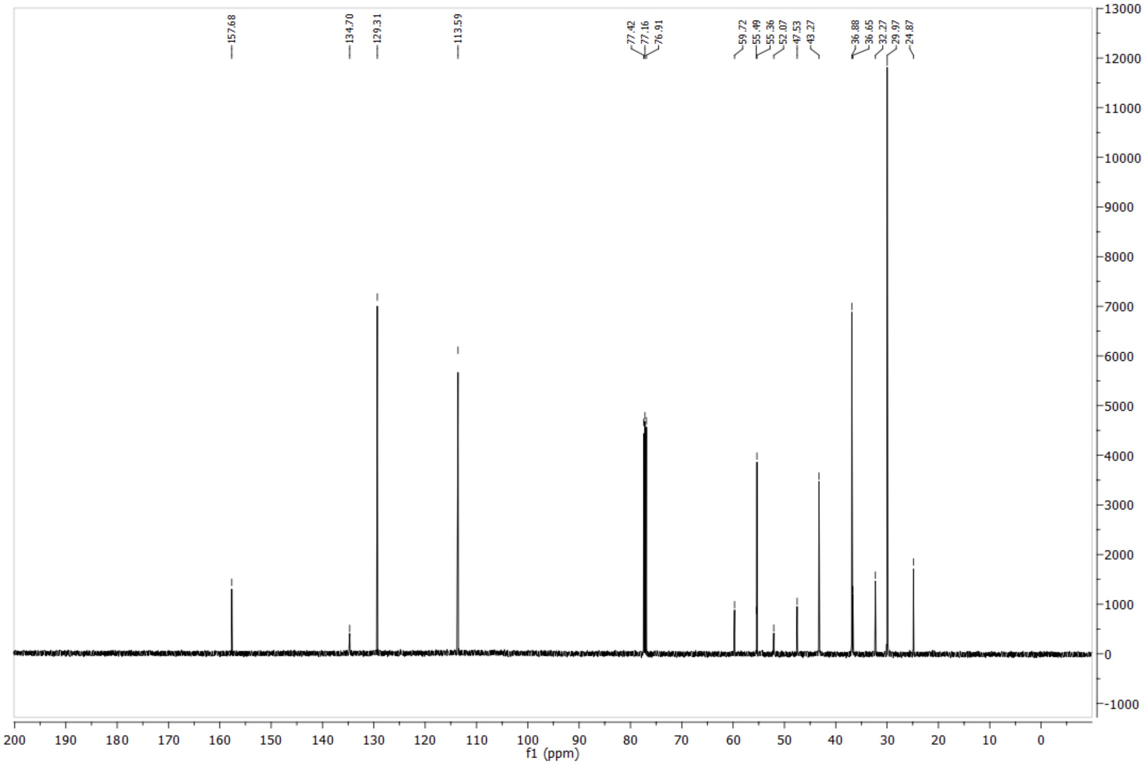

Figure S39: 10eb ^{13}C NMR, 125 MHz, CDCl$_3$

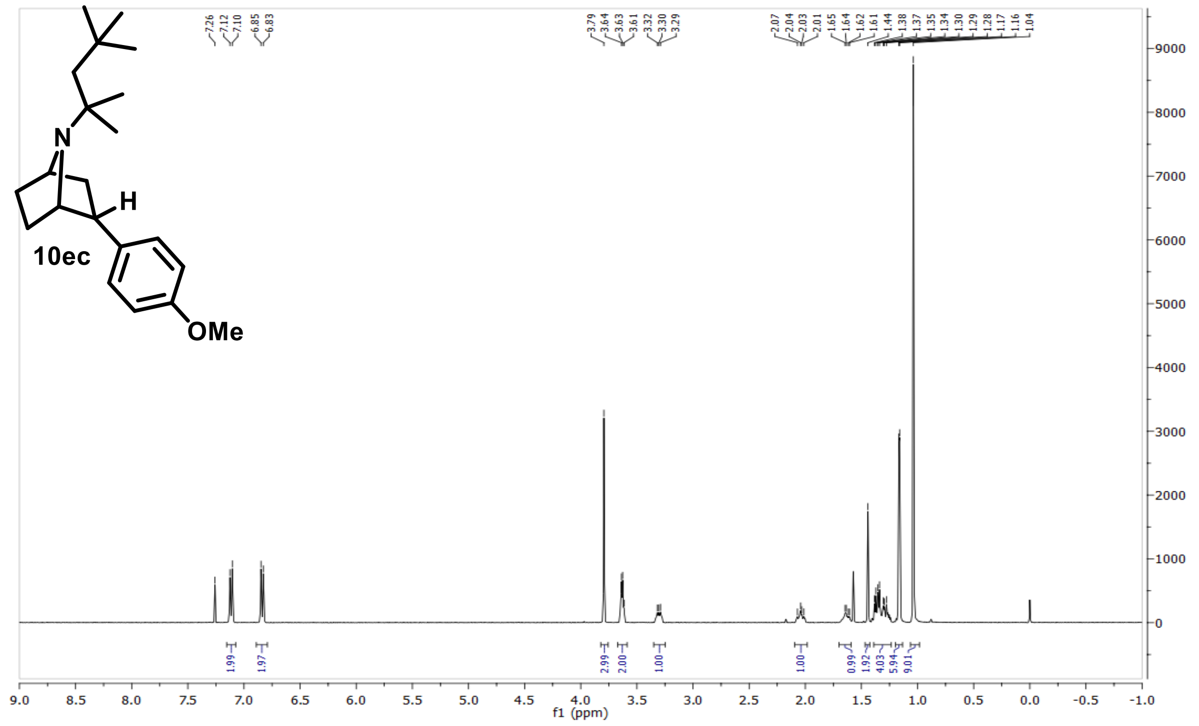

Figure S40: 10ec ^{1}H NMR, 400 MHz, $CDCl_3$

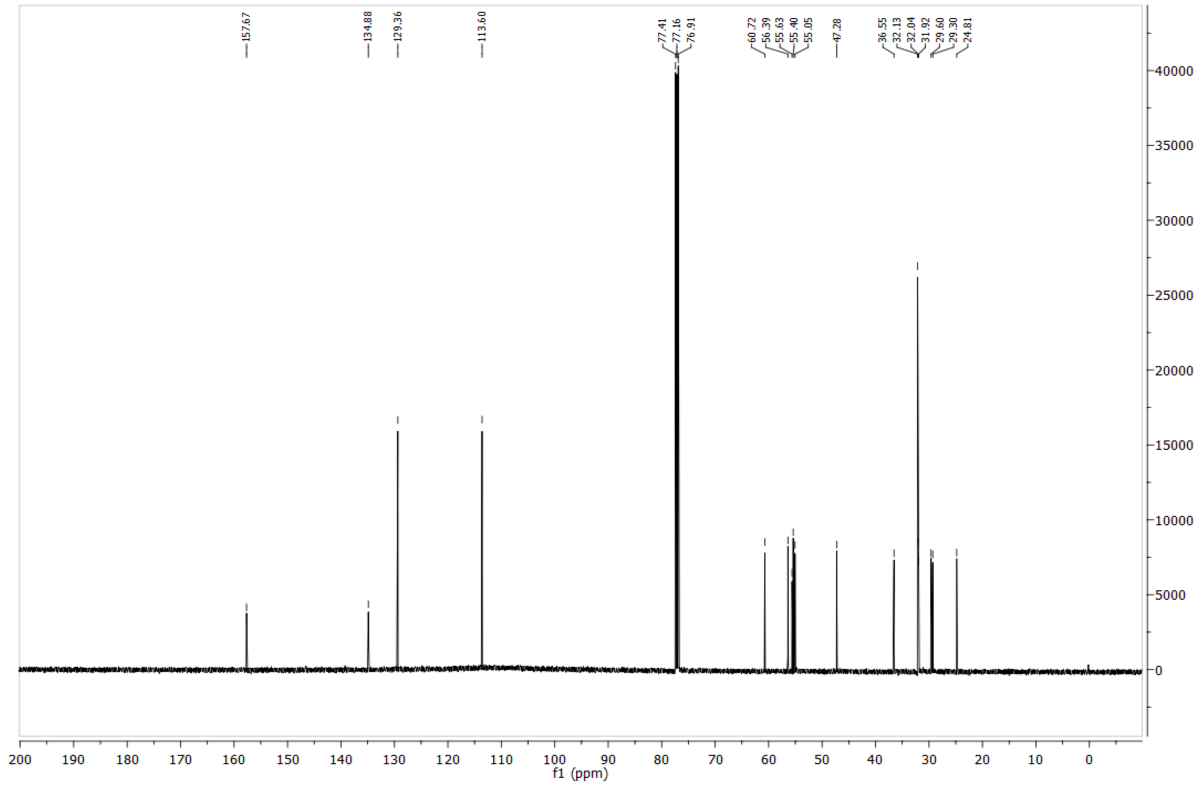

Figure S41: 10ec ^{13}C NMR, 126 MHz, $CDCl_3$

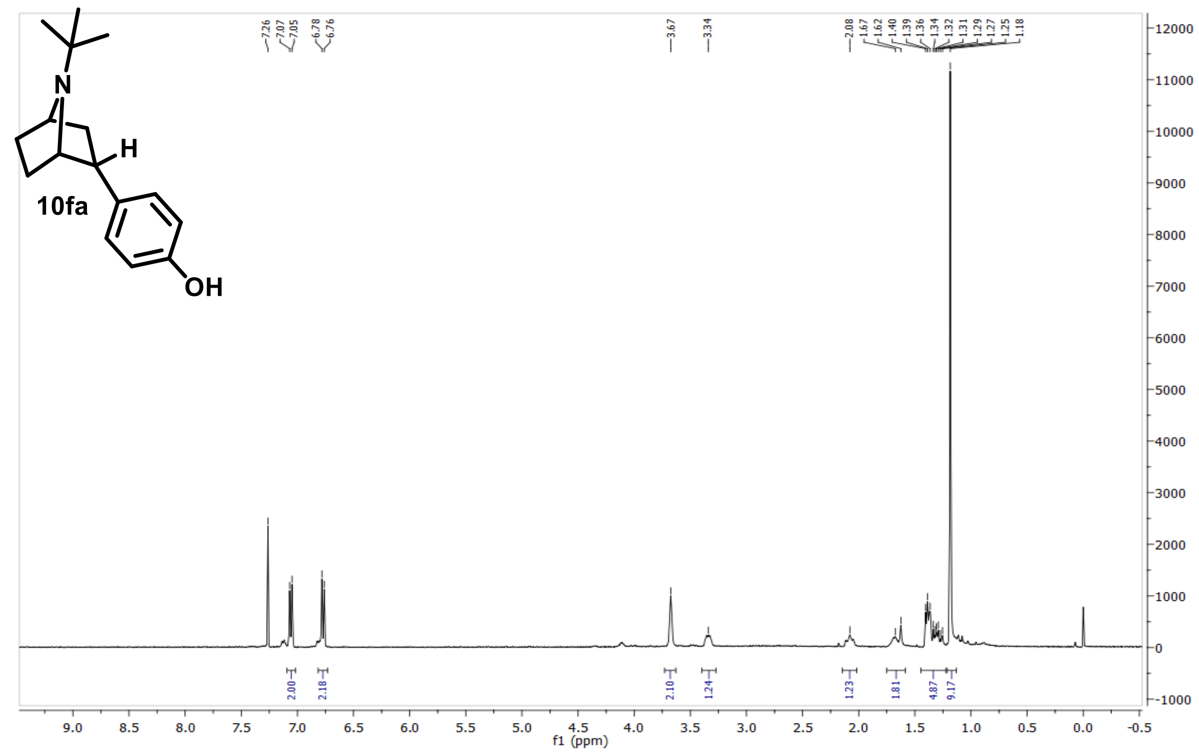

Figure S42: 10fa ¹H NMR, 400 MHz, CDCl₃

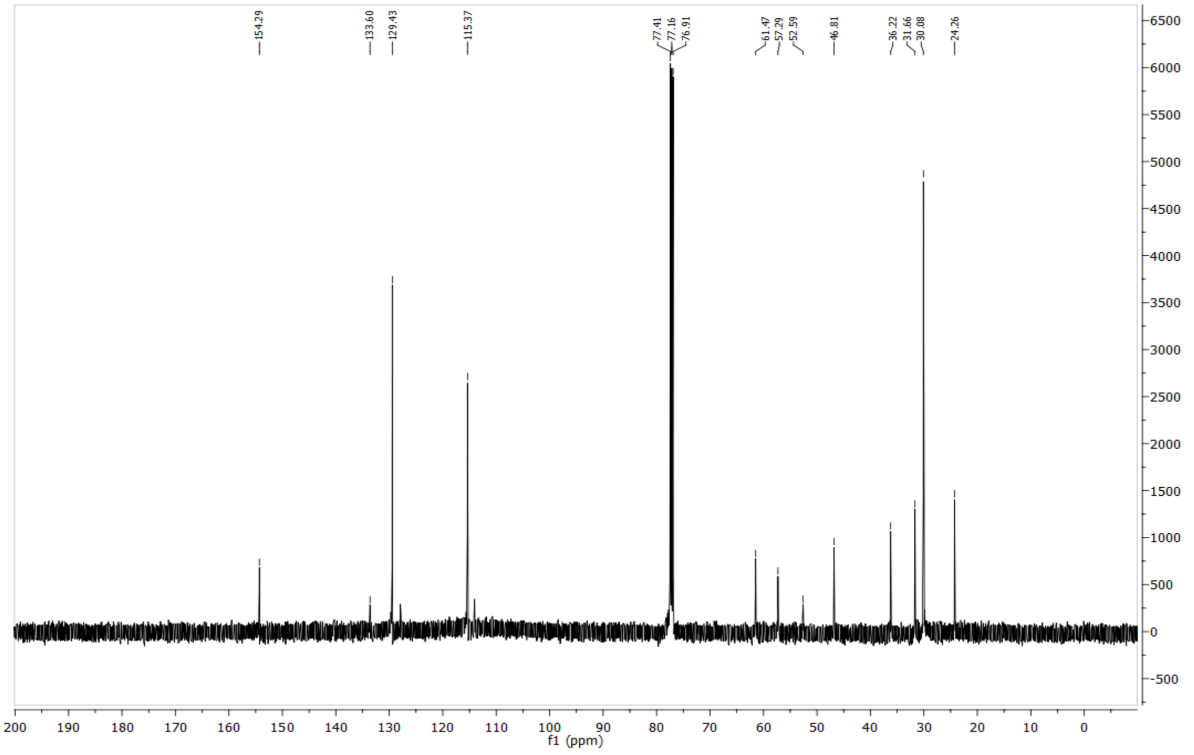

Figure S43: 10fa ¹³C NMR, 126 MHz, CDCl₃

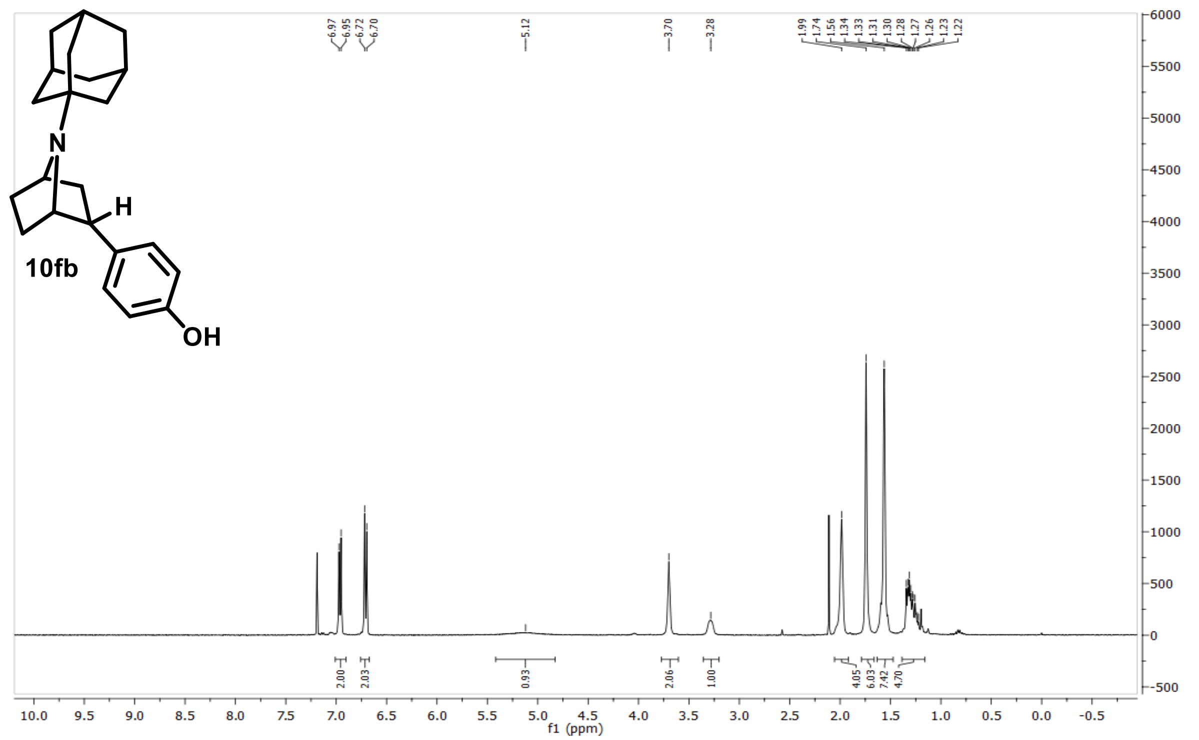

Figure S44: 10fb ^{1}H NMR, 400 MHz, $CDCl_3$

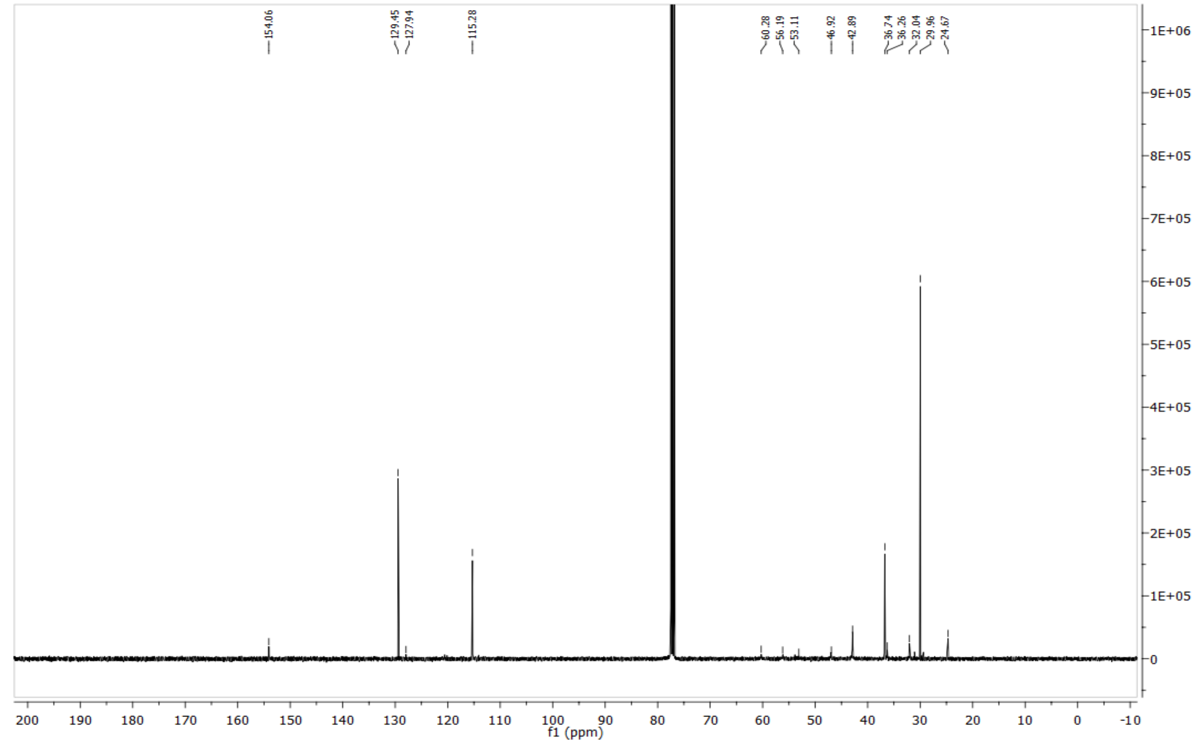

Figure S45: 10fb ^{13}C NMR, 126 MHz, $CDCl_3$

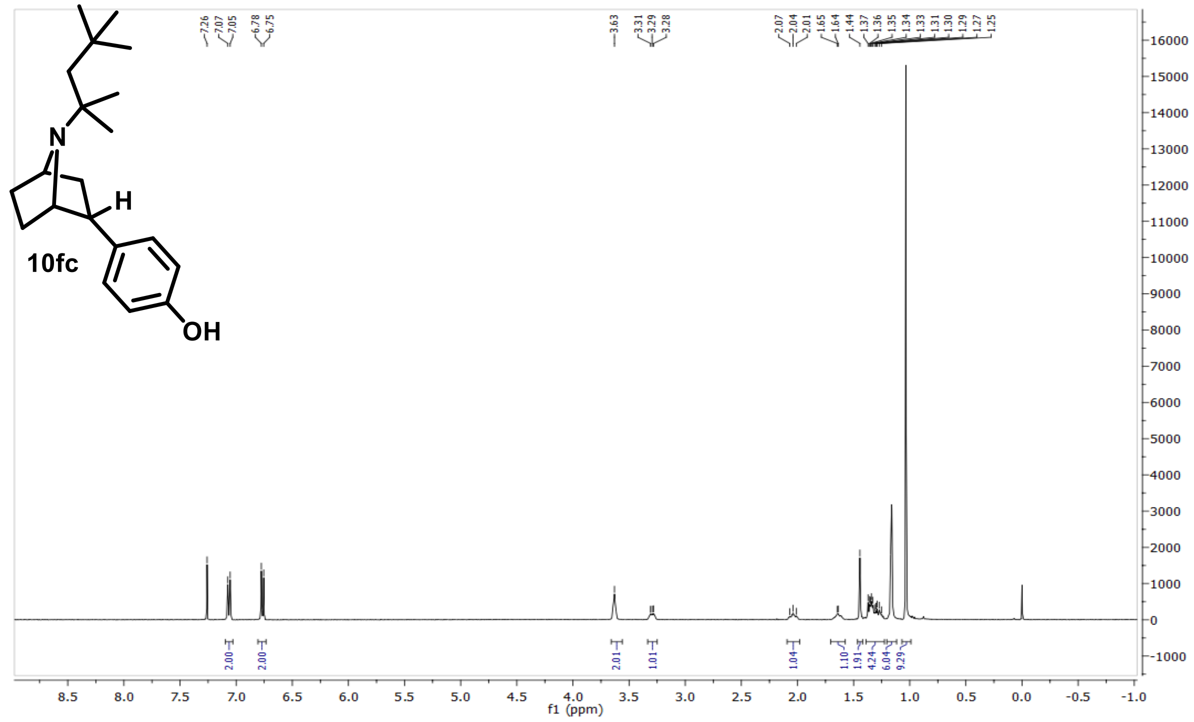

Figure S46: 10fc ^{1}H NMR, 400 MHz, $CDCl_3$

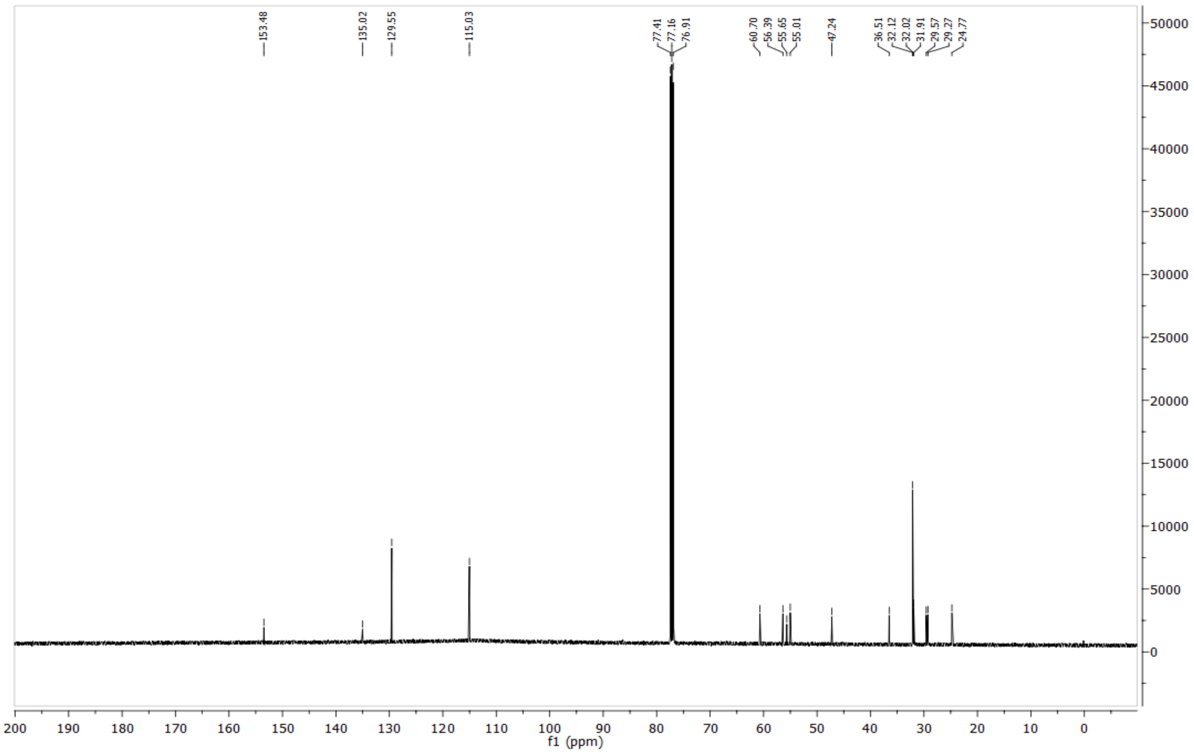

Figure S47: 10fc ^{13}C NMR, 126 MHz, $CDCl_3$

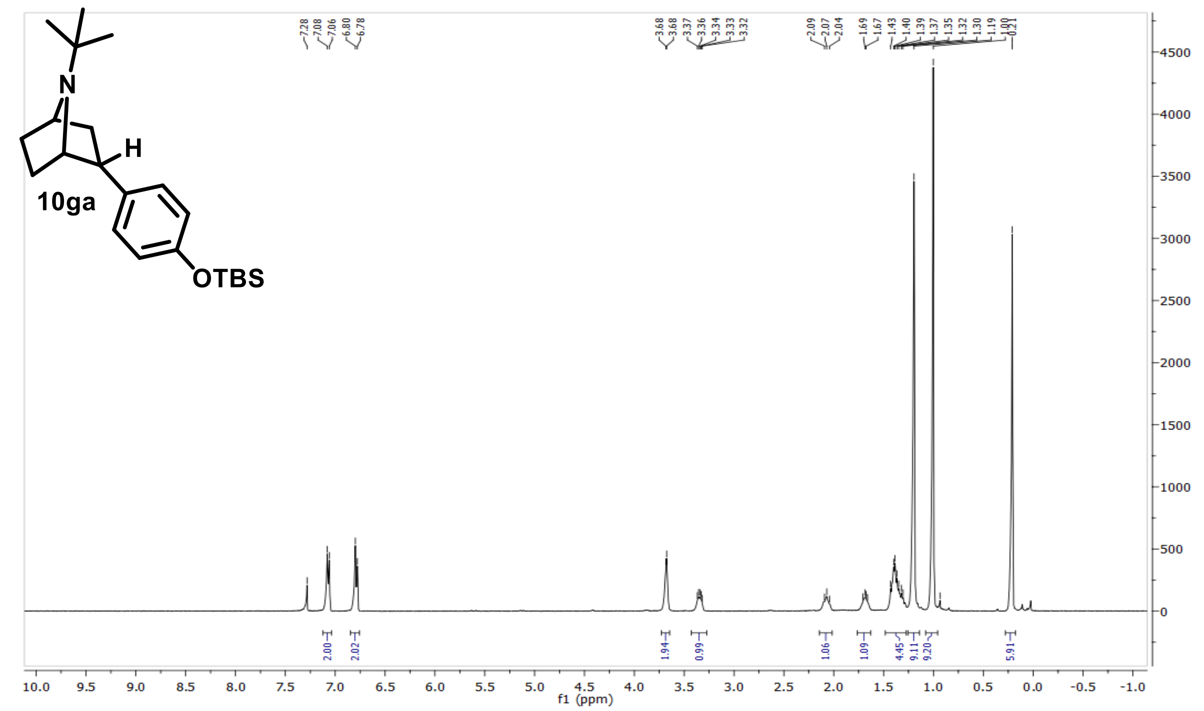

Figure S48: 10ga ^{1}H NMR, 400 MHz, $CDCl_3$

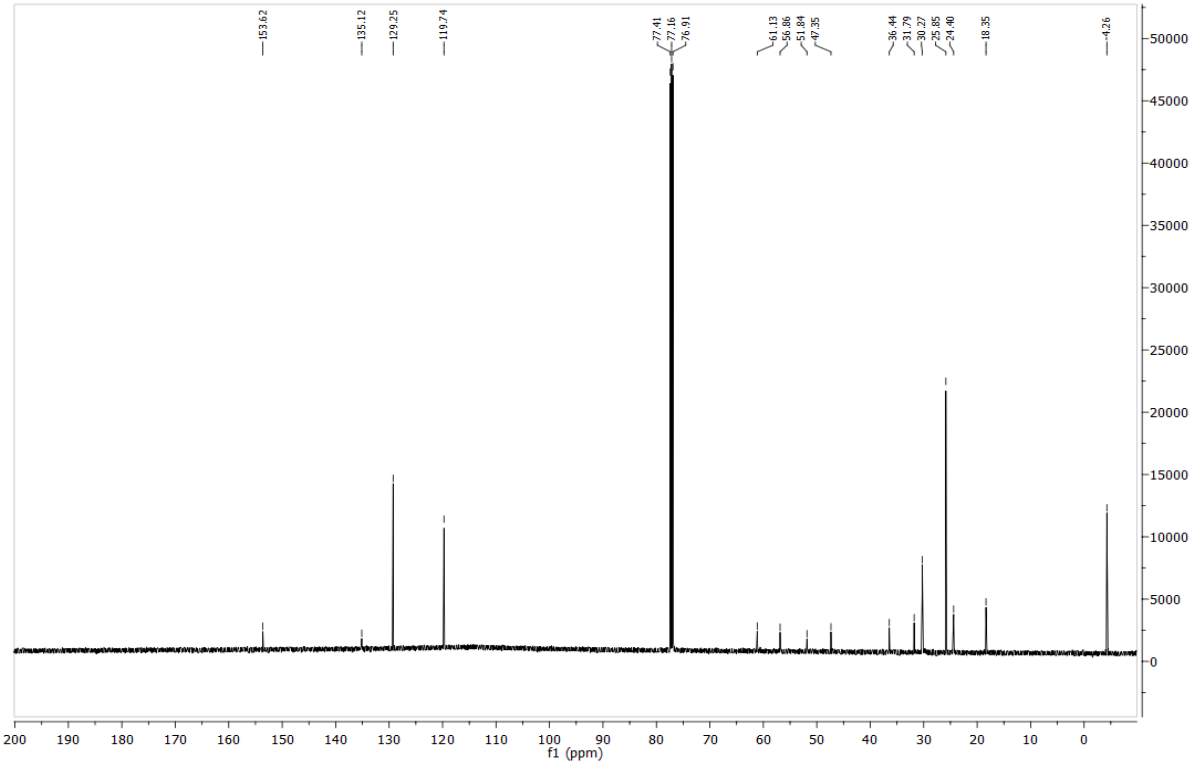

Figure S49: 10ga ^{13}C NMR, 126 MHz, $CDCl_3$

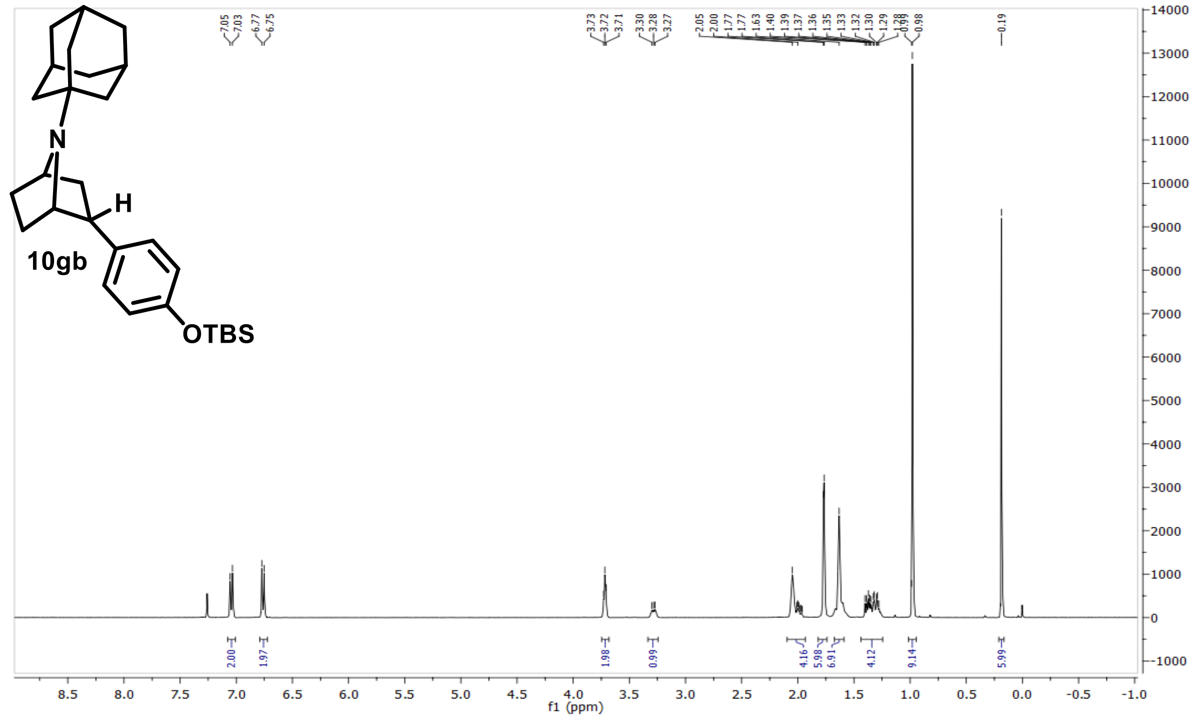

Figure S50: 10gb ^{1}H NMR, 400 MHz, $CDCl_3$

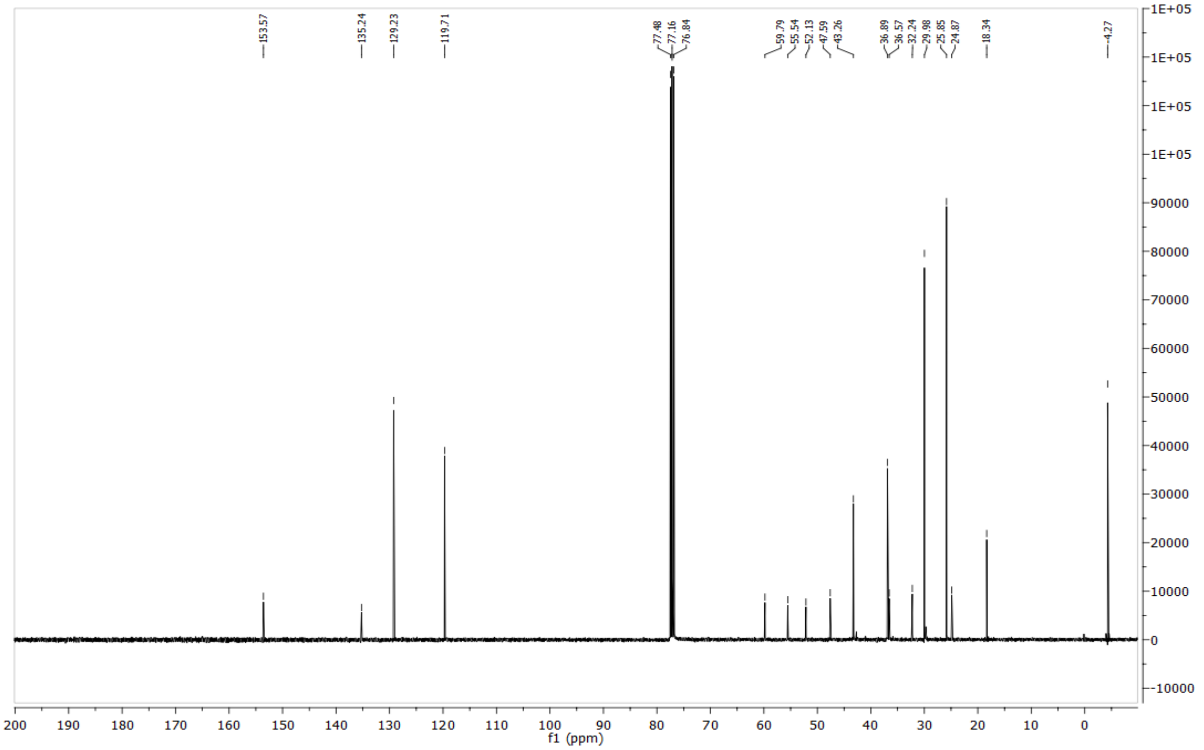

Figure S51: 10gb ^{13}C NMR, 101 MHz, $CDCl_3$

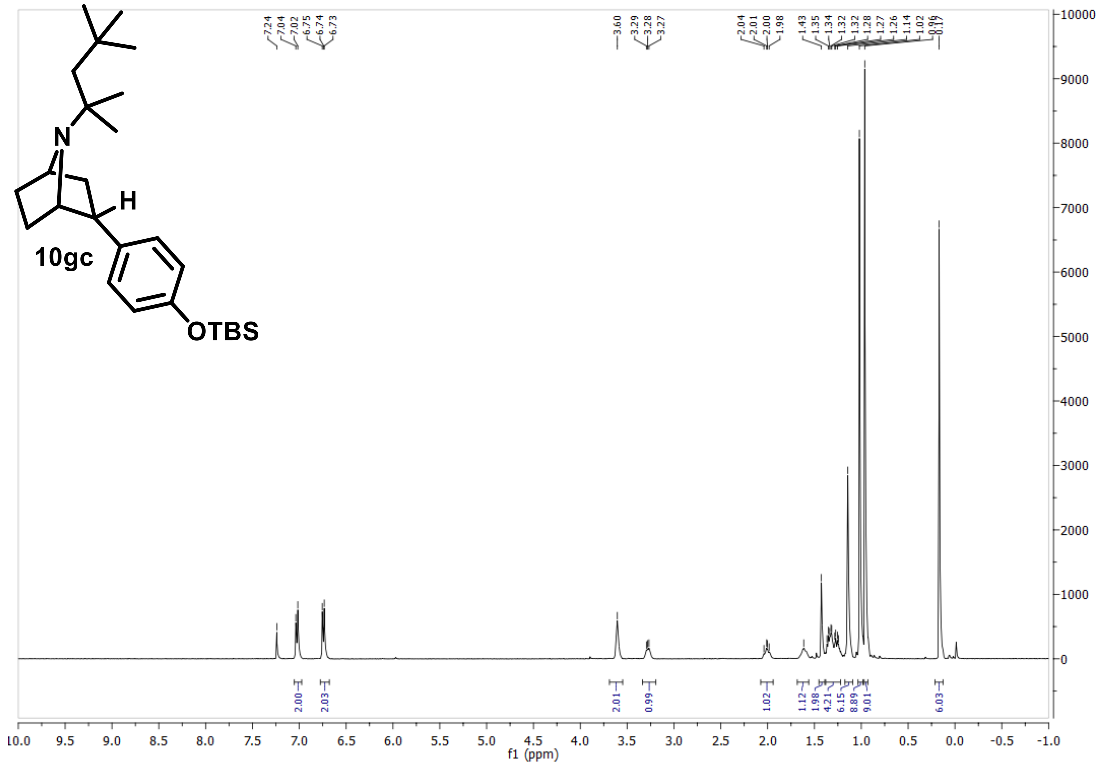

Figure S52: 10gc ^{1}H NMR, 400 MHz, $CDCl_3$

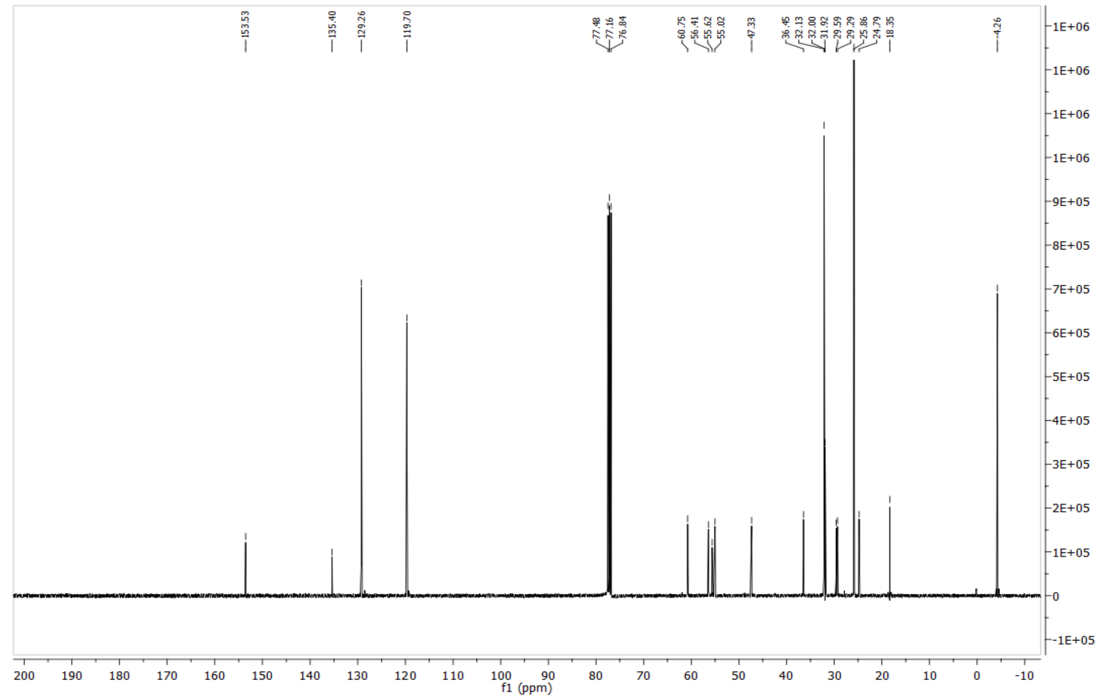

Figure S53: 10gc ^{13}C NMR, 126 MHz, $CDCl_3$

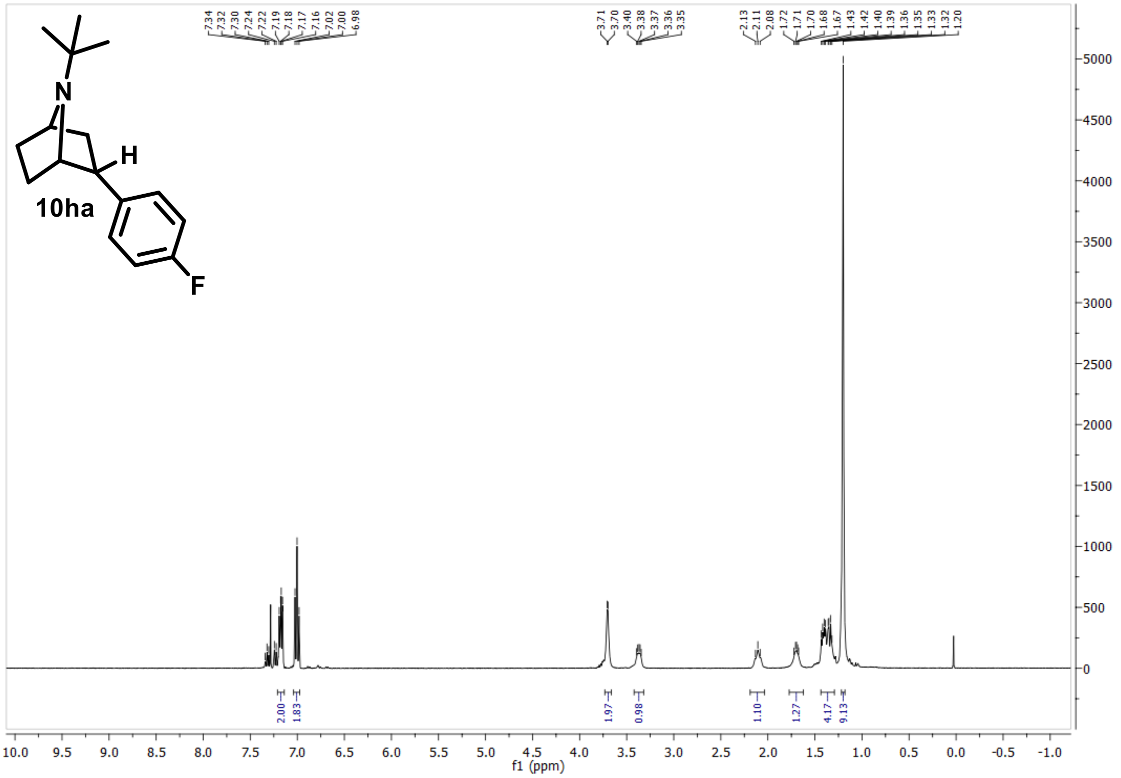

Figure S54: 10ha ^{1}H NMR, 400 MHz, $CDCl_3$

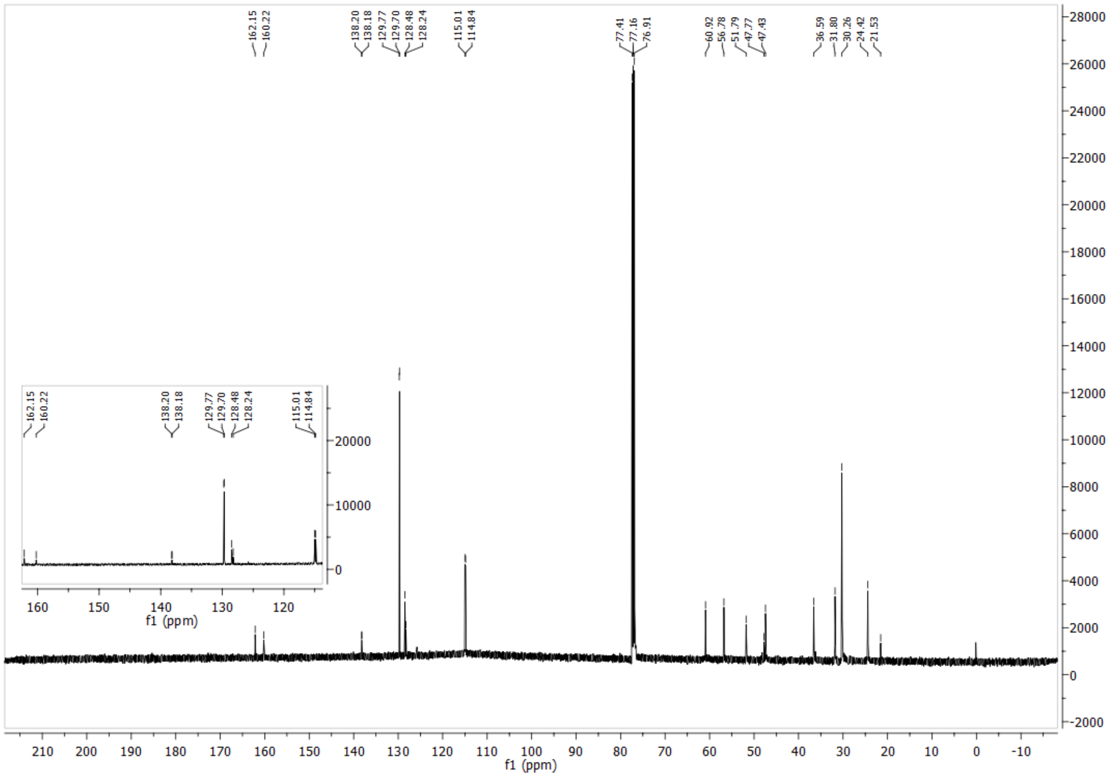

Figure S55: 10ha ^{13}C NMR, 126 MHz, $CDCl_3$

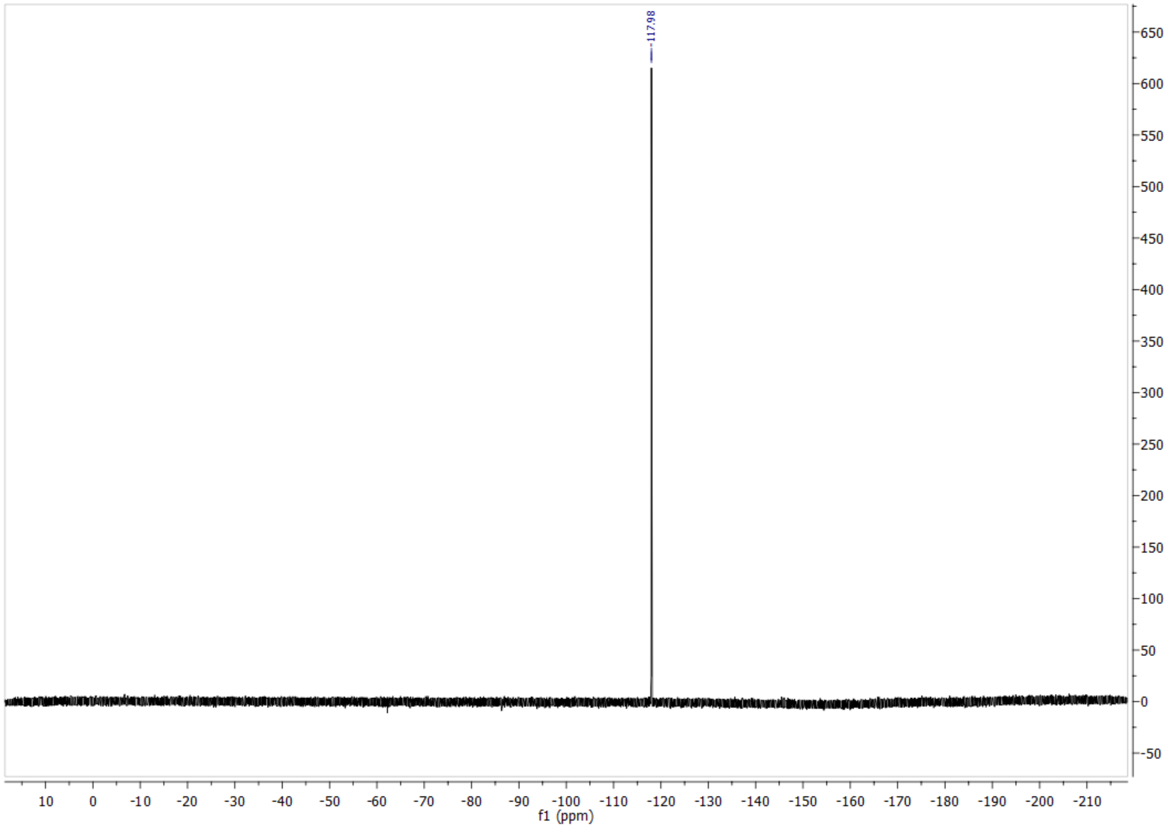

Figure S56: 10ha ^{19}F NMR, 376 MHz, $CDCl_3$

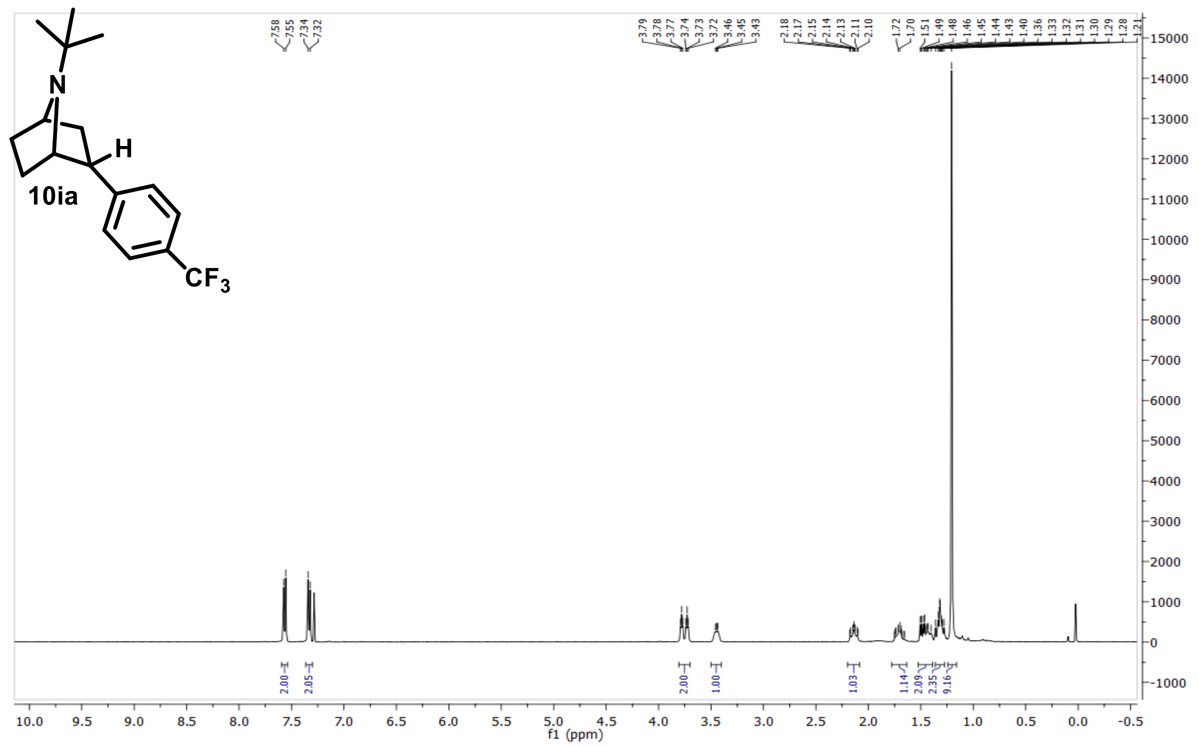

Figure S57: 10ia ^{1}H NMR, 400 MHz, $CDCl_3$

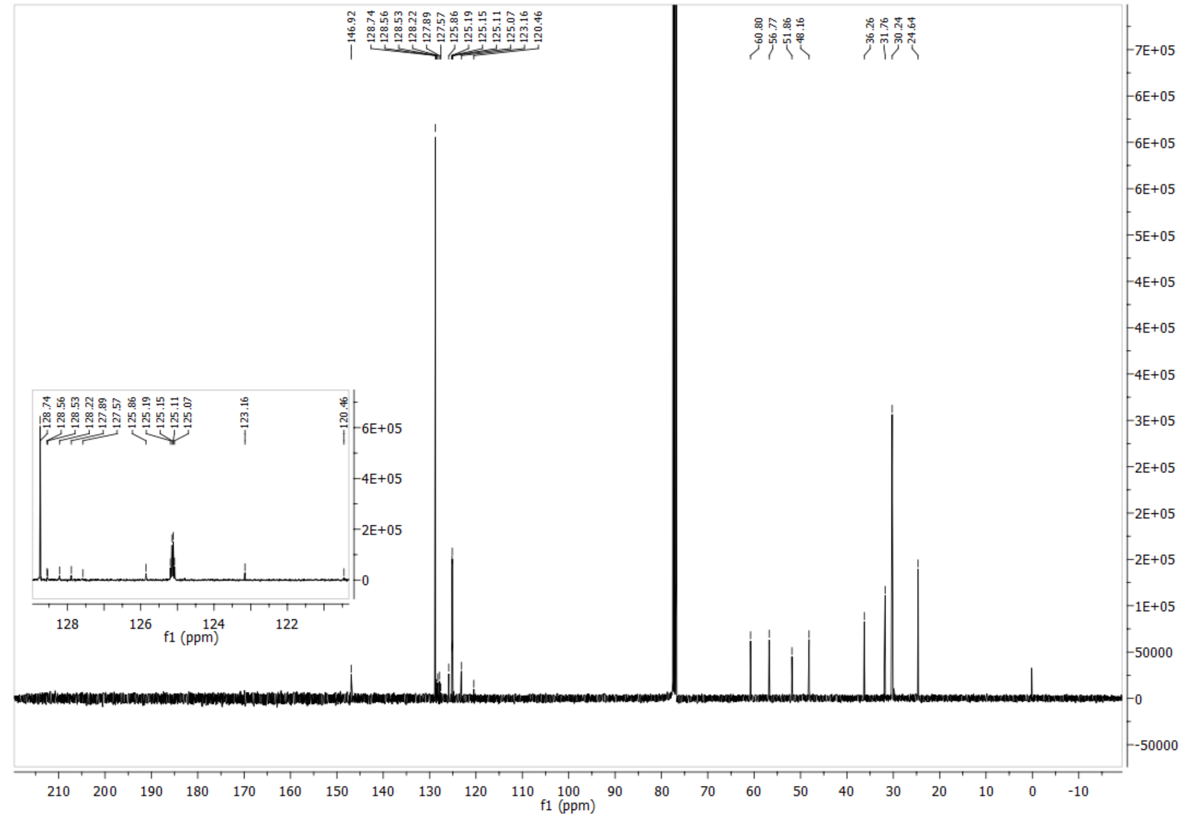

Figure S58: 10ia ^{13}C NMR, 101 MHz, $CDCl_3$

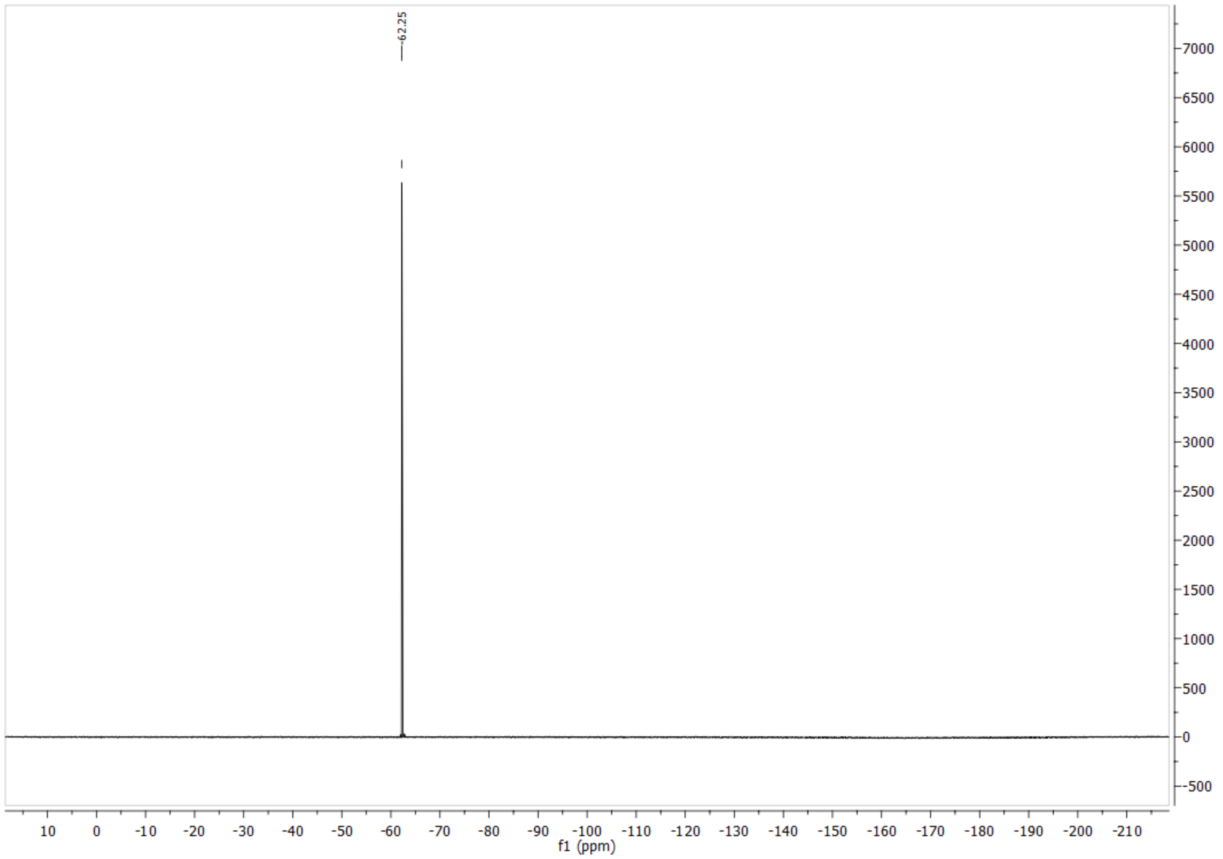

Figure S59: 10ia ^{19}F NMR, 376 MHz, $CDCl_3$

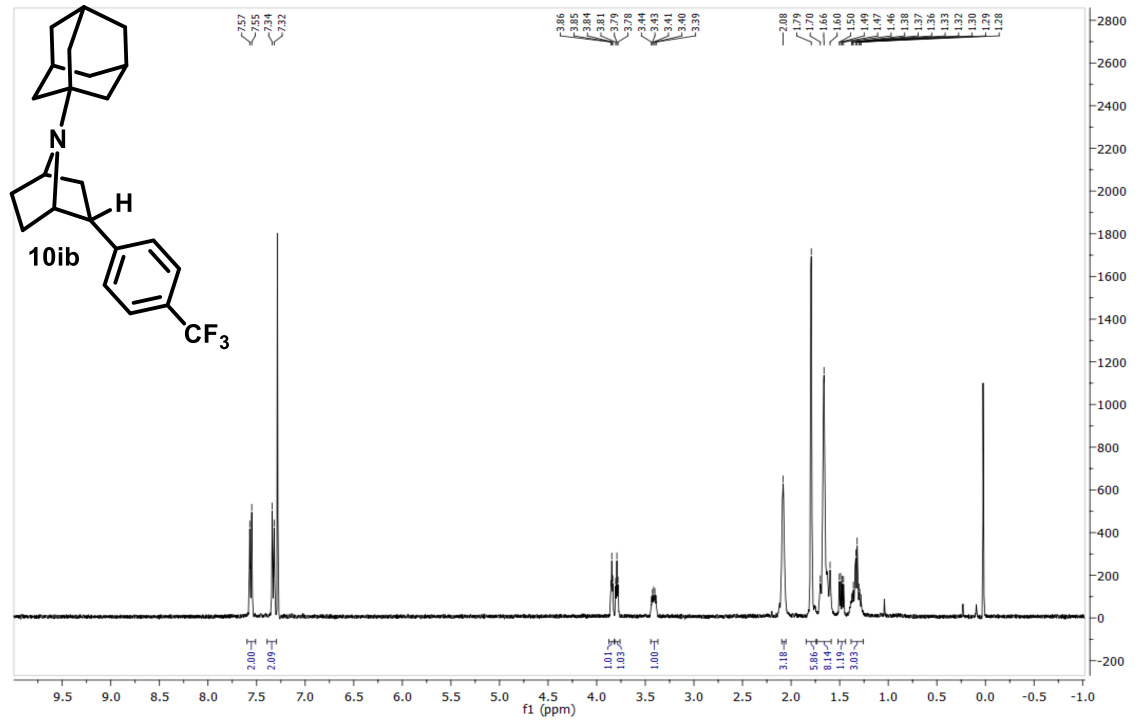

Figure S60: 10ib ^{1}H NMR, 400 MHz, $CDCl_3$

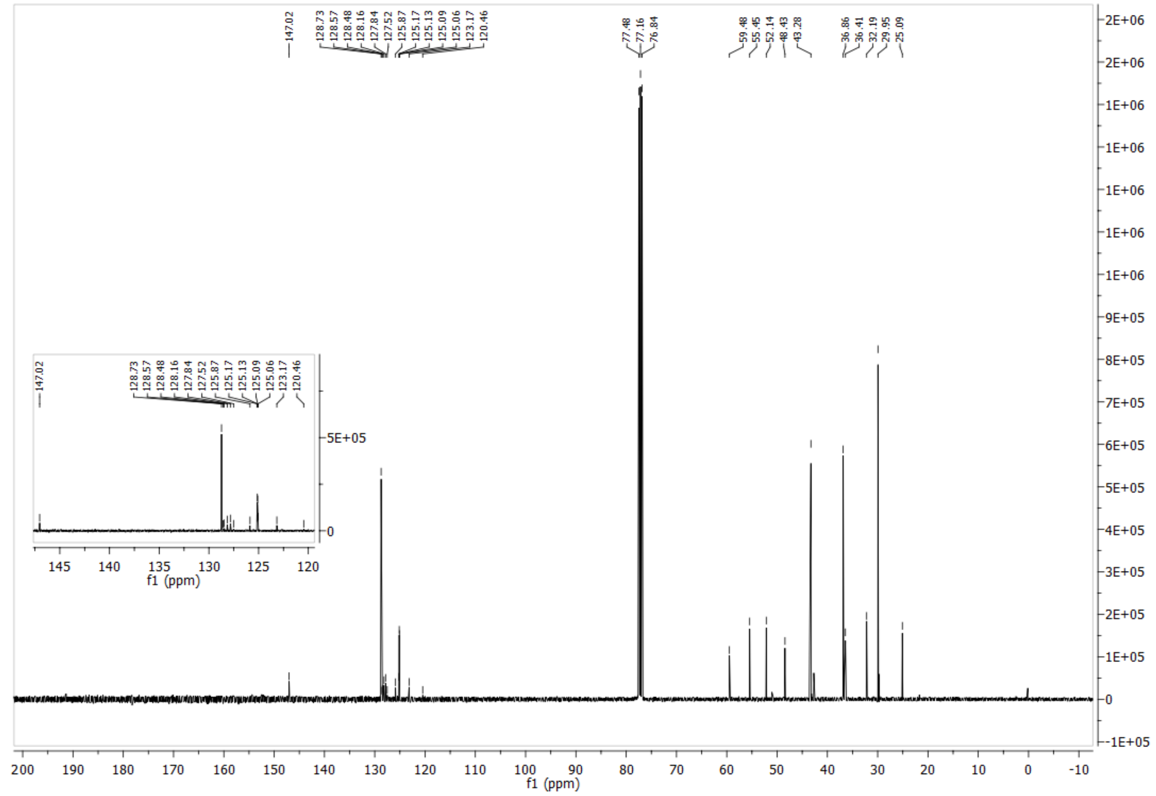

Figure S61: 10ib ^{13}C NMR, 101 MHz, $CDCl_3$

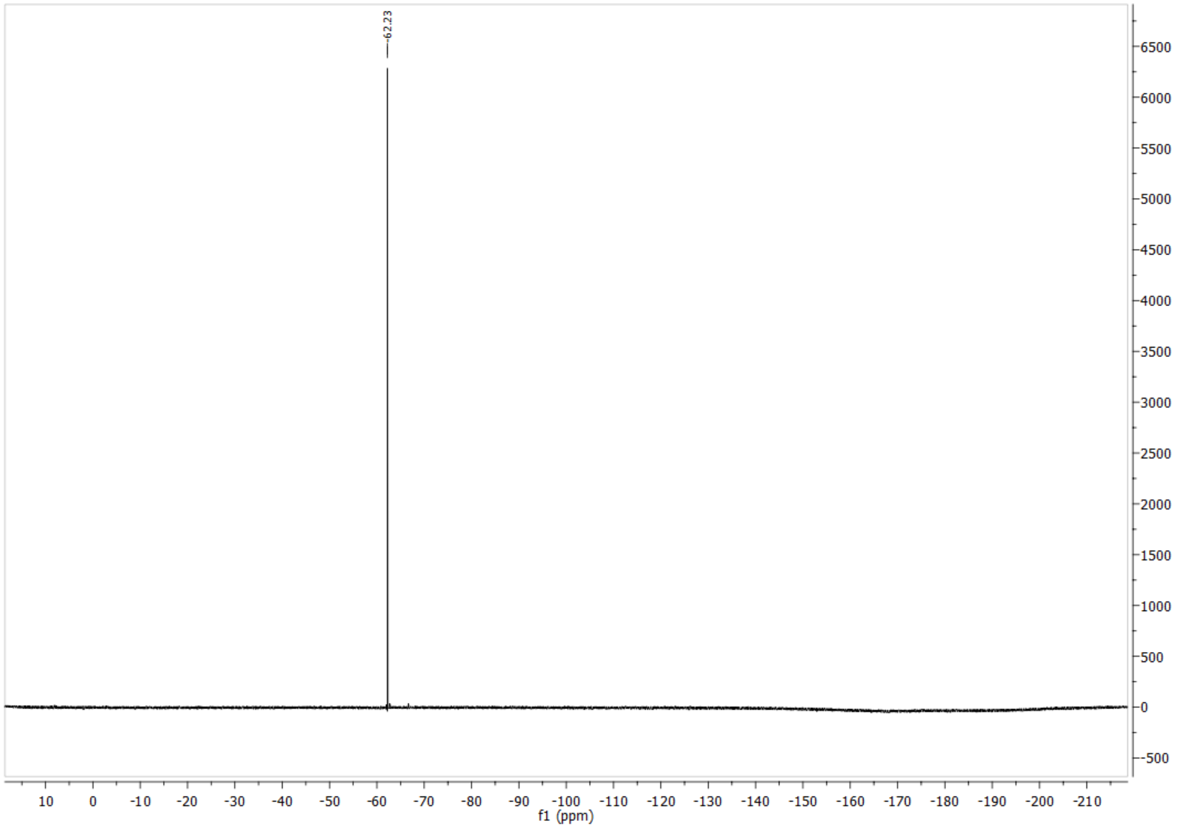

Figure S62: 10ib ^{19}F NMR, 376 MHz, $CDCl_3$

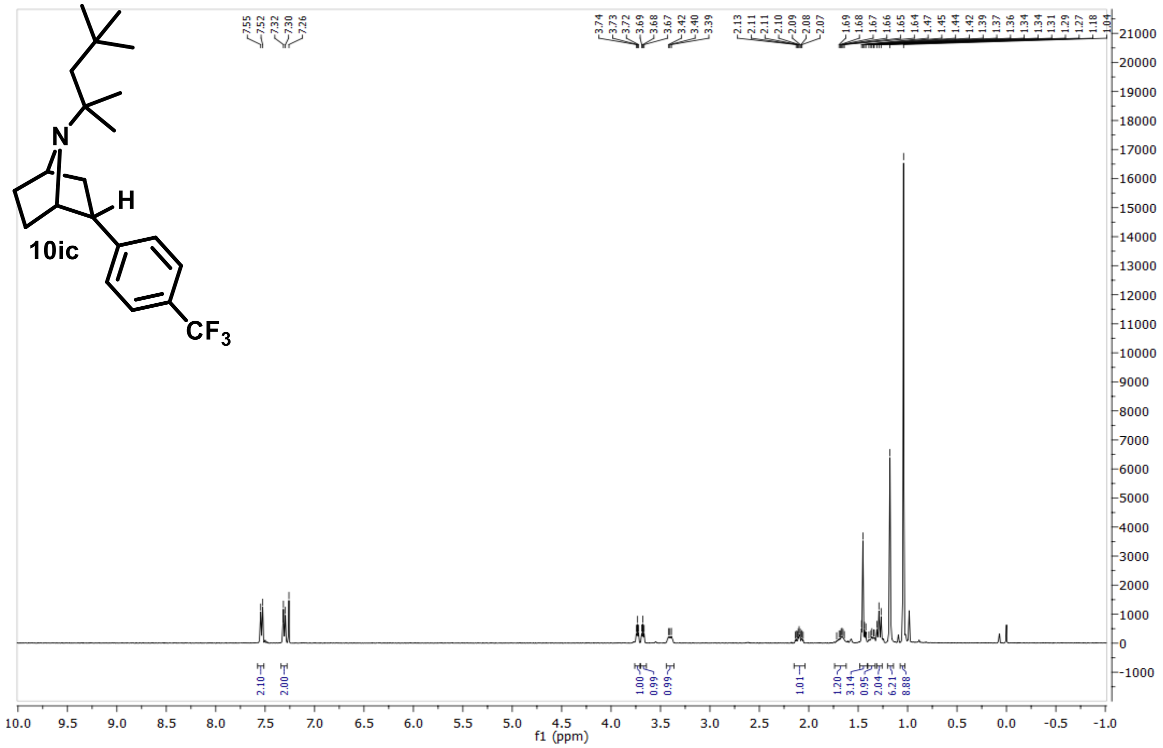

Figure S63: 10ic ^{1}H NMR, 400 MHz, CDCl$_3$

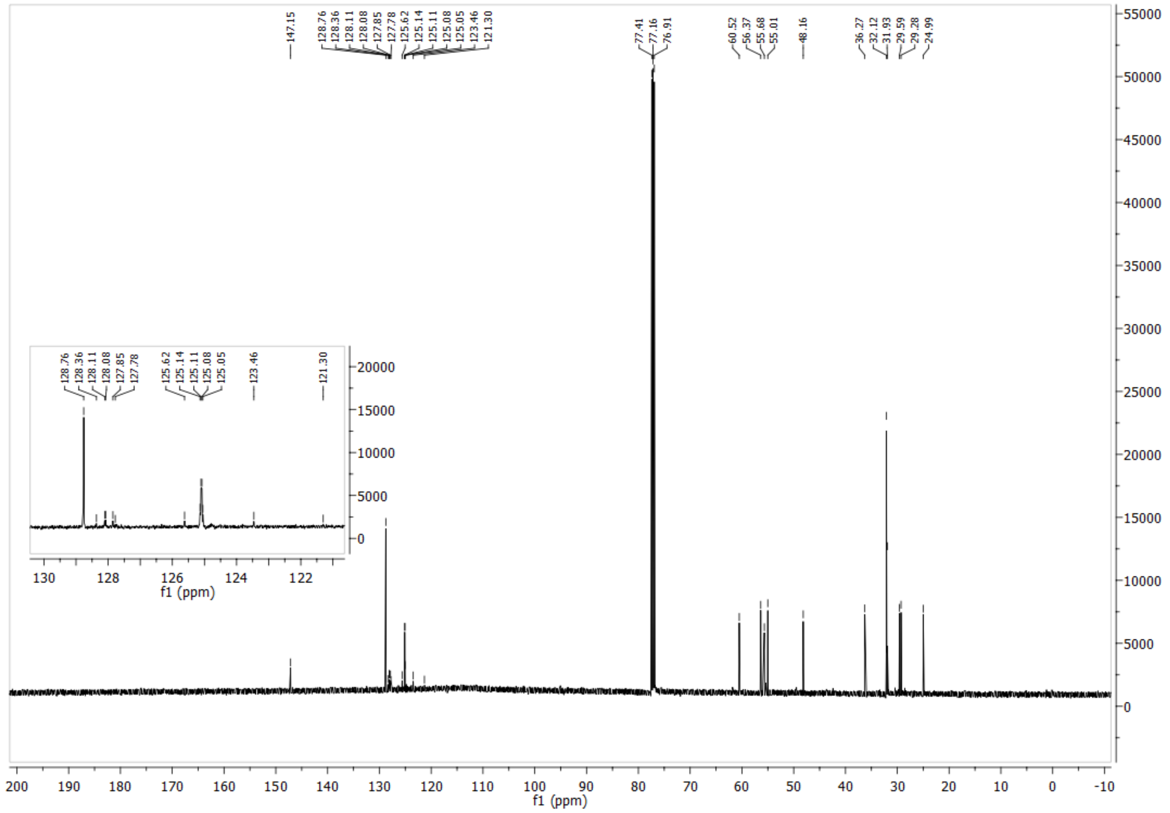

Figure S64: 10ic ^{13}C NMR, 126 MHz, CDCl$_3$

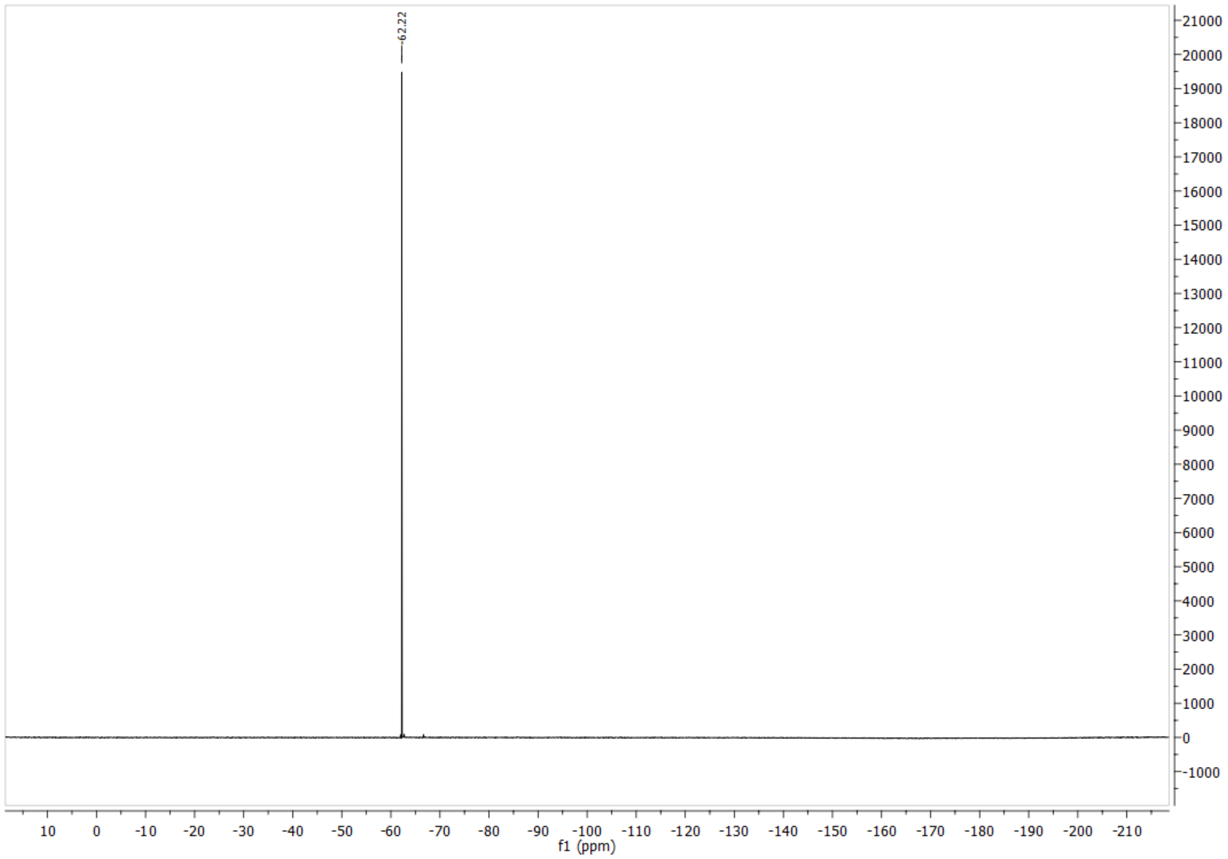

Figure S65: 10ic ^{19}F NMR, 376 MHz, $CDCl_3$

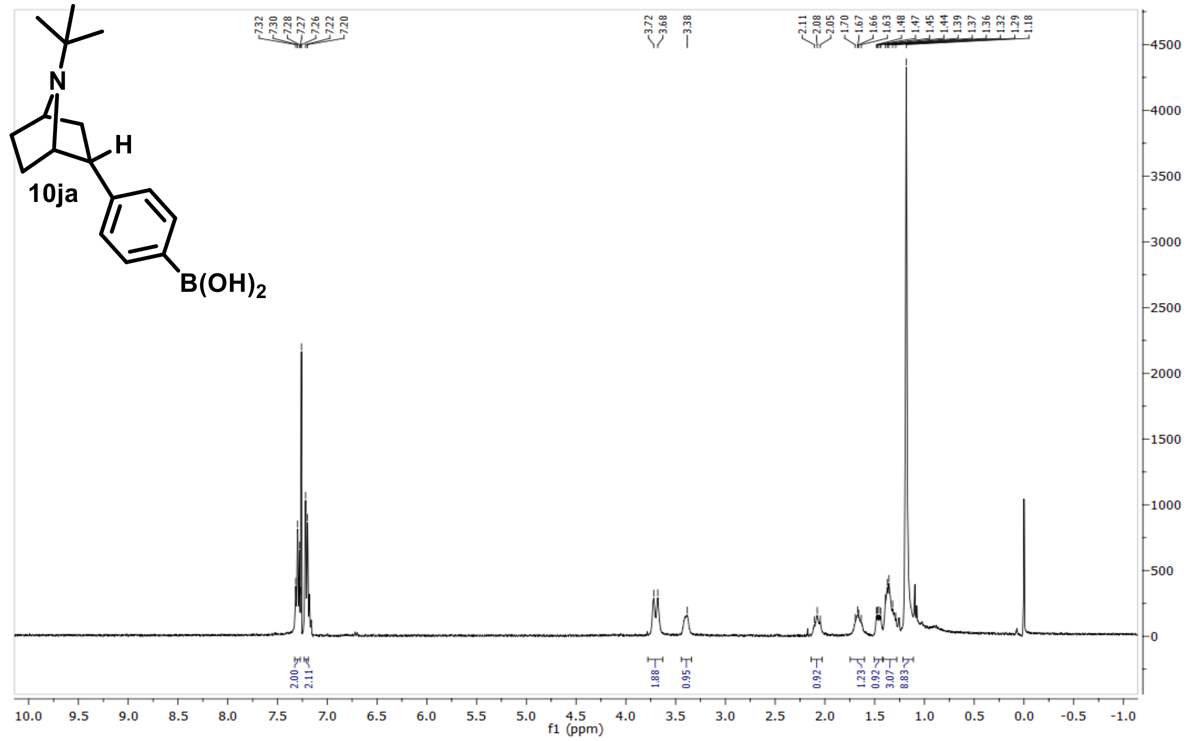

Figure S66: 10ja ^{1}H NMR, 400 MHz, $CDCl_3$

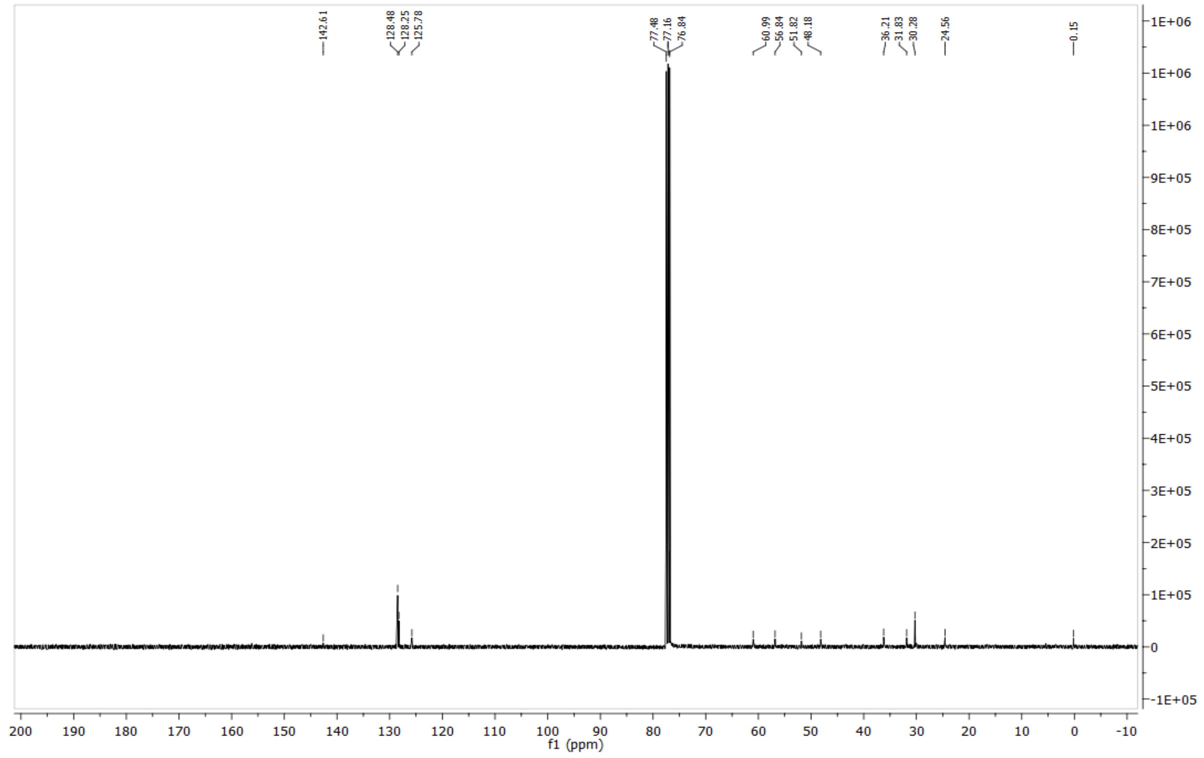

Figure S67: 10ja ^{13}C NMR, 126 MHz, $CDCl_3$

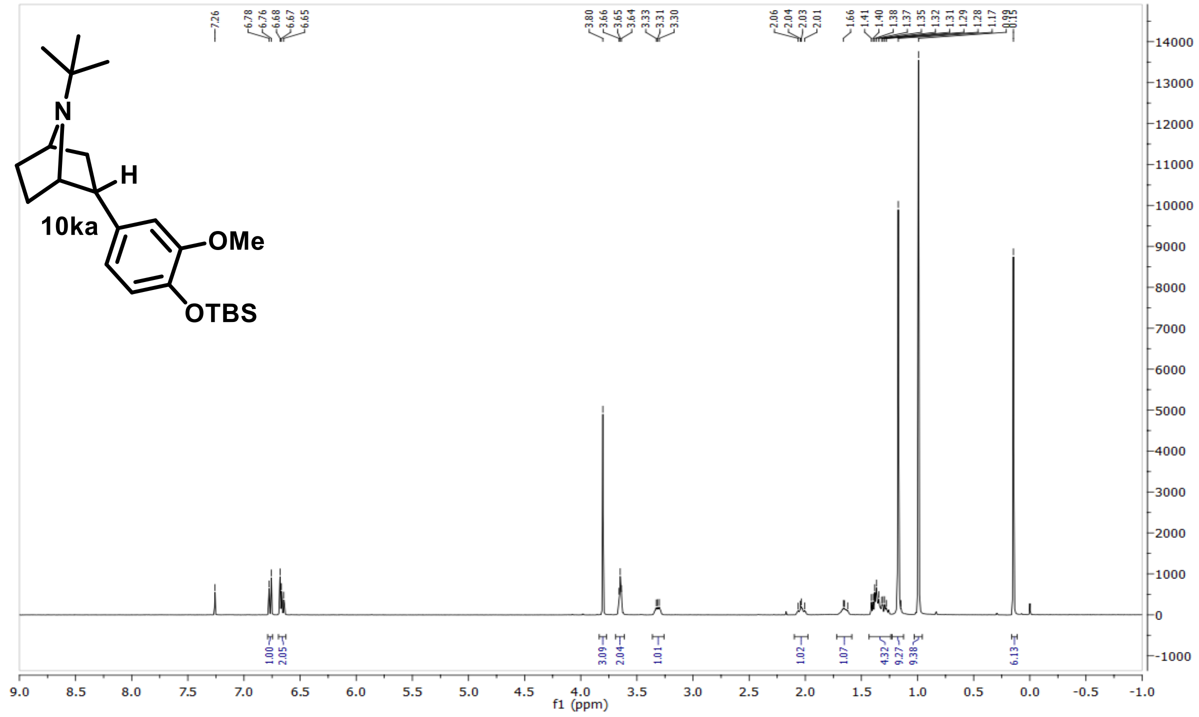

Figure S68: 10ka ^{1}H NMR, 400 MHz, $CDCl_3$

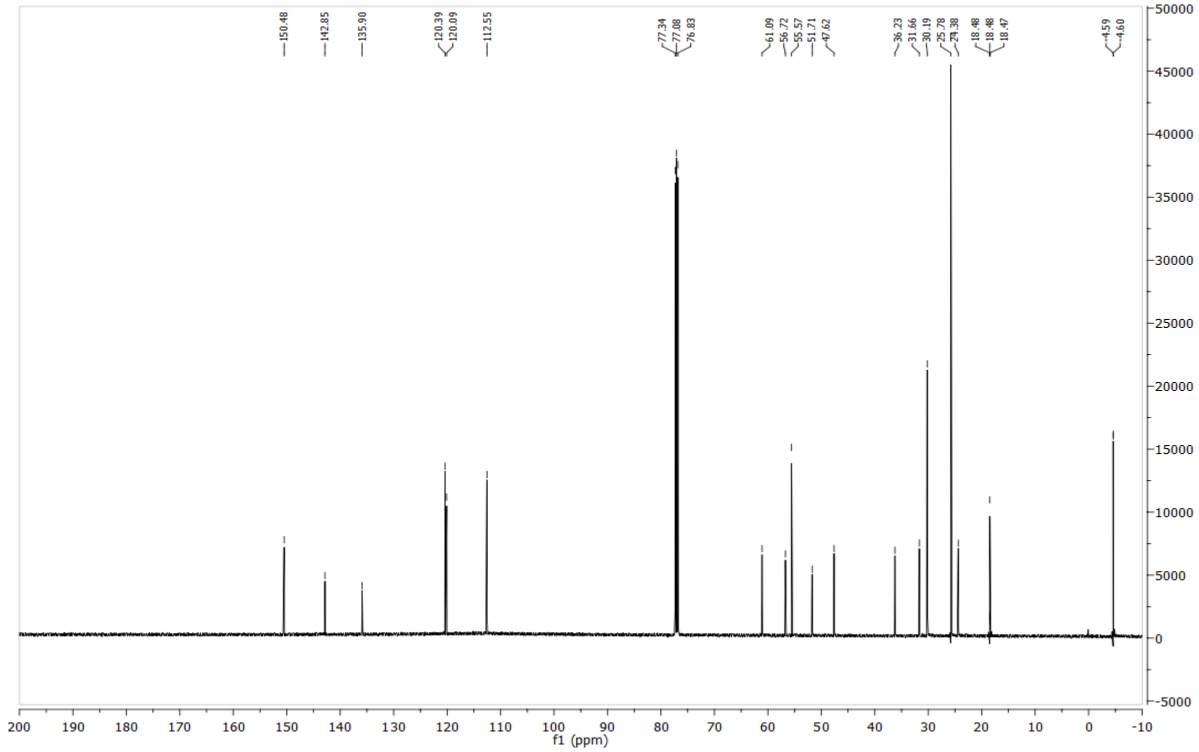

Figure S69: 10ka ^{13}C NMR, 126 MHz, $CDCl_3$

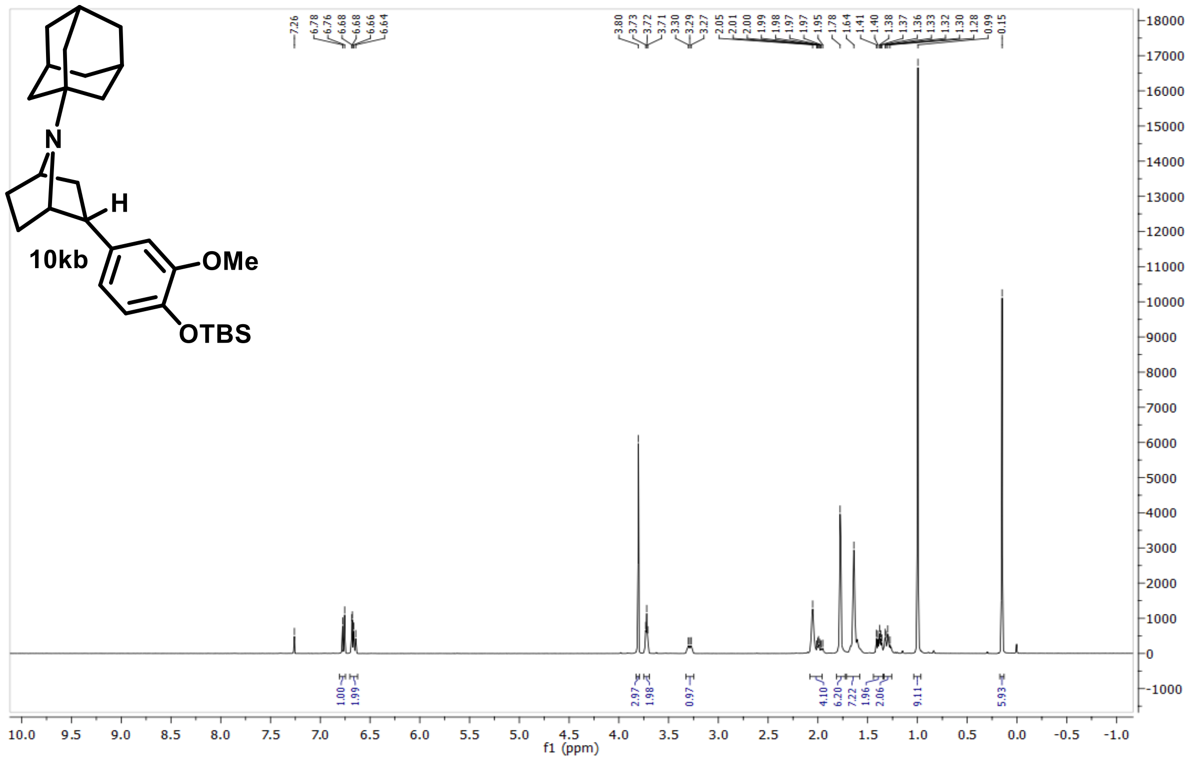

Figure S70: 10kb ^{1}H NMR, 400 MHz, $CDCl_3$

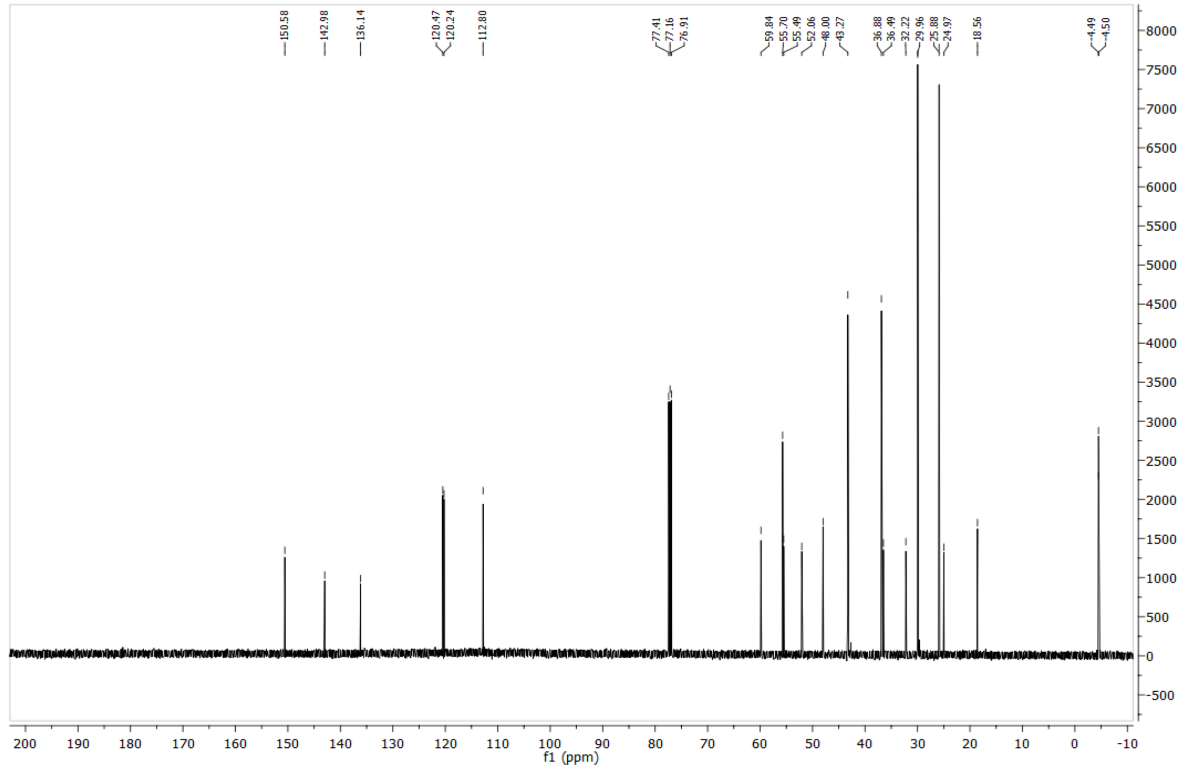

Figure S71: 10kb ^{13}C NMR, 126 MHz, $CDCl_3$

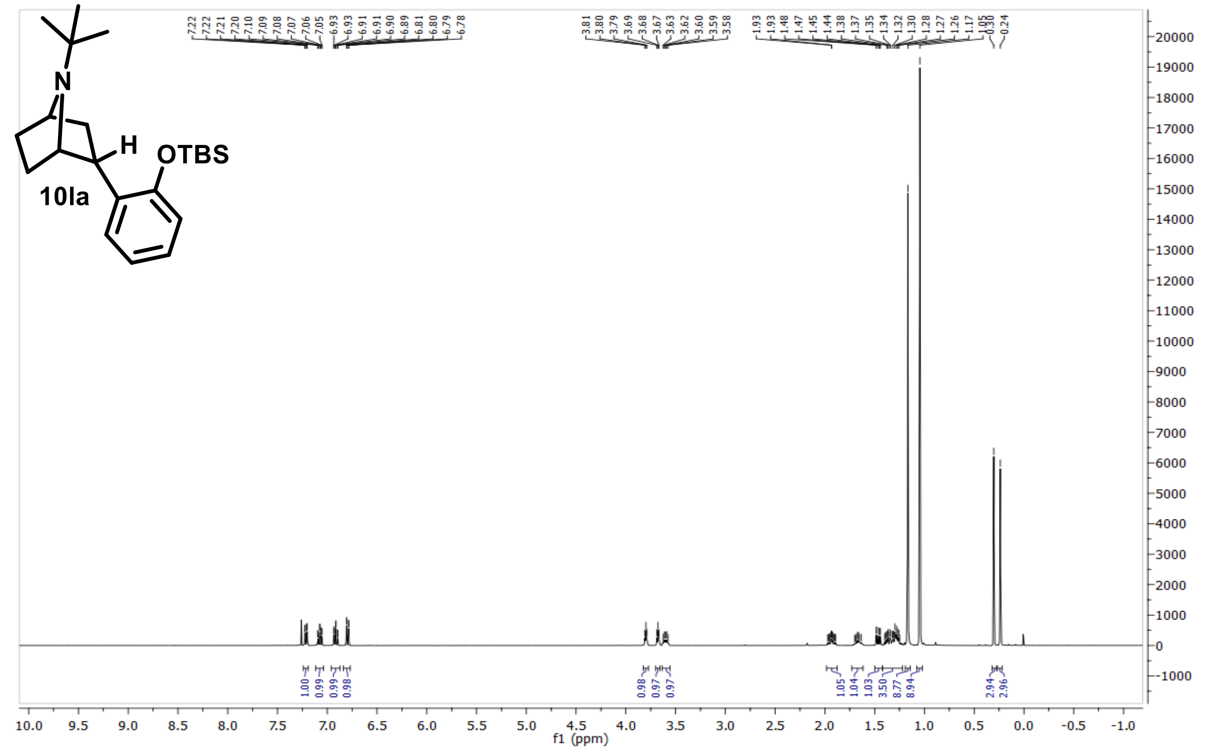

Figure S72: 10la ^{1}H NMR, 400 MHz, $CDCl_3$

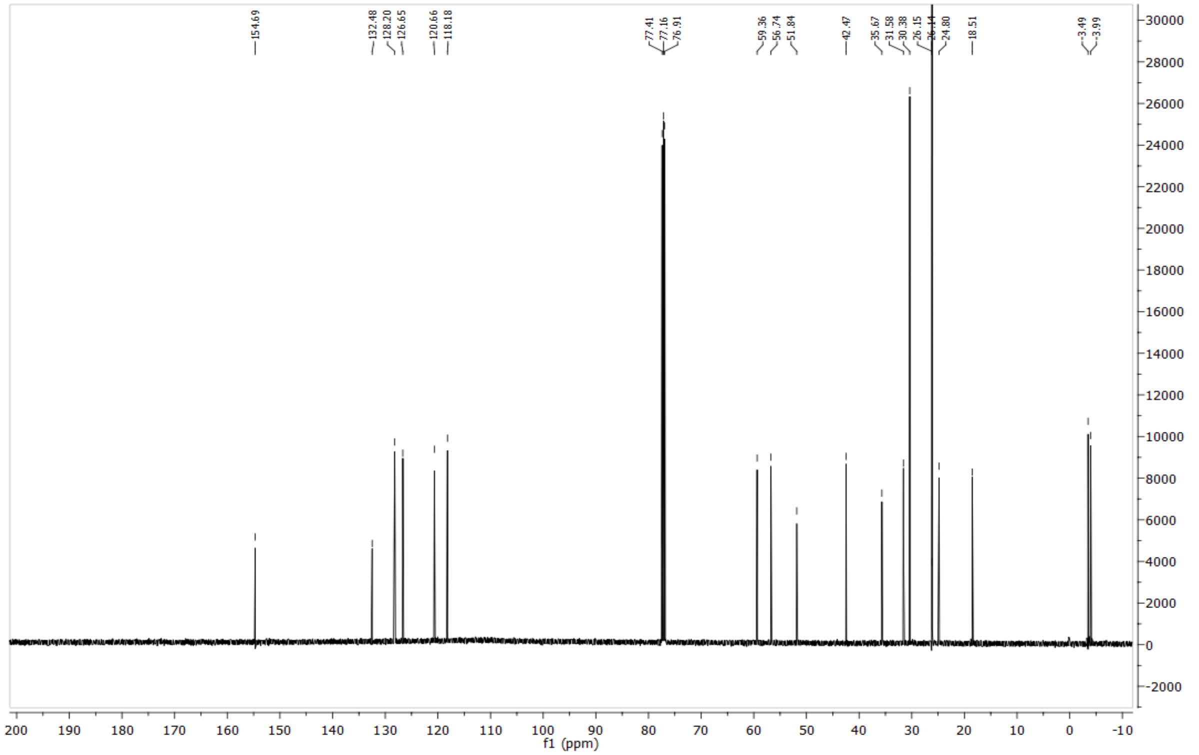

Figure S73: 10la ^{13}C NMR, 126 MHz, $CDCl_3$

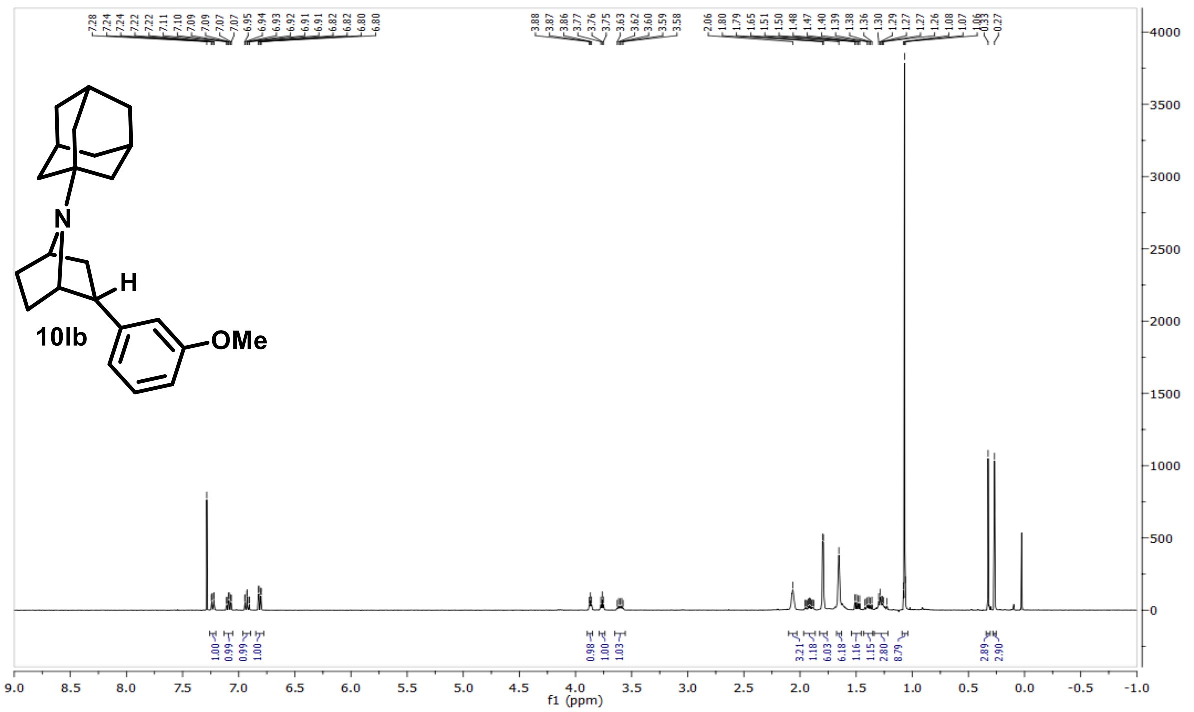

Figure S74: 10lb ^{1}H NMR, 400 MHz, $CDCl_3$

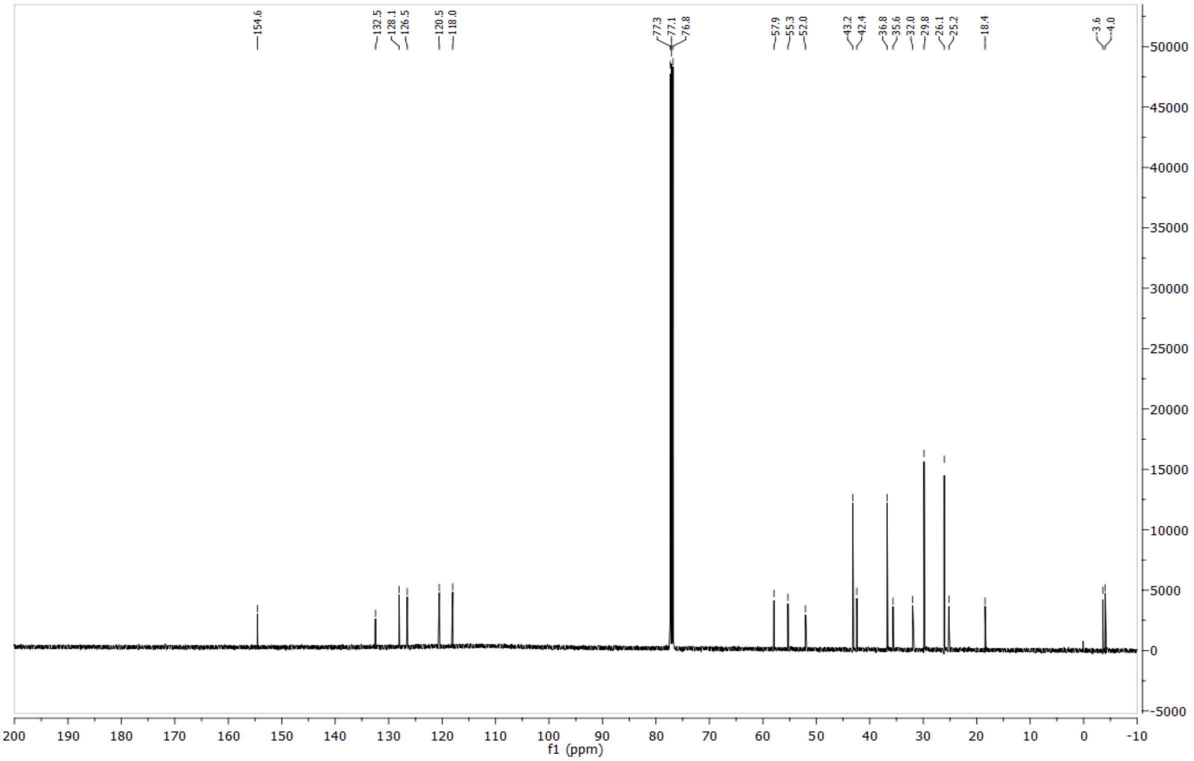

Figure S75: 10lb ^{13}C NMR, 126 MHz, $CDCl_3$

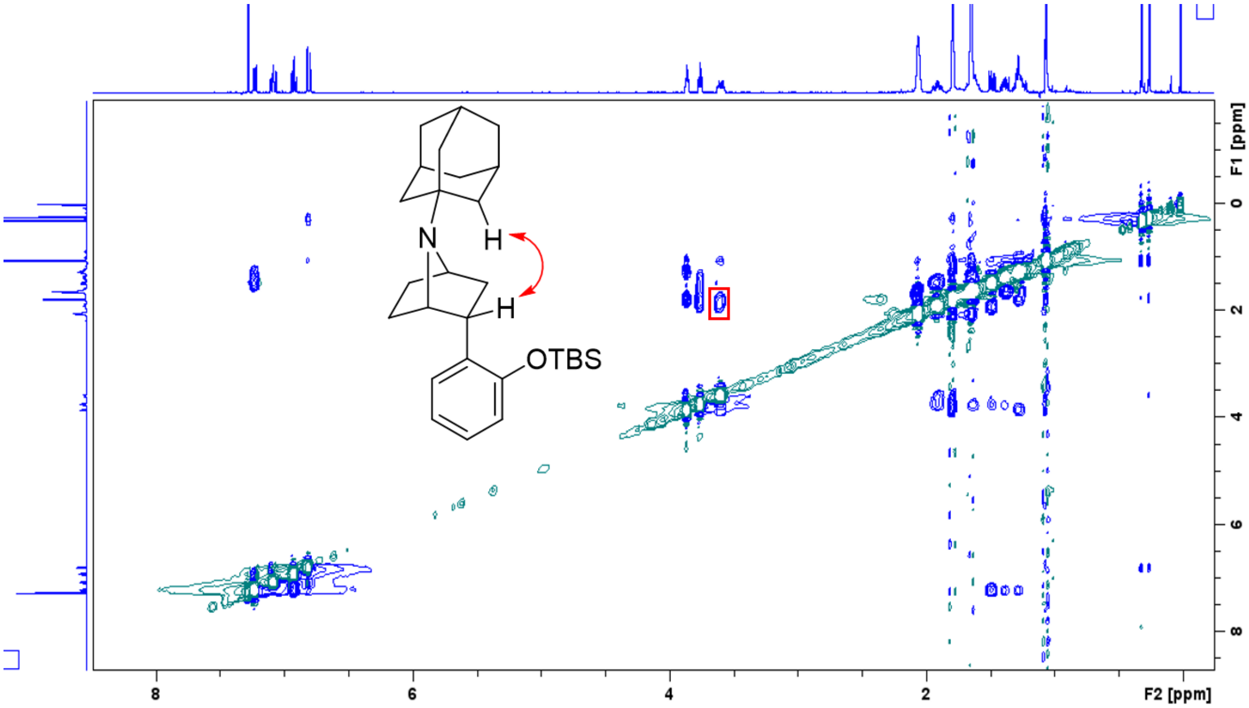

Figure S76: 10lb NOESY, 400 MHz, $CDCl_3$

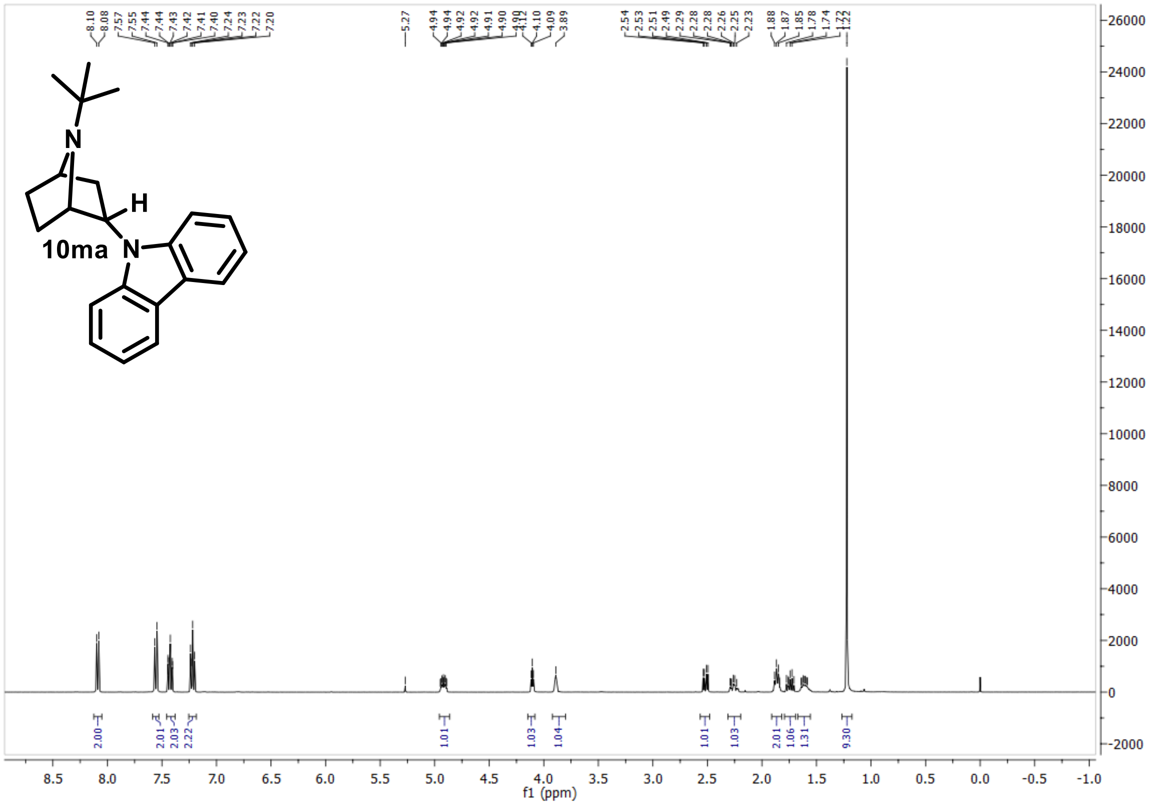

Figure S77: 10ma ^{1}H NMR, 400 MHz, $CDCl_3$

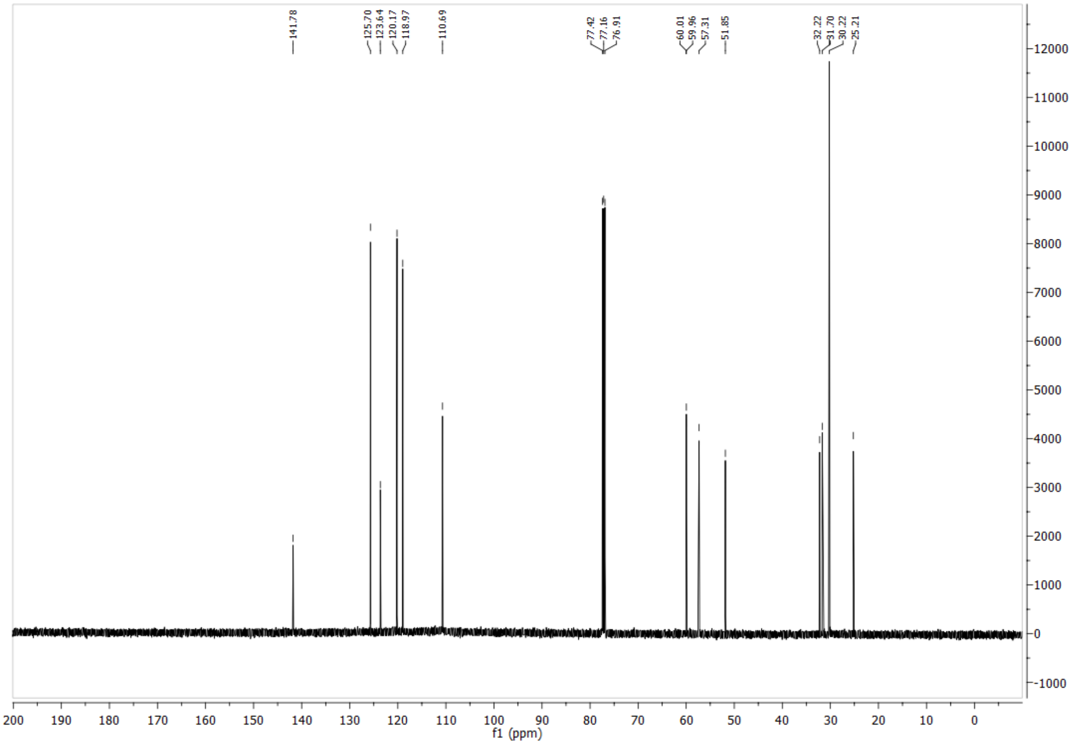

Figure S78: 10ma ^{13}C NMR, 126 MHz, $CDCl_3$

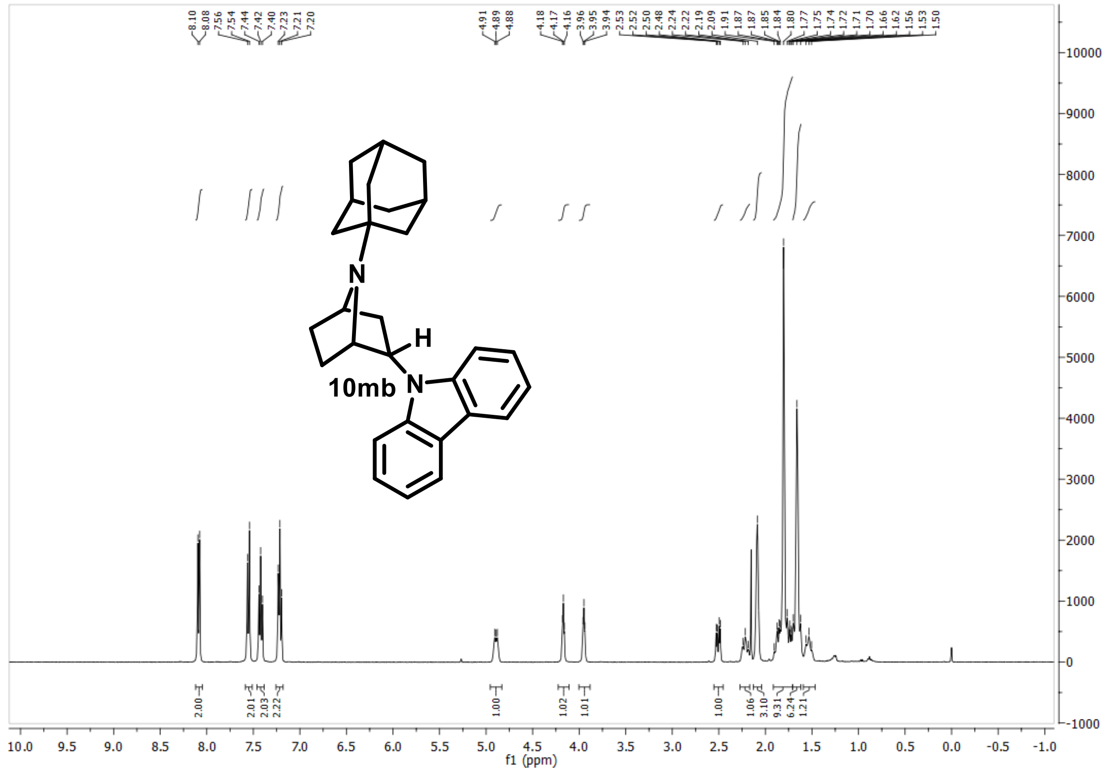

Figure S79: 10mb ^{1}H NMR, 400 MHz, $CDCl_3$

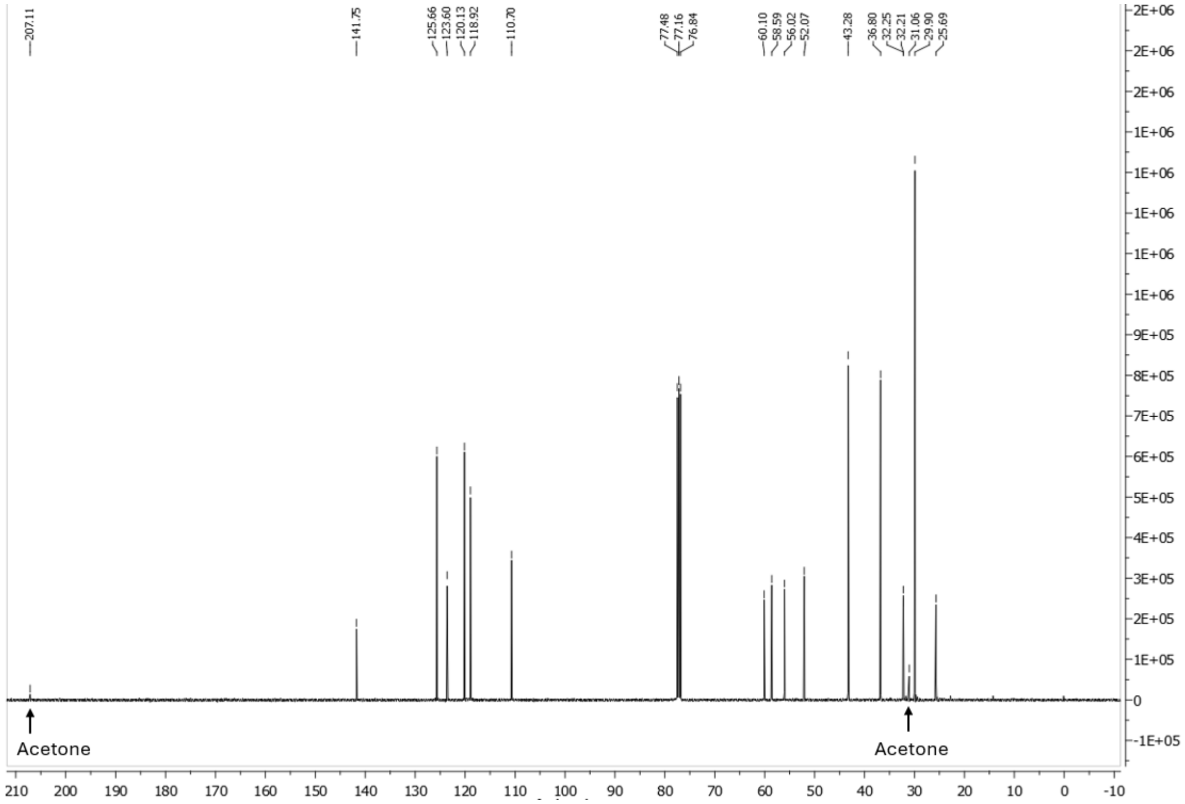

Figure S80: 10mb ^{13}C NMR, 101 MHz, $CDCl_3$

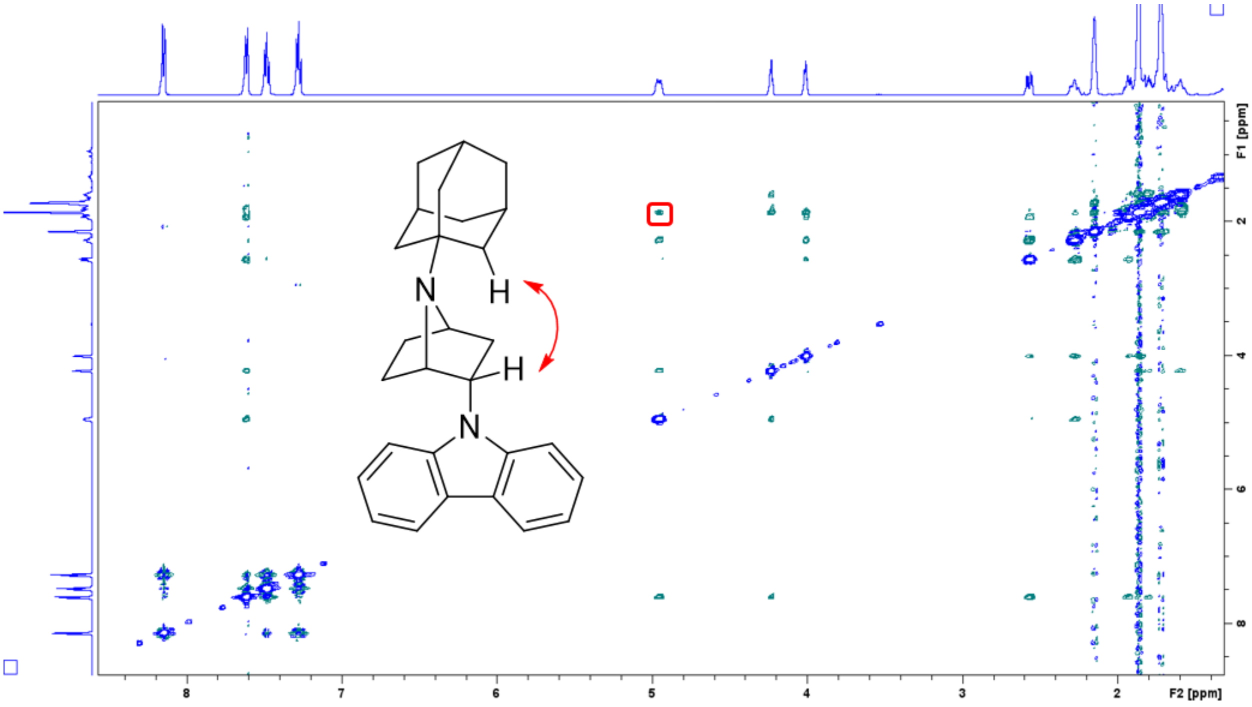

Figure S81: 10mb NOESY, 500 MHz, $CDCl_3$

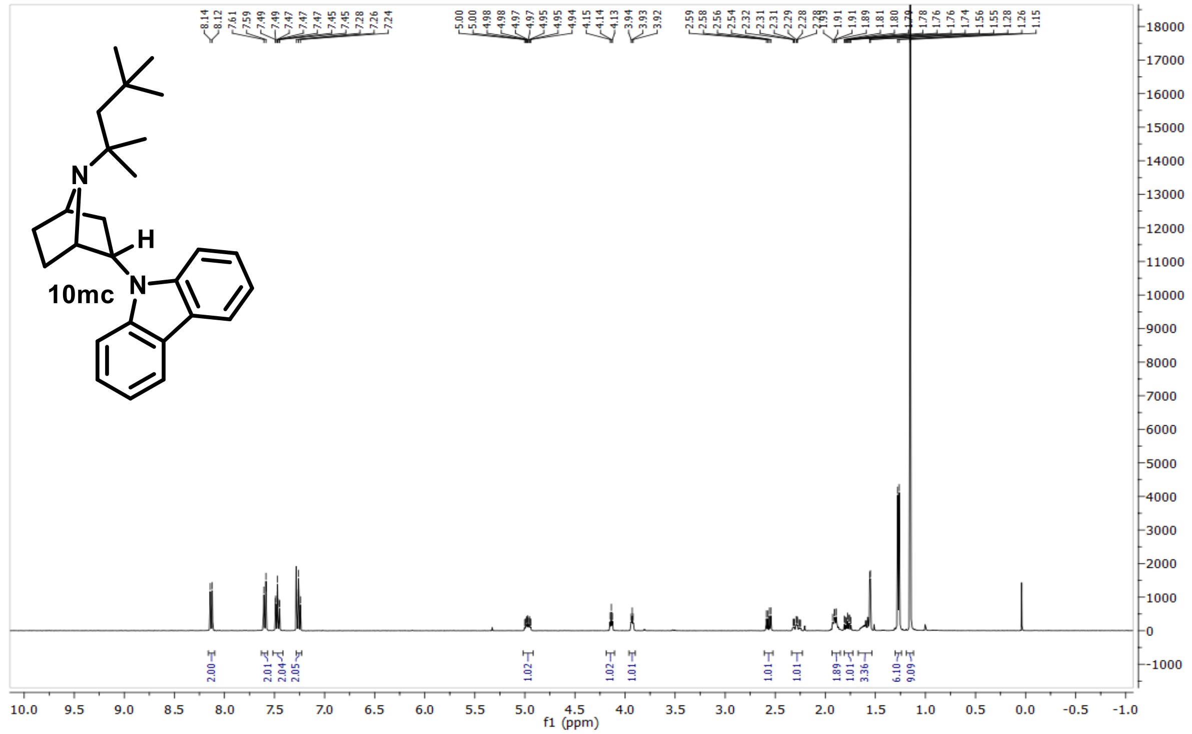

Figure S82: 10mc ^{1}H NMR, 400 MHz, $CDCl_3$

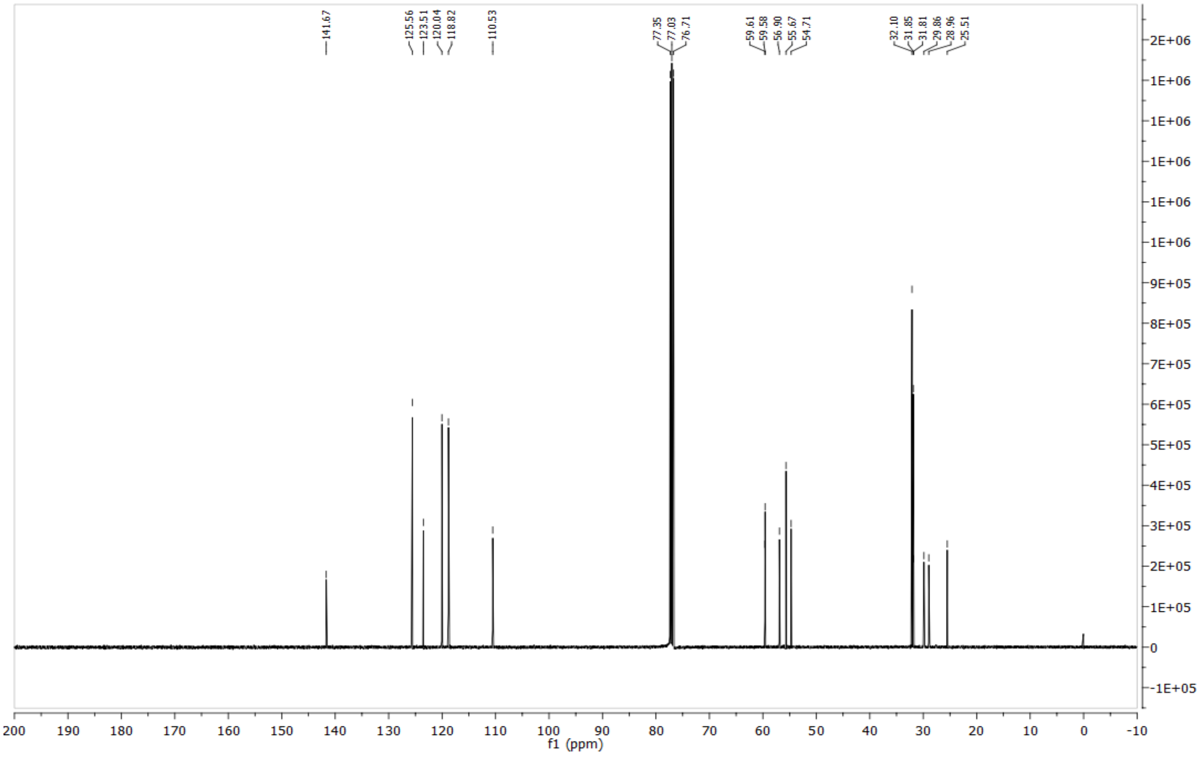

Figure S83: 10mc ^{13}C NMR, 126 MHz, $CDCl_3$

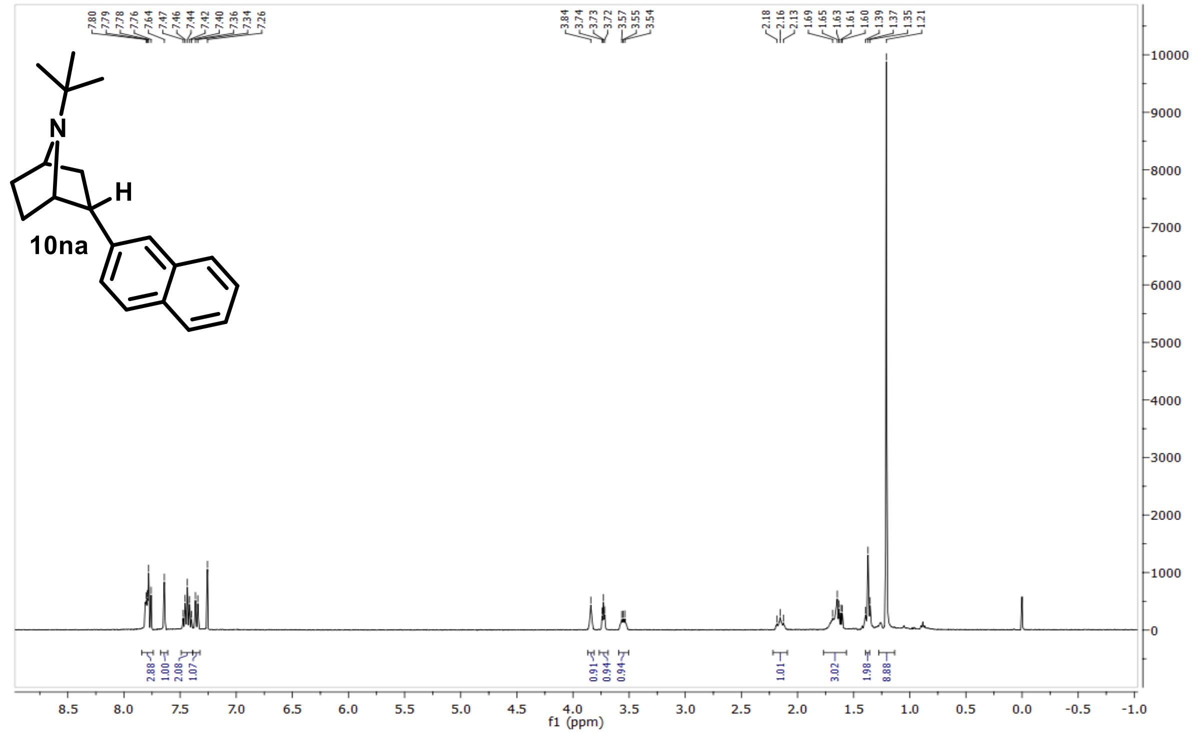

Figure S84: 10na ^{1}H NMR, 400 MHz, $CDCl_3$

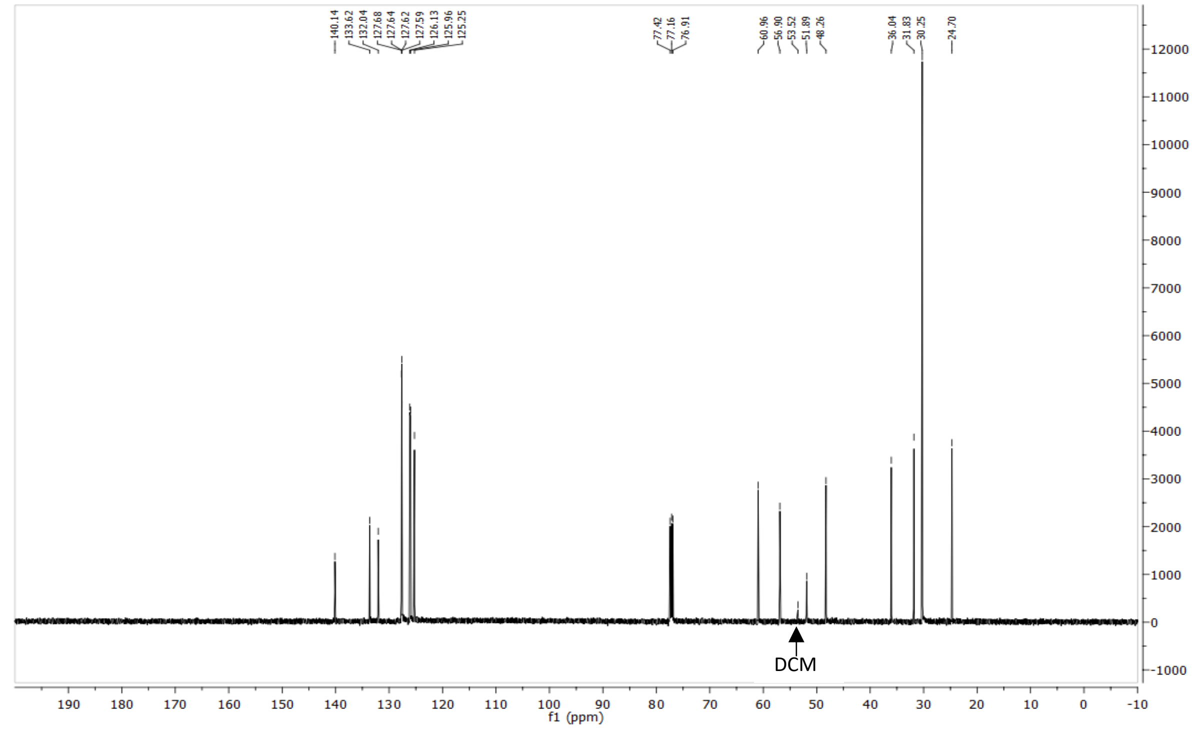

Figure S85: 10na ^{13}C NMR, 126 MHz, $CDCl_3$

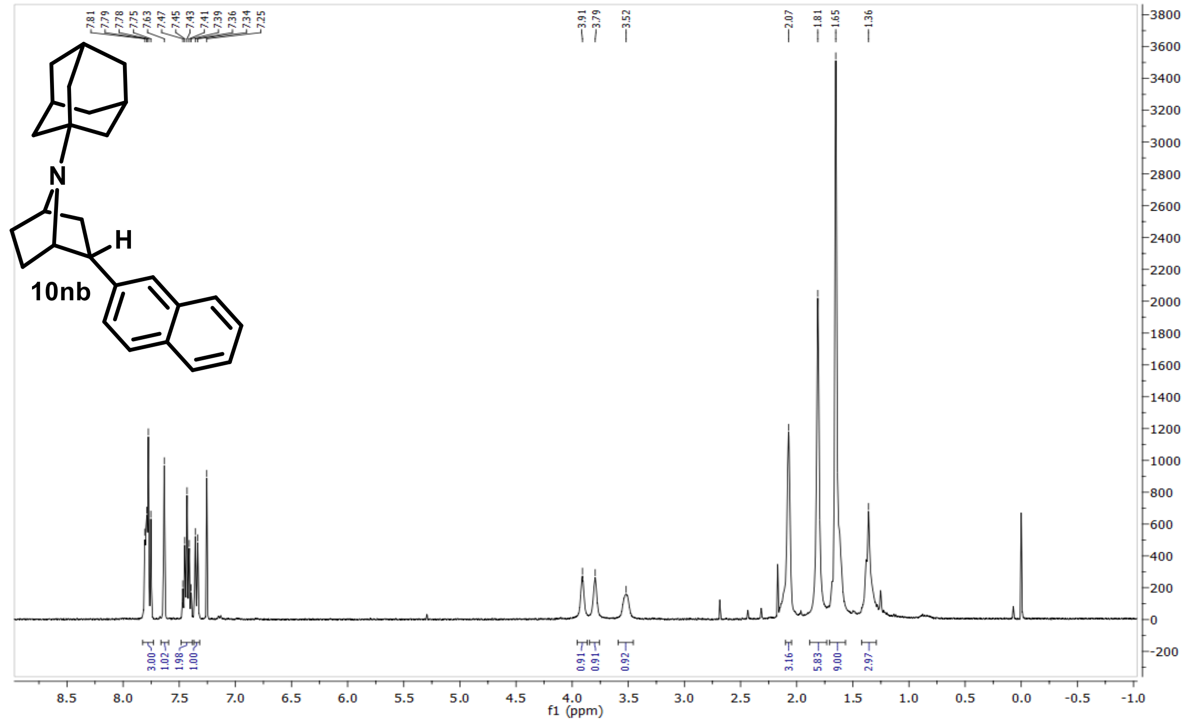

Figure S86: 10nb ^{1}H NMR, 400 MHz, $CDCl_3$

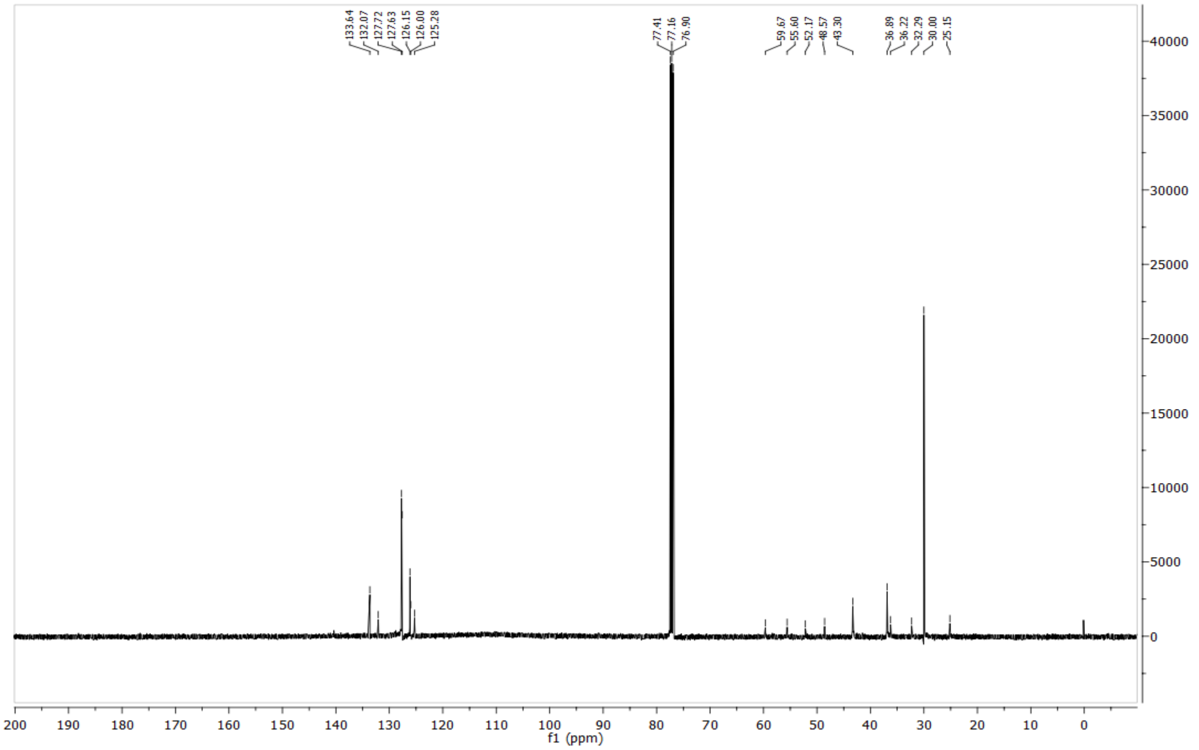

Figure S87: 10nb ^{13}C NMR, 126 MHz, $CDCl_3$

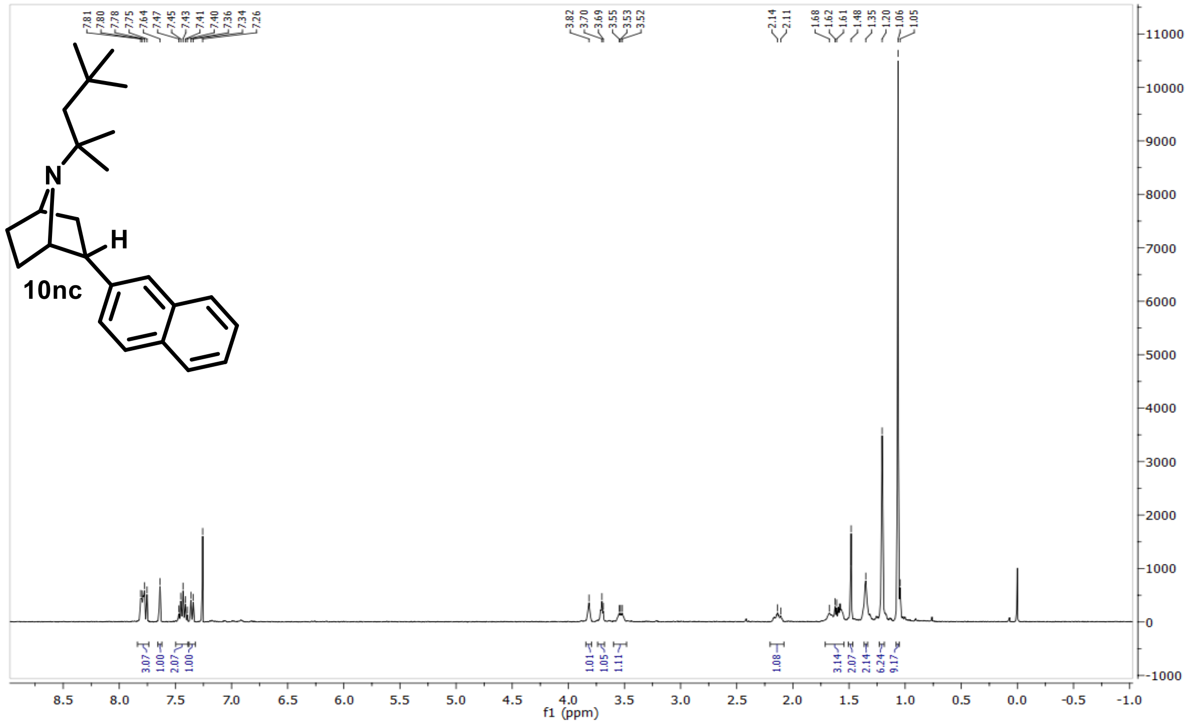

Figure S88: 10nc ^{1}H NMR, 400 MHz, $CDCl_3$

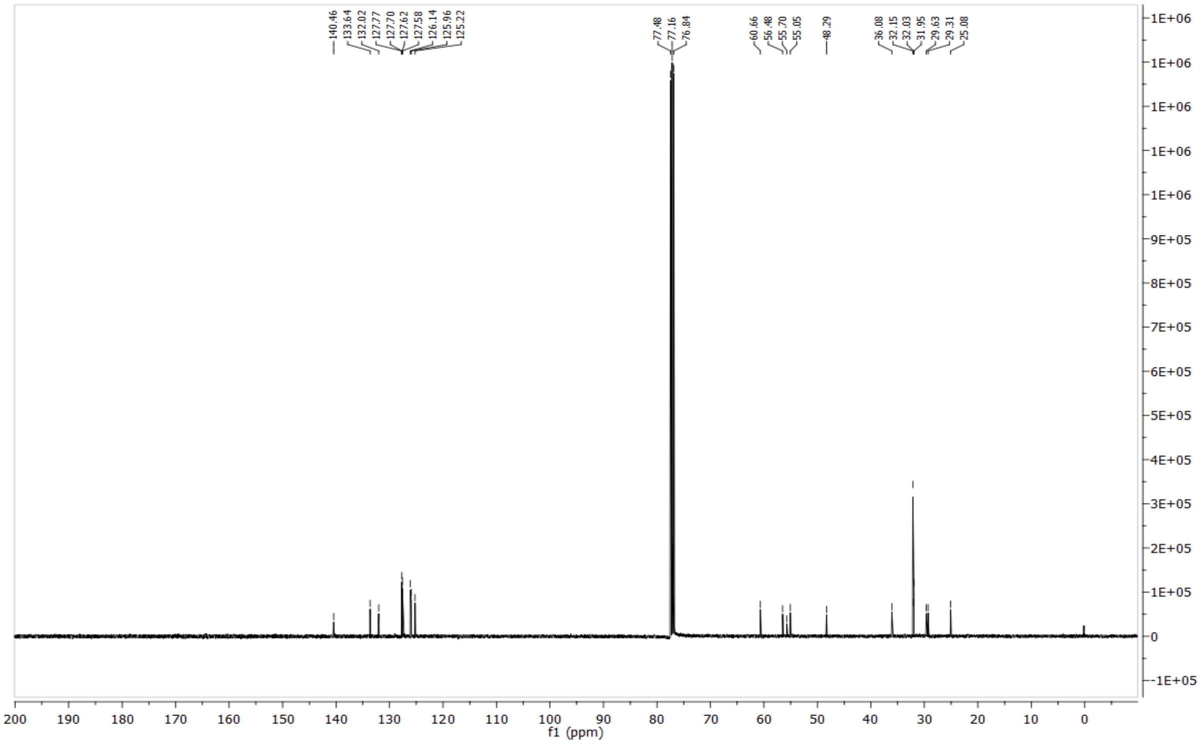

Figure S89: 10nc ^{13}C NMR, 126 MHz, $CDCl_3$

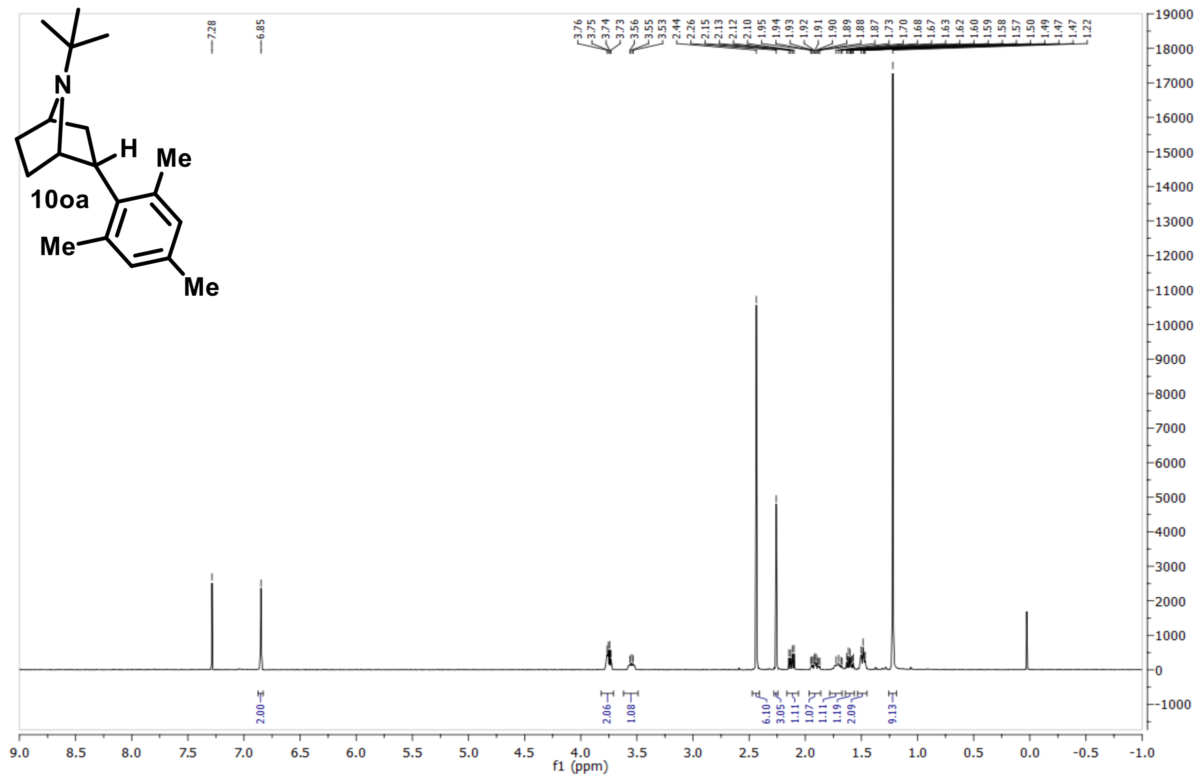

Figure S90: 10oa ^{1}H NMR, 400 MHz, CDCl$_3$

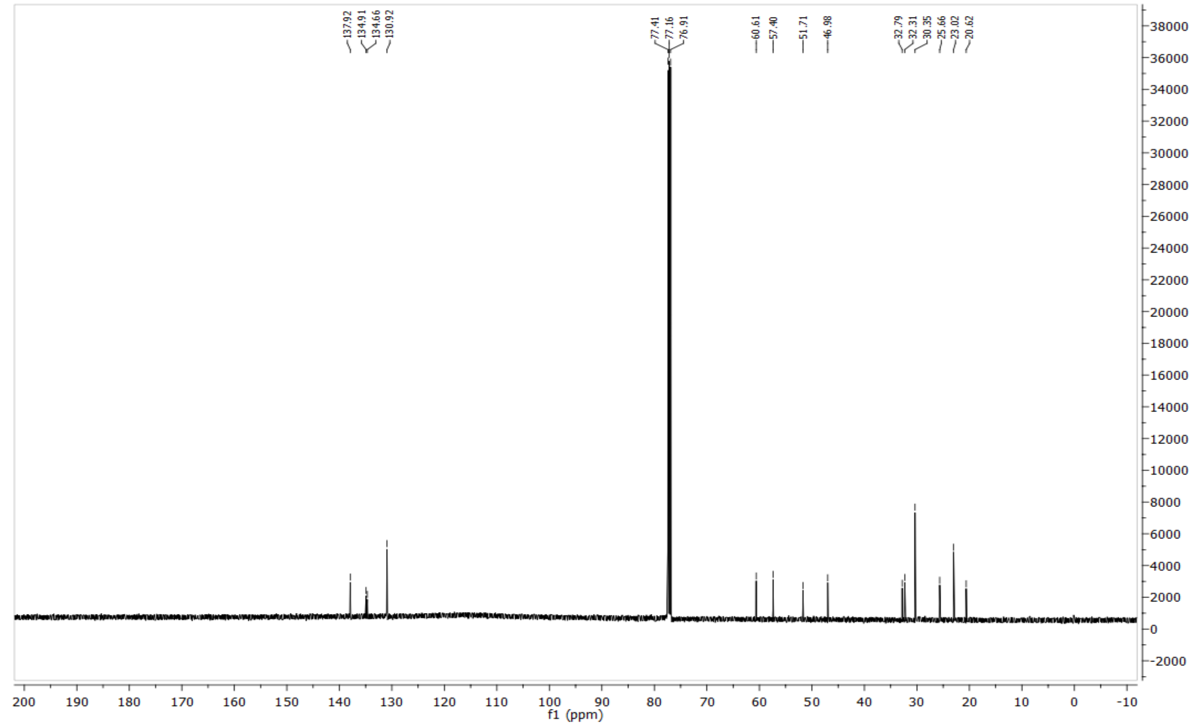

Figure S91: 10oa ^{13}C NMR, 126 MHz, CDCl$_3$

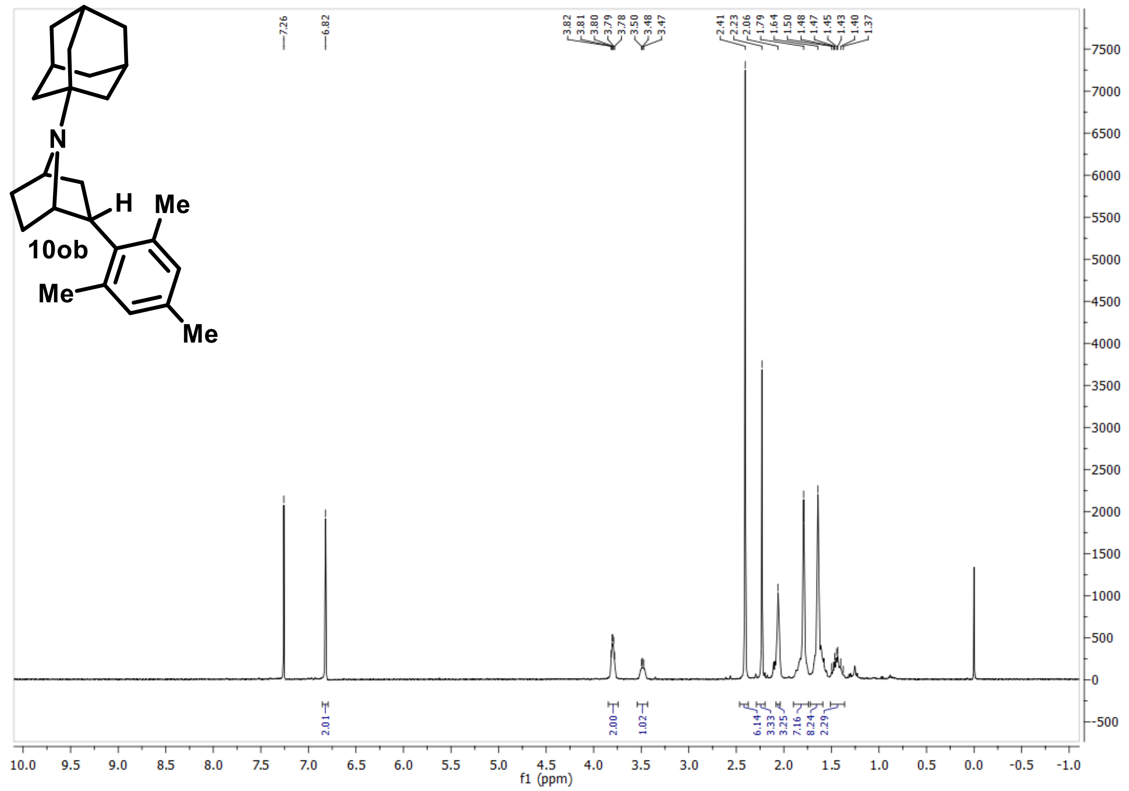

Figure S92: 10ob ^{1}H NMR, 400 MHz, $CDCl_3$

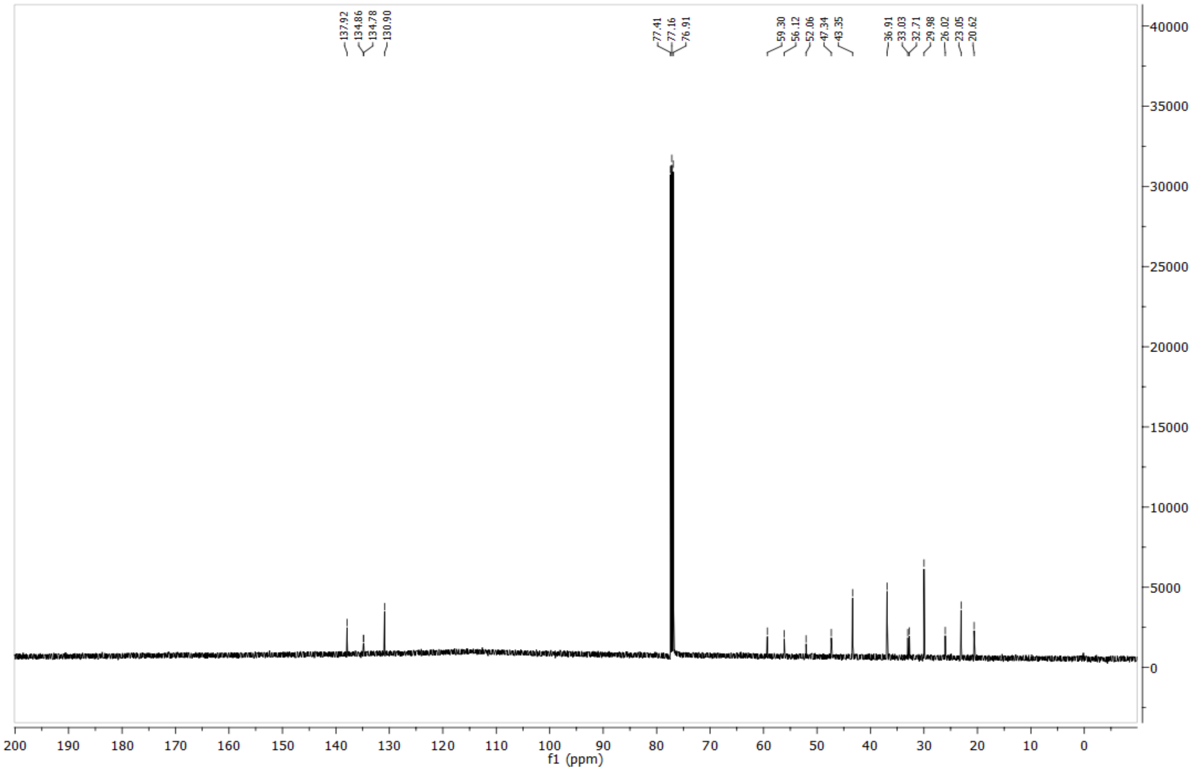

Figure S93: 10ob ^{13}C NMR, 126 MHz, $CDCl_3$

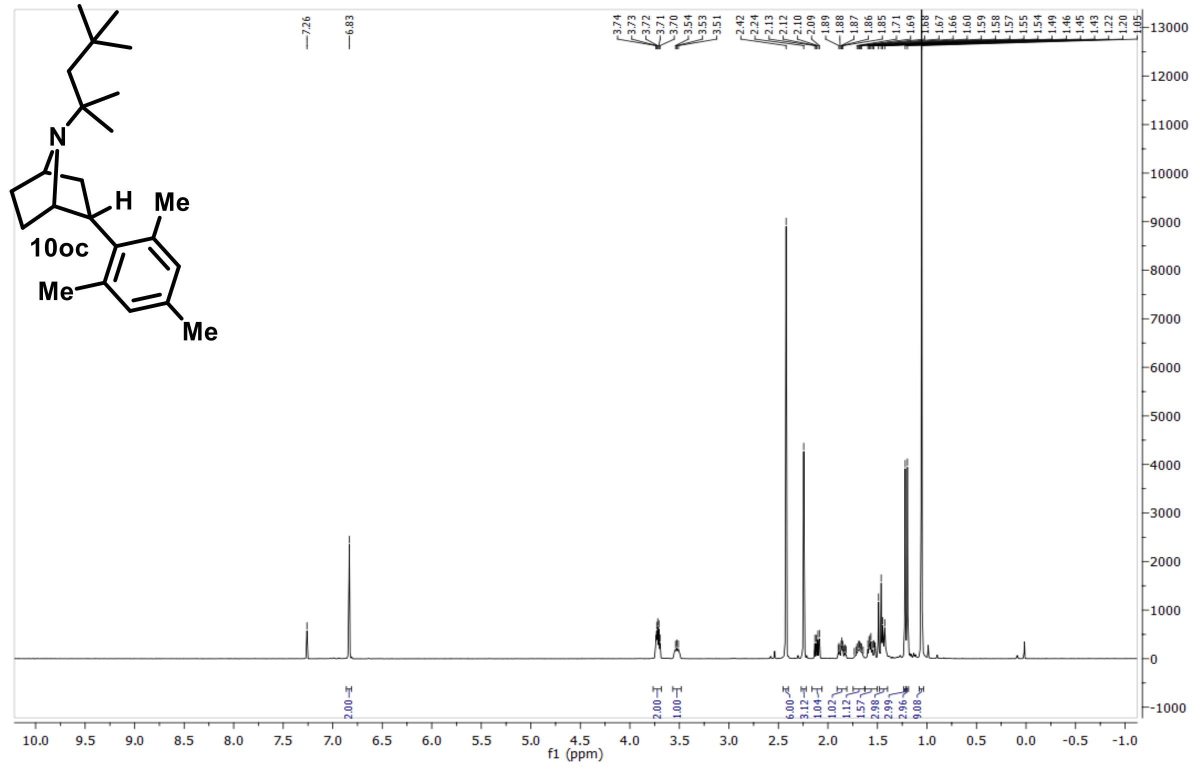

Figure S94: 10oc ^{1}H NMR, 400 MHz, $CDCl_3$

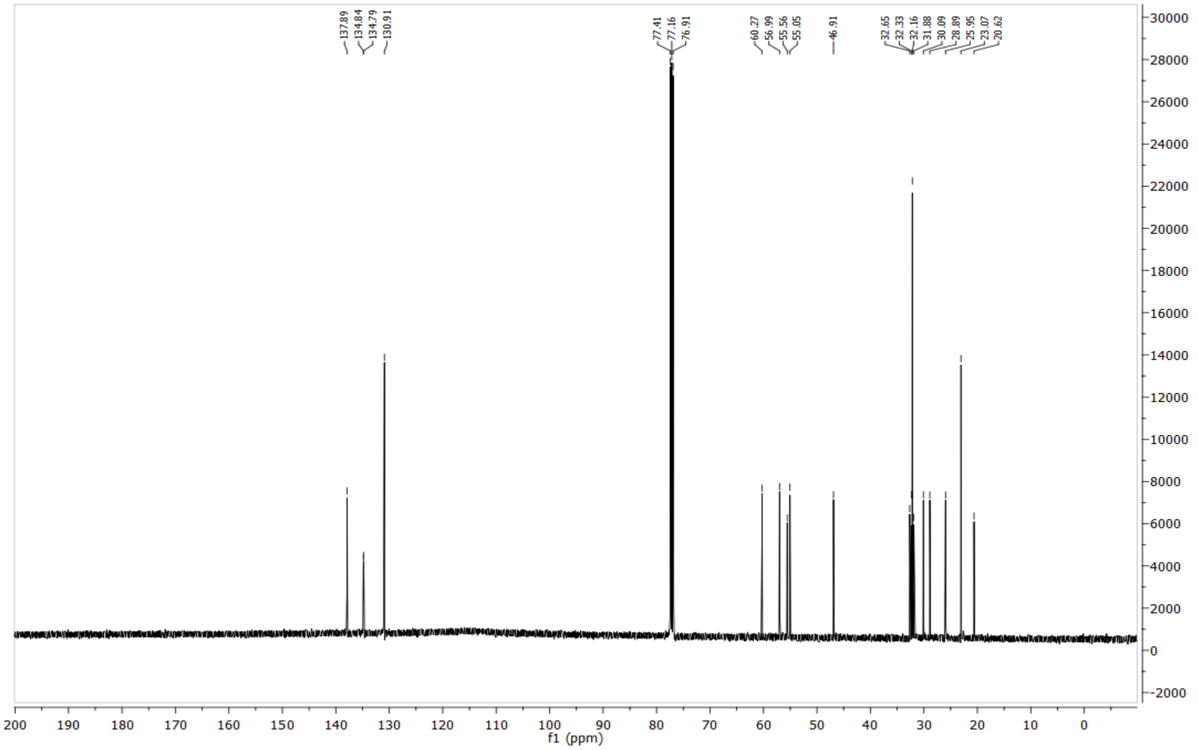

Figure S95: 10oc ^{13}C NMR, 126 MHz, $CDCl_3$

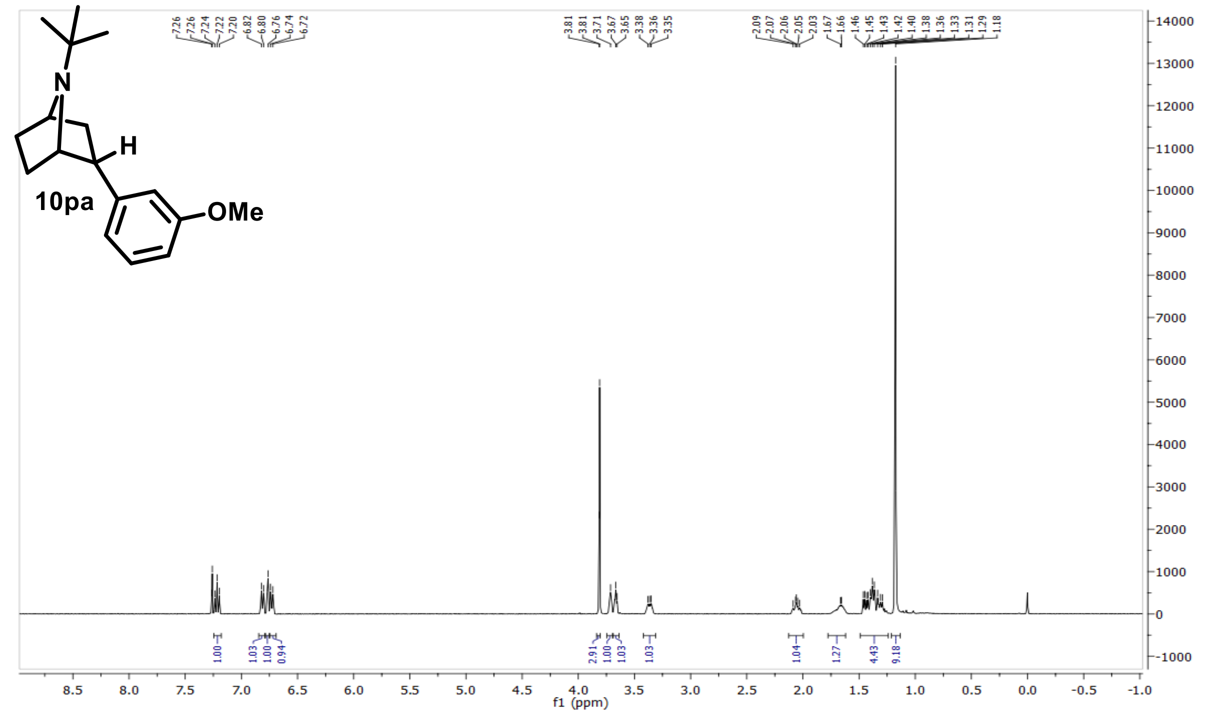

Figure S96: 10pa ^{1}H NMR, 400 MHz, $CDCl_3$

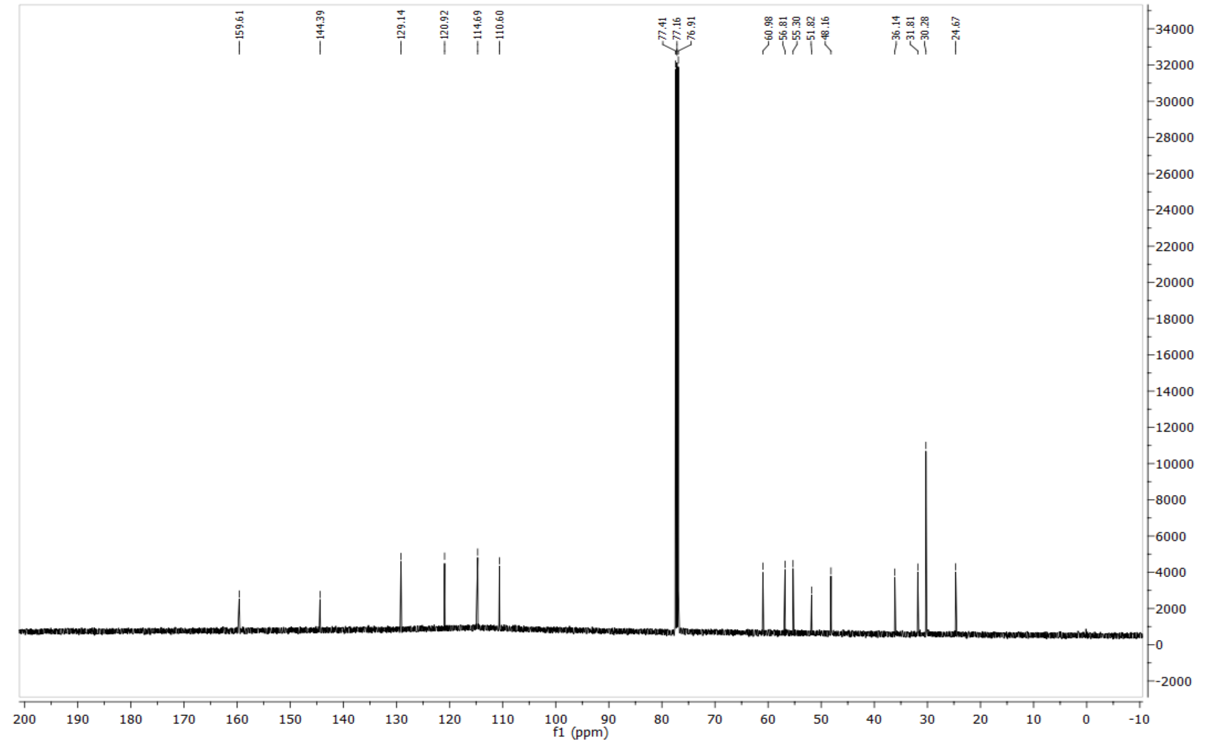

Figure S97: 10pa ^{13}C NMR, 126 MHz, $CDCl_3$

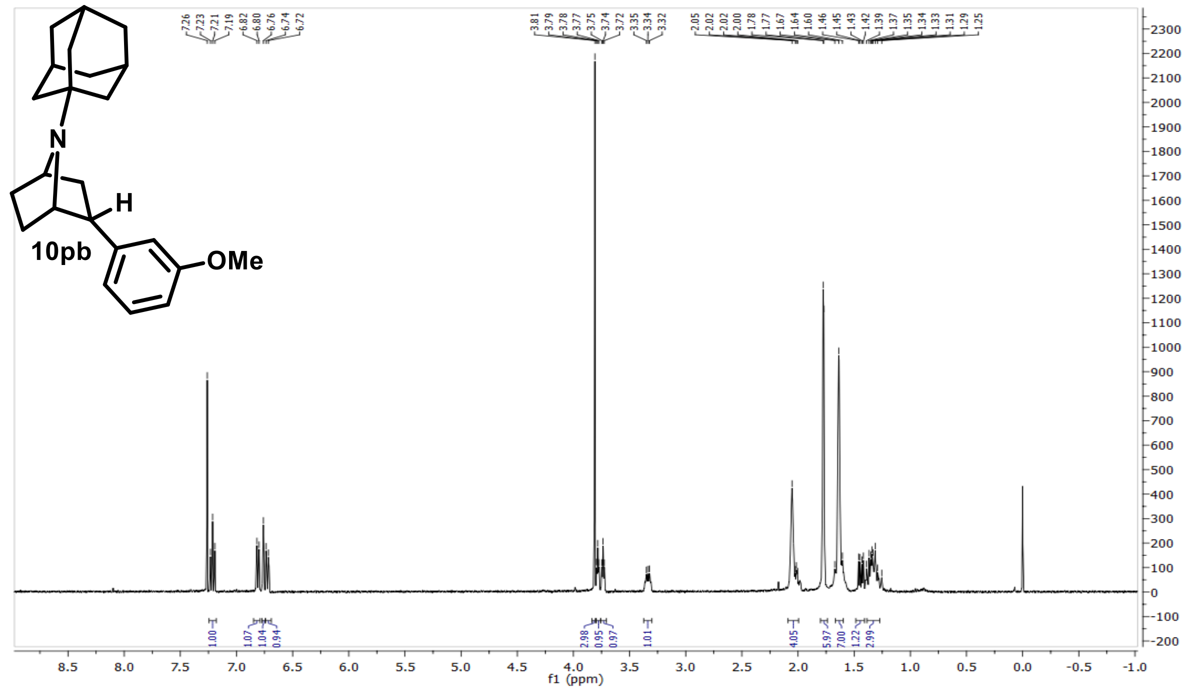

Figure S98: 10pb ^{1}H NMR, 400 MHz, $CDCl_3$

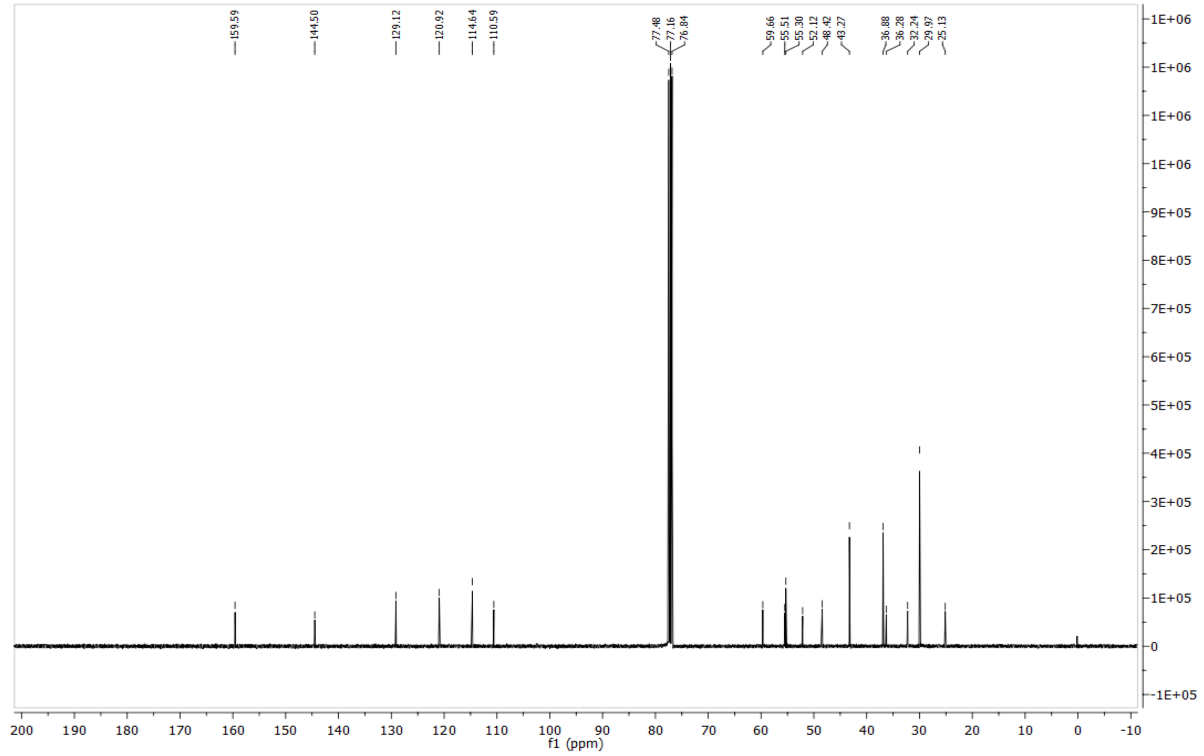

Figure S99: 10pb ^{13}C NMR, 101 MHz, $CDCl_3$

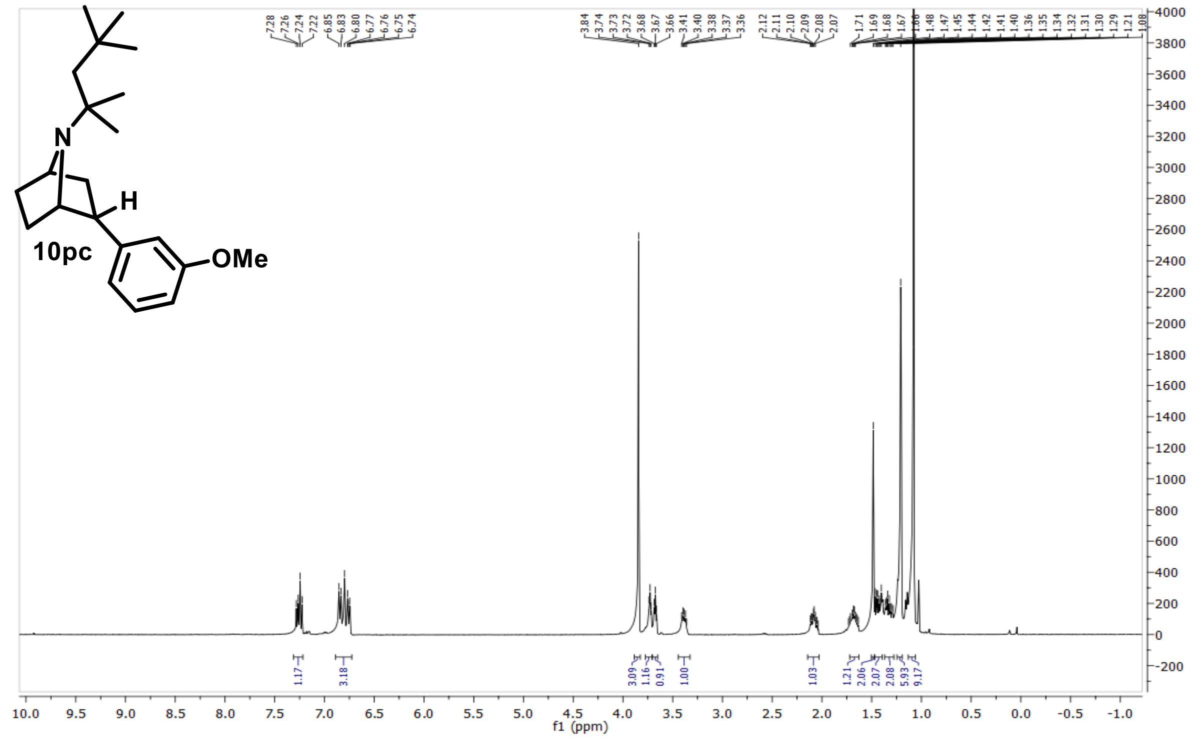

Figure S100: 10pc ^{1}H NMR, 400 MHz, $CDCl_3$

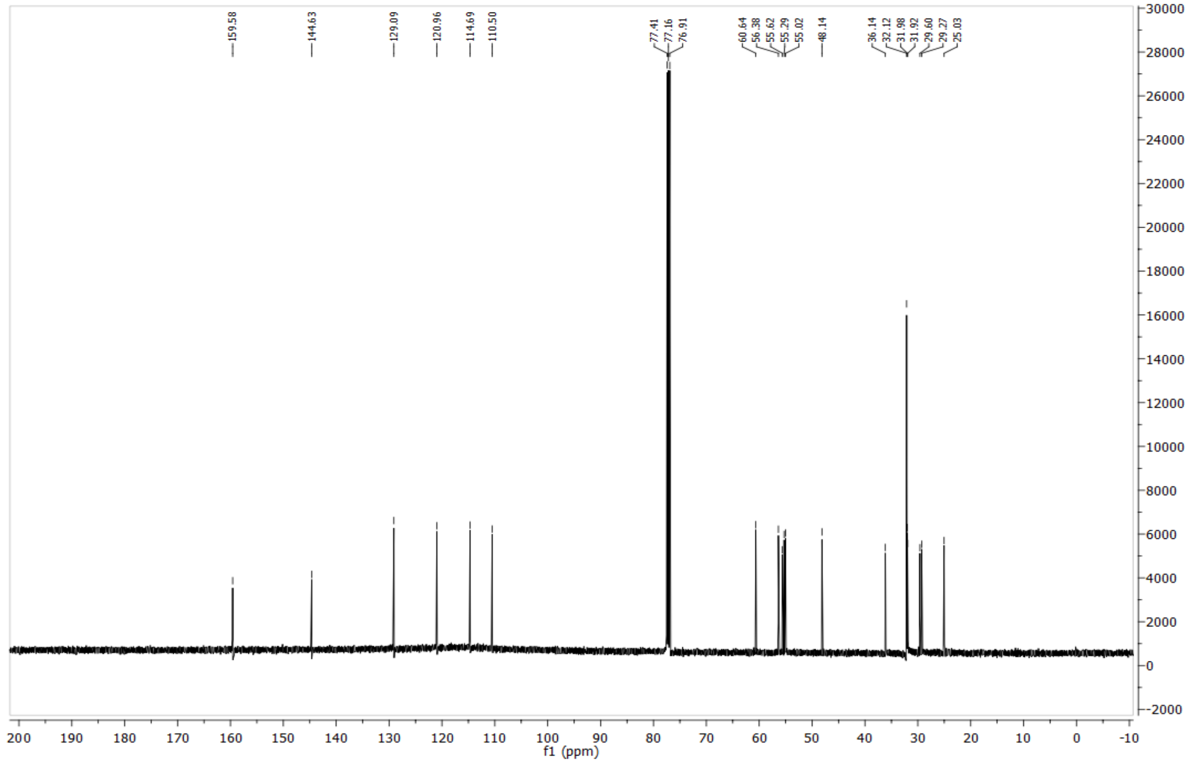

Figure S101: 10pc ^{13}C NMR, 126 MHz, $CDCl_3$

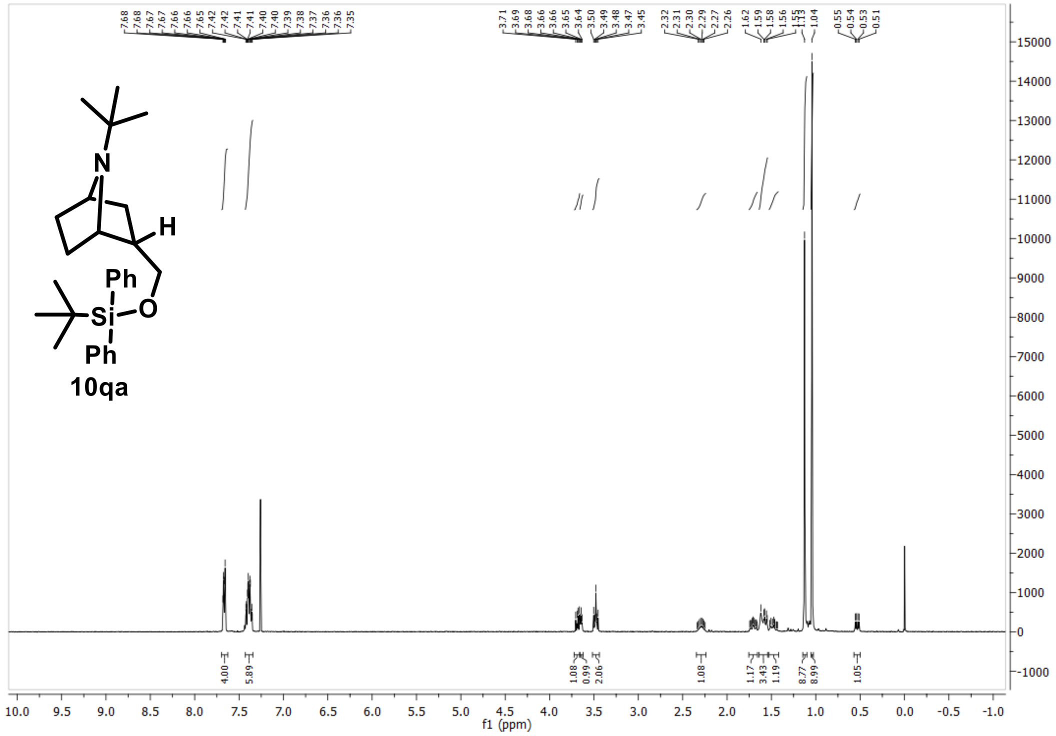

Figure S102: 10qa ^{1}H NMR, 400 MHz, $CDCl_3$

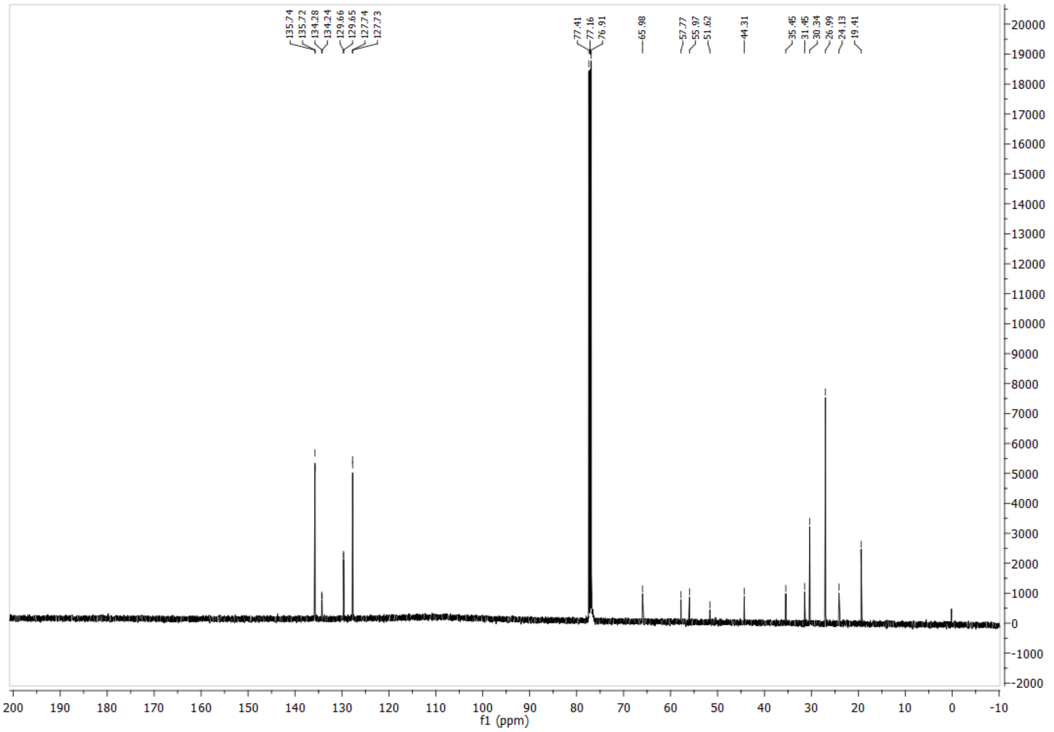

Figure S103: 10qa ^{13}C NMR, 125 MHz, $CDCl_3$

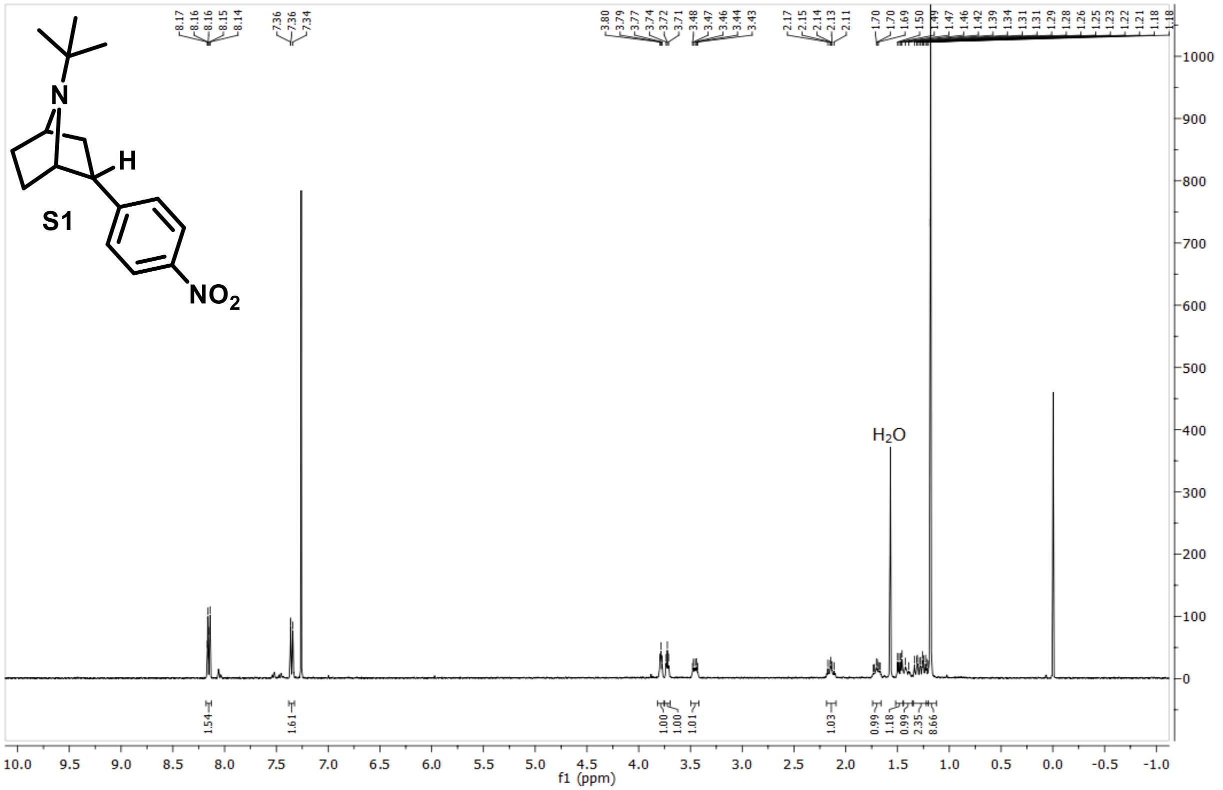

Figure S104: S1 ^{1}H NMR, 400 MHz, $CDCl_3$

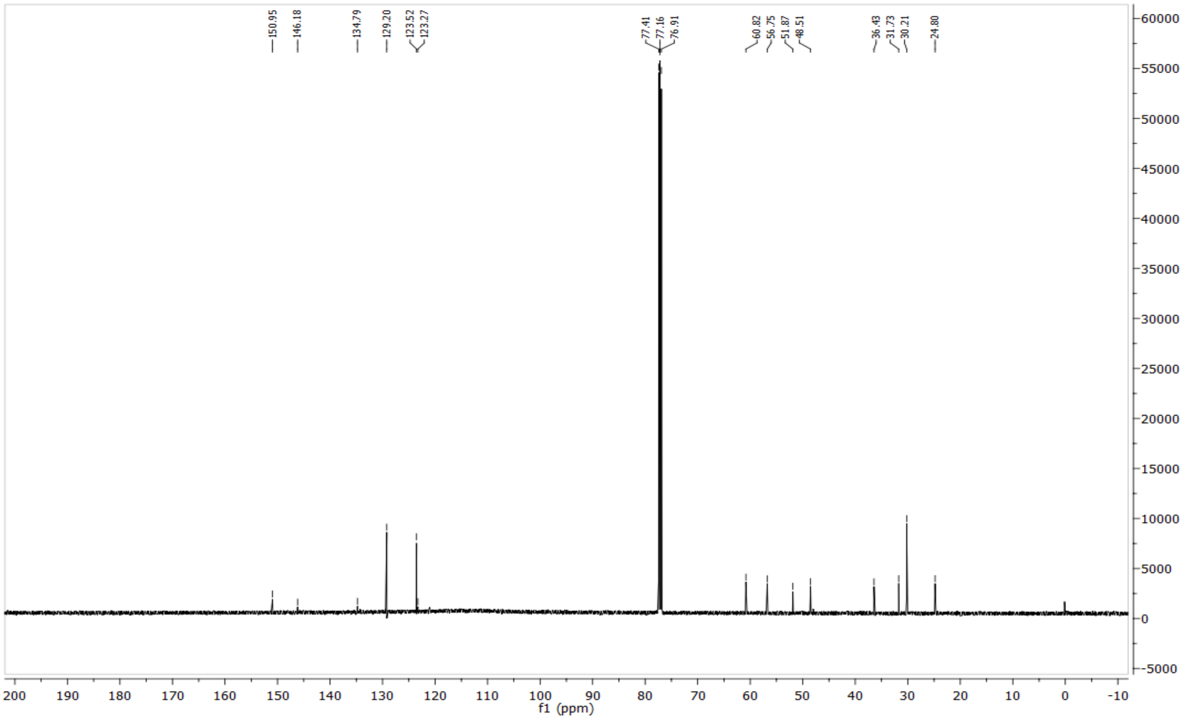

Figure S105: S1 ^{13}C NMR, 126 MHz, $CDCl_3$

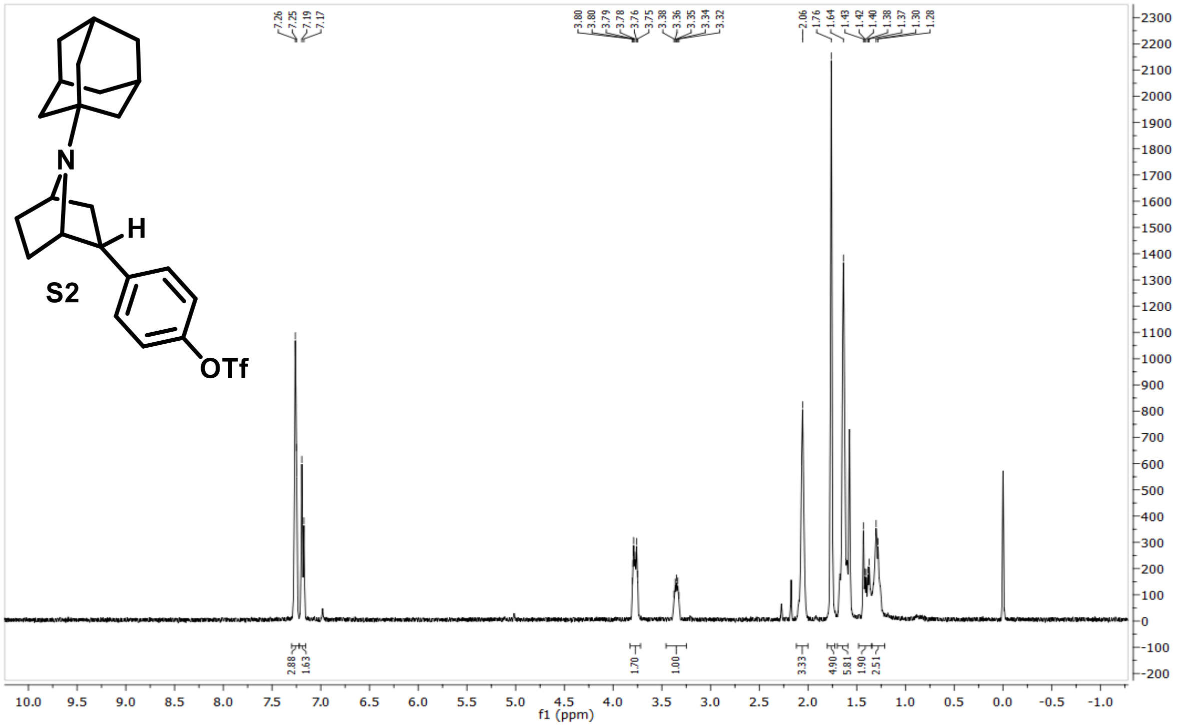

Figure S106: S2 ^{1}H NMR, 400 MHz, $CDCl_3$

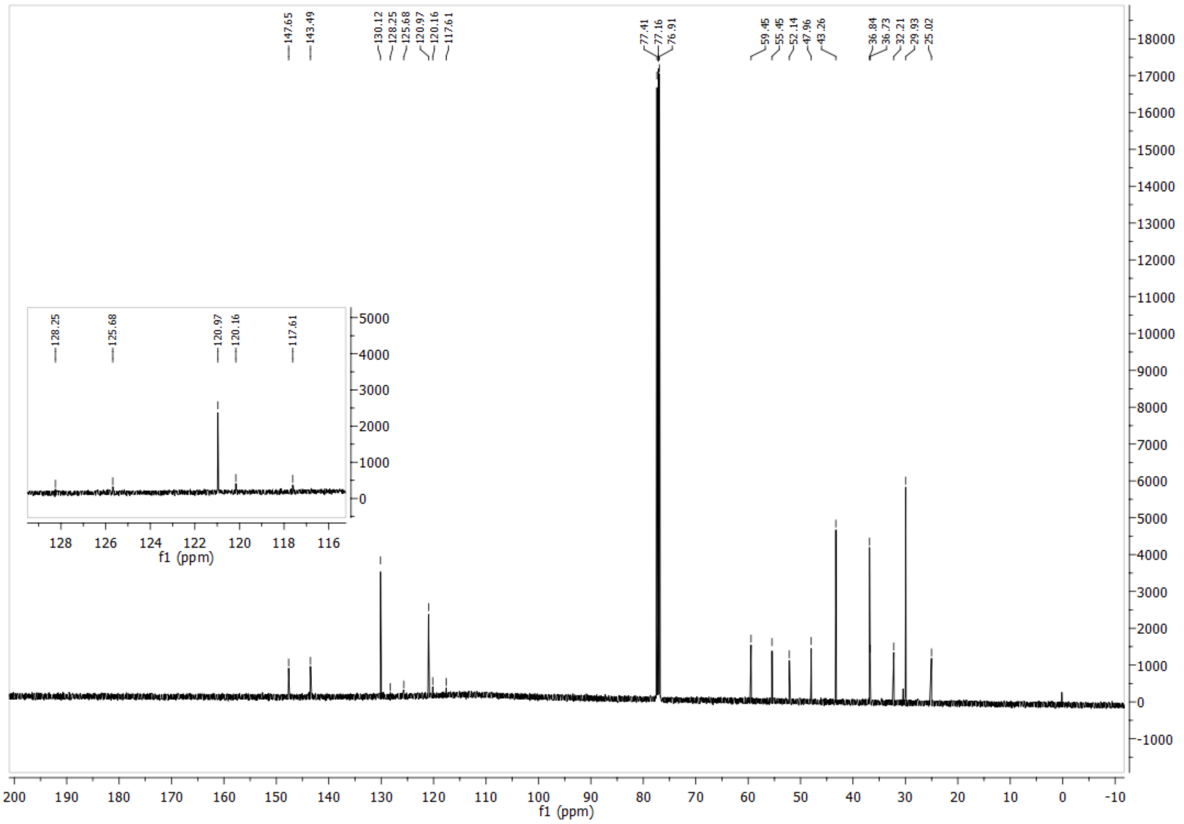

Figure S107: S2 ^{13}C NMR, 126 MHz, $CDCl_3$

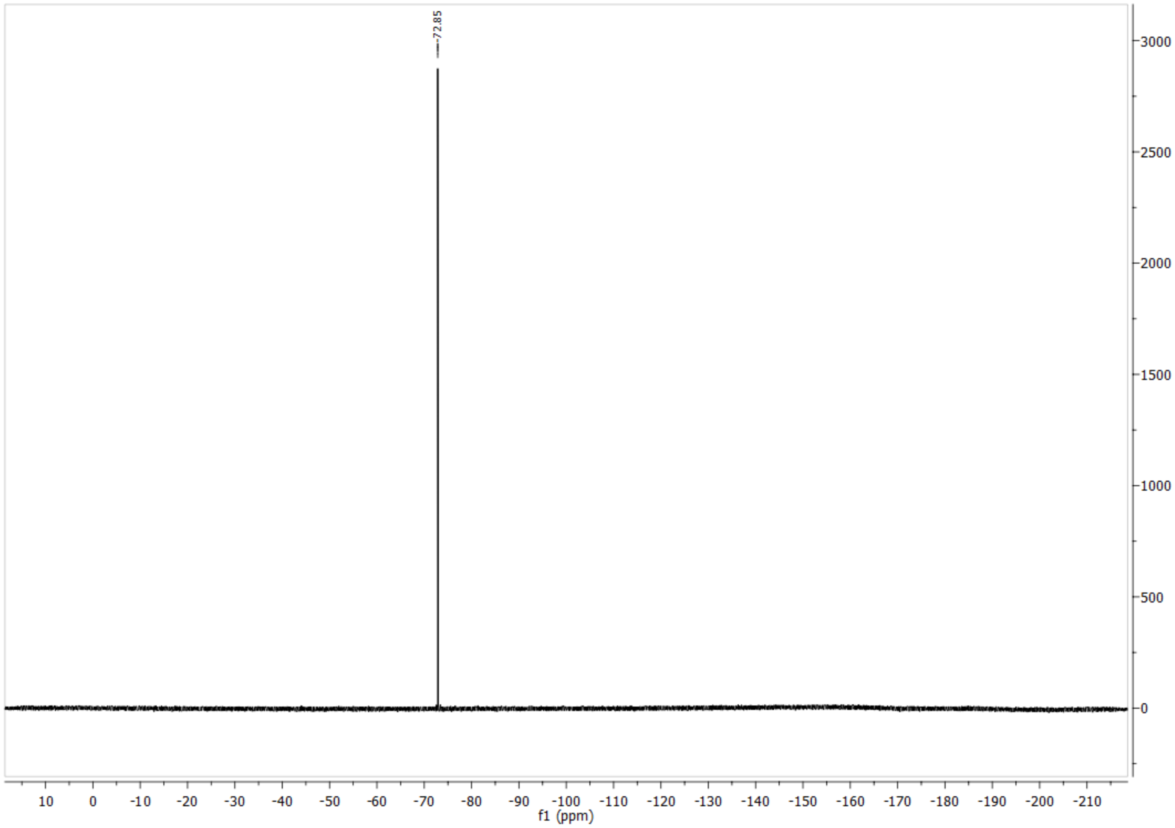

Figure S108: S2 ^{19}F NMR, 376 MHz, $CDCl_3$

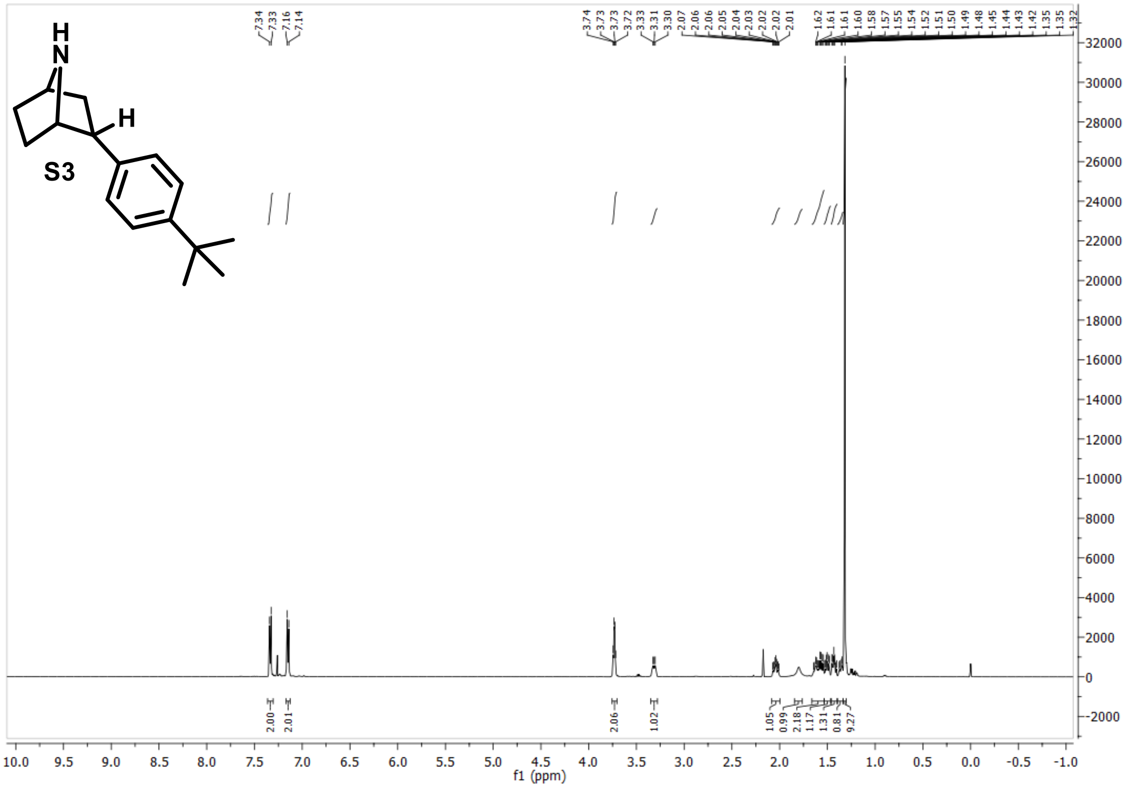

Figure S109: S3 ^{1}H NMR, 400 MHz, $CDCl_3$

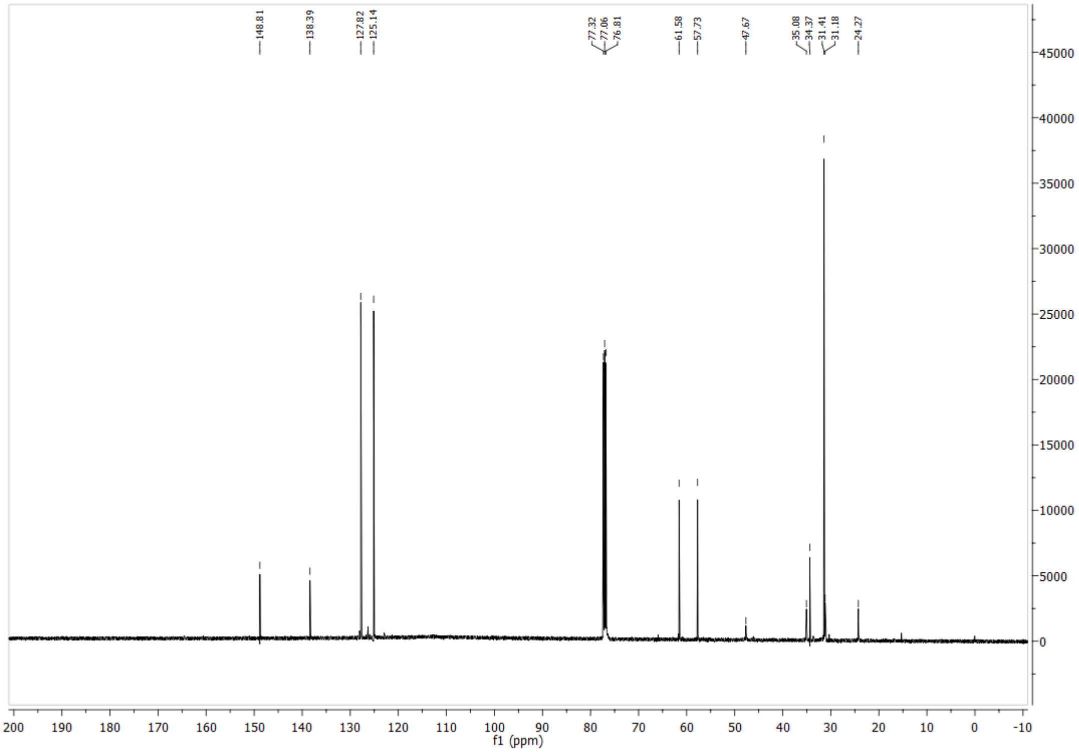

Figure S110: S3 ^{13}C NMR, 125 MHz, $CDCl_3$

2.2 Single Crystal X-Ray crystallography

Crystals of suitable quality for single crystal X-ray diffraction analysis of the tetrafluoroborate salt, **[10mb•H]BF_4** were prepared as follows. Compound **10mb** (50 mg, 0.12 mmol) was dissolved in 4 mL of DCM, and 50 μL (0.40 mmol) of 48% w/w tetrafluoroboric acid was added. After stirring for 30 minutes, 0.50 g $MgSO_4$ was added to the solution, and the mixture was filtered using a 0.20 μm PTFE syringe filter. The solution was layered with 5 mL of Et_2O and stored at -35 °C for 5 days, after which crystals of suitable quality for analysis had grown.

Single crystal XRD measurements were collected from a Bruker Smart Apex II CCD diffractometer using graphite-monochromated Mo-K (λ_α = 0.71073 Å) radiation. Refinement was performed using SHELXL, and molecular structure images were processed through Mercury.

Empirical formula	$C_{28}H_{33}BF_4N_2$	
Formula weight	484.390	
Temperature	170(2) K	
Wavelength	0.71073 Å	
Crystal system	Monoclinic	
Space group	P 2_1 (4)	
Unit cell dimensions	a = 13.2313(6) Å	α = 90°.
	b = 13.2633(6) Å	β = 90.053(3)°.
	c = 14.0926(6) Å	γ = 90°.
Volume	2473.12(19) Å^3	
Z	4	
F(000)	1024.0	
Crystal size	0.059 x 0.090 x 0.240 mm^3	
Theta range for data collection	1.445 to 27.710°.	
Index ranges	-17<=h<=17, --17<=k<=17, -18<=l<=18	
Reflections collected	11593	
Independent reflections	4933	
Completeness to theta = 25.242°	99.9 %	
Data / restraints / parameters	11593 / 1 / 652	
Goodness-of-fit on F^2	0.948	
Final R indices [I>2sigma(I)]	R1 = 0.0586, wR2 = 0.0727	
R indices (all data)	R1 = 0.1104, wR2 = 0.1744	
Extinction coefficient	0.0058(9)	
Largest diff. peak and hole	0.192 and -0.191 e.Å^{-3}	

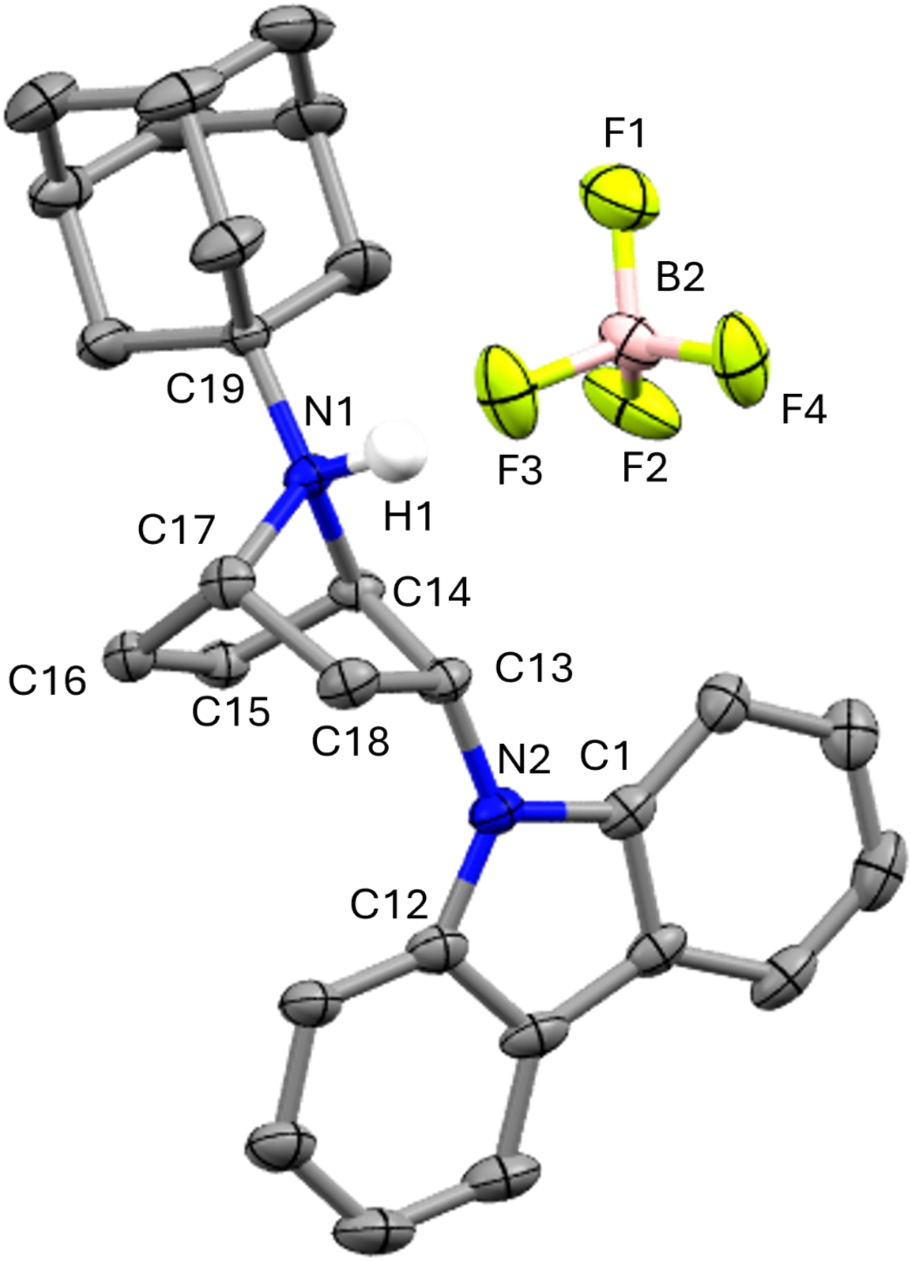

Figure S111. Molecular structure of (r)-[10mb•H]BF_4 with thermal ellipsoids shown at the 30% probability level. All carbon-bound hydrogen atoms are omitted for clarity.

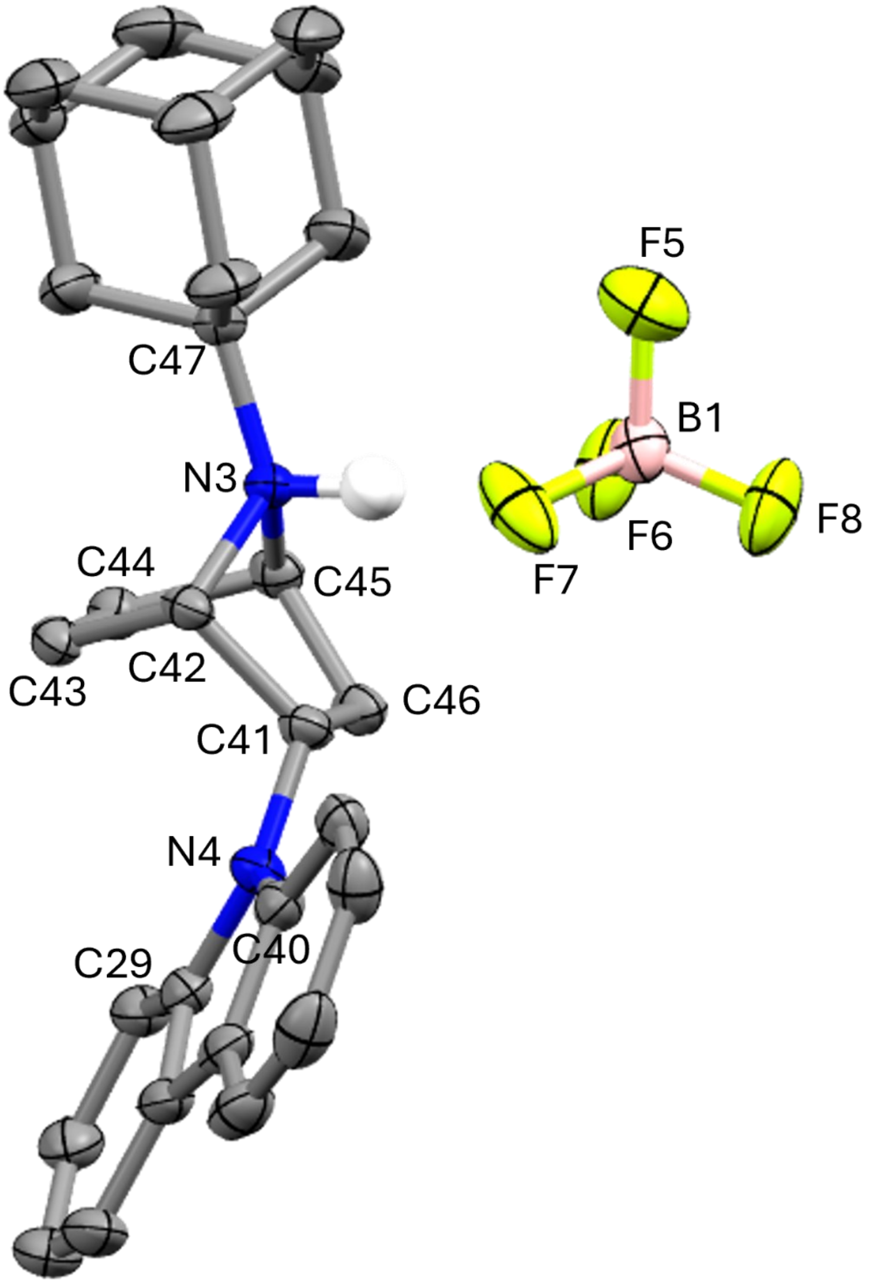

Figure S112. Molecular structure of (s)-[10mb•H]BF4 with thermal ellipsoids shown at the 30% probability level. All carbon-bound hydrogen atoms are omitted for clarity.

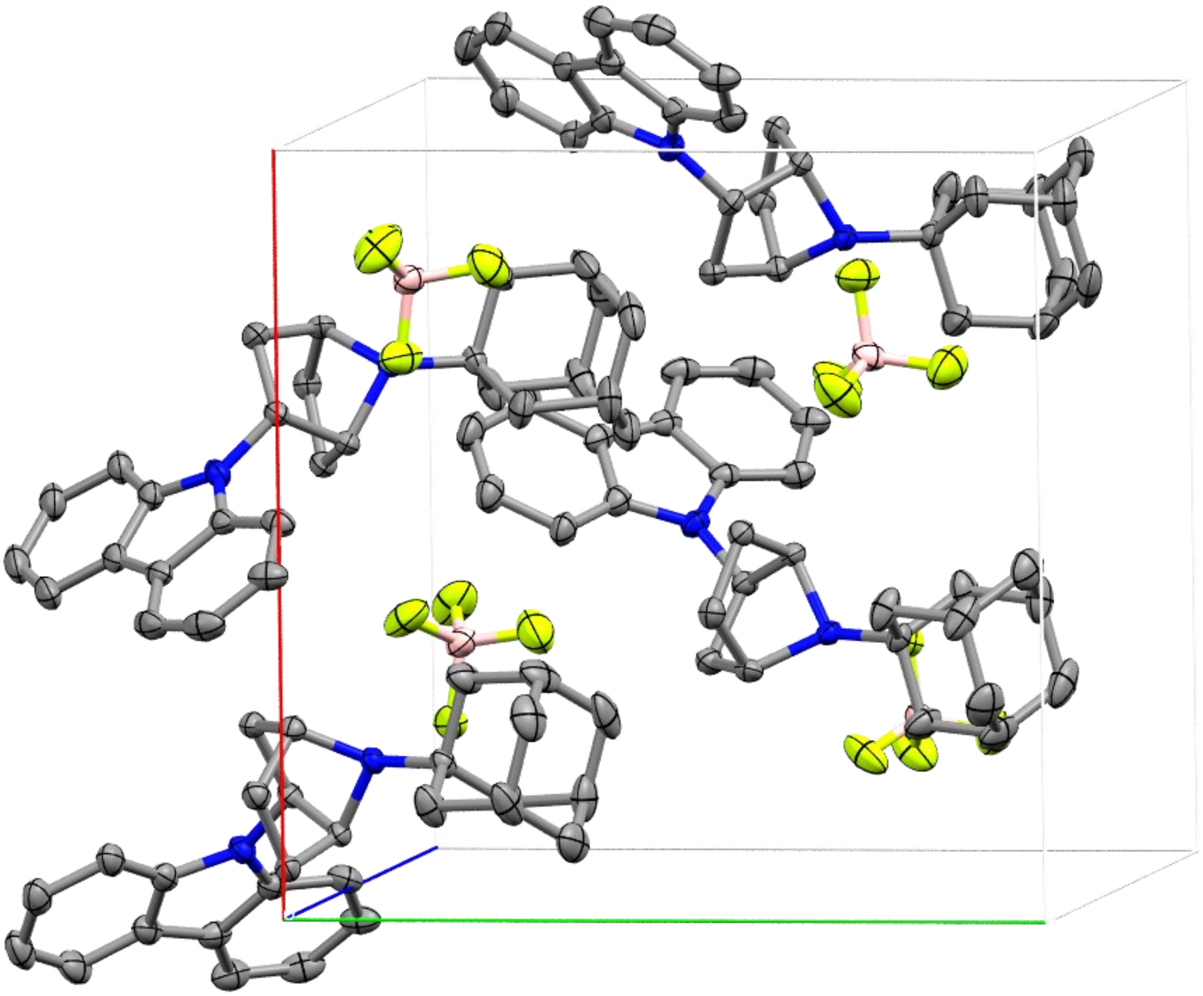

Figure S113. Packing diagram of (rac)-[10mb•H]BF4 with thermal ellipsoids shown at the 30% probability level. All carbon-bound hydrogen atoms are omitted for clarity. Each unit cell features four molecules of **[10mb•H]BF_4,** with two r-enantiomer and two s-enantiomer.

Table S2. Bond lengths for (rac)-[10mb•H]BF_4

Atom1	Atom2	Cyclicity	Length
B2	F3	acyclic	1.383(9)
B2	F4	cyclic	1.34(1)
B2	F1	acyclic	1.357(9)
B2	F2	acyclic	1.383(9)
N3	C42	cyclic	1.519(7)
N3	C45	cyclic	1.535(7)
N3	C47	acyclic	1.541(6)
N4	C29	cyclic	1.428(7)
N4	C40	cyclic	1.391(7)
N4	C41	acyclic	1.451(7)
C29	C30	cyclic	1.366(8)
C29	C34	cyclic	1.396(8)
C30	C31	cyclic	1.405(9)
C31	C32	cyclic	1.38(1)
C32	C33	cyclic	1.38(1)
C33	C34	cyclic	1.396(9)
C34	C35	cyclic	1.447(8)
C35	C36	cyclic	1.381(9)
C35	C40	cyclic	1.404(8)
C36	C37	cyclic	1.36(1)
C37	C38	cyclic	1.40(1)
C38	C39	cyclic	1.382(8)
C39	C40	cyclic	1.384(8)
C41	C42	cyclic	1.533(8)
C41	C46	cyclic	1.549(8)
C42	C43	cyclic	1.517(8)
C43	C44	cyclic	1.546(8)
C44	C45	cyclic	1.523(8)
C45	C46	cyclic	1.529(8)
C47	C48	cyclic	1.525(8)
C47	C52	cyclic	1.525(8)
C47	C53	cyclic	1.511(8)
C48	C49	cyclic	1.53(1)
C49	C50	cyclic	1.51(1)
C49	C56	cyclic	1.50(1)
C50	C51	cyclic	1.49(1)
C51	C52	cyclic	1.537(9)
C51	C55	cyclic	1.50(1)
C53	C54	cyclic	1.553(9)
C54	C55	cyclic	1.53(1)
C54	C56	cyclic	1.54(1)
C3	C4	cyclic	1.39(1)
C3	C2	cyclic	1.382(8)
C4	C5	cyclic	1.35(1)
C5	C6	cyclic	1.380(9)
C6	C7	cyclic	1.439(8)
C6	C1	cyclic	1.411(8)
C7	C8	cyclic	1.398(9)
C7	C12	cyclic	1.410(8)
C8	C9	cyclic	1.38(1)
C9	C10	cyclic	1.38(1)
C10	C11	cyclic	1.393(9)
N1	C14	cyclic	1.525(7)
N1	C17	cyclic	1.543(7)
N1	C19	acyclic	1.531(6)
C1	N2	cyclic	1.392(7)
C1	C2	cyclic	1.383(8)
C11	C12	cyclic	1.374(8)
C12	N2	cyclic	1.420(7)
C13	C14	cyclic	1.540(8)
C13	C18	cyclic	1.556(8)
C13	N2	acyclic	1.450(7)
C14	C15	cyclic	1.508(7)
C15	C16	cyclic	1.542(7)
C16	C17	cyclic	1.524(8)
C17	C18	cyclic	1.529(8)
C19	C20	cyclic	1.521(8)
C19	C24	cyclic	1.528(8)
C19	C25	cyclic	1.517(8)
C20	C21	cyclic	1.536(9)
C21	C22	cyclic	1.49(1)
C21	C28	cyclic	1.48(1)
C22	C23	cyclic	1.52(1)
C23	C24	cyclic	1.53(1)
C23	C27	cyclic	1.51(1)
C25	C26	cyclic	1.549(9)
C26	C27	cyclic	1.53(1)
C26	C28	cyclic	1.55(1)
F5	B1	acyclic	1.379(9)
F6	B1	acyclic	1.350(9)
F7	B1	cyclic	1.32(2)
F8	B1	acyclic	1.395(9)

3.1 Computational Calculations

The quantum chemistry method of meta-hybrid density functional theory (DFT)[9] was carried out at the Center for Computational Sciences (CCS) at Duquesne University using Gaussian 16. The M06-2X functional[10] with Dunning's jul-cc-pv[D,T,Q]z basis sets[11] were used to calculate electronic, enthalpic and free energies for both ground and transition structures. These energies can then be extrapolated to the complete basis set (CBS) limit (Eq S1). The CBS limit is not a basis set, rather it is an extrapolated estimate of a result using an infinitely large basis set.[12] The procedure removes any error from the linear combination of atomic orbitals approximation. The use M06-2X, developed by Truhlar and co-workers, has been reported to be accurate to within 1.2 kcal/mol for reaction barriers and within 0.37 kcal/mol of non-covalent interaction energies.[13] Our group's previously reported paper verified our theory level, while also using jul-cc-pVDZ, with second-order Møller-Plesset calculations on the first deprotonation transition structure.[14] Vibrational frequency calculations were used to confirm all stationary points as either minima or transition structures and to provide thermodynamic corrections for enthalpies and free energies.

$$Complete\ Basis\ Set\ (CBS) = E_\infty = \frac{-E_{TZ}^2 + E_{DZ}E_{QZ}}{E_{DZ} - 2E_{TZ} + E_{QZ}} \quad \textbf{(Eq. S1)}$$

Table S3: Calculated energies for the [3+2] transition state between styrene and tert-butyl pyrrolidine *N*-oxide. All calculations were completed in M062x-jul-cc-pv_z, [pcm = THF]. CBS = complete basis set.

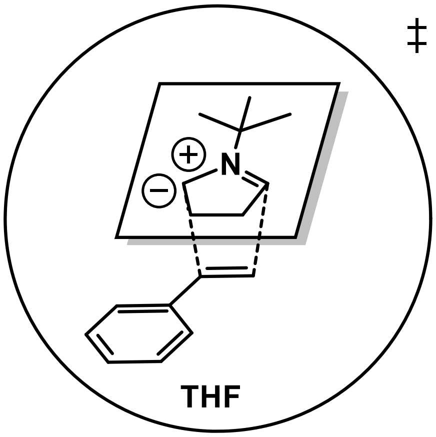

	G298 (kcal/mol)			
	pv**D**z	pv**T**z	pv**Q**z	**CBS**
**endo TS**	5.71	5.77	6.72	7.53
**exo TS**	6.84	6.96	7.92	8.73
**ΔΔG‡**	1.13	1.19	1.20	1.20
**% endo**	87	88	88	88

Note: The % endo was calculated based off the applied Curtin-Hammet principle from free energy to % diastereomer (Eq. S2 & Eq. S3)

$$\%\,exo = 100\%\ x\ \frac{[exo]}{[exo]+[endo]} = 100\%\ x\ \frac{\frac{[exo]}{[endo]}}{1+\frac{[exo]}{[endo]}} = 100\%\ x\ \frac{e^{-\frac{\Delta\Delta G^\ddagger}{RT}}}{1+e^{-\frac{\Delta\Delta G^\ddagger}{RT}}} \quad \textbf{(Eq. S2)}$$

$$\%\,endo = 100\% - \%\,exo \quad \textbf{(Eq. S3)}$$

3.1.1 Computational Outputs

Computational Outputs

Transition State Endo Double Zeta – [Endo TS jul-cc-pvDz]

Input:

```
--------------------------------------------------------------------

 # opt=(calcfc,noeigen,ts) freq scrf=(solvent=thf,pcm) nosymm jul-cc-pvdz m062x optcyc=180

 --------------------------------------------------------------------
```

Optimized Structure:

```
0 1
N 0.94594100 -0.30433500 0.44845500
C 0.85995500 -0.36104600 1.77459600
C 1.94042600 0.48744500 2.38764400
C 2.67272700 1.06847900 1.14386000
C 1.74982200 0.69359400 0.00465600
C 0.04981500 2.41654800 0.36015100
C -0.77178500 1.93899300 1.34167400
H 0.22191900 -1.06912800 2.28214200
H 1.52432700 1.27530000 3.02990000
H 2.61053400 -0.11128100 3.01880300
H 3.66102000 0.59701900 1.01763200
H 2.83928200 2.14908300 1.22431200
H 2.04063400 0.70097700 -1.04074700
H -0.20642300 2.28672200 -0.68699200
H 0.80582300 3.17344600 0.56924100
H -1.59909200 1.28254200 1.06069700
C -0.75007100 2.31857600 2.75537300
C -1.66652400 1.73159000 3.64950000
C -1.68853500 2.06879800 4.99913800
C -0.78911800 3.01234200 5.50590800
C 0.12591600 3.60741300 4.63582400
C 0.14630800 3.26745200 3.28356800
H -2.37040100 0.98934600 3.26649900
H -2.41196600 1.59316100 5.66221700
H -0.80329900 3.27965600 6.56207900
H 0.83298300 4.34723600 5.01278100
H 0.86749200 3.75529400 2.62659900
C 0.15577800 -1.16748100 -0.48308800
C -0.85480700 -2.01039500 0.29137900
C -0.58913300 -0.29837800 -1.49743400
C 1.15337600 -2.08641000 -1.19426400
H -1.54561500 -1.38019100 0.86435900
H -1.43829500 -2.59051700 -0.43286700
H -0.36032000 -2.71588100 0.96930800
H 0.09405000 0.34216400 -2.06515400
H -1.10408800 -0.96088800 -2.20412700
```

H -1.33929600 0.32883700 -1.00227600
H 1.73069300 -2.66150700 -0.45935500
H 0.60576000 -2.78675400 -1.83730300
H 1.84695500 -1.51089600 -1.81831300

Thermochemistry:

Zero-point correction= 0.352606 (Hartree/Particle)
Thermal correction to Energy= 0.369488
Thermal correction to Enthalpy= 0.370432
Thermal correction to Gibbs Free Energy= 0.308212
Sum of electronic and zero-point Energies= -677.627746
Sum of electronic and thermal Energies= -677.610864
Sum of electronic and thermal Enthalpies= -677.609920
Sum of electronic and thermal Free Energies= -677.672140

Transition State Endo Triple Zeta – [Endo TS jul-cc-pvTz]

Input:

----

opt=(calcfc,ts,noeigen,recalcfc=50) freq scrf=(solvent=thf,pcm) nosymm jul-cc-pvtz m062x optcyc=100
----

0 1
N -0.09623200 -0.29858800 -0.20156000
C -0.10203600 -0.44078400 1.11706600
C 1.27830100 -0.78219300 1.59493500
C 2.11305000 -0.76000000 0.28431400
C 1.14905600 -0.16972800 -0.71689300
C 1.35666900 2.13821300 -0.07049400
C 0.55864400 2.30411100 1.01980900
H -1.01388000 -0.50806900 1.67865500
H 1.64429200 -0.06293900 2.32874000
H 1.30187200 -1.76010600 2.07806300
H 2.40318300 -1.77462000 -0.00570600
H 3.03024800 -0.18182300 0.38609300
H 1.27565500 -0.23467700 -1.78538700
H 0.99929100 2.39691600 -1.05435700
H 2.43184300 2.05795300 0.02472600
H -0.49012800 2.53599300 0.86491200
C 0.99209900 2.36256300 2.41150800
C 0.04864800 2.59670000 3.42289700
C 0.41588000 2.67705900 4.75530600
C 1.74805100 2.52543400 5.12839000
C 2.69808400 2.29120200 4.14289800
C 2.32835900 2.20963800 2.80799400
H -0.99257700 2.71360100 3.14486000

H -0.33960500 2.85971900 5.50857800
H 2.03844400 2.58857500 6.16803100
H 3.73891600 2.17029700 4.41455100
H 3.09157500 2.02790600 2.06167400
C -1.32616100 -0.15595200 -1.03778800
C -2.56914600 -0.07384900 -0.15997100
C -1.23601600 1.10594200 -1.89211800
C -1.41079800 -1.39635500 -1.92736600
H -2.51505000 0.77019600 0.52726600
H -3.43228600 0.06827100 -0.80837400
H -2.72382700 -0.99021600 0.40806900
H -0.35771000 1.09893700 -2.53444500
H -2.12094000 1.15583300 -2.52586800
H -1.21012200 1.99800900 -1.26820300
H -1.42891300 -2.29913200 -1.31676500
H -2.32622000 -1.35596500 -2.51729300
H -0.56354800 -1.45065900 -2.60918000

Thermochemistry:

Zero-point correction= 0.354044 (Hartree/Particle)
Thermal correction to Energy= 0.370657
Thermal correction to Enthalpy= 0.371602
Thermal correction to Gibbs Free Energy= 0.310190
Sum of electronic and zero-point Energies= -677.789067
Sum of electronic and thermal Energies= -677.772453
Sum of electronic and thermal Enthalpies= -677.771509
Sum of electronic and thermal Free Energies= -677.832921

Transition State Endo Quadruple Zeta – [Endo TS jul-cc-pvQz]

Input:

----

opt=(calcfc,ts,noeigen,recalcfc=15) freq scrf=(solvent=thf,pcm) nosymm jul-cc-pvqz m062x optcyc=30
----

0 1
N -0.09739900 -0.29732400 -0.20041500
C -0.10513400 -0.43768500 1.11743600
C 1.27362900 -0.78081700 1.59790900
C 2.11095000 -0.75672800 0.28913500
C 1.14795200 -0.16769600 -0.71373300
C 1.35447300 2.13722800 -0.07107300
C 0.55734900 2.30365400 1.01959700
H -1.01689100 -0.50525600 1.67715500
H 1.63803200 -0.06501100 2.33450400
H 1.29498800 -1.75897100 2.07866600

H 2.40345600 -1.76975600 -0.00046600
H 3.02670400 -0.17822800 0.39234700
H 1.27628700 -0.23464400 -1.78094400
H 0.99803600 2.39771600 -1.05378800
H 2.42881700 2.05774900 0.02387700
H -0.49041200 2.53688100 0.86568500
C 0.99240200 2.36211300 2.41068700
C 0.05017100 2.59367800 3.42333700
C 0.41913900 2.67316500 4.75483700
C 1.75168400 2.52304300 5.12564100
C 2.70042800 2.29140000 4.13899000
C 2.32911900 2.21094200 2.80481500
H -0.99082700 2.70896500 3.14717800
H -0.33476200 2.85375600 5.50891400
H 2.04320000 2.58532800 6.16410000
H 3.74091500 2.17188300 4.40900000
H 3.09090500 2.03151400 2.05773400
C -1.32551300 -0.15577400 -1.03793500
C -2.57005300 -0.07433000 -0.16198000
C -1.23488600 1.10590900 -1.89275500
C -1.40804700 -1.39666200 -1.92727700
H -2.51879800 0.76991700 0.52366700
H -3.43225200 0.06509700 -0.81048900
H -2.72403500 -0.98878500 0.40738600
H -0.35692200 1.09879900 -2.53389300
H -2.11830000 1.15639700 -2.52679800
H -1.20894700 1.99775500 -1.27022600
H -1.42757200 -2.29872900 -1.31747200
H -2.32092600 -1.35739700 -2.51934000
H -0.55999500 -1.45168400 -2.60644400

Thermochemistry:

Zero-point correction= 0.354140 (Hartree/Particle)
Thermal correction to Energy= 0.370747
Thermal correction to Enthalpy= 0.371692
Thermal correction to Gibbs Free Energy= 0.310275
Sum of electronic and zero-point Energies= -677.831128
Sum of electronic and thermal Energies= -677.814521
Sum of electronic and thermal Enthalpies= -677.813577
Sum of electronic and thermal Free Energies= -677.874993

Transition State Exo Double Zeta– [Exo TS jul-cc-pvDz]

Input:

```
----------------------------------------------------------------------
 # opt=(calcfc,ts,noeigen,recalcfc=50,modredundant) freq scrf=(solvent=thf,pcm) nosymm jul-cc-pvdz
m062x optcyc=100
 ----------------------------------------------------------------------
0 1
N -0.24757900 0.31272900 0.01790400
C -0.19703400 -0.19355200 1.24984300
C 1.20564100 -0.04545000 1.79125200
C 1.91198200 0.80558300 0.69394200
C 0.77996800 1.15364800 -0.25005400
C -0.13483700 2.92008800 1.17227000
C -0.81835200 2.23490900 2.13943200
H -0.96466900 -0.85620600 1.62415300
H 1.22807300 0.43845700 2.77433800
H 1.68691900 -1.02632200 1.91262700
H 2.68271800 0.21352900 0.17703900
H 2.41295600 1.68864100 1.10720900
H 0.93553600 1.47980600 -1.27273900
H -0.65296600 3.42424400 0.35701600
H 0.89658200 3.22371800 1.33445300
H -0.28403700 1.89002500 3.02428400
C -2.27751500 2.06101800 2.19250600
C -2.84060000 1.14249900 3.10007500
C -4.21283000 0.92539700 3.16191600
C -5.07729900 1.62823300 2.31556200
C -4.54187600 2.56606000 1.43205600
C -3.16546800 2.78891600 1.38140500
H -2.17249300 0.57163200 3.74842800
H -4.61325000 0.19855400 3.86928800
H -6.15234300 1.45546900 2.35506900
H -5.20263400 3.14074100 0.78205000
H -2.77761700 3.55207400 0.70666200
C -1.35505600 0.06436100 -0.95613900
C -1.77658800 1.37455300 -1.62076500
C -0.80353800 -0.91005600 -2.00206900
C -2.56300300 -0.55796000 -0.25746000
H -0.94966500 1.85046600 -2.15778900
H -2.56792400 1.15008500 -2.34676500
H -2.17413500 2.07334400 -0.87919600
H -0.45998500 -1.83332700 -1.51838800
H -1.59610600 -1.16231100 -2.71782000
H 0.03445200 -0.46327700 -2.55068100
H -2.93058800 0.07876300 0.55623300
H -3.36099800 -0.66862100 -1.00124700
H -2.33336900 -1.55587400 0.13421000
```

Thermochemistry:

Zero-point correction= 0.353088 (Hartree/Particle)
Thermal correction to Energy= 0.369782
Thermal correction to Enthalpy= 0.370726
Thermal correction to Gibbs Free Energy= 0.310118
Sum of electronic and zero-point Energies= -677.627259
Sum of electronic and thermal Energies= -677.610565
Sum of electronic and thermal Enthalpies= -677.609621
Sum of electronic and thermal Free Energies= -677.670229

Transition State Exo Triple Zeta – [Exo TS jul-cc-pvTz]

Input:

----

opt=(calcfc,ts,noeigen,recalcfc=50,modredundant) freq scrf=(solvent=thf,pcm) nosymm jul-cc-pvtz m062x optcyc=100

----

0 1
N 0.63734400 -0.07217000 0.40819000
C 0.57060600 -0.01023500 1.73528600
C 1.79949800 0.68681700 2.26016000
C 2.48302500 1.20350400 0.96148800
C 1.48378800 0.84375900 -0.11309200
C -0.00680900 2.68127600 0.36057800
C -0.67282900 2.27542800 1.47914300
H -0.08447300 -0.65215300 2.29435600
H 1.56237100 1.49071400 2.95453800
H 2.44459300 -0.01100300 2.79812400
H 3.43315500 0.68895700 0.79573300
H 2.70274100 2.26891400 1.00158000
H 1.73566400 0.78405400 -1.15905000
H -0.47982800 2.67339900 -0.61240900
H 0.87746600 3.29573900 0.44061400
H -0.23821400 2.47185700 2.45053700
C -2.04976600 1.77179100 1.51751500
C -2.54152300 1.17990000 2.69047000
C -3.82551900 0.66965600 2.76402800
C -4.67252400 0.73573800 1.66077800
C -4.21541600 1.34390000 0.49983800
C -2.92992800 1.86506200 0.43349400
H -1.88279200 1.10452600 3.54815800
H -4.16866700 0.21203400 3.68290400
H -5.67422700 0.33177500 1.71200800
H -4.86785900 1.42545400 -0.36005000
H -2.61225600 2.36548200 -0.47181800
C -0.20560600 -0.96282200 -0.44453300

C -1.34224600 -1.57538400 0.36641000
C -0.79057600 -0.17690600 -1.61434600
C 0.71253300 -2.07188000 -0.96127600
H -1.97497300 -0.80724500 0.81153900
H -1.95106200 -2.17582100 -0.30834400
H -0.96884600 -2.23423100 1.14953400
H -0.01779000 0.26589900 -2.23899300
H -1.37164200 -0.86221100 -2.23116700
H -1.45105600 0.60881100 -1.25677800
H 1.15786300 -2.61461100 -0.12732100
H 0.13289800 -2.77205000 -1.56258900
H 1.50996800 -1.66139900 -1.57970800
Thermochemistry:

Zero-point correction= 0.354298 (Hartree/Particle)
Thermal correction to Energy= 0.370792
Thermal correction to Enthalpy= 0.371736
Thermal correction to Gibbs Free Energy= 0.311463
Sum of electronic and zero-point Energies= -677.788192
Sum of electronic and thermal Energies= -677.771698
Sum of electronic and thermal Enthalpies= -677.770754
Sum of electronic and thermal Free Energies= -677.831027

Transition State Exo Quadruple Zeta – [Exo TS jul-cc-pvQz]

Input:

 # opt=(calcfc,ts,noeigen,recalcfc=15) freq scrf=(solvent=thf,pcm) nosymm jul-cc-pvqz m062x optcyc=30
----
0 1
N 0.63703000 -0.07205600 0.40781200
C 0.57136800 -0.01133500 1.73414800
C 1.79991500 0.68602600 2.25887000
C 2.48133800 1.20489100 0.96026800
C 1.48098200 0.84532500 -0.11313800
C -0.00686900 2.67879000 0.36128300
C -0.67237900 2.27370500 1.48036200
H -0.08056700 -0.65505000 2.29290800
H 1.56283200 1.48764000 2.95446300
H 2.44545500 -0.01117600 2.79519000
H 3.43115500 0.69237400 0.79270300
H 2.70032000 2.26950200 1.00083600
H 1.73172500 0.78726700 -1.15847200
H -0.48098700 2.67210500 -0.61018400
H 0.87567600 3.29425800 0.44021800
H -0.23778000 2.47108100 2.45066100

C -2.04969900 1.77141700 1.51893800
C -2.54215500 1.17973100 2.69133000
C -3.82596200 0.67004300 2.76384900
C -4.67190900 0.73653300 1.66033800
C -4.21420400 1.34455200 0.50015100
C -2.92895000 1.86543200 0.43478300
H -1.88472500 1.10399900 3.54883800
H -4.16978200 0.21288000 3.68164500
H -5.67292500 0.33316300 1.71086600
H -4.86568900 1.42635300 -0.35930300
H -2.61065200 2.36532800 -0.46953900
C -0.20531200 -0.96245300 -0.44418500
C -1.34297300 -1.57410600 0.36629600
C -0.78918500 -0.17732200 -1.61522800
C 0.71286600 -2.07235400 -0.95949000
H -1.97717400 -0.80674500 0.80828500
H -1.94978500 -2.17655100 -0.30682600
H -0.97131600 -2.22989600 1.15138900
H -0.01657600 0.26363100 -2.23970200
H -1.37031300 -0.86154500 -2.23136700
H -1.44842600 0.60923900 -1.26012700
H 1.15641100 -2.61521900 -0.12598500
H 0.13513100 -2.77197200 -1.56143700
H 1.51117300 -1.66315900 -1.57592300

Thermochemistry:

Zero-point correction= 0.354404 (Hartree/Particle)
Thermal correction to Energy= 0.370909
Thermal correction to Enthalpy= 0.371854
Thermal correction to Gibbs Free Energy= 0.311519
Sum of electronic and zero-point Energies= -677.830194
Sum of electronic and thermal Energies= -677.813689
Sum of electronic and thermal Enthalpies= -677.812745
Sum of electronic and thermal Free Energies= -677.873080

Ground State Endo Triple Zeta – [Endo GS jul-cc-pvTz]

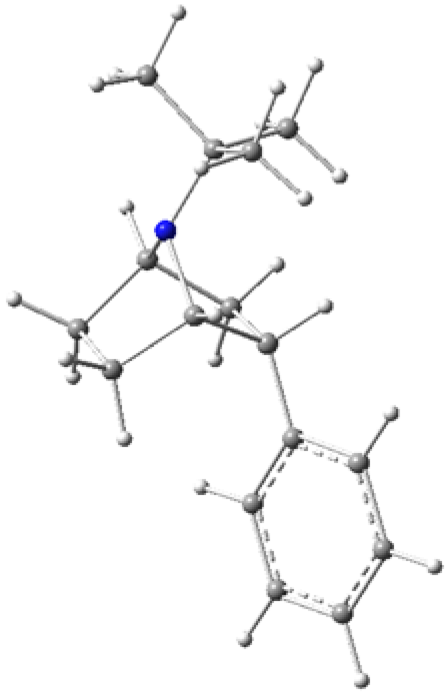

Figure S114: Ground state of 10aa M062x-cc-pvTz (pcm = THF)

Input:

 # opt=(calcfc,noeigen,recalcfc=60,modredundant) freq scrf=(solvent=thf,pcm) nosymm jul-cc-pvtz m062x optcyc=180

 ----

0 1
N 0.02113600 0.10013200 -0.01840200
C 0.13033400 0.05183500 1.44337600
C 1.65315200 0.17501600 1.59694700
C 2.01844700 1.15084300 0.44440100
C 0.63783700 1.42745600 -0.16951000
C -0.12336900 2.35306900 0.81692700
C -0.56139300 1.36545700 1.92714100
H -0.28753500 -0.84556600 1.89006300
H 1.94912400 0.53793800 2.57820700
H 2.11386900 -0.79850900 1.44380800
H 2.67431000 0.67394200 -0.28114300
H 2.50017700 2.06308100 0.79565700
H 0.65862800 1.77809700 -1.19866900
H -0.98542500 2.82841000 0.35669300
H 0.52311100 3.14709400 1.18501800
H -1.63532900 1.19347800 1.84885700
C -0.29128000 1.73508200 3.36327100

C -1.13845800 1.23687100 4.35377800
C -0.91741800 1.50465500 5.69736600
C 0.16385000 2.28610300 6.08139500
C 1.01689900 2.79023700 5.10891900
C 0.79087900 2.51671000 3.76652200
H -1.98886500 0.63157700 4.06164800
H -1.59280200 1.10716200 6.44328600
H 0.33850000 2.50259300 7.12648100
H 1.86326800 3.40060000 5.39483800
H 1.47466000 2.91715400 3.02891300
C -1.21599100 -0.23339600 -0.74949900
C -2.23646500 0.90446200 -0.91654100
C -0.77712400 -0.67044800 -2.15118000
C -1.89623100 -1.41092300 -0.05712100
H -1.79832100 1.75199300 -1.44444700
H -3.07352800 0.54140800 -1.51438600
H -2.63695400 1.25197300 0.03414200
H -0.12631000 -1.54159900 -2.08343600
H -1.64212300 -0.92052000 -2.76714100
H -0.22719600 0.13031200 -2.64790500
H -2.31923000 -1.11988800 0.90566300
H -2.70863900 -1.78193800 -0.68197500
H -1.18637300 -2.22312400 0.10442300

Thermochemistry:

Zero-point correction= 0.361221 (Hartree/Particle)
Thermal correction to Energy= 0.376181
Thermal correction to Enthalpy= 0.377125
Thermal correction to Gibbs Free Energy= 0.319804
Sum of electronic and zero-point Energies= -677.873578
Sum of electronic and thermal Energies= -677.858618
Sum of electronic and thermal Enthalpies= -677.857674
Sum of electronic and thermal Free Energies= -677.914995

Ground State Exo Triple Zeta – [Exo GS jul-cc-pvTz]

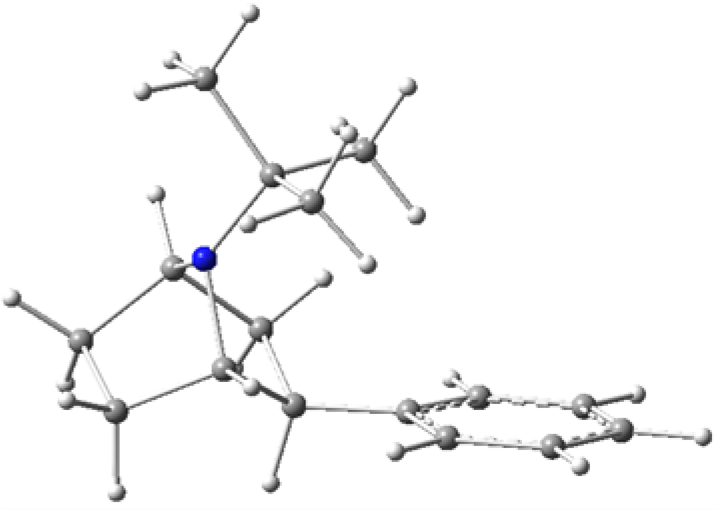

Figure S115: Ground state of 11aa M062x-cc-pvTz (pcm = THF)

Input:

 # opt=(calcfc,noeigen,recalcfc=60,modredundant) freq scrf=(solvent=thf,pcm) nosymm jul-cc-pvtz m062x optcyc=180
 ----

0 1
N 0.01271100 0.00826300 -0.09671800
C -0.15676000 0.04033600 1.35999900
C 1.32538300 0.06240300 1.77846600
C 1.95044100 0.97980000 0.69016800
C 0.72952600 1.28437900 -0.19422200
C -0.15742800 2.30123600 0.56113000
C -0.73893300 1.44289300 1.71807500
H -0.70537300 -0.80992500 1.74869300
H 1.46696000 0.44071500 2.78951700
H 1.73964900 -0.94208300 1.72484100
H 2.71479000 0.45230200 0.12328400
H 2.39166200 1.88635400 1.10225600
H 0.96796300 1.57203000 -1.21542400
H -0.94595400 2.67923300 -0.08383100
H 0.41771600 3.15399700 0.91962700
H -0.29241700 1.76094500 2.66317800
C -2.23778200 1.54275600 1.92080300
C -2.97540200 0.48850700 2.45803500
C -4.34204700 0.60371700 2.67933100
C -5.00286100 1.78653800 2.38086100
C -4.27830200 2.85548800 1.87085600
C -2.91434100 2.73201800 1.64671500
H -2.48490200 -0.44078700 2.71407800
H -4.88948800 -0.23527000 3.08813000

H -6.06719700 1.87694000 2.54971800
H -4.77561100 3.78943300 1.64475600
H -2.36854500 3.57834600 1.25042700
C -1.03892600 -0.36017700 -1.05735600
C -2.03514500 0.74626500 -1.43290100
C -0.31879900 -0.80324400 -2.33546700
C -1.81359200 -1.55027300 -0.49719200
H -1.52738200 1.59157900 -1.89954400
H -2.74994800 0.35113100 -2.15653400
H -2.59427100 1.10164200 -0.56872400
H 0.32839900 -1.65429500 -2.12460600
H -1.04059300 -1.08844900 -3.10206200
H 0.29536800 0.00462800 -2.73540200
H -2.44444000 -1.25457900 0.34175900
H -2.45898900 -1.96271200 -1.27287700
H -1.12789500 -2.33136900 -0.16574800

Thermochemistry:

Zero-point correction= 0.361066 (Hartree/Particle)
Thermal correction to Energy= 0.376042
Thermal correction to Enthalpy= 0.376986
Thermal correction to Gibbs Free Energy= 0.319950
Sum of electronic and zero-point Energies= -677.871566
Sum of electronic and thermal Energies= -677.856590
Sum of electronic and thermal Enthalpies= -677.855646
Sum of electronic and thermal Free Energies= -677.912682

Steric analysis was carried out using the NBO 7.0 program[15] to compare the 90% experimental *endo* selectivity of the [3+2] reaction of *tert*-butyl pyrrolidine ylide with styrene. The steric energy differences between the *endo* and *exo* transition structures were calculated with the M06-2X density functional[10] and the jul-cc-pvDz basis set[16] with implicit THF solvent using the polarizable continuum model (PCM).[17, 18]

NBO analysis showed that the *endo* transition structure had intramolecular steric components totaling 66.2 kcal/mol, while the *exo* transition structure had steric components of 76.6 kcal/mol (Figure S115). Thus, NBO analysis predicts that the *exo* transition structure has a steric exchange value 10.4 kcal/mol higher than the *endo* transition state. This data is in agreement with experiment which shows a strong preference for the *endo* structure.

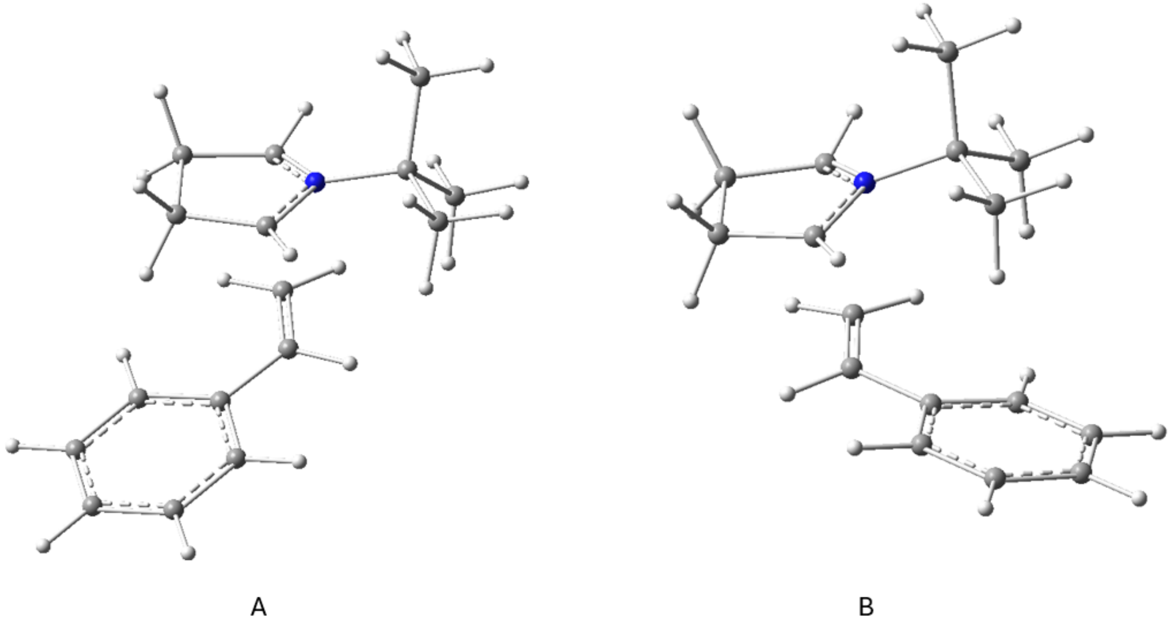

Figure S115: (A) Endo (10aa) transition structure. (B) Exo (11aa) transition structure. Color key: N (Blue), C (Grey), H (White).

The steric interactions of the *endo* and *exo* transition structures were divided into two general categories: those common to both the *endo* and *exo* transition structures, and those only observed in one of the transition structures. It was found that interactions shared between the transition structures contributed 52.0 kcal/mol of steric energy to the *endo* transition state and 56.0 kcal/mol to the *exo* transition state, which accounts for 4.0 of the 10.4 kcal/mol difference (38%). This proportion of the steric interaction can be attributed primarily to the difference between the 1,3-dipole N-C pi-system (1-2) and the pi-bond (3-4) in the ethylene portion of styrene (Figure S116).

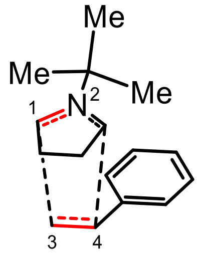

Figure S116. Shared steric interactions for both endo and exo shown in red.

Specifically, the steric analysis measures this interaction in the *endo* transition state as having a steric exchange value of 9.6 kcal/mol, while the *exo* transition state has a corresponding value of 13.6 kcal/mol, giving this steric interaction a difference of 4.0 kcal/mol between the *endo* and *exo* transition states. While this is not the only energetic difference between shared steric interactions between the *endo* and *exo* transition structures, it is the most significant (xx%) and the other differences effectively cancel out.

The steric interactions that are not shared among the *endo* and *exo* transition states are of significant interest and can be categorized by which parts of the ylide and styrene are repelling each other. To gain further insight, steric interactions between the ylide and styrene steric were further categorized into five possible intramolecular interaction groups as shown below (Figure S117) for the unique steric interactions.

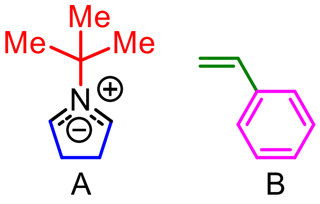

Figure S117. (a) Three t-butyl pyrrolidine ylide categories for steric analysis: Red is the t-butyl group, black is the 1,3-dipole, and blue is the ethylene linker. (b) Two styrene categories for steric analysis: pink is the phenyl component and green is the ethylene fragment. All six intramolecular combinations are analyzed.

Shown in Tables S4 and S5 are the computed steric interactions not shared by the *endo* and *exo* transition structures.

Table S4. Endo transition structure unshared steric interactions (kcal/mol)

	ethylene	phenyl	Total
t-butyl	1.9	0.0	1.9
1,3-dipole	2.2	2.1	4.3
ethylene linker	0.8	7.2	8.0
Total	5.0	9.3	14.2

Table S5. Exo transition structure unshared steric interactions (kcal/mol)

	ethylene	phenyl	Total
t-butyl	0.0	8.3	8.3
1,3-dipole	3.4	4.0	7.4
ethylene linker	4.9	0.0	4.9
Total	8.3	12.3	20.6

When directly comparing the steric interaction between the exo and endo transition states we see that the total interactions between the 1,3-dipole (+3.1 kcal/mol) and the ethylene linker (-3.1 kcal/mol) cancel out.

Table S6. Difference in steric interactions (kcal/mol) [exo-endo]

	ethylene	phenyl	Total
t-butyl	-1.9	8.3	6.4
1,3-dipole	1.2	1.9	3.1

ethylene linker	4.1	-7.2	-3.1
Total	3.4	3.0	6.4

Thus, we can conclude that steric interactions with the *t*-butyl group and styrene (phenyl) are responsible for the remaining 6.4 kcal/mol (62%) of the 10.4 kcal/mol steric interaction difference (Figure S118). In summary, we find that the *t*-butyl interactions with the phenyl component of styrene present in the *exo* transition structure (not in the *endo* transition structure) account for the majority of the steric difference between the two transition states which correlates with the observed experimental *endo* selectivity.

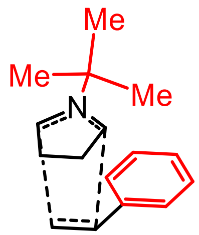

Figure S118. Major steric effects unique to exo transition structure shown in red.

References

(1) Nibbs, A. E.; Montgomery, T. D.; Zhu, Y.; Rawal, V. H. Access to Spirocyclized Oxindoles and Indolenines via Palladium-Catalyzed Cascade Reactions of Propargyl Carbonates with 2-Oxotryptamines and Tryptamines. *The Journal of Organic Chemistry* **2015**, *80* (10), 4928-4941. DOI: 10.1021/acs.joc.5b00277.

(2) Love, B. E.; Jones, E. G. The Use of Salicylaldehyde Phenylhydrazone as an Indicator for the Titration of Organometallic Reagents. *The Journal of Organic Chemistry* **1999**, *64* (10), 3755-3756. DOI: 10.1021/jo982433e.

(3) Bottini, A. T.; Roberts, J. D. Nuclear Magnetic Resonance Spectra. Nitrogen Inversion Rates of N-Substituted Aziridines (Ethylenimines)1. *Journal of the American Chemical Society* **1958**, *80* (19), 5203-5208. DOI: 10.1021/ja01552a048.

(4) Wei, D.; Netkaew, C.; Wu, J.; Darcel, C. Iron-catalyzed hydrosilylation of diacids in the presence of amines: a new route to cyclic amines. *ChemCatChem* **2020**, *12* (21), 5449-5455. DOI: https://doi.org/10.1002/cctc.202000881.

(5) Oakdale, J. S.; Kwisnek, L.; Fokin, V. V. Selective and Orthogonal Post-Polymerization Modification using Sulfur(VI) Fluoride Exchange (SuFEx) and Copper-Catalyzed Azide–Alkyne Cycloaddition (CuAAC) Reactions. *Macromolecules* **2016**, *49* (12), 4473-4479. DOI: 10.1021/acs.macromol.6b00101.

(6) Takeshima, H.; Satoh, K.; Kamigaito, M. Bio-based vinylphenol family: Synthesis via decarboxylation of naturally occurring cinnamic acids and living radical polymerization for functionalized polystyrenes. *Journal of Polymer Science* **2020**, *58* (1), 91-100. DOI: https://doi.org/10.1002/pola.29453.

(7) Toneto Novaes, L. F.; Martins Avila, C.; Pelizzaro-Rocha, K. J.; Vendramini-Costa, D. B.; Pereira Dias, M.; Barbosa Trivella, D. B.; Ernesto de Carvalho, J.; Ferreira-Halder, C. V.; Pilli, R. A. (−)-Tarchonanthuslactone: Design of New Analogues, Evaluation of their Antiproliferative Activity on Cancer Cell Lines, and Preliminary Mechanistic Studies. *ChemMedChem* **2015**, *10* (10), 1687-1699. DOI: https://doi.org/10.1002/cmdc.201500246.

(8) Felix, A. M. Cleavage of protecting groups with boron tribromide. *The Journal of Organic Chemistry* **1974**, *39* (10), 1427-1429. DOI: 10.1021/jo00926a025.

(9) Verma, P.; Truhlar, D. G. Status and Challenges of Density Functional Theory. *Trends in Chemistry* **2020**, *2* (4), 302-318.

(10) Zhao, Y.; Truhlar, D. G. The M06 suite of density functionals for main group thermochemistry, thermochemical kinetics, noncovalent interactions, excited states, and transition elements: two new functionals and systematic testing of four M06-class functionals and 12 other functionals. *Theoretical Chemistry Accounts* **2008**, *120* (1), 215-241. DOI: 10.1007/s00214-007-0310-x.

(11) Dunning, T. H., Jr. Gaussian basis sets for use in correlated molecular calculations. I. The atoms boron through neon and hydrogen. *Journal of Chemical Physics* **1989**, *90* (2), 1007-1023.

(12) Jensen, J. H. *Molecular Modeling Basics*; CRC Press, 2010. DOI: https://doi.org/10.1201/9781420075274

(13) Zhao, Y.; Truhlar, D. G. The M06 suite of density functionals for main group thermochemistry, thermochemical kinetics, noncovalent interactions, excited states, and transition elements: two new functionals and systematic testing of four M06-class functionals and 12 other functionals. *Theorectical Chemistry Accounts* **2008**, *120* (1-3), 215-241.

(14) Neal, M. J.; Hejnosz, S. L.; Rohde, J. J.; Evanseck, J. D.; Montgomery, T. D. Multi-Ion Bridged Pathway of N-Oxides to 1,3-Dipole Dilithium Oxide Complexes. *J. Org. Chem.* **2021**, *86* (17), 11502-11518.

(15) Glendening E. G., B., J. K., Reed A. E., Carpenter J.E., Bohmann J. A., Morales C. M., Karafiloglou P., Landis C. R., Weinhold F. NBO 7.0. Theoretical Chemistry Institute, University of Wisconsin, Madison, WI 2018.

(16) Dunning, T. H., Jr. Gaussian basis sets for use in correlated molecular calculations. I. The atoms boron through neon and hydrogen. *The Journal of Chemical Physics* **1989**, *90* (2), 1007-1023. DOI: 10.1063/1.456153 (acccessed 4/4/2024).

(17) Miertuš, S.; Scrocco, E.; Tomasi, J. Electrostatic interaction of a solute with a continuum. A direct utilizaion of AB initio molecular potentials for the prevision of solvent effects. *Chemical Physics* **1981**, *55* (1), 117-129. DOI: https://doi.org/10.1016/0301-0104(81)85090-2.
(18) Mennucci, B. Polarizable continuum model. *WIREs Computational Molecular Science* **2012**, *2* (3), 386-404. DOI: https://doi.org/10.1002/wcms.1086.
